# Visible light induced cooperative carbonylation and (hetero)aryl migration: synthesis of multi-carbonyl compounds†

**DOI:** 10.1039/d4sc03221g

**Published:** 2024-08-14

**Authors:** Hefei Yang, Yuanrui Wang, Le-Cheng Wang, Xiao-Feng Wu

**Affiliations:** a Dalian National Laboratory for Clean Energy, Dalian Institute of Chemical Physics, Chinese Academy of Sciences 116023 Dalian Liaoning China xwu2020@dicp.ac.cn; b Leibniz-Institut für Katalyse e.V. 18059 Rostock Germany Xiao-Feng.Wu@catalysis.de

## Abstract

Carbonylative transformation represents one of the most straightforward procedures for the synthesis of carbonyl-containing compounds. However, the carbonylative procedure toward 1,4-diketones is still limited which are key moieties with potent applications in various areas. Herein, we report a new strategy for the synthesis of multi-carbonyl compounds containing a 1,4-diketone skeleton through remote heteroaryl migration of traditionally restricted 1,3-migratory substrates utilizing carbon monoxide (CO) as the C1 synthon and diazonium compounds as the starting material.

## Abstract 2

A new carbonylative strategy for the synthesis of 1,4-diketones has been developed.

## Graphical abstract

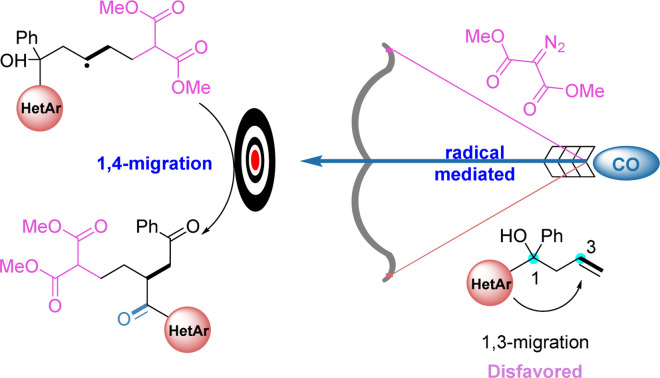

## Introduction

**Fig. 1:**
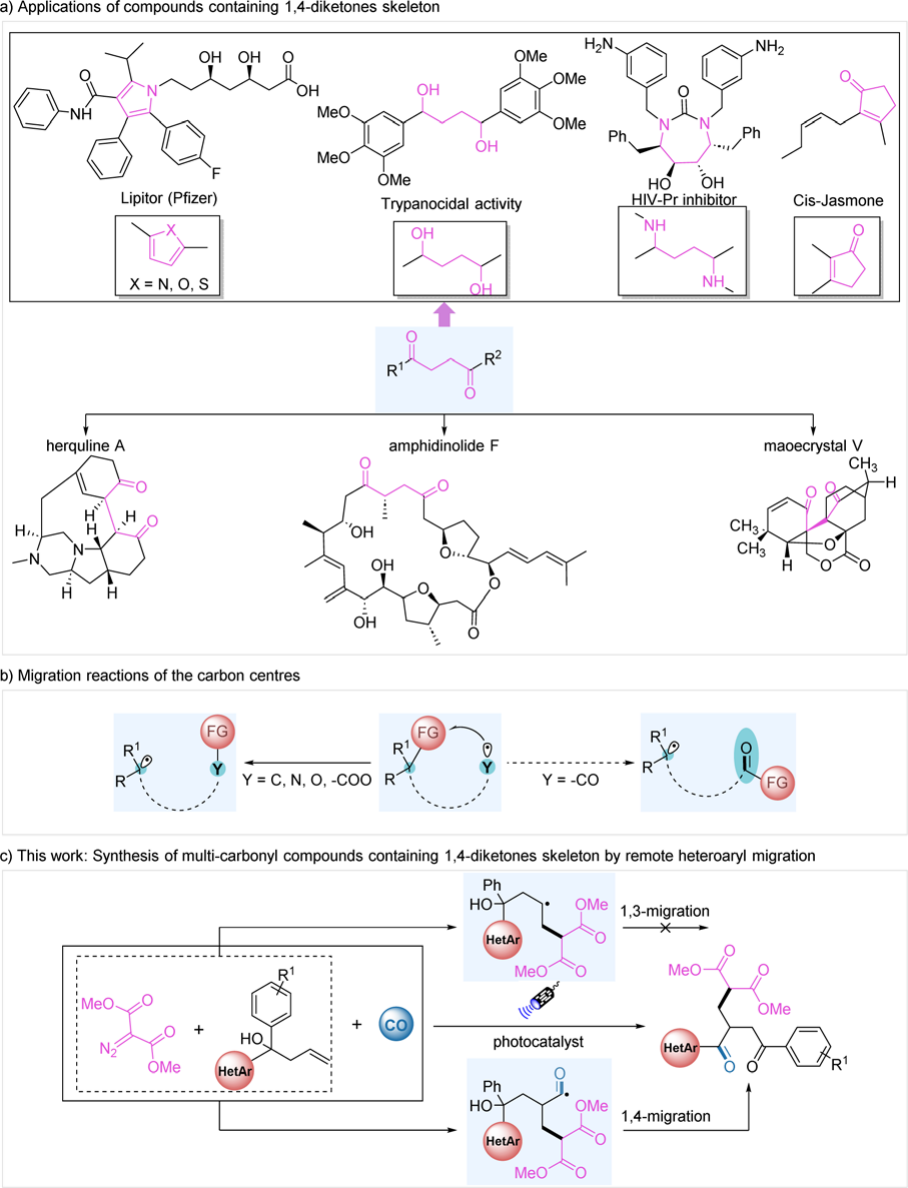
Study on multi-carbonyl compounds containing a 1,4-diketone skeleton.

Carbonyl-containing compounds possess versatile reactive sites and are privileged building blocks for the construction of a wide range of useful chemicals. Among them, 1,4-diketones are widely used as synthetic precursors for the assembly of various bioactive compounds, including bioactive molecules such as pyrroles, furans, thiophenes, diols, diamines, cyclopentenones, *etc.*^1^ Many natural products also contain a 1,4-diketone skeleton. Examples include herquline A,^2^ amphidinolide F^3^ and maoecrystal V (Fig. 1(a)).^4^ Due to the recognized importance of the carbonyl group, it is therefore particularly important to develop new methods to produce these valuable multi-carbonyl compounds in synthetic chemistry.

Compared with using complexed molecules for constructing challenging natural product skeletons, the use of simple molecules is more attractive in synthetic chemistry based on their readily availability. Carbon monoxide (CO) serves as a cost-effective and readily available C1 source, making it a significant synthon in carbonylation reactions.^5^ Radical mediated carbonylation is attracting growing interest because of its versatility for radical generation and relatively mild reaction conditions. Over the past few decades, there has been a systematic investigation into the radical-promoted difunctionalization reactions of unactivated alkenes, including remote functional group migration.^6^ Since the first report of radical-mediated aryl migration by Wieland in 1911,^7^ migration reactions have been extensively studied but are mainly limited to migration between two carbon atoms. In 1995, Kim *et al.* reported the migration of 1,2-aryl groups from carbon to N-centered radicals derived from azido groups.^8^ This type of reaction was subsequently enriched by Shi and Nevado *et al.*^9^ Migration reactions from the carbon center to the oxygen center and from the carbon center to carboxylated radicals have also developed over time.^10^ However, migration from carbon to acyl radicals has not been well achieved and needs to be explored and investigated (Fig. 1(b)).

Due to their high reactivity and wide range of applications, diazo compounds are highly regarded as carbene precursors under transition metal catalysis or direct light irradiation.^11^ In contrast, relevant research studies on the reactions of diazo compounds as radical precursors were less reported.^12^ We envision that the conversion of diazo compounds into carbon radicals through the proton-coupled electron transfer process^13^ might be a solution. Additionally, 1,3-migrating substrates are limited for remote functional group migration. Herein, we describe a mild and efficient protocol for the heteroaromatic group migration and carbonylation of unactivated alkenes with carbon monoxide and a variety of useful multi-carbonyl compounds containing a 1,4-dicarbonyl skeleton were prepared in good yields in general (Fig. 1(c)).

## Results and discussion

To realise this transformation, we studied the reaction using dimethyl 2-diazomalonate (1a) and benzothiazole-substituted tertiary alcohol (2a) as model substrates for the heteroaromatic group migration and carbonylation in the presence of 1.0 mol% *fac*-Ir(ppy)_3_. After exposure to CO at 40 bar with blue LEDs in *N*,*N*-dimethylformamide (DMF) at room temperature for 24 hours, the desired product 3a was obtained in 72% yield (Table 1, entry 1). Control reactions revealed that omission of the photocatalyst *fac*-Ir(ppy)_3_, or light, gave very low product 3a formation. Moreover, replacing *fac*-Ir(ppy)_3_ with 4CzIPN or changing the power of the lamp resulted in a decrease in the yield of the desired product 3a. Diverse solvents were examined for the reaction, and the desired product was identified, although the outcomes were found to be unsatisfactory (for details, please refer to the ESI† for more information).

**Table 1:** Optimization of the reaction conditions^a^

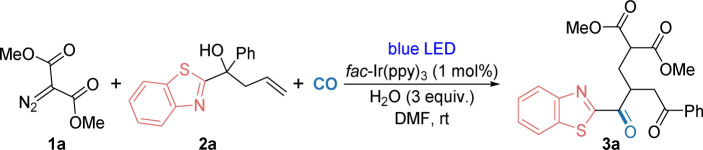		
Entry	Variation from the standard reaction conditions	Yield [%]
1	None	72
2	No PC	9
3	No light	2
4	4CzIPN instead of *fac*-Ir(ppy)_3_	53
5	7 W instead of 15 W	60
6	Other solvents instead of DMF	<60
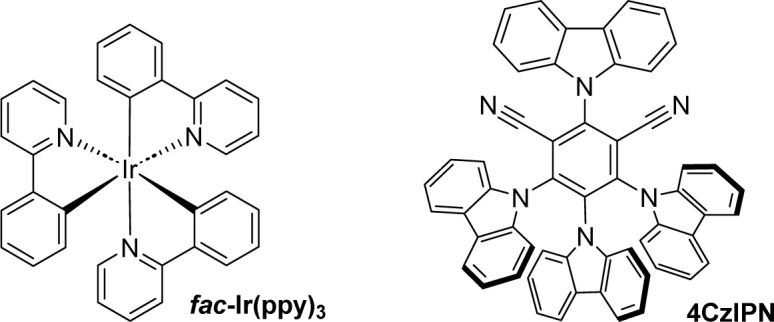		

a Reaction conditions: 1a (0.6 mmol), 2a (0.2 mmol), photocatalyst (1 mol%), H_2_O (3 equiv.), DMF (2 mL) with blue LEDs (15 W) at room temperature for 24 h under CO (40 bar). Isolated yields.

**Fig. 2:**
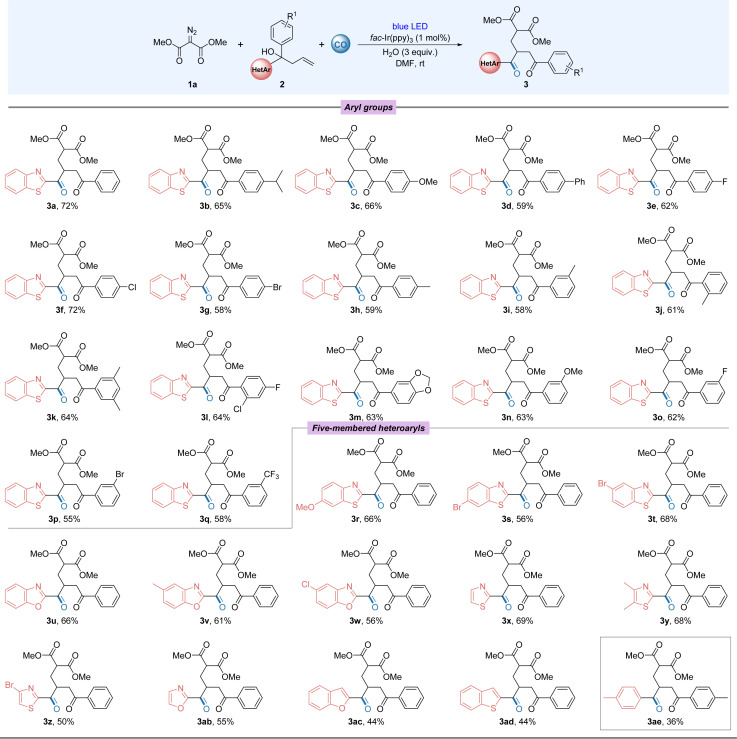
Substate scope: testing of the tertiary alcohols. Reaction conditions: 1a (0.6 mmol), 2 (0.2 mmol), *fac*-Ir(ppy)_3_ (1 mol%), H_2_O (3 equiv.), DMF (2 mL) with blue LEDs (15 W) at room temperature for 24 h under CO (40 bar), isolated yield.

After mastering the optimal conditions for the reaction, we began to examine the compatibility of this transformation with a series of different benzothiazole-substituted tertiary alcohols and dimethyl 2-diazomalonate (Fig. 2). The overall experimental results showed that the benzothiazole group preferentially migrated chemoselectively over the aryl group. When different electron-donating or electron-absorbing groups were used to replace the aryl group in the substrate, the carbonylated products were obtained in good yields with little variation in yields (3a–3g and 3n–3q). Surprisingly, halides, especially chlorides (3f), were compatible in this remote migration reaction, providing a basis for further modifications of the substrate. By changing the substitution site on the benzene ring, we also tested the tolerance of the reaction to spatial effects, which proved to have no significant effect on the reaction yields (3h–3j). When the aryl group is replaced by a double group, the target product can still be obtained in good yields (3k–3m). The tolerance of the reaction to aromatic heterocycles other than benzothiazole groups was then investigated. The migration of various substituted benzothiazoles proceeded smoothly, with electronic and spatial effects largely unaffected (3r–3t). In addition to substituted benzothiazoles, benzoxazoles and thiazoles, among others, also reacted smoothly and good yields were obtained (3u–3ab). Benzofuranyl and benzothiophene groups were slightly sensitive to reaction conditions and the established products were obtained in 44% yields (3ac–3ad). To our surprise, diaryl-substituted alcohols were also able to normalise the migration reaction. However, because of the migration efficiency, the target product was obtained in 36% yield (3ae). It's also worth mentioning that the reactions with 1-(benzo[*d*]thiazol-2-yl)-1-phenylpent-4-en-1-ol and 1-(benzo[*d*]thiazol-2-yl)-1-phenylprop-2-en-1-ol with diazo compound toward 1,5- and 1,3-aryl migration failed without the desired product being detectable. In the cases of 1-phenyl-1-(pyridin-2-yl)but-3-en-1-ol and 1-(1-methyl-1*H*-indol-2-yl)-1-phenylbut-3-en-1-ol, no targeted product could be detected.

**Fig. 3:**
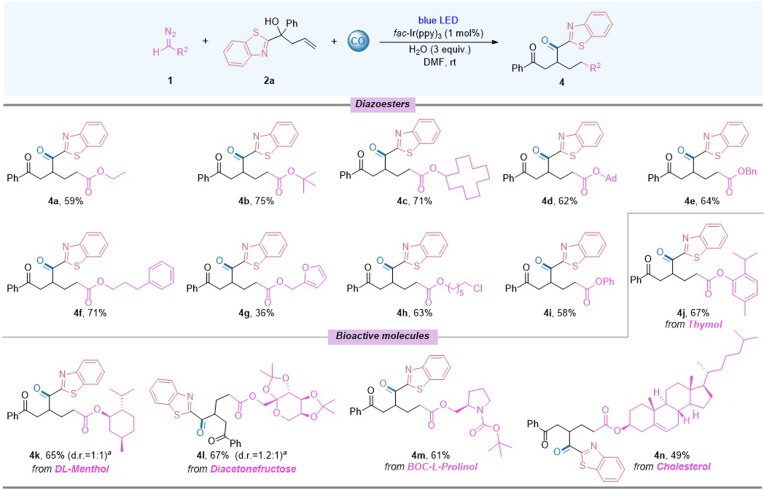
Substrate scope: testing of diazo compounds. Reaction conditions: 1 (0.6 mmol), 2a (0.2 mmol), *fac*-Ir(ppy)_3_ (1 mol%), H_2_O (3 equiv.), DMF (2 mL) with blue LEDs (15 W) at room temperature for 36 h under CO (40 bar), isolated yield.^*a*^ The d.r. value was determined by ^1^H NMR.

In order to further expand the product diversity of the reaction, diazo compounds were tested subsequently and decreased conversion was observed in some cases (Fig. 3). Considering the reduced activity of the diazo compounds compared to dimethyl 2-diazomalonate, the reaction was slightly optimised with an appropriate extension of the reaction time. Various alkyl alcohol substituted diazo esters were tested first and the results showed good compatibility (4a–4i). When the substituent group was a heterocycle, it showed sensitivity to the reaction and the target product could only be obtained in 36% yield (4g). Finally, we attempted to introduce various biomolecules into the reaction products and the corresponding products were obtained in moderate to good yields (4j–4n). Diazoacetylbenzene was also tested, but low yield was obtained.

**Fig. 4:**
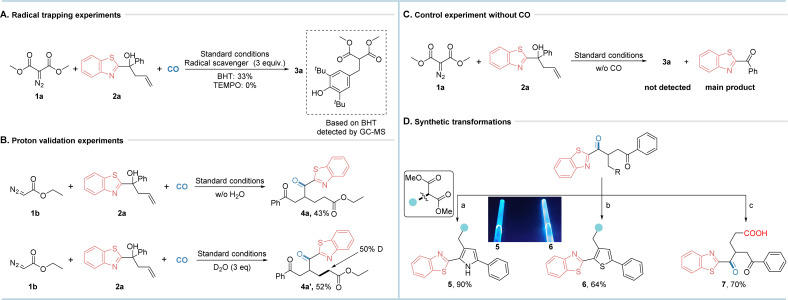
Control experiments and synthetic applications.

To better understand how the migration reaction occurs, we performed several control experiments as shown in Fig. 4. The model reaction was inhibited when a radical scavenger was added to the reaction under the standard conditions. In the presence of BHT (butylated hydroxytoluene), radical scavenging by-products were detected by GC-MS. The results of these radical quenching experiments suggest that the reaction may involve radical intermediates.

Additional control experiments were also carried out to obtain further mechanistic information. During the optimization of the reaction, it was found that the extra addition of H_2_O favored the conversion of diazonium compounds which promoted the reaction. To investigate the role of H_2_O, the experimental results were monitored by controlling the addition of H_2_O. When H_2_O was not present in the reaction system, the target product was obtained in 43% yield. After addition of 3 equivalents of D_2_O, the established product was obtained in a yield of 52% and 50% D was detected. The experimental results showed that the protons in the formation of carbon radicals from diazonium compounds partially originate from water in the reaction system. When carbon monoxide was not present in the system, the modelled reaction did not occur and only significant amounts of by-products were detected. This indicates that carbon monoxide plays an important role in the reaction by acting as a linker type of promoter.

To further demonstrate the value of the synthetic method, the products generated were further transformed. 1,4-Dicarbonyl compounds were used as synthetic building blocks for biologically active heterocycle preparation, and the corresponding pyrrole derivative 5 and thiophene derivative 6 were obtained in excellent yields using product 3a as a template substrate. Surprisingly, both products 5 and 6 showed excellent fluorescence under 365 nm light, which may further expand their applicability. The ester group of product 4a can be easily hydrolysed to afford the carboxylic acid derivative 7. With the research on carboxylic acid derivatives in recent years, a number of decarboxylation halogenations, decarboxylation arylations, decarboxylation borylations, decarboxylation unsaturations, *etc.* can be obtained by modifying the carboxylic acid group.^14^ These expressed reaction advantages may have potential applications in the future for drug developments. Concerning large-scale synthesis, this method can be easily expanded by performing the reaction with several vials parallelly in the same autoclave which is even more stable with the reaction outcome.

**Fig. 5:**
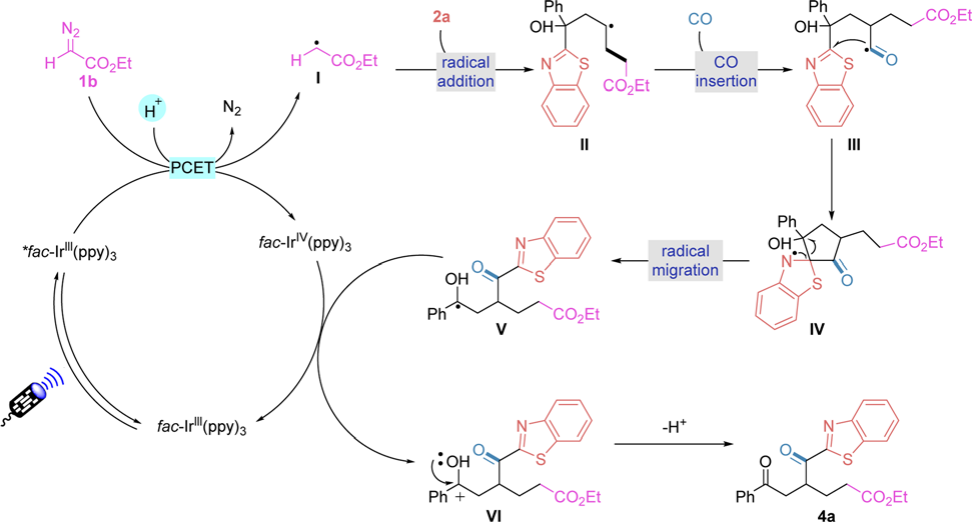
Proposed mechanism.

Based on the experimental results and literature,^15^ we proposed a possible reaction pathway for this transformation (Fig. 5). Initially, the diazonium compound was reduced using an excited state catalyst *fac-Ir(ppy)_3_ and undergoes a PCET process to release nitrogen to obtain alkyl radical I. Addition of alkyl radical I to the unactivated olefinic substrate 2a yields a new carbon radical intermediate II. At this point, the radical intermediate II can capture carbon monoxide under a carbon monoxide atmosphere to generate acyl radical III. Subsequent intramolecular addition of heteroaryl groups provides intermediate IV, which undergoes radical mobilisation to afford intermediate V. The final sequence yields the established product 4a*via* SET oxidation and deprotonation with simultaneous regeneration of the base state photocatalyst.

## Conclusions

In conclusion, we developed a new preparation strategy for multi-carbonyl compounds containing a 1,4-diketone skeleton. The reaction proceeds *via* radical-mediated difunctionalisation of unactivated alkenes using carbon monoxide (CO) as the C1 linker. By this procedure, remote heteroaryl migration of traditionally restricted 1,3-migrating substrates has been successfully achieved utilizing carbon monoxide (CO) as the C1 synthon, providing a new pathway for the subsequent use and study of carbon monoxide. In the future, efforts on 1,3-migration, 1,5-migration and others should be made.

## Author contributions

X.-F. W. conceived and directed the project. H. Y. performed all the experiments. X.-F. W. and H. Y. wrote and revised the manuscript. H. Y., Y. W., and L. C. W. prepared the ESI.†

## Conflicts of interest

There are no conflicts to declare.

## Supplementary Material

SC-015-D4SC03221G-s001

## Data Availability

The data supporting this article have been included as part of the ESI.†
