# Supplementary material for: Visible light induced cooperative carbonylation and (hetero)aryl migration: synthesis of multi-carbonyl compounds
Source: Chem Sci. 2024 Aug 14;15(35):14304–9. doi: 10.1039/d4sc03221g (PMC11325186; doi:10.1039/d4sc03221g)
Supplement: SC-015-D4SC03221G-s001 [file SC-015-D4SC03221G-s001.pdf]

Supplementary Information (SI) for Chemical Science.
This journal is © The Royal Society of Chemistry 2024

Supporting Information

Contents

1. General Information S1
2. Optimization of the Reaction Conditions S2
3. General Procedure S4
4. Control Experiments S5
5. Synthetic transformations S8
6. Spectroscopic Data of Products S9
7. The NMR Spectrum S30

1. General Information

Reagents, solvents, and analytical methods:

Unless otherwise noted, all reactions were carried out under carbon monoxide or nitrogen atmosphere. All reagents were from commercial sources and used as received without further purification. All solvents were dried by standard techniques and distilled prior to use. Column chromatography was performed on silica gel (200-300 meshes) using petroleum ether (bp. 60~90 °C), dichloromethane and ethyl acetate as eluent. All NMR spectra were recorded at ambient temperature using Bruker Avance III 400 MHz NMR (^{1}H, 400 MHz; ^{13}C {^{1}H}, 101 MHz, ^{19}F 376 MHz), Bruker AVANCE III HD 700 MHz NMR spectrometers (^{1}H, 700 MHz; ^{13}C{^{1}H}, 176 MHz). 1H NMR chemical shifts are reported relative to TMS and were referenced via residual proton resonances of the corresponding deuterated solvent ($CDCl_3$: 7.26 ppm; d_6-DMSO: 2.50 ppm) whereas ^{13}C{^{1}H} NMR spectra are reported relative to TMS via the carbon signals of the deuterated solvent ($CDCl_3$: 77.0 ppm; d_6-DMSO: 39.5 ppm. Data for ^{1}H are reported as follows: chemical shift (δ ppm), multiplicity (s = singlet, d = doublet, t = triplet, q = quartet, dd (doublet of doublets), dt (doublet of triplets), qd (quartet of doublets), quint = quintet, m = multiplet, br = broad), coupling constant (Hz), and integration. All ^{13}C NMR spectra were broad-band ^{1}H decoupled. All reactions were monitored by GC-FID or NMR analysis. HRMS data was obtained with Micromass HPLC-Q-TOF mass spectrometer (ESI-TOF) or Agilent 6540 Accurate-MS spectrometer (Q-TOF).

Because of the high toxicity of carbon monoxide, all the reactions should be performed in an autoclave. The laboratory should be well-equipped with a CO detector and alarm system.

2. Optimization of the Reaction Conditions

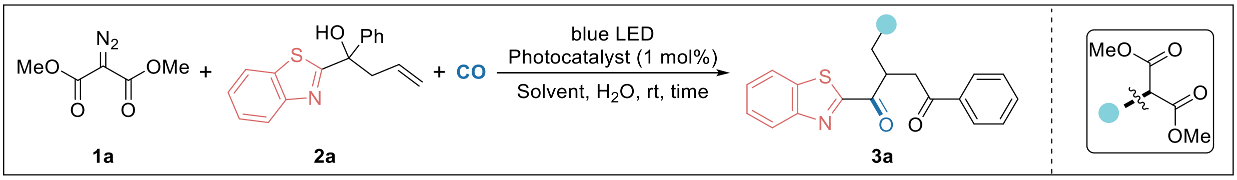

Table S1. **Optimization of solvent.**

Entry	Solvent	Yield (%)
1	DMF	68
2	DMAc	60
3	EA	49
4	Acetone	50
5	MeCN	44
6	DCE	33

Reaction conditions: **1a** (3 equiv.), **2a** (0.2 mmol), *fac*-Ir(ppy)$_3$ (1 mol%), CO (40 bar) in Solvent (2 mL), irradiation with a 15 W blue LED at rt for 24 hours. The yields were determined by islolated.

Table S2. **Optimization of photocatalysts.**

Entry	Photocatalyst	Yield (%)
1	*fac*-Ir(ppy)$_3$	68
2	4CzIPN	53
3	[Ir(dtbbpy)(ppy)$_2$]PF$_6$	57
4	[Ir(dF(CF$_3$)ppy)$_2$(dtbbpy)]PF$_6$	64
5	[Ru(bpy)$_3$]Cl$_2$ • 6H$_2$O	Trace
6	No PC	9

Reaction conditions: **1a** (3 equiv.), **2a** (0.2 mmol), Photocatalyst (1 mol%), CO (40 bar) in DMF (2 mL), irradiation with a 15 W blue LED at rt for 24 hours. The yields were determined by islolated.

Table S3. **Optimization of blue LED.**

Entry	blue LED (w)	Yield (%)
1	No light	2
2	7	60
3	15	68
4	45	48

Reaction conditions: **1a** (3 equiv.), **2a** (0.2 mmol), *fac*-Ir(ppy)$_3$ (1 mol%), CO (40 bar) in DMF (2 mL), irradiation with a blue LED at rt for 24 hours. The yields were determined by islolated.

Table S4. **Optimization of H_2O.**

Entry	H_2O (equiv)	Yield (%)
1	1	63
2	2	66
3	3	72

Reaction conditions: **1a** (3 equiv.), **2a** (0.2 mmol), *fac*-Ir(ppy)$_3$ (1 mol%), H_2O (x equiv), CO (40 bar) in DMF (2 mL), irradiation with a 15 W blue LED at rt for 24 hours. The yields were determined by islolated.

3. General Procedure

General carbonylation I

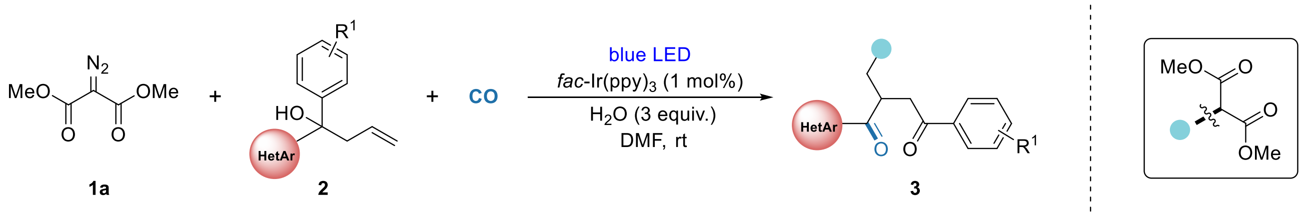

To a dried 4 mL vial equipped with a magnetic stir bar was added *fac*-Ir(ppy)$_3$ (1.3 mg, 1 mol%), **2** (0.2 mmol, 1.0 equiv.), The vial was closed with a Teflon septum and cap and connected to the atmosphere via a needle. The vial was evacuated under vacuum and recharged with argon for three times. After **1a** (3 equiv., 0.6 mmol), H_2O (3 equiv., 0.6 mmol), and dry DMF (2 mL). were added with a syringe under nitrogen atmosphere, The vials (usually 8) were placed on an S6 alloy plate, which was transferred into an autoclave with two inserted quartz-glass windows (2 x 10 cm^2). After the autoclave was flushed three times with CO, it was pressurised with 40 atm of CO and then irradiated with two 15 W blue LEDs (450-460 nm) at room temperature for 24 h. Upon completion of the reaction, the pressure was carefully released, the reaction was diluted with 20 mL EtOAc. Then the reaction washed with brine and extracted with EtOAc 3x15 mL, then dried over Na_2SO_4. The solvent was removed under reduced pressure. The crude product was purified by flash chromatography on silica gel (silica: 200-300 mesh, eluent: petroleum ether/ethyl acetate (10:1-1:1, v/v) to afford the corresponding pure product **3**.

General carbonylation II

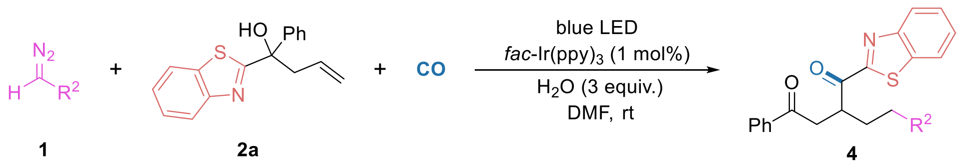

To a dried 4 mL vial equipped with a magnetic stir bar was added *fac*-Ir(ppy)$_3$ (1.3 mg, 1 mol%), **2** (0.2 mmol, 1.0 equiv.), The vial was closed with a Teflon septum and cap and connected to the atmosphere via a needle. The vial was evacuated under vacuum and recharged with argon for three times. After **1a** (3 equiv., 0.6 mmol), H_2O (3 equiv., 0.6 mmol), and dry DMF (2 mL). were added with a syringe under nitrogen atmosphere, The vials (usually 8) were placed on an S6 alloy plate,

which was transferred into an autoclave with two inserted quartz-glass windows (2 x 10 cm^2). After the autoclave was flushed three times with CO, it was pressurised with 40 atm of CO and then irradiated with two 15 W blue LEDs (450-460 nm) at room temperature for 36 h. Upon completion of the reaction, the pressure was carefully released, the reaction was diluted with 20 mL EtOAc. Then the reaction washed with brine and extracted with EtOAc 3x15 mL, then dried over Na_2SO_4. The solvent was removed under reduced pressure. The crude product was purified by flash chromatography on silica gel (silica: 200-300 mesh, eluent: petroleum ether/ethyl acetate (10:1-1:1, v/v) to afford the corresponding pure product **4**.

4. Control Experiments

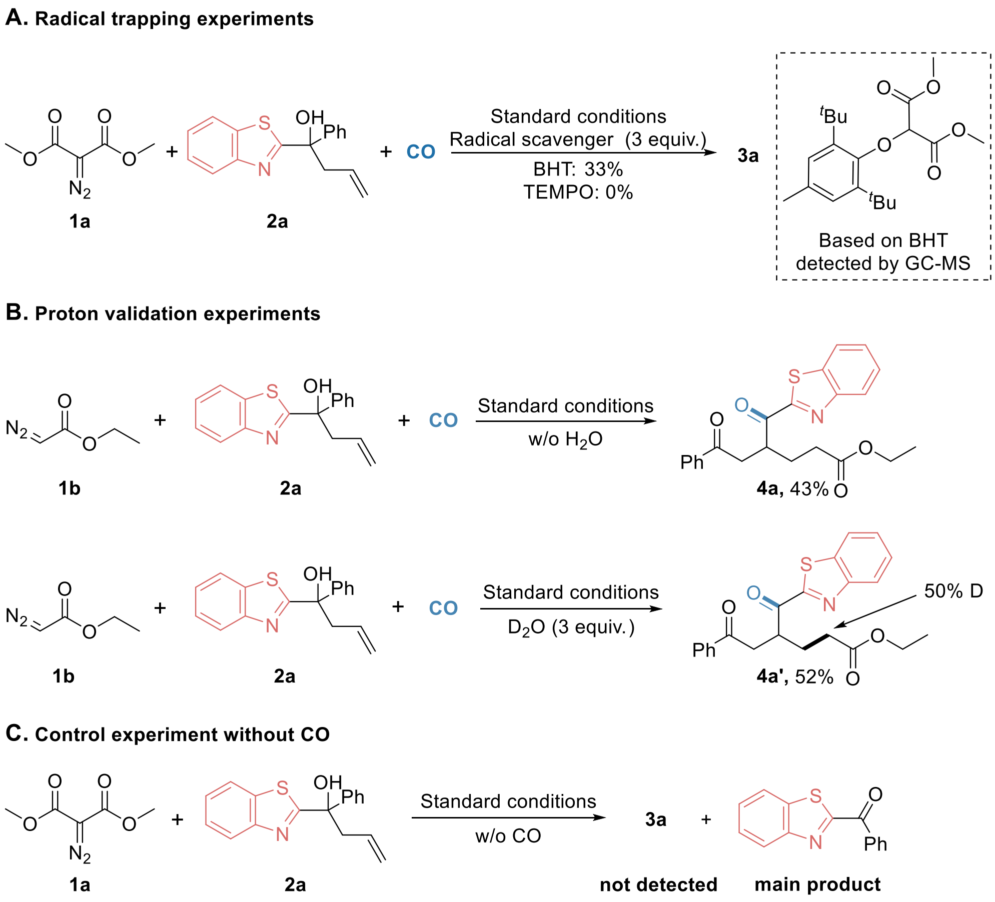

Eq A, Radical trapping experiments: To a dried 4 mL vial equipped with a magnetic stir bar was added *fac*-Ir(ppy)₃ (1.3 mg, 1 mol%), **2a** (0.2 mmol, 1 equiv.), radical scavenger (3 equiv.). The vial was closed with a Teflon septum and cap and connected to the atmosphere via a needle. The vial

was evacuated under vacuum and recharged with argon for three times. After **1a** (3 equiv., 0.6 mmol), H_2O (3 equiv., 0.6 mmol), and dry DMF (2 mL). were added with a syringe under nitrogen atmosphere, which was transferred into an autoclave with two inserted quartz-glass windows (2 x 10 cm^2). After the autoclave was flushed three times with CO, it was pressurised with 40 atm of CO and then irradiated with two 15 W blue LEDs (450-460 nm) at room temperature for 36 h. The sample of the reaction was tested by GC-MS and the radical-trapping products was detected by GC-MS.

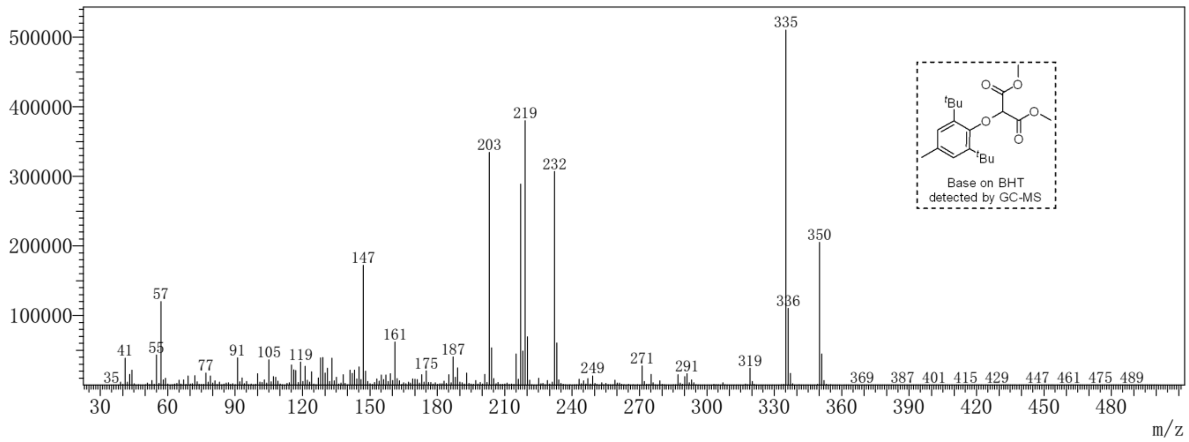

Eq B, Proton validation experiments: To a dried 4 mL vial equipped with a magnetic stir bar was added *fac*-Ir(ppy)$_3$ (1.3 mg, 1 mol%), **2a** (0.2 mmol, 1 equiv.), The vial was closed with a Teflon septum and cap and connected to the atmosphere via a needle. The vial was evacuated under vacuum and recharged with argon for three times. After **1b** (3 equiv., 0.6 mmol), D_2O (3 equiv., 0.6 mmol), and dry DMF (2 mL). were added with a syringe under nitrogen atmosphere, The vials (usually 8) were placed on an S6 alloy plate, which was transferred into an autoclave with two inserted quartz-glass windows (2 x 10 cm^2). After the autoclave was flushed three times with CO, it was pressurised with 40 atm of CO and then irradiated with two 15 W blue LEDs (450-460 nm) at room temperature for 36 h. Upon completion of the reaction, the pressure was carefully released, the reaction was diluted with 20 mL EtOAc. Then the reaction washed with brine and extracted with EtOAc 3x15 mL, then dried over Na_2SO_4. The solvent was removed under reduced pressure. The crude product was purified by flash chromatography on silica gel (silica: 200-300 mesh, eluent: petroleum ether/ethyl acetate (10:1-1:1, v/v) to afford the corresponding pure product **4a'**. The sample of deuterated product **4a'** was tested by 1H NMR.

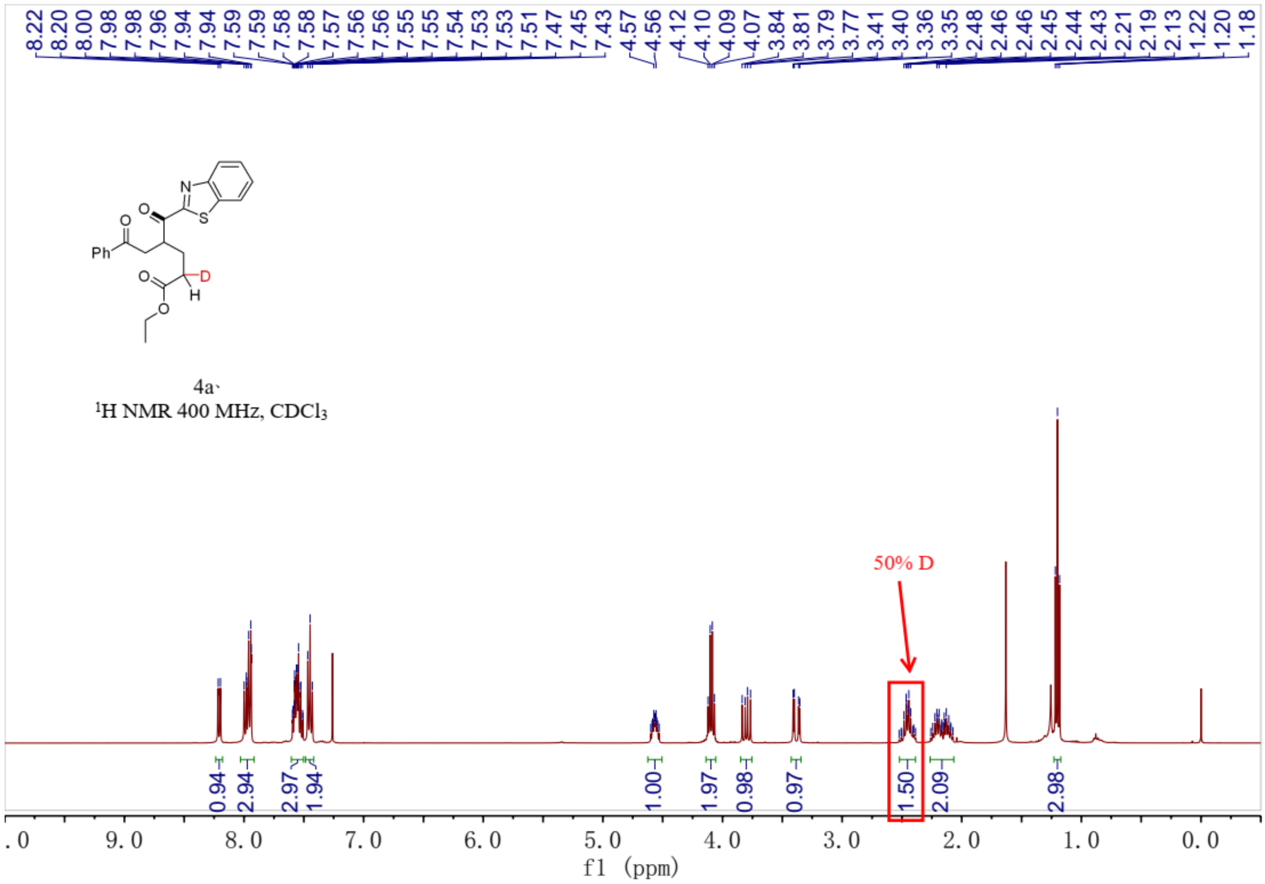

Eq C, Control experiment without CO: To a dried 4 mL vial equipped with a magnetic stir bar was added *fac*-Ir(ppy)$_3$ (1.3 mg, 1 mol%), **2a** (0.2 mmol, 1 equiv.). The vial was closed with a Teflon septum and cap and connected to the atmosphere via a needle. The vial was evacuated under vacuum and recharged with argon for three times. After **1a** (3 equiv., 0.6 mmol), H_2O (3 equiv., 0.6 mmol), and dry DMF (2 mL). were added with a syringe under nitrogen atmosphere, which was transferred into an autoclave with two inserted quartz-glass windows (2 x 10 cm^2). Without CO and then irradiated with two 15 W blue LEDs (450-460 nm) at room temperature for 36 h. The sample of the reaction was tested by GC-MS.

5. Synthetic transformations

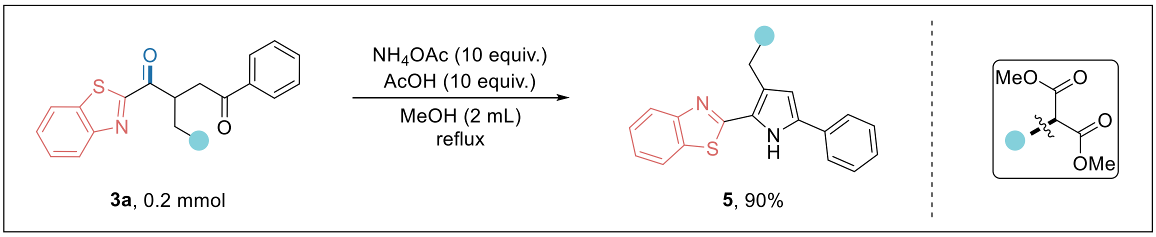

solution of **3a** (87.9 mg, 0.2 mmol) and ammonium acetate (154 mg, 20 mmol) , acetic acid (20 mmol) in MeOH was heated to 85 °C under nitrogen for 12 hours. Then EA (20 mL) was added and the reaction mixture was washed with water (20 mL) and saturated sodium bicarbonate solution (20 mL) and dried over sodium sulfate. The solvent of organic layer was removed under reduce pressure and the residue was purified by column chromatography (Hexane:EA = 5:1) to afford the product **5** as pale yellow oil (75.6 mg, 90%).

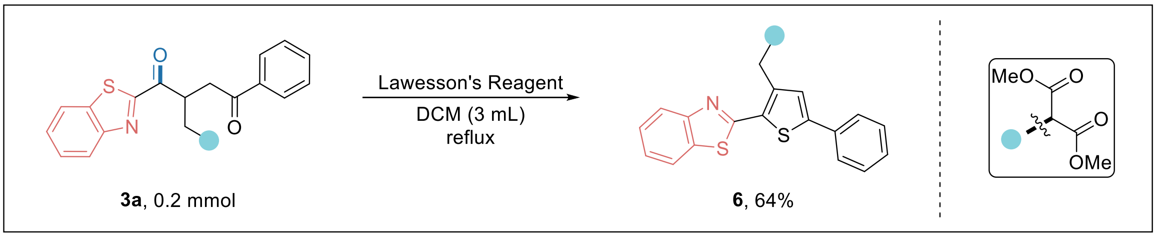

solution of **3a** (87.9 mg, 0.2 mmol) and Lawesson's Reagent (89 mg, 0.22 mmol) in dichloromethane (3 mL) was stirred at 40 °C under nitrogen for 6 hours. Then EA (20 mL) was added and the reaction mixture was washed with water (10 mL) and saturated sodium bicarbonate solution (10 mL) and dried over sodium sulfate. The solvent of organic layer was removed under reduce pressure and the residue was purified by column chromatography (Hexane:EA = 5:1) to afford the product **6** as yellow solid (55.6 mg, 64%).

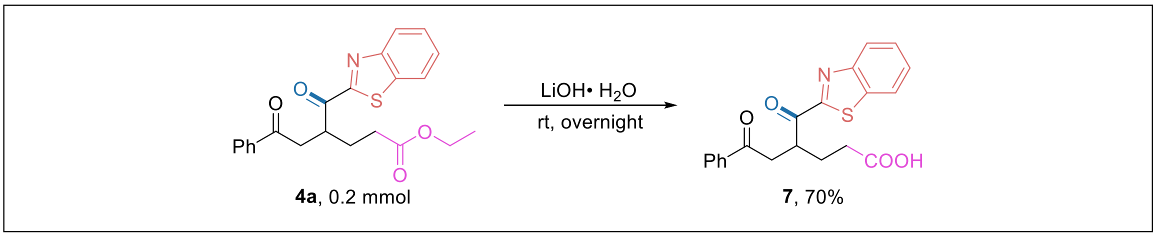

solution of **4a** (79.2 mg, 0.2 mmol) and LiOH·H_2O (12.6 mg, 0.3 mmol) in THF/water (1:1, 4 mL). The reaction mixture was stirred until the solid had dissolved and was then left overnight at room temperature. The solvents were removed in vacuo, and the residue was dissolved in water (3 mL). The resulting solution was washed with diethyl ether (2 mL). The aqueous layer was concentrated to half of its volume and then acidified with 30% hydrochloric acid (3 mL). The mixture was extracted with EtOAc for three times (3×10 mL). The organic phase was washed with water and dried over sodium sulphate. The solvent was removed under reduced pressure and the residue was purified by flash column chromatography (ethyl acetate = 1, Rf = 0.2) to give the product **7** as a yellow oil (51.2 mg, 70%).

6. Spectroscopic Data of Products

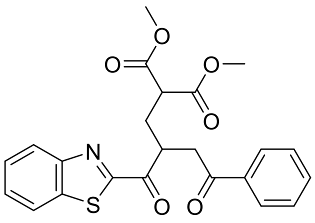

dimethyl 2-(2-(benzo[*d*]thiazole-2-carbonyl)-4-oxo-4-phenylbutyl)malonate (3a)

63.1 mg, 72% yield, slight yellow oil. Eluent: pentane/ethyl acetate = 5:1-3:1
^{1}H NMR (400 MHz, $CDCl_3$) δ 8.20 – 8.15 (m, 1H), 8.00 – 7.96 (m, 1H), 7.96 – 7.91 (m, 2H), 7.59 – 7.50 (m, 3H), 7.44 (t, *J* = 7.7 Hz, 2H), 4.55 (tt, *J* = 9.7, 5.0 Hz, 1H), 3.81 (dd, *J* = 18.0, 9.6 Hz, 1H), 3.75 (s, 3H), 3.66 (s, 3H), 3.60 (dd, *J* = 8.5, 6.4 Hz, 1H), 3.43 (dd, *J* = 17.9, 4.6 Hz, 1H), 2.50 – 2.36 (m, 2H).
^{13}C NMR (101 MHz, $CDCl_3$) δ 197.3, 196.7, 169.2, 169.2, 166.2, 153.6, 137.6, 136.0, 133.5, 128.7, 128.2, 127.7, 126.9, 125.7, 122.5, 52.8, 52.7, 49.7, 42.1, 40.0, 30.7.
HRMS (ESI-TOF) m/z: $[M + H]^+$ Calcd for $C_{23}H_{22}NO_6S$ 440.1162; Found: 440.1163.

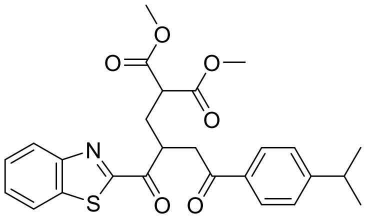

dimethyl 2-(2-(benzo[*d*]thiazole-2-carbonyl)-4-(4-isopropylphenyl)-4-oxobutyl)malonate (3b)

62.5 mg, 65% yield, slight yellow oil. Eluent: pentane/ethyl acetate = 5:1-3:1
^{1}H NMR (400 MHz, $CDCl_3$) δ 8.16 (d, *J* = 7.7 Hz, 1H), 7.97 (d, *J* = 8.1 Hz, 1H), 7.87 (d, *J* = 8.3 Hz, 2H), 7.58 – 7.49 (m, 2H), 7.29 (d, *J* = 8.2 Hz, 2H), 4.60 – 4.49 (m, 1H), 3.79 (dd, *J* = 12.1, 5.7 Hz, 1H), 3.74 (s, 3H), 3.66 (s, 3H), 3.59 (dd, *J* = 8.6, 6.4 Hz, 1H), 3.42 (dd, *J* = 17.9, 4.6 Hz, 1H), 2.99 – 2.90 (m, 1H), 2.48 – 2.36 (m, 2H), 1.25 (d, *J* = 6.9 Hz, 6H).
^{13}C NMR (101 MHz, $CDCl_3$) δ 197.0, 196.8, 169.2, 169.2, 166.2, 155.0, 153.6, 137.6, 133.9, 128.5, 127.7, 126.9, 126.7, 125.7, 122.4, 52.8, 52.7, 49.7, 42.1, 40.1, 34.3, 30.7, 23.7.
HRMS (ESI-TOF) m/z: $[M + H]^+$ Calcd for $C_{26}H_{28}NO_6S$ 482.1632; Found: 482.1635.

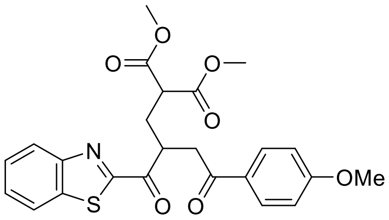

dimethyl 2-(2-(benzo[*d*]thiazole-2-carbonyl)-4-(4-methoxyphenyl)-4-oxobutyl)malonate (3c)

61.9 mg, 66% yield, slight yellow oil. Eluent: pentane/ethyl acetate = 5:1-3:1

^{1}H NMR (400 MHz, CDCl$_3$) δ 8.18 – 8.14 (m, 1H), 7.97 (d, *J* = 7.5 Hz, 1H), 7.93 – 7.88 (m, 2H), 7.58 – 7.48 (m, 2H), 6.90 (d, *J* = 8.9 Hz, 2H), 4.53 (tt, *J* = 9.7, 5.0 Hz, 1H), 3.84 (s, 3H), 3.79 – 3.73 (m, 4H), 3.65 (s, 3H), 3.58 (dd, *J* = 8.5, 6.4 Hz, 1H), 3.39 (dd, *J* = 17.7, 4.6 Hz, 1H), 2.48 – 2.34 (m, 2H).

^{13}C NMR (101 MHz, CDCl$_3$) δ 196.9, 195.8, 169.2, 169.2, 166.3, 163.8, 153.6, 137.6, 130.5, 129.1, 127.6, 126.9, 125.7, 122.4, 113.8, 55.5, 52.8, 52.7, 49.7, 41.9, 40.1, 30.7.

HRMS (ESI-TOF) m/z: $[M + H]^+$ Calcd for $C_{24}H_{24}NO_7S$ 470.1268; Found: 470.1263.

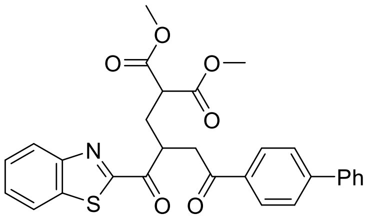

dimethyl 2-(4-([1,1'-biphenyl]-4-yl)-2-(benzo[d]thiazole-2-carbonyl)-4-oxobutyl)malonate (3d)

60.8 mg, 59% yield, slight yellow oil. Eluent: pentane/ethyl acetate = 5:1-3:1

^{1}H NMR (400 MHz, CDCl$_3$) δ 8.18 (d, *J* = 7.7 Hz, 1H), 8.00 (dd, *J* = 13.9, 4.7 Hz, 3H), 7.67 (d, *J* = 8.4 Hz, 2H), 7.62 (d, *J* = 7.3 Hz, 2H), 7.54 (ddd, *J* = 13.7, 7.8, 6.4 Hz, 2H), 7.46 (t, *J* = 7.4 Hz, 2H), 7.39 (t, *J* = 7.3 Hz, 1H), 4.64 – 4.53 (m, 1H), 3.85 (dd, *J* = 17.9, 9.5 Hz, 1H), 3.76 (s, 3H), 3.68 (s, 3H), 3.62 (dd, *J* = 8.5, 6.4 Hz, 1H), 3.47 (dd, *J* = 17.9, 4.6 Hz, 1H), 2.53 – 2.38 (m, 2H).

^{13}C NMR (101 MHz, CDCl$_3$) δ 196.9, 196.8, 169.2, 169.2, 166.2, 153.6, 146.1, 139.8, 137.6, 134.7, 129.0, 128.8, 128.3, 127.7, 127.3, 126.9, 125.7, 122.5, 52.8, 52.8, 49.7, 42.2, 40.1, 30.8.

HRMS (ESI-TOF) m/z: $[M + H]^+$ Calcd for $C_{29}H_{26}NO_6S$ 516.1475; Found: 516.1482.

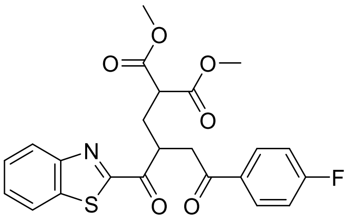

dimethyl 2-(2-(benzo[*d*]thiazole-2-carbonyl)-4-(4-fluorophenyl)-4-oxobutyl)malonate (3e)

57.6 mg, 62% yield, slight yellow oil. Eluent: pentane/ethyl acetate = 5:1-3:1
^{1}H NMR (400 MHz, CDCl$_3$) δ 8.16 (d, *J* = 7.5 Hz, 1H), 8.01 – 7.93 (m, 3H), 7.60 – 7.50 (m, 2H), 7.11 (t, *J* = 8.6 Hz, 2H), 4.59 – 4.49 (m, 1H), 3.81 – 3.74 (m, 4H), 3.66 (s, 3H), 3.59 (dd, *J* = 8.5, 6.4 Hz, 1H), 3.39 (dd, *J* = 17.9, 4.5 Hz, 1H), 2.49 – 2.34 (m, 2H).
^{13}C NMR (101 MHz, CDCl$_3$) δ 196.7, 195.8, 169.2, 169.1, 166.1, 166.0 (d, J_{C-F} = 255.4 Hz), 153.6, 137.6, 132.5 (d, J_{C-F} = 3.0 Hz), 130.9 (d, J_{C-F} = 9.4 Hz), 127.7, 126.9, 125.7, 122.5, 115.8 (d, J_{C-F} = 21.9 Hz), 52.8, 52.8, 49.6, 42.0, 40.0, 30.7.
^{19}F NMR (377 MHz, CDCl$_3$) δ -104.4.
HRMS (ESI-TOF) m/z: $[M + H]^+$ Calcd for $C_{23}H_{21}FNO_6S$ 458.1068; Found: 458.1069.

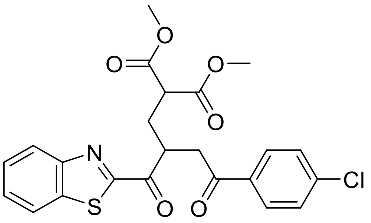

dimethyl 2-(2-(benzo[*d*]thiazole-2-carbonyl)-4-(4-chlorophenyl)-4-oxobutyl)malonate (3f)

68.1 mg, 72% yield, slight yellow oil. Eluent: pentane/ethyl acetate = 5:1-3:1
^{1}H NMR (400 MHz, CDCl$_3$) δ 8.17 (d, *J* = 7.5 Hz, 1H), 7.99 (d, *J* = 7.3 Hz, 1H), 7.88 (d, *J* = 8.6 Hz, 2H), 7.60 – 7.51 (m, 2H), 7.42 (d, *J* = 8.5 Hz, 2H), 4.59 – 4.49 (m, 1H), 3.80 – 3.74 (m, 4H), 3.66 (s, 3H), 3.58 (dd, *J* = 8.5, 6.4 Hz, 1H), 3.38 (dd, *J* = 17.9, 4.5 Hz, 1H), 2.48 – 2.35 (m, 2H).
^{13}C NMR (101 MHz, CDCl$_3$) δ 196.6, 196.2, 169.2, 169.1, 166.0, 153.6, 140.0, 137.6, 134.3, 129.6, 129.0, 127.8, 126.9, 125.7, 122.5, 52.8, 52.8, 49.6, 42.0, 40.0, 30.7.
HRMS (ESI-TOF) m/z: $[M + H]^+$ Calcd for $C_{23}H_{21}ClNO_6S$ 474.0773; Found: 474.0779.

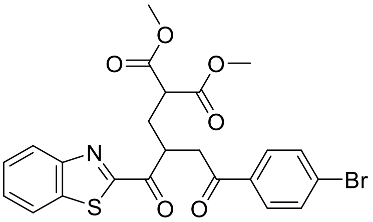

dimethyl 2-(2-(benzo[*d*]thiazole-2-carbonyl)-4-(4-bromophenyl)-4-oxobutyl)malonate (3g)

59.9 mg, 58% yield, slight yellow oil. Eluent: pentane/ethyl acetate = 5:1-3:1

^{1}H NMR (400 MHz, CDCl$_3$) δ 8.17 (d, *J* = 7.6 Hz, 1H), 7.99 (d, *J* = 7.4 Hz, 1H), 7.80 (d, *J* = 8.6 Hz, 2H), 7.60 – 7.51 (m, 4H), 4.57 – 4.50 (m, 1H), 3.77 – 3.73 (m, 4H), 3.66 (s, 3H), 3.58 (dd, *J* = 8.6, 6.3 Hz, 1H), 3.37 (dd, *J* = 17.9, 4.6 Hz, 1H), 2.49 – 2.35 (m, 2H).
^{13}C NMR (101 MHz, CDCl$_3$) δ 196.6, 196.4, 169.2, 169.1, 166.0, 153.6, 137.6, 134.7, 132.0, 129.7, 128.7, 127.8, 126.9, 125.7, 122.5, 52.8, 52.8, 49.6, 42.0, 40.0, 30.7.
HRMS (ESI-TOF) m/z: [M + H]$^+$ Calcd for $C_{23}H_{21}BrNO_6S$ 518.0267; Found: 518.0267.

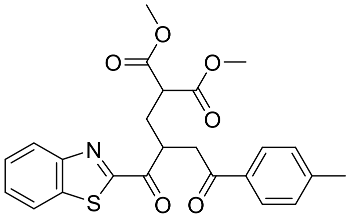

dimethyl 2-(2-(benzo[*d*]thiazole-2-carbonyl)-4-oxo-4-(*p*-tolyl)butyl)malonate (3h)

53.5 mg, 59% yield, slight yellow oil. Eluent: pentane/ethyl acetate = 5:1-3:1
^{1}H NMR (400 MHz, CDCl$_3$) δ 8.19 – 8.14 (m, 1H), 8.00 – 7.95 (m, 1H), 7.83 (d, *J* = 8.2 Hz, 2H), 7.58 – 7.49 (m, 2H), 7.23 (d, *J* = 8.0 Hz, 2H), 4.54 (tt, *J* = 9.7, 5.0 Hz, 1H), 3.82 – 3.75 (m, 1H), 3.74 (s, 3H), 3.66 (s, 3H), 3.59 (dd, *J* = 8.5, 6.4 Hz, 1H), 3.41 (dd, *J* = 17.9, 4.6 Hz, 1H), 2.47 – 2.35 (m, 5H).
^{13}C NMR (101 MHz, CDCl$_3$) δ 196.9, 196.8, 169.2, 169.2, 166.2, 153.6, 144.3, 137.6, 133.6, 129.3, 128.3, 127.7, 126.9, 125.7, 122.4, 52.8, 52.7, 49.7, 42.1, 40.0, 30.7, 21.7.
HRMS (ESI-TOF) m/z: [M + H]$^+$ Calcd for $C_{24}H_{24}NO_6S$ 454.1319; Found: 454.1321.

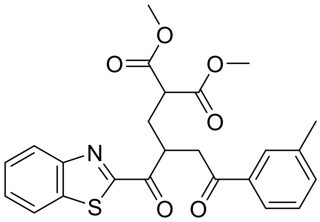

dimethyl 2-(2-(benzo[*d*]thiazole-2-carbonyl)-4-oxo-4-(*m*-tolyl)butyl)malonate (3i)

52.5 mg, 58% yield, slight yellow oil. Eluent: pentane/ethyl acetate = 5:1-3:1
^{1}H NMR (400 MHz, CDCl$_3$) δ 8.17 (d, *J* = 7.6 Hz, 1H), 7.98 (d, *J* = 7.5 Hz, 1H), 7.73 (d, *J* = 5.8 Hz, 2H), 7.59 – 7.50 (m, 2H), 7.38 – 7.31 (m, 2H), 4.59 – 4.50 (m, 1H), 3.79 (dd, *J* = 15.3, 6.9 Hz, 1H), 3.75 (s, 3H), 3.66 (s, 3H), 3.60 (dd, *J* = 8.6, 6.4 Hz, 1H), 3.42 (dd, *J* = 17.9, 4.6 Hz, 1H), 2.47 – 2.35 (m, 5H).
^{13}C NMR (101 MHz, CDCl$_3$) δ 197.5, 196.8, 169.2, 169.2, 166.2, 153.6, 138.4, 137.6, 136.0, 134.2, 128.7, 128.5, 127.7, 126.9, 125.7, 125.4, 122.5, 52.8, 52.7, 49.7, 42.2, 40.0, 30.7, 21.3.
HRMS (ESI-TOF) m/z: [M + H]$^+$ Calcd for $C_{24}H_{24}NO_6S$ 454.1319; Found: 454.1320.

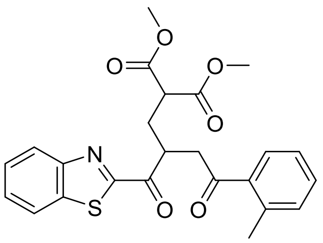

dimethyl 2-(2-(benzo[*d*]thiazole-2-carbonyl)-4-oxo-4-(*o*-tolyl)butyl)malonate (3j)

55.3 mg, 61% yield, slight yellow oil. Eluent: pentane/ethyl acetate = 5:1-3:1
^{1}H NMR (400 MHz, CDCl$_3$) δ 8.20 – 8.16 (m, 1H), 7.99 (d, *J* = 7.5 Hz, 1H), 7.74 (d, *J* = 6.7 Hz, 1H), 7.59 – 7.51 (m, 2H), 7.37 (t, *J* = 6.8 Hz, 1H), 7.27 (t, *J* = 7.3 Hz, 1H), 7.21 (d, *J* = 7.6 Hz, 1H), 4.56 – 4.47 (m, 1H), 3.78 – 3.74 (m, 4H), 3.67 (s, 3H), 3.59 (dd, *J* = 8.6, 6.3 Hz, 1H), 3.32 (dd, *J* = 17.9, 4.6 Hz, 1H), 2.47 – 2.35 (m, 5H).
^{13}C NMR (101 MHz, CDCl$_3$) δ 200.8, 196.7, 169.2, 169.2, 166.1, 153.6, 138.7, 137.5, 136.6, 132.1, 131.7, 128.9, 127.7, 126.9, 125.7, 125.7, 122.5, 52.8, 52.7, 49.6, 44.5, 40.4, 30.7, 21.5.
HRMS (ESI-TOF) m/z: $[M + H]^+$ Calcd for $C_{24}H_{24}NO_6S$ 454.1319; Found: 454.1319.

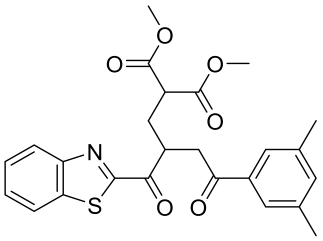

dimethyl 2-(2-(benzo[*d*]thiazole-2-carbonyl)-4-(3,5-dimethylphenyl)-4-oxobutyl)malonate (3k)

59.8 mg, 64% yield, slight yellow oil. Eluent: pentane/ethyl acetate = 5:1-3:1
^{1}H NMR (400 MHz, CDCl$_3$) δ 8.17 (d, *J* = 7.6 Hz, 1H), 7.98 (d, *J* = 7.5 Hz, 1H), 7.57 – 7.50 (m, 4H), 7.19 (s, 1H), 4.59 – 4.49 (m, 1H), 3.81 – 3.74 (m, 4H), 3.66 (s, 3H), 3.60 (dd, *J* = 8.6, 6.4 Hz, 1H), 3.42 (dd, *J* = 17.9, 4.7 Hz, 1H), 2.48 – 2.38 (m, 2H), 2.34 (s, 6H).
^{13}C NMR (101 MHz, CDCl$_3$) δ 197.7, 196.8, 169.2, 169.2, 166.2, 153.6, 138.3, 137.6, 136.1, 135.1, 127.7, 126.9, 126.0, 125.7, 122.4, 52.8, 52.7, 49.7, 42.3, 40.1, 30.7, 21.2.
HRMS (ESI-TOF) m/z: $[M + H]^+$ Calcd for $C_{25}H_{26}NO_6S$ 468.1475; Found: 468.1483.

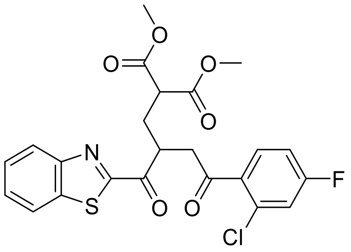

dimethyl 2-(2-(benzo[*d*]thiazole-2-carbonyl)-4-(2-chloro-4-fluorophenyl)-4-oxobutyl)malonate (3l)

62.8 mg, 64% yield, slight yellow oil. Eluent: pentane/ethyl acetate = 5:1-3:1

^{1}H NMR (400 MHz, CDCl$_3$) δ 8.17 (d, *J* = 7.1 Hz, 1H), 7.99 (d, *J* = 7.5 Hz, 1H), 7.63 (dd, *J* = 8.7, 6.1 Hz, 1H), 7.60 – 7.51 (m, 2H), 7.15 (dd, *J* = 8.5, 2.5 Hz, 1H), 7.05 – 7.00 (m, 1H), 4.57 – 4.47 (m, 1H), 3.81 – 3.74 (m, 4H), 3.67 (s, 3H), 3.58 (dd, *J* = 8.5, 6.3 Hz, 1H), 3.40 (dd, *J* = 18.2, 4.4 Hz, 1H), 2.49 – 2.34 (m, 2H).
^{13}C NMR (101 MHz, CDCl$_3$) δ 198.3, 196.3, 169.1, 169.1, 165.8, 163.9 (d, J_{C-F} = 256.2 Hz), 153.5, 137.5, 133.9 (d, J_{C-F} = 3.6 Hz), 133.4 (d, J_{C-F} = 10.7 Hz), 132.0 (d, J_{C-F} = 9.6 Hz), 127.8, 127.0, 125.7, 122.5, 118.3 (d, J_{C-F} = 24.8 Hz), 114.5 (d, J_{C-F} = 21.3 Hz), 52.8, 52.8, 49.6, 45.7, 40.6, 30.6.
^{19}F NMR (377 MHz, CDCl$_3$) δ -105.8.
HRMS (ESI-TOF) m/z: [M + H]$^+$ Calcd for $C_{23}H_{20}ClFNO_6S$ 492.0678; Found: 492.0682.

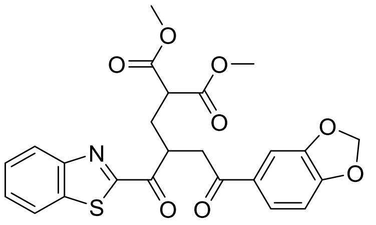

dimethyl 2-(4-(benzo[*d*][1,3]dioxol-5-yl)-2-(benzo[*d*]thiazole-2-carbonyl)-4-oxobutyl)malonate (3m)

61.0 mg, 63% yield, slight yellow oil. Eluent: pentane/ethyl acetate = 5:1-3:1
^{1}H NMR (400 MHz, CDCl$_3$) δ 8.17 (d, *J* = 7.6 Hz, 1H), 7.98 (d, *J* = 7.5 Hz, 1H), 7.59 – 7.50 (m, 3H), 7.38 (d, *J* = 1.6 Hz, 1H), 6.83 (d, *J* = 8.2 Hz, 1H), 6.02 (s, 2H), 4.56 – 4.47 (m, 1H), 3.76 – 3.69 (m, 4H), 3.66 (s, 3H), 3.58 (dd, *J* = 8.6, 6.4 Hz, 1H), 3.34 (dd, *J* = 17.7, 4.7 Hz, 1H), 2.47 – 2.33 (m, 2H).
^{13}C NMR (101 MHz, CDCl$_3$) δ 196.8, 195.4, 169.2, 169.2, 166.2, 153.6, 152.1, 148.2, 137.6, 130.9, 127.7, 126.9, 125.7, 124.6, 122.4, 107.9, 101.9, 52.8, 52.7, 49.6, 42.0, 40.1, 30.7.
HRMS (ESI-TOF) m/z: [M + H]$^+$ Calcd for $C_{24}H_{22}NO_8S$ 484.1061; Found: 484.1064.

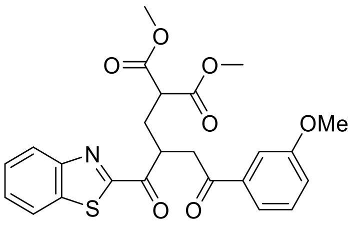

dimethyl 2-(2-(benzo[*d*]thiazole-2-carbonyl)-4-(3-methoxyphenyl)-4-oxobutyl)malonate (3n)

59.1 mg, 63% yield, slight yellow oil. Eluent: pentane/ethyl acetate = 5:1-3:1
^{1}H NMR (400 MHz, CDCl$_3$) δ 8.17 (d, *J* = 7.3 Hz, 1H), 7.99 (d, *J* = 7.5 Hz, 1H), 7.59 – 7.51 (m, 3H), 7.43 (dd, *J* = 2.3, 1.6 Hz, 1H), 7.36 (t, *J* = 7.9 Hz, 1H), 7.10 (dd, *J* = 8.2, 2.6 Hz, 1H), 4.59 – 4.50 (m, 1H), 3.83 – 3.78 (m, 4H), 3.75 (s, 3H), 3.66 (s, 3H), 3.59 (dd, *J* = 8.6, 6.4 Hz, 1H), 3.42 (dd, *J* = 17.9, 4.6 Hz, 1H), 2.49 – 2.35 (m, 2H).
^{13}C NMR (101 MHz, CDCl$_3$) δ 197.2, 196.7, 169.2, 169.2, 166.2, 159.8, 153.6, 137.6, 137.3, 129.6, 127.7, 126.9, 125.7, 122.4, 120.9, 120.2, 112.2, 55.4, 52.8, 52.7, 49.6, 42.3, 40.1, 30.7.

HRMS (ESI-TOF) m/z: $[M + H]^+$ Calcd for $C_{24}H_{24}NO_7S$ 470.1268; Found: 470.1260.

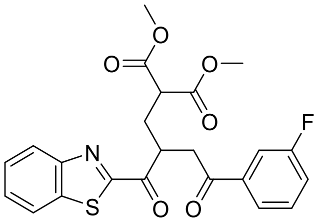

dimethyl 2-(2-(benzo[*d*]thiazole-2-carbonyl)-4-(3-fluorophenyl)-4-oxobutyl)malonate (3o)

56.7 mg, 62% yield, slight yellow oil. Eluent: pentane/ethyl acetate = 5:1-3:1

^{1}H NMR (400 MHz, CDCl$_3$) δ 8.17 (d, *J* = 7.5 Hz, 1H), 7.99 (d, *J* = 7.5 Hz, 1H), 7.73 (d, *J* = 7.8 Hz, 1H), 7.62 (d, *J* = 8.7 Hz, 1H), 7.60 – 7.51 (m, 2H), 7.47 – 7.41 (m, 1H), 7.27 (t, *J* = 9.5 Hz, 1H), 4.59 – 4.50 (m, 1H), 3.81 – 3.74 (m, 4H), 3.67 (s, 3H), 3.59 (dd, *J* = 8.6, 6.3 Hz, 1H), 3.38 (dd, *J* = 17.9, 4.6 Hz, 1H), 2.50 – 2.35 (m, 2H).

^{13}C NMR (101 MHz, CDCl$_3$) δ 196.5, 196.2, 169.2, 169.1, 166.0, 162.8 (d, $J_{C\text{-}F}$ = 248.1 Hz), 153.6, 138.1 (d, $J_{C\text{-}F}$ = 6.2 Hz), 137.6, 130.4 (d, $J_{C\text{-}F}$ = 7.6 Hz), 127.8, 127.0, 125.7, 124.0 (d, $J_{C\text{-}F}$ = 3.0 Hz), 122.5, 120.5 (d, $J_{C\text{-}F}$ = 21.4 Hz), 115.0 (d, $J_{C\text{-}F}$ = 22.4 Hz), 52.8, 52.8, 49.6, 42.1, 40.0, 30.6.

^{19}F NMR (377 MHz, CDCl$_3$) δ -111.6.

HRMS (ESI-TOF) m/z: $[M + H]^+$ Calcd for $C_{23}H_{21}FNO_6S$ 458.1068; Found: 458.1062.

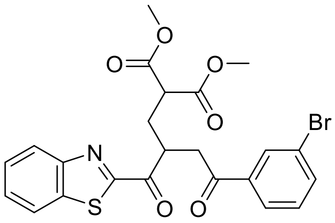

dimethyl 2-(2-(benzo[*d*]thiazole-2-carbonyl)-4-(3-bromophenyl)-4-oxobutyl)malonate (3p)

56.8 mg, 55% yield, slight yellow oil. Eluent: pentane/ethyl acetate = 5:1-3:1

^{1}H NMR (400 MHz, CDCl$_3$) δ 8.18 (d, *J* = 7.5 Hz, 1H), 8.07 (t, *J* = 1.7 Hz, 1H), 7.99 (d, *J* = 7.6 Hz, 1H), 7.86 (d, *J* = 7.8 Hz, 1H), 7.68 (d, *J* = 8.8 Hz, 1H), 7.59 – 7.51 (m, 2H), 7.33 (t, *J* = 7.9 Hz, 1H), 4.58 – 4.50 (m, 1H), 3.81 – 3.74 (m, 4H), 3.67 (s, 3H), 3.59 (dd, *J* = 8.6, 6.3 Hz, 1H), 3.37 (dd, *J* = 17.9, 4.7 Hz, 1H), 2.49 – 2.34 (m, 2H).

^{13}C NMR (101 MHz, CDCl$_3$) δ 196.5, 196.1, 169.2, 169.1, 165.9, 153.5, 137.7, 137.6, 136.3, 131.3, 130.3, 127.8, 127.0, 126.7, 125.7, 123.0, 122.5, 52.8, 52.8, 49.6, 42.0, 40.0, 30.6.

HRMS (ESI-TOF) m/z: $[M + H]^+$ Calcd for $C_{23}H_{21}BrNO_6S$ 518.0267; Found:518.0265.

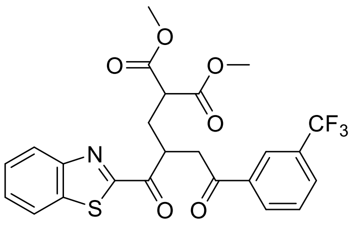

dimethyl 2-(2-(benzo[*d*]thiazole-2-carbonyl)-4-oxo-4-(3-(trifluoromethyl)phenyl)butyl)malonate (3q)

58.8 mg, 58% yield, slight yellow oil. Eluent: pentane/ethyl acetate = 5:1-3:1

^{1}H NMR (400 MHz, CDCl$_3$) δ 8.22 – 8.15 (m, 2H), 8.12 (d, *J* = 7.9 Hz, 1H), 7.99 (d, *J* = 7.5 Hz, 1H), 7.82 (d, *J* = 7.8 Hz, 1H), 7.63 – 7.51 (m, 3H), 4.63 – 4.52 (m, 1H), 3.83 (dd, *J* = 18.0, 9.6 Hz, 1H), 3.75 (s, 3H), 3.67 (s, 3H), 3.60 (dd, *J* = 8.6, 6.3 Hz, 1H), 3.43 (dd, *J* = 18.0, 4.6 Hz, 1H), 2.51 – 2.36 (m, 2H).

^{13}C NMR (101 MHz, CDCl$_3$) δ 196.5, 196.1, 169.1, 169.1, 165.9, 153.5, 137.6, 136.5, 131.3 (q, J_{C-F} = 33.0 Hz), 131.3, 129.9 (q, J_{C-F} = 3.6 Hz), 129.4, 127.8, 127.0, 125.7, 125.1 (q, J_{C-F} = 3.9 Hz), 123.6 (q, J_{C-F} = 272.7 Hz), 122.5, 52.8, 52.8, 49.6, 42.0, 40.0, 30.7.

^{19}F NMR (377 MHz, CDCl$_3$) δ -62.8.

HRMS (ESI-TOF) m/z: $[M + H]^+$ Calcd for $C_{24}H_{21}F_3NO_6S$ 508.1036; Found: 508.1040.

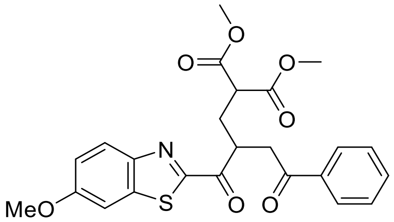

dimethyl 2-(2-(6-methoxybenzo[*d*]thiazole-2-carbonyl)-4-oxo-4-phenylbutyl)malonate (3r)

61.9 mg, 66% yield, slight yellow oil. Eluent: pentane/ethyl acetate = 5:1-3:1

^{1}H NMR (400 MHz, CDCl$_3$) δ 8.03 (d, *J* = 9.1 Hz, 1H), 7.94 (d, *J* = 7.2 Hz, 2H), 7.55 (t, *J* = 7.4 Hz, 1H), 7.44 (t, *J* = 7.7 Hz, 2H), 7.37 (d, *J* = 2.5 Hz, 1H), 7.16 (dd, *J* = 9.1, 2.5 Hz, 1H), 4.56 – 4.47 (m, 1H), 3.91 (s, 3H), 3.83 – 3.75 (m, 1H), 3.74 (s, 3H), 3.66 (s, 3H), 3.58 (dd, *J* = 8.6, 6.3 Hz, 1H), 3.39 (dd, *J* = 17.9, 4.7 Hz, 1H), 2.48 – 2.33 (m, 2H).

^{13}C NMR (101 MHz, CDCl$_3$) δ 197.4, 196.4, 169.2, 169.2, 163.6, 159.8, 148.2, 139.6, 136.1, 133.4, 128.6, 128.2, 126.4, 117.6, 103.6, 55.9, 52.7, 52.7, 49.7, 42.0, 39.9, 30.8.

HRMS (ESI-TOF) m/z: $[M + H]^+$ Calcd for $C_{24}H_{24}NO_7S$ 470.1268; Found: 470.1264.

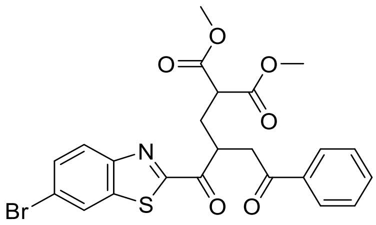

dimethyl 2-(2-(6-bromobenzo[*d*]thiazole-2-carbonyl)-4-oxo-4-phenylbutyl)malonate (3s)

57.9 mg, 56% yield, slight yellow oil. Eluent: pentane/ethyl acetate = 5:1-3:1

^{1}H NMR (400 MHz, CDCl$_3$) δ 8.32 (d, *J* = 1.8 Hz, 1H), 7.92 (d, *J* = 7.3 Hz, 2H), 7.85 (d, *J* = 8.6 Hz, 1H), 7.63 (dd, *J* = 8.6, 1.8 Hz, 1H), 7.56 (t, *J* = 7.4 Hz, 1H), 7.45 (t, *J* = 7.7 Hz, 2H), 4.53 – 4.43 (m, 1H), 3.80 (dd, *J* = 16.8, 8.5 Hz, 1H), 3.75 (s, 3H), 3.67 (s, 3H), 3.59 (dd, *J* = 8.4, 6.4 Hz, 1H), 3.45 (dd, *J* = 18.0, 4.4 Hz, 1H), 2.50 – 2.34 (m, 2H).

^{13}C NMR (101 MHz, CDCl$_3$) δ 197.3, 196.4, 169.1, 169.1, 167.7, 154.6, 136.3, 135.9, 133.6, 130.8, 128.7, 128.3, 128.2, 123.5, 120.5, 52.8, 52.8, 49.6, 42.1, 40.1, 30.6.

HRMS (ESI-TOF) m/z: $[M + H]^+$ Calcd for $C_{23}H_{21}BrNO_6S$ 518.0267; Found: 518.0270.

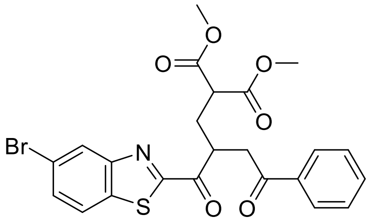

dimethyl 2-(2-(5-bromobenzo[*d*]thiazole-2-carbonyl)-4-oxo-4-phenylbutyl)malonate (3t)

70.3 mg, 68% yield, slight yellow oil. Eluent: pentane/ethyl acetate = 5:1-3:1

^{1}H NMR (400 MHz, CDCl$_3$) δ 8.13 (d, *J* = 1.9 Hz, 1H), 8.01 (d, *J* = 8.8 Hz, 1H), 7.92 (d, *J* = 7.2 Hz, 2H), 7.66 (dd, *J* = 8.8, 1.9 Hz, 1H), 7.56 (t, *J* = 7.4 Hz, 1H), 7.44 (t, *J* = 7.7 Hz, 2H), 4.55 – 4.45 (m, 1H), 3.83 – 3.76 (m, 1H), 3.74 (s, 3H), 3.67 (s, 3H), 3.57 (dd, *J* = 8.4, 6.5 Hz, 1H), 3.44 (dd, *J* = 18.0, 4.5 Hz, 1H), 2.49 – 2.34 (m, 2H).

^{13}C NMR (101 MHz, CDCl$_3$) δ 197.3, 196.5, 169.1, 169.1, 166.6, 152.3, 139.1, 135.9, 133.6, 130.6, 128.7, 128.2, 126.7, 125.0, 121.9, 52.8, 52.8, 49.6, 42.1, 40.1, 30.7.

HRMS (ESI-TOF) m/z: $[M + H]^+$ Calcd for $C_{23}H_{21}BrNO_6S$ 518.0267; Found: 518.0270.

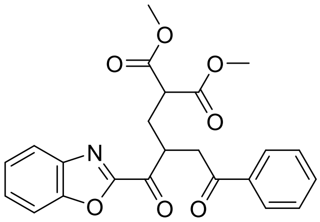

dimethyl 2-(2-(benzo[*d*]oxazole-2-carbonyl)-4-oxo-4-phenylbutyl)malonate (3u)

55.8 mg, 66% yield, slight yellow oil. Eluent: pentane/ethyl acetate = 5:1-3:1

^{1}H NMR (400 MHz, CDCl$_3$) δ 7.94 – 7.87 (m, 3H), 7.67 (d, *J* = 8.2 Hz, 1H), 7.59 – 7.51 (m, 2H), 7.48 – 7.42 (m, 3H), 4.46 – 4.36 (m, 1H), 3.80 (d, *J* = 8.3 Hz, 1H), 3.75 (s, 3H), 3.67 (s, 3H), 3.59 (dd, *J* = 8.3, 6.5 Hz, 1H), 3.47 (dd, *J* = 18.0, 4.4 Hz, 1H), 2.50 – 2.35 (m, 2H).
^{13}C NMR (101 MHz, CDCl$_3$) δ 197.3, 191.7, 169.1, 169.1, 157.4, 151.0, 140.7, 135.8, 133.6, 128.7, 128.5, 128.2, 125.7, 122.4, 112.0, 52.8, 49.5, 42.3, 40.9, 30.6.
HRMS (ESI-TOF) m/z: $[M + H]^+$ Calcd for $C_{23}H_{22}NO_7$ 424.1391; Found: 424.1399.

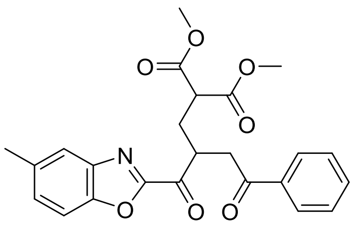

dimethyl 2-(2-(5-methylbenzo[d]oxazole-2-carbonyl)-4-oxo-4-phenylbutyl)malonate (3v)

53.3 mg, 61% yield, slight yellow solid. Eluent: pentane/ethyl acetate = 5:1-3:1
^{1}H NMR (400 MHz, CDCl$_3$) δ 7.92 (d, *J* = 7.2 Hz, 2H), 7.65 (s, 1H), 7.55 (dd, *J* = 15.7, 8.0 Hz, 2H), 7.44 (t, *J* = 7.7 Hz, 2H), 7.34 (d, *J* = 9.9 Hz, 1H), 4.44 – 4.34 (m, 1H), 3.84 – 3.73 (m, 4H), 3.67 (s, 3H), 3.58 (dd, *J* = 8.3, 6.5 Hz, 1H), 3.45 (dd, *J* = 18.0, 4.5 Hz, 1H), 2.50 (d, *J* = 4.7 Hz, 3H), 2.47 – 2.34 (m, 2H).
^{13}C NMR (101 MHz, CDCl$_3$) δ 197.2, 191.6, 169.1, 169.1, 157.5, 149.3, 140.9, 135.9, 135.7, 133.6, 130.0, 128.7, 128.2, 122.0, 111.4, 52.8, 52.8, 49.5, 42.2, 40.8, 30.6, 21.5.
HRMS (ESI-TOF) m/z: $[M + H]^+$ Calcd for $C_{24}H_{24}NO_7$ 438.1547; Found: 438.1553.

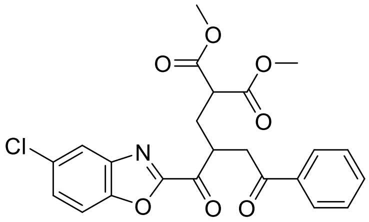

dimethyl 2-(2-(5-chlorobenzo[*d*]oxazole-2-carbonyl)-4-oxo-4-phenylbutyl)malonate (3w)

51.2 mg, 56% yield, slight yellow oil. Eluent: pentane/ethyl acetate = 5:1-3:1
^{1}H NMR (400 MHz, CDCl$_3$) δ 7.91 (d, *J* = 7.2 Hz, 2H), 7.87 (d, *J* = 2.0 Hz, 1H), 7.61 – 7.55 (m, 2H), 7.50 (dd, *J* = 8.8, 2.0 Hz, 1H), 7.45 (t, *J* = 7.7 Hz, 2H), 4.40 – 4.30 (m, 1H), 3.79 (dd, *J* = 16.6, 8.3 Hz, 1H), 3.75 (s, 3H), 3.68 (s, 3H), 3.57 (dd, *J* = 8.2, 6.6 Hz, 1H), 3.48 (dd, *J* = 18.1, 4.3 Hz, 1H), 2.48 – 2.34 (m, 2H).
^{13}C NMR (101 MHz, CDCl$_3$) δ 197.3, 191.4, 169.0, 169.0, 158.4, 149.5, 141.7, 135.7, 133.7, 131.3, 128.9, 128.7, 128.2, 122.1, 112.9, 52.8, 49.5, 42.4, 40.9, 30.6.
HRMS (ESI-TOF) m/z: $[M + H]^+$ Calcd for $C_{23}H_{21}ClNO_7$ 458.1001; Found: 458.0999.

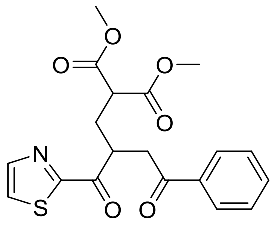

dimethyl 2-(4-oxo-4-phenyl-2-(thiazole-2-carbonyl)butyl)malonate (3x)

53.7 mg, 69% yield, slight yellow oil. Eluent: pentane/ethyl acetate = 5:1-3:1
^{1}H NMR (400 MHz, CDCl$_3$) δ 8.03 (d, *J* = 3.0 Hz, 1H), 7.93 (d, *J* = 7.2 Hz, 2H), 7.69 (d, *J* = 3.0 Hz, 1H), 7.56 (t, *J* = 7.4 Hz, 1H), 7.44 (t, *J* = 7.7 Hz, 2H), 4.47 – 4.38 (m, 1H), 3.79 – 3.73 (m, 4H), 3.68 (s, 3H), 3.54 (dd, *J* = 8.4, 6.5 Hz, 1H), 3.35 (dd, *J* = 17.9, 4.6 Hz, 1H), 2.47 – 2.39 (m, 1H), 2.36 – 2.28 (m, 1H).
^{13}C NMR (101 MHz, CDCl$_3$) δ 197.3, 195.1, 169.2, 169.2, 166.7, 144.9, 136.1, 133.4, 128.6, 128.2, 126.5, 52.7, 52.7, 49.6, 41.6, 40.1, 30.6.
HRMS (ESI-TOF) m/z: [M + H]$^+$ Calcd for $C_{19}H_{20}NO_6S$ 390.1006; Found: 390.1004.

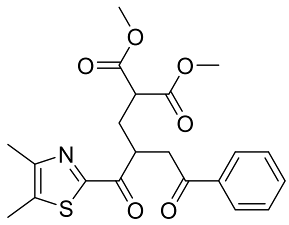

dimethyl 2-(2-(4,5-dimethylthiazole-2-carbonyl)-4-oxo-4-phenylbutyl)malonate (3y)

56.7 mg, 68% yield, slight yellow oil. Eluent: pentane/ethyl acetate = 5:1-3:1
^{1}H NMR (400 MHz, CDCl$_3$) δ 7.93 (dd, *J* = 8.3, 1.2 Hz, 2H), 7.54 (t, *J* = 7.4 Hz, 1H), 7.43 (t, *J* = 7.6 Hz, 2H), 4.41 – 4.32 (m, 1H), 3.76 – 3.67 (m, 7H), 3.53 (dd, *J* = 8.5, 6.4 Hz, 1H), 3.27 (dd, *J* = 17.8, 4.7 Hz, 1H), 2.46 – 2.42 (m, 3H), 2.39 (s, 3H), 2.35 – 2.27 (m, 2H).
^{13}C NMR (101 MHz, CDCl$_3$) δ 197.5, 194.9, 169.3, 169.2, 161.3, 151.6, 136.4, 136.2, 133.3, 128.6, 128.2, 52.7, 49.7, 41.7, 39.4, 30.8, 15.0, 12.2.
HRMS (ESI-TOF) m/z: [M + H]$^+$ Calcd for $C_{21}H_{24}NO_6S$ 418.1319; Found: 418.1324.

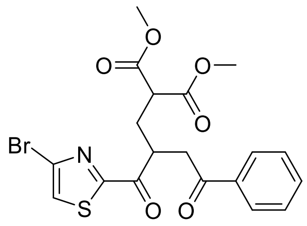

dimethyl 2-(2-(4-bromothiazole-2-carbonyl)-4-oxo-4-phenylbutyl)malonate (3z)

23.3 mg, 50% yield (0.1 mmol), slight yellow oil. Eluent: pentane/ethyl acetate = 5:1-3:1

^{1}H NMR (400 MHz, CDCl$_3$) δ 7.92 (d, *J* = 7.3 Hz, 2H), 7.62 – 7.53 (m, 2H), 7.45 (t, *J* = 7.7 Hz, 2H), 4.37 – 4.26 (m, 1H), 3.77 (s, 3H), 3.76 – 3.69 (m, 4H), 3.50 (dd, *J* = 8.4, 6.7 Hz, 1H), 3.39 (dd, *J* = 17.9, 4.3 Hz, 1H), 2.42 – 2.28 (m, 2H).
^{13}C NMR (101 MHz, CDCl$_3$) δ 197.3, 194.1, 169.1, 169.0, 166.6, 135.9, 133.5, 128.7, 128.2, 127.1, 125.2, 52.9, 52.8, 49.6, 42.0, 39.7, 30.7.
HRMS (ESI-TOF) m/z: $[M + H]^+$ Calcd for $C_{19}H_{19}BrNO_6S$ 468.0111; Found: 468.0115.

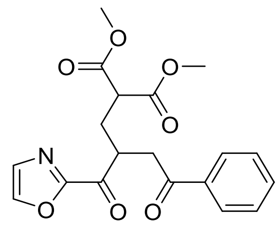

dimethyl 2-(2-(oxazole-2-carbonyl)-4-oxo-4-phenylbutyl)malonate (3ab)

41.0 mg, 55% yield, slight yellow oil. Eluent: pentane/ethyl acetate = 5:1-3:1
^{1}H NMR (400 MHz, CDCl$_3$) δ 7.94 – 7.89 (m, 2H), 7.84 (s, 1H), 7.56 (t, *J* = 7.4 Hz, 1H), 7.44 (t, *J* = 7.7 Hz, 2H), 7.36 (s, 1H), 4.29 – 4.19 (m, 1H), 3.76 – 3.68 (m, 7H), 3.54 (dd, *J* = 8.2, 6.7 Hz, 1H), 3.36 (dd, *J* = 18.0, 4.5 Hz, 1H), 2.46 – 2.36 (m, 1H), 2.35 – 2.26 (m, 1H).
^{13}C NMR (101 MHz, CDCl$_3$) δ 197.2, 189.6, 169.1, 169.1, 158.0, 141.8, 135.9, 133.5, 129.3, 128.7, 128.2, 52.8, 52.8, 49.5, 41.6, 40.7, 30.5.
HRMS (ESI-TOF) m/z: $[M + H]^+$ Calcd for $C_{19}H_{20}NO_7$ 374.1234; Found: 374.1235.

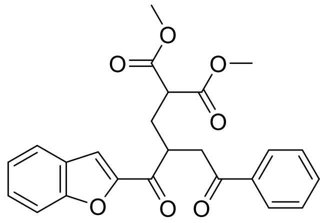

dimethyl 2-(2-(benzofuran-2-carbonyl)-4-oxo-4-phenylbutyl)malonate (3ac)

37.1 mg, 44% yield, slight yellow oil. Eluent: pentane/ethyl acetate = 5:1-3:1
^{1}H NMR (400 MHz, CDCl$_3$) δ 7.94 (d, *J* = 7.2 Hz, 2H), 7.73 (dd, *J* = 9.4, 4.2 Hz, 2H), 7.57 (t, *J* = 8.5 Hz, 2H), 7.51 – 7.42 (m, 3H), 7.32 (t, *J* = 7.8 Hz, 1H), 4.15 – 4.07 (m, 1H), 3.80 – 3.69 (m, 7H), 3.50 (t, *J* = 7.6 Hz, 1H), 3.25 (dd, *J* = 18.0, 4.6 Hz, 1H), 2.53 – 2.43 (m, 1H), 2.28 – 2.20 (m, 1H).
^{13}C NMR (101 MHz, CDCl$_3$) δ 197.3, 192.6, 169.2, 169.1, 155.9, 152.1, 136.2, 133.5, 128.7, 128.5, 128.1, 127.2, 124.0, 123.5, 114.2, 112.5, 52.8, 49.4, 41.0, 40.0, 31.1.
HRMS (ESI-TOF) m/z: $[M + H]^+$ Calcd for $C_{24}H_{23}O_7$ 423.1438; Found: 423.1438.

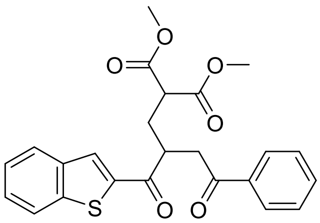

dimethyl 2-(2-(benzo[*b*]thiophene-2-carbonyl)-4-oxo-4-phenylbutyl)malonate (3ad)

38.5 mg, 44% yield, slight yellow oil. Eluent: pentane/ethyl acetate = 5:1-3:1
^{1}H NMR (400 MHz, $CDCl_3$) δ 8.20 (s, 1H), 7.94 (d, *J* = 7.3 Hz, 2H), 7.87 (d, *J* = 7.9 Hz, 1H), 7.57 (t, *J* = 7.4 Hz, 1H), 7.52 – 7.38 (m, 5H), 4.25 – 4.16 (m, 1H), 3.76 (d, *J* = 11.5 Hz, 4H), 3.71 (s, 3H), 3.49 (t, *J* = 7.6 Hz, 1H), 3.24 (dd, *J* = 18.0, 4.7 Hz, 1H), 2.57 – 2.48 (m, 1H), 2.25 – 2.17 (m, 1H).
^{13}C NMR (101 MHz, $CDCl_3$) δ 197.4, 196.4, 169.2, 169.1, 143.1, 143.0, 139.3, 136.2, 133.5, 130.2, 128.7, 128.1, 127.6, 126.3, 125.0, 123.0, 52.8, 52.8, 49.4, 41.1, 40.4, 31.5.
HRMS (ESI-TOF) m/z: $[M + H]^+$ Calcd for $C_{24}H_{23}O_6S$ 439.1210; Found: 439.1210.

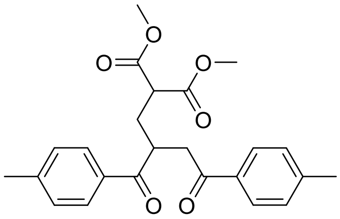

dimethyl 2-(2-(4-methylbenzoyl)-4-oxo-4-(*p*-tolyl)butyl)malonate (3ae)

29.5 mg, 36% yield, slight yellow oil. Eluent: pentane/ethyl acetate = 5:1-3:1
^{1}H NMR (400 MHz, $CDCl_3$) δ 7.96 (d, *J* = 8.2 Hz, 2H), 7.83 (d, *J* = 8.2 Hz, 2H), 7.28 (d, *J* = 8.1 Hz, 2H), 7.23 (d, *J* = 8.1 Hz, 2H), 4.25 – 4.16 (m, 1H), 3.72 (s, 3H), 3.69 (s, 3H), 3.61 (dd, *J* = 17.9, 8.2 Hz, 1H), 3.43 (t, *J* = 7.6 Hz, 1H), 3.14 (dd, *J* = 17.9, 5.0 Hz, 1H), 2.48 – 2.36 (m, 7H), 2.15 – 2.06 (m, 1H).
^{13}C NMR (101 MHz, $CDCl_3$) δ 201.8, 197.2, 169.3, 169.2, 144.2, 144.1, 133.9, 133.8, 129.4, 129.3, 128.8, 128.2, 52.7, 52.7, 49.4, 40.9, 38.7, 31.0, 21.7, 21.7.
HRMS (ESI-TOF) m/z: $[M + H]^+$ Calcd for $C_{24}H_{27}O_6$ 411.1802; Found: 411.1804.

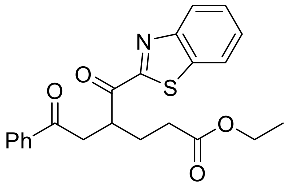

ethyl 4-(benzo[*d*]thiazole-2-carbonyl)-6-oxo-6-phenylhexanoate (4a)

46.6 mg, 59% yield, slight yellow solid. Eluent: pentane/ethyl acetate = 5:1-3:1
^{1}H NMR (400 MHz, $CDCl_3$) δ 8.21 (d, *J* = 7.6 Hz, 1H), 8.01 – 7.93 (m, 3H), 7.59 – 7.50 (m, 3H), 7.45 (t, *J* = 7.7 Hz, 2H), 4.62 – 4.52 (m, 1H), 4.09 (q, *J* = 7.1 Hz, 2H), 3.80 (dd, *J* = 18.0, 10.0 Hz,

1H), 3.38 (dd, *J* = 18.0, 4.3 Hz, 1H), 2.52 – 2.39 (m, 2H), 2.26 – 2.17 (m, 1H), 2.16 – 2.07 (m, 1H), 1.20 (t, *J* = 7.1 Hz, 3H).
^{13}C NMR (101 MHz, CDCl$_3$) δ 197.7, 197.3, 172.7, 166.3, 153.7, 137.6, 136.1, 133.4, 128.6, 128.2, 127.6, 126.9, 125.7, 122.4, 60.6, 41.6, 41.1, 31.9, 27.1, 14.2.
HRMS (ESI-TOF) m/z: $[M + H]^+$ Calcd for $C_{22}H_{22}NO_4S$ 396.1264; Found: 396.1267.

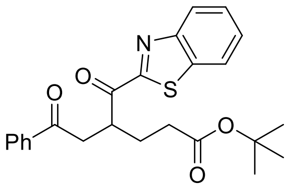

tert-butyl 4-(benzo[*d*]thiazole-2-carbonyl)-6-oxo-6-phenylhexanoate (4b)

63.5 mg, 75% yield, slight yellow oil. Eluent: pentane/ethyl acetate = 5:1-3:1
^{1}H NMR (400 MHz, CDCl$_3$) δ 8.21 (d, *J* = 7.5 Hz, 1H), 8.00 – 7.94 (m, 3H), 7.59 – 7.51 (m, 3H), 7.44 (t, *J* = 7.7 Hz, 2H), 4.61 – 4.49 (m, 1H), 3.79 (dd, *J* = 18.0, 10.1 Hz, 1H), 3.38 (dd, *J* = 18.0, 4.2 Hz, 1H), 2.48 – 2.29 (m, 2H), 2.22 – 2.01 (m, 2H), 1.41 (s, 9H).
^{13}C NMR (101 MHz, CDCl$_3$) δ 197.8, 197.4, 172.0, 166.3, 153.7, 137.6, 136.2, 133.4, 128.6, 128.2, 127.6, 126.8, 125.7, 122.4, 80.6, 41.5, 41.1, 33.0, 28.1, 27.2.
HRMS (ESI-TOF) m/z: $[M + H]^+$ Calcd for $C_{24}H_{26}NO_4S$ 424.1577; Found: 424.1582.

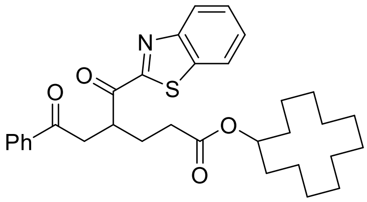

cyclododecyl 4-(benzo[*d*]thiazole-2-carbonyl)-6-oxo-6-phenylhexanoate (4c)

75.7 mg, 71% yield, slight yellow oil. Eluent: pentane/ethyl acetate = 5:1-3:1
^{1}H NMR (400 MHz, CDCl$_3$) δ 8.21 (d, *J* = 7.7 Hz, 1H), 8.01 – 7.93 (m, 3H), 7.60 – 7.50 (m, 3H), 7.44 (t, *J* = 7.7 Hz, 2H), 5.02 – 4.95 (m, 1H), 4.61 – 4.52 (m, 1H), 3.80 (dd, *J* = 18.0, 10.0 Hz, 1H), 3.38 (dd, *J* = 18.0, 4.2 Hz, 1H), 2.52 – 2.35 (m, 2H), 2.27 – 2.05 (m, 2H), 1.64 (d, *J* = 13.8 Hz, 2H), 1.43 – 1.29 (m, 20H).
^{13}C NMR (101 MHz, CDCl$_3$) δ 197.7, 197.3, 172.4, 166.3, 153.7, 137.6, 136.2, 133.4, 128.6, 128.2, 127.6, 126.8, 125.8, 122.4, 72.6, 41.6, 41.2, 32.2, 29.0, 29.0, 27.1, 24.1, 24.1, 23.9, 23.3, 23.2, 23.1, 20.9, 20.8.
HRMS (ESI-TOF) m/z: $[M + H]^+$ Calcd for $C_{32}H_{40}NO_4S$ 534.2673; Found: 534.2671.

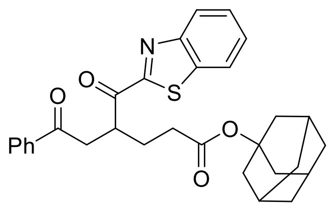

adamantan-1-yl 4-(benzo[*d*]thiazole-2-carbonyl)-6-oxo-6-phenylhexanoate (4d)

62.1 mg, 62% yield, slight yellow oil. Eluent: pentane/ethyl acetate = 5:1-3:1

^{1}H NMR (400 MHz, CDCl$_3$) δ 8.21 (d, *J* = 7.7 Hz, 1H), 8.00 – 7.94 (m, 3H), 7.59 – 7.50 (m, 3H), 7.44 (t, *J* = 7.7 Hz, 2H), 4.59 – 4.51 (m, 1H), 3.79 (dd, *J* = 18.0, 10.1 Hz, 1H), 3.38 (dd, *J* = 18.0, 4.2 Hz, 1H), 2.45 – 2.31 (m, 2H), 2.12 (s, 4H), 2.07 – 2.02 (m, 7H), 1.63 (s, 6H).

^{13}C NMR (101 MHz, CDCl$_3$) δ 197.8, 197.4, 171.8, 166.3, 153.7, 137.6, 136.2, 133.4, 128.6, 128.2, 127.6, 126.8, 125.7, 122.4, 80.7, 41.5, 41.3, 41.2, 36.2, 33.2, 30.8, 27.2.

HRMS (ESI-TOF) m/z: $[M + H]^+$ Calcd for $C_{30}H_{32}NO_4S$ 502.2047; Found: 502.2050.

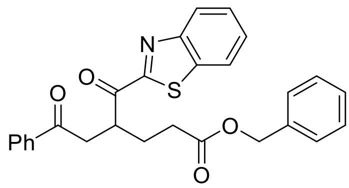

benzyl 4-(benzo[*d*]thiazole-2-carbonyl)-6-oxo-6-phenylhexanoate (4e)

58.5 mg, 64% yield, slight yellow oil. Eluent: pentane/ethyl acetate = 5:1-3:1

^{1}H NMR (400 MHz, CDCl$_3$) δ 8.19 (d, *J* = 7.6 Hz, 1H), 7.98 (d, *J* = 7.4 Hz, 1H), 7.94 (d, *J* = 7.2 Hz, 2H), 7.60 – 7.50 (m, 3H), 7.44 (t, *J* = 7.7 Hz, 2H), 7.33 – 7.28 (m, 5H), 5.09 (s, 2H), 4.64 – 4.52 (m, 1H), 3.80 (dd, *J* = 18.0, 10.0 Hz, 1H), 3.37 (dd, *J* = 18.0, 4.3 Hz, 1H), 2.61 – 2.45 (m, 2H), 2.30 – 2.10 (m, 2H).

^{13}C NMR (101 MHz, CDCl$_3$) δ 197.7, 197.2, 172.6, 166.3, 153.7, 137.6, 136.1, 135.8, 133.4, 128.6, 128.6, 128.3, 128.2, 127.6, 126.9, 125.8, 122.4, 66.5, 41.6, 41.1, 31.9, 27.0.

HRMS (ESI-TOF) m/z: $[M + H]^+$ Calcd for $C_{27}H_{24}NO_4S$ 458.1421; Found: 458.1423.

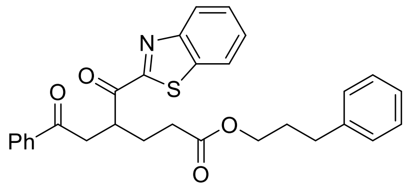

3-phenylpropyl 4-(benzo[*d*]thiazole-2-carbonyl)-6-oxo-6-phenylhexanoate (4f)

68.9 mg, 71% yield, slight yellow oil. Eluent: pentane/ethyl acetate = 5:1-3:1

^{1}H NMR (400 MHz, CDCl$_3$) δ 8.17 (d, *J* = 7.5 Hz, 1H), 8.00 – 7.91 (m, 3H), 7.57 – 7.48 (m, 3H), 7.43 (t, *J* = 7.7 Hz, 2H), 7.24 (t, *J* = 7.3 Hz, 2H), 7.18 – 7.10 (m, 3H), 4.61 – 4.51 (m, 1H), 4.05 (t, *J* = 6.6 Hz, 2H), 3.79 (dd, *J* = 18.0, 9.9 Hz, 1H), 3.37 (dd, *J* = 18.0, 4.3 Hz, 1H), 2.64 – 2.59 (m, 2H), 2.51 – 2.38 (m, 2H), 2.26 – 2.16 (m, 1H), 2.15 – 2.06 (m, 1H), 1.93 – 1.85 (m, 2H).

^{13}C NMR (101 MHz, CDCl$_3$) δ 197.7, 197.3, 172.7, 166.3, 153.7, 141.2, 137.6, 136.2, 133.4, 128.7, 128.4, 128.4, 128.2, 127.6, 126.9, 126.0, 125.7, 122.4, 64.1, 41.6, 41.2, 32.2, 31.8, 30.1, 27.1.
HRMS (ESI-TOF) m/z: $[M + H]^+$ Calcd for $C_{29}H_{28}NO_4S$ 486.1734; Found: 486.1740.

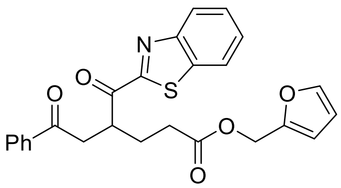

furan-2-ylmethyl 4-(benzo[*d*]thiazole-2-carbonyl)-6-oxo-6-phenylhexanoate (4g)

32.2 mg, 36% yield, slight yellow oil. Eluent: pentane/ethyl acetate = 5:1-3:1
^{1}H NMR (400 MHz, CDCl$_3$) δ 8.20 (d, *J* = 7.6 Hz, 1H), 7.98 (d, *J* = 7.0 Hz, 1H), 7.94 (d, *J* = 7.2 Hz, 2H), 7.60 – 7.50 (m, 3H), 7.45 (t, *J* = 7.7 Hz, 2H), 7.36 (dd, *J* = 1.7, 0.7 Hz, 1H), 6.37 – 6.31 (m, 2H), 5.03 (s, 2H), 4.60 – 4.51 (m, 1H), 3.79 (dd, *J* = 18.0, 10.0 Hz, 1H), 3.37 (dd, *J* = 18.0, 4.3 Hz, 1H), 2.57 – 2.42 (m, 2H), 2.27 – 2.08 (m, 2H).
^{13}C NMR (101 MHz, CDCl$_3$) δ 197.6, 197.2, 172.3, 166.2, 153.7, 149.3, 143.3, 137.6, 136.1, 133.4, 128.6, 128.2, 127.6, 126.8, 125.8, 122.4, 110.7, 110.6, 58.2, 41.6, 41.1, 31.7, 27.0.
HRMS (ESI-TOF) m/z: $[M + H]^+$ Calcd for $C_{25}H_{22}NO_5S$ 448.1213; Found: 448.1208.

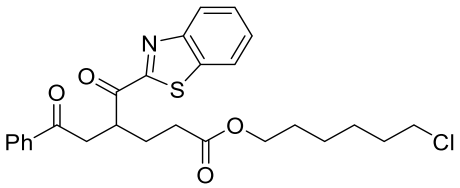

6-chlorohexyl 4-(benzo[*d*]thiazole-2-carbonyl)-6-oxo-6-phenylhexanoate (4h)

61.1 mg, 63% yield, slight yellow oil. Eluent: pentane/ethyl acetate = 5:1-3:1
^{1}H NMR (400 MHz, CDCl$_3$) δ 8.21 (d, *J* = 8.6 Hz, 1H), 8.01 – 7.93 (m, 3H), 7.60 – 7.51 (m, 3H), 7.45 (t, *J* = 7.7 Hz, 2H), 4.61 – 4.52 (m, 1H), 4.03 (t, *J* = 6.7 Hz, 2H), 3.80 (dd, *J* = 18.0, 9.9 Hz, 1H), 3.49 (t, *J* = 6.7 Hz, 2H), 3.38 (dd, *J* = 18.0, 4.3 Hz, 1H), 2.52 – 2.39 (m, 2H), 2.26 – 2.08 (m, 2H), 1.77 – 1.70 (m, 2H), 1.60 – 1.53 (m, 2H), 1.44 – 1.38 (m, 2H), 1.35 – 1.29 (m, 2H).
^{13}C NMR (101 MHz, CDCl$_3$) δ 197.6, 197.2, 172.8, 166.3, 153.7, 137.6, 136.2, 133.4, 128.6, 128.2, 127.6, 126.9, 125.7, 122.4, 64.5, 44.9, 41.6, 41.2, 32.4, 31.9, 28.4, 27.1, 26.5, 25.2.
HRMS (ESI-TOF) m/z: $[M + H]^+$ Calcd for $C_{26}H_{29}ClNO_4S$ 486.1500; Found: 486.1506.

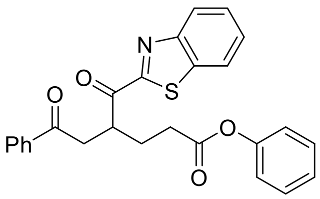

phenyl 4-(benzo[*d*]thiazole-2-carbonyl)-6-oxo-6-phenylhexanoate (4i)

51.4 mg, 58% yield, white solid. Eluent: pentane/ethyl acetate = 5:1-3:1

^{1}H NMR (400 MHz, $CDCl_3$) δ 8.20 (d, *J* = 7.7 Hz, 1H), 8.02 – 7.95 (m, 3H), 7.60 – 7.51 (m, 3H), 7.46 (t, *J* = 7.7 Hz, 2H), 7.36 (t, *J* = 7.9 Hz, 2H), 7.22 (t, *J* = 7.4 Hz, 1H), 7.07 (d, *J* = 7.6 Hz, 2H), 4.72 – 4.63 (m, 1H), 3.86 (dd, *J* = 18.0, 9.8 Hz, 1H), 3.45 (dd, *J* = 18.0, 4.4 Hz, 1H), 2.82 – 2.67 (m, 2H), 2.38 – 2.21 (m, 2H).

^{13}C NMR (101 MHz, $CDCl_3$) δ 197.6, 197.2, 171.3, 166.3, 153.7, 150.6, 137.6, 136.1, 133.5, 129.4, 128.7, 128.2, 127.7, 126.9, 125.9, 125.8, 122.4, 121.5, 41.7, 41.1, 32.0, 27.0.

HRMS (ESI-TOF) m/z: $[M + H]^+$ Calcd for $C_{26}H_{22}NO_4S$ 444.1264; Found: 444.1270.

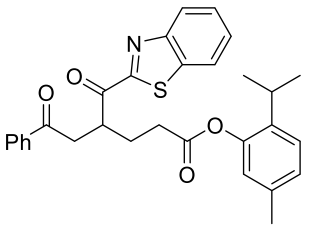

2-isopropyl-5-methylphenyl 4-(benzo[*d*]thiazole-2-carbonyl)-6-oxo-6-phenylhexanoate (4j)

66.9 mg, 67% yield, slight yellow oil. Eluent: pentane/ethyl acetate = 5:1-3:1

^{1}H NMR (400 MHz, $CDCl_3$) δ 8.23 (d, *J* = 7.2 Hz, 1H), 8.02 – 7.96 (m, 3H), 7.60 – 7.52 (m, 3H), 7.46 (t, *J* = 7.7 Hz, 2H), 7.18 (d, *J* = 7.9 Hz, 1H), 7.01 (d, *J* = 7.9 Hz, 1H), 6.80 (s, 1H), 4.72 – 4.62 (m, 1H), 3.87 (dd, *J* = 18.0, 9.8 Hz, 1H), 3.46 (dd, *J* = 18.0, 4.4 Hz, 1H), 2.97 – 2.88 (m, 1H), 2.84 – 2.69 (m, 2H), 2.39 – 2.27 (m, 5H), 1.15 (dd, *J* = 6.9, 1.3 Hz, 6H).

^{13}C NMR (101 MHz, $CDCl_3$) δ 197.6, 197.2, 171.6, 166.3, 153.7, 147.8, 137.6, 137.0, 136.6, 136.1, 133.5, 128.7, 128.2, 127.7, 127.2, 126.9, 126.4, 125.8, 122.7, 122.4, 41.7, 41.3, 31.9, 27.1, 27.0, 23.1, 20.8.

HRMS (ESI-TOF) m/z: $[M + H]^+$ Calcd for $C_{30}H_{30}NO_4S$ 500.1890; Found: 500.1895.

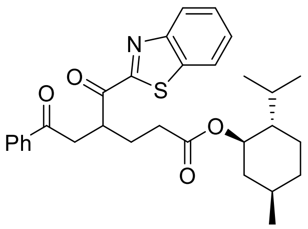

(1*R*,2*S*,5*R*)-2-isopropyl-5-methylcyclohexyl 4-(benzo[*d*]thiazole-2-carbonyl)-6-oxo-6-phenylhexanoate (4k)

The d.r.=1:1 was determined by ^{1}H NMR.

65.7 mg, 65% yield, slight yellow oil. Eluent: pentane/ethyl acetate = 5:1-3:1

^{1}H NMR (400 MHz, CDCl$_3$) δ 8.22 (d, *J* = 7.5 Hz, 1H), 8.01 – 7.93 (m, 3H), 7.61 – 7.49 (m, 3H), 7.45 (t, *J* = 7.7 Hz, 2H), 4.70 – 4.62 (m, 1H), 4.60 – 4.52 (m, 1H), 3.86 – 3.75 (m, 1H), 3.44 – 3.34 (m, 1H), 2.52 – 2.38 (m, 2H), 2.28 – 2.17 (m, 1H), 2.16 – 2.07 (m, 1H), 1.97 (d, *J* = 11.1 Hz, 1H), 1.89 (d, *J* = 11.4 Hz, 1H), 1.84 – 1.77 (m, 1H), 1.68 – 1.60 (m, 3H), 1.49 – 1.41 (m, 1H), 1.30 (d, *J* = 11.4 Hz, 1H), 1.04 – 0.98 (m, 1H), 0.89 – 0.83 (m, 7H), 0.71 (dd, *J* = 6.9, 3.2 Hz, 3H).

^{13}C NMR (101 MHz, CDCl$_3$) δ 197.7, 197.2, 172.3, 166.3, 153.7, 137.6, 136.2, 133.4, 128.6, 128.2, 127.6, 126.8, 125.8, 122.4, 74.4, 46.9, 41.5, 41.2, 40.8, 34.2, 32.1, 31.3, 27.1, 26.2, 23.3, 22.0, 20.8, 16.2.

HRMS (ESI-TOF) m/z: $[M + H]^+$ Calcd for $C_{30}H_{36}NO_4S$ 506.2360; Found: 506.2364.

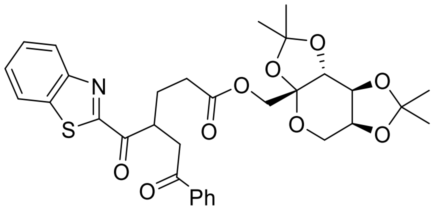

((3a*R*,5a*S*,8a*S*,8b*R*)-2,2,7,7-tetramethyltetrahydro-3a*H*-bis([1,3]dioxolo)[4,5-*b*:4',5'-*d*]pyran-3a-yl)methyl 4-(benzo[*d*]thiazole-2-carbonyl)-6-oxo-6-phenylhexanoate (4l)

The d.r.=1.2:1 was determined by ^{1}H NMR.

82.0 mg, 67% yield, slight yellow oil. Eluent: pentane/ethyl acetate = 5:1-3:1

^{1}H NMR (400 MHz, CDCl$_3$) δ 8.20 (d, *J* = 8.3 Hz, 1H), 7.96 (dd, *J* = 16.1, 7.6 Hz, 3H), 7.60 – 7.50 (m, 3H), 7.45 (t, *J* = 7.7 Hz, 2H), 4.60 – 4.50 (m, 2H), 4.44 – 4.35 (m, 1H), 4.26 – 4.18 (m, 2H), 4.02 (dd, *J* = 11.7, 6.9 Hz, 1H), 3.88 – 3.70 (m, 3H), 3.40 (t, *J* = 4.2 Hz, 1H), 3.35 (t, *J* = 4.2 Hz, 1H), 2.60 – 2.45 (m, 2H), 2.27 – 2.10 (m, 2H), 1.48 (d, *J* = 8.3 Hz, 3H), 1.44 (d, *J* = 1.9 Hz, 3H), 1.33 – 1.28 (m, 6H).

^{13}C NMR (101 MHz, CDCl$_3$) δ 197.6, 197.2, 197.1, 172.1, 166.2, 153.7, 137.5, 136.1, 133.4, 128.6, 128.2, 127.6, 126.9, 125.8, 125.8, 122.4, 109.1, 108.7, 101.5, 70.7, 70.6, 70.0, 65.6, 65.5, 61.2, 41.5, 41.5, 41.2, 31.7, 26.9, 26.4, 26.4, 25.9, 25.1, 25.1, 24.0.

HRMS (ESI-TOF) m/z: $[M + H]^+$ Calcd for $C_{32}H_{36}NO_9S$ 610.2105; Found: 610.2112.

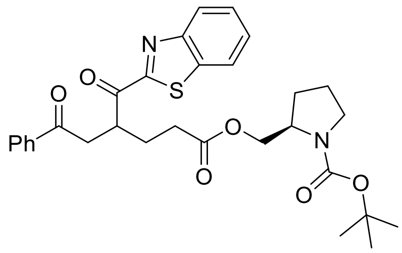

***tert*-butyl (2*R*)-2-(((4-(benzo[*d*]thiazole-2-carbonyl)-6-oxo-6-phenylhexanoyl)oxy)methyl)pyrrolidine-1-carboxylate (4m)**

The d.r. value was not determined.
67.0 mg, 61% yield, slight yellow oil. Eluent: pentane/ethyl acetate = 5:1-3:1
^{1}H NMR (400 MHz, $CDCl_3$) δ 8.23 – 8.17 (m, 1H), 8.00 – 7.92 (m, 3H), 7.59 – 7.49 (m, 3H), 7.44 (t, *J* = 7.7 Hz, 2H), 4.60 – 4.50 (m, 1H), 4.13 (dd, *J* = 11.4, 8.2 Hz, 1H), 4.07 – 3.88 (m, 2H), 3.79 (dd, *J* = 18.0, 9.9 Hz, 1H), 3.41 – 3.26 (m, 3H), 2.55 – 2.40 (m, 2H), 2.27 – 2.06 (m, 2H), 1.88 – 1.74 (m, 4H), 1.43 (s, 9H).
^{13}C NMR (101 MHz, $CDCl_3$) δ 197.6, 197.1, 172.5, 166.2, 153.7, 137.5, 136.1, 133.4, 128.6, 128.2, 127.6, 126.9, 125.7, 122.4, 79.7, 64.9, 55.4, 46.4, 41.5, 41.2, 31.8, 28.5, 27.0.
HRMS (ESI-TOF) m/z: $[M + H]^+$ Calcd for $C_{30}H_{35}N_2O_6S$ 551.2210; Found: 551.2205.

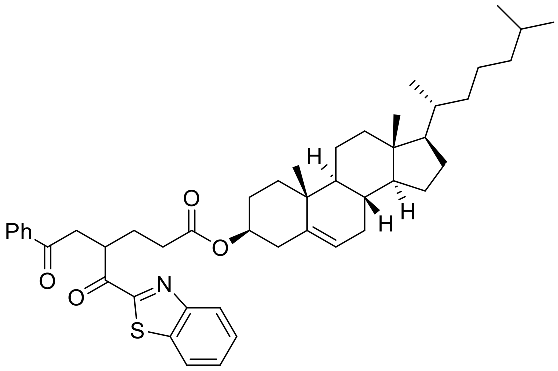

(3*S*,8*S*,9*S*,10*R*,13*R*,14*S*,17*R*)-10,13-dimethyl-17-((*R*)-6-methylheptan-2-yl)-2,3,4,7,8,9,10,11,12,13,14,15,16,17-tetradecahydro-1*H*-cyclopenta[*a*]phenanthren-3-yl 4-(benzo[*d*]thiazole-2-carbonyl)-6-oxo-6-phenylhexanoate (4n)

The d.r. value was not determined.
72.1 mg, 49% yield, slight yellow oil. Eluent: pentane/ethyl acetate = 5:1-3:1
^{1}H NMR (400 MHz, $CDCl_3$) δ 8.21 (d, *J* = 7.7 Hz, 1H), 8.01 – 7.93 (m, 3H), 7.60 – 7.51 (m, 3H), 7.45 (t, *J* = 7.7 Hz, 2H), 5.34 (dd, *J* = 9.4, 4.8 Hz, 1H), 4.62 – 4.53 (m, 2H), 3.80 (dd, *J* = 18.0, 10.0 Hz, 1H), 3.39 (dd, *J* = 18.0, 3.9 Hz, 1H), 2.50 – 2.38 (m, 2H), 2.30 – 2.17 (m, 3H), 2.15 – 2.07 (m, 1H), 2.02 – 1.93 (m, 2H), 1.86 – 1.77 (m, 3H), 1.58 – 1.45 (m, 6H), 1.31 (dd, *J* = 20.2, 15.2 Hz, 4H), 1.18 – 1.06 (m, 7H), 1.02 – 0.96 (m, 5H), 0.91 (d, *J* = 6.5 Hz, 4H), 0.86 (dd, *J* = 6.6, 1.7 Hz, 7H), 0.67 (s, 3H).

^{13}C NMR (101 MHz, CDCl$_3$) δ 197.7, 197.3, 172.1, 166.3, 153.7, 139.6, 137.6, 136.1, 133.4, 128.6, 128.2, 127.6, 126.8, 125.8, 122.7, 122.4, 74.2, 56.7, 56.1, 50.0, 42.3, 41.6, 41.1, 39.7, 39.5, 38.1, 38.0, 37.0, 36.6, 36.2, 35.8, 32.2, 31.9, 31.9, 28.2, 28.0, 27.7, 27.7, 27.1, 24.3, 23.8, 22.8, 22.6, 21.0, 19.3, 18.7, 11.9.
HRMS (ESI-TOF) m/z: $[M + H]^+$ Calcd for $C_{47}H_{62}NO_4S$ 736.4394; Found: 736.4401.

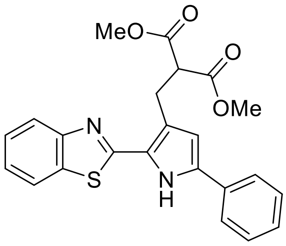

dimethyl 2-((2-(benzo[*d*]thiazol-2-yl)-5-phenyl-1*H*-pyrrol-3-yl)methyl)malonate (5)

75.6 mg, 90% yield, slight yellow oil. Eluent: pentane/ethyl acetate = 5:1-3:1
^{1}H NMR (400 MHz, CDCl$_3$) δ 9.84 (s, 1H), 7.92 (d, *J* = 8.0 Hz, 1H), 7.86 (d, *J* = 7.9 Hz, 1H), 7.58 (d, *J* = 7.2 Hz, 2H), 7.46 (t, *J* = 7.1 Hz, 1H), 7.39 (t, *J* = 7.7 Hz, 2H), 7.32 (t, *J* = 7.1 Hz, 1H), 7.28 (d, *J* = 7.4 Hz, 1H), 6.47 (d, *J* = 2.8 Hz, 1H), 3.89 (t, *J* = 7.8 Hz, 1H), 3.76 (s, 6H), 3.51 (d, *J* = 7.8 Hz, 2H).
^{13}C NMR (101 MHz, CDCl$_3$) δ 169.4, 158.0, 152.6, 134.9, 134.0, 131.1, 129.0, 127.5, 126.5, 124.6, 124.5, 124.4, 124.3, 121.8, 121.5, 109.4, 52.7, 52.3, 26.7.
HRMS (ESI-TOF) m/z: $[M + H]^+$ Calcd for $C_{23}H_{21}N_2O_4S$ 421.1217; Found: 421.1218.

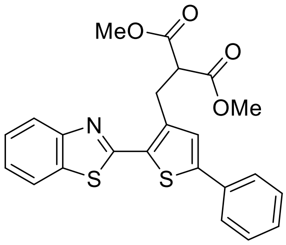

dimethyl 2-((2-(benzo[*d*]thiazol-2-yl)-5-phenylthiophen-3-yl)methyl)malonate (6)

55.6 mg, 64% yield, slight yellow solid. Eluent: pentane/ethyl acetate = 5:1-3:1
^{1}H NMR (400 MHz, CDCl$_3$) δ 8.02 (d, *J* = 8.1 Hz, 1H), 7.87 (d, *J* = 7.6 Hz, 1H), 7.66 – 7.60 (m, 2H), 7.48 (t, *J* = 8.3 Hz, 1H), 7.43 – 7.31 (m, 4H), 7.24 (s, 1H), 4.12 (t, *J* = 7.7 Hz, 1H), 3.75 (s, 6H), 3.70 (d, *J* = 7.7 Hz, 2H).
^{13}C NMR (101 MHz, CDCl$_3$) δ 169.4, 160.0, 153.5, 146.1, 140.1, 134.7, 133.2, 131.7, 129.1, 128.5, 127.5, 126.5, 125.9, 125.2, 123.1, 121.4, 52.7, 51.8, 29.3.
HRMS (ESI-TOF) m/z: $[M + H]^+$ Calcd for $C_{23}H_{20}NO_4S_2$ 438.0828; Found: 438.0827.

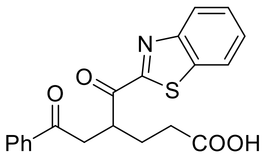

4-(benzo[*d*]thiazole-2-carbonyl)-6-oxo-6-phenylhexanoic acid (7)

51.2 mg, 70% yield, slight yellow solid. Eluent: pentane/ethyl acetate = 3:1-1:1

^{1}H NMR (400 MHz, CDCl$_3$) δ 8.20 (d, *J* = 8.0 Hz, 1H), 7.96 (dd, *J* = 12.8, 7.6 Hz, 3H), 7.60 – 7.49 (m, 3H), 7.44 (t, *J* = 7.7 Hz, 2H), 4.63 – 4.52 (m, 1H), 3.81 (dd, *J* = 18.0, 9.8 Hz, 1H), 3.38 (dd, *J* = 18.0, 4.3 Hz, 1H), 2.58 – 2.44 (m, 2H), 2.25 – 2.07 (m, 2H).

^{13}C NMR (101 MHz, CDCl$_3$) δ 197.6, 197.2, 178.3, 166.3, 153.6, 137.5, 136.1, 133.5, 128.7, 128.2, 127.7, 127.0, 125.7, 122.4, 41.6, 41.1, 31.6, 26.8.

HRMS (ESI-TOF) m/z: [M + H]$^+$ Calcd for $C_{21}H_{17}NO_4S$ 368.0951; Found: 368.0952.

7. The NMR Spectrum

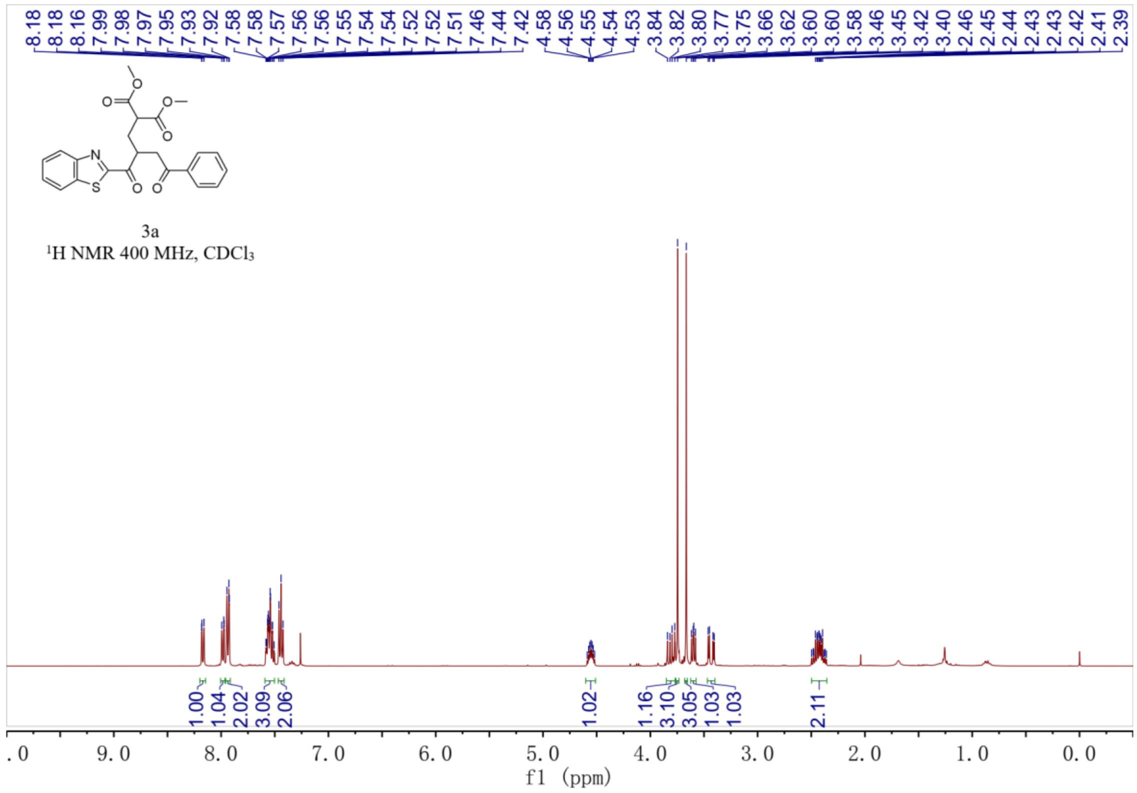

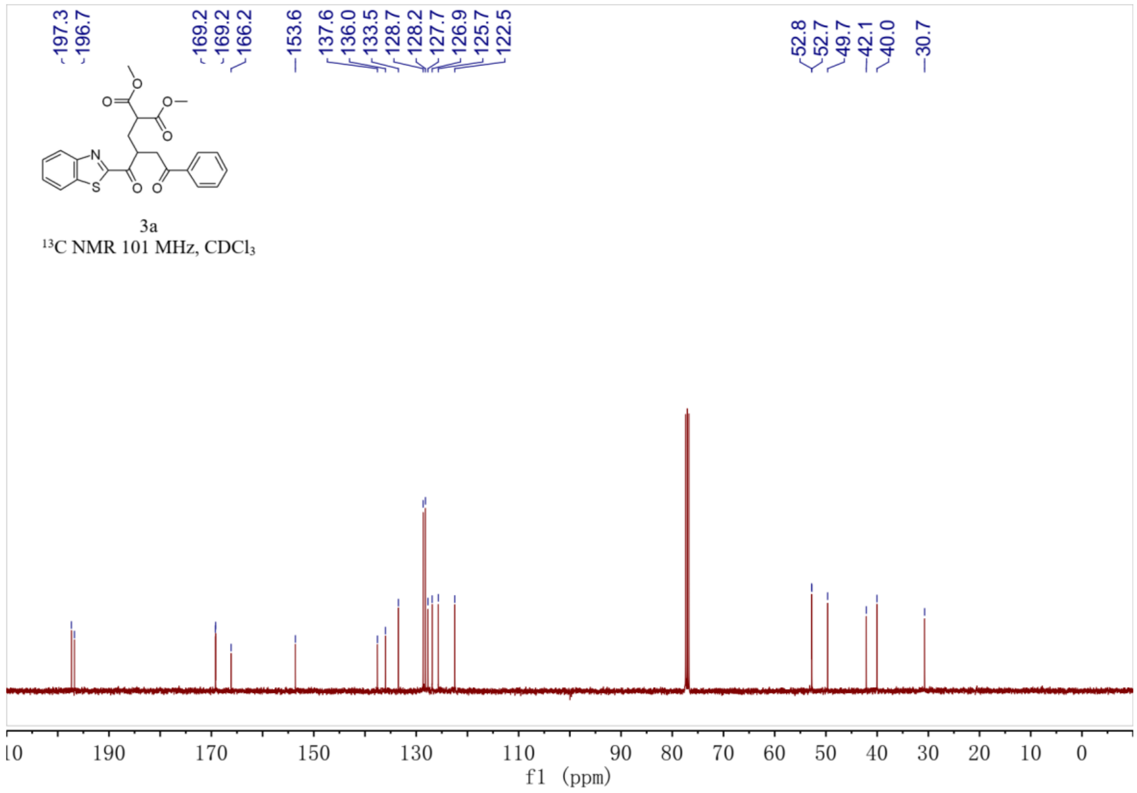

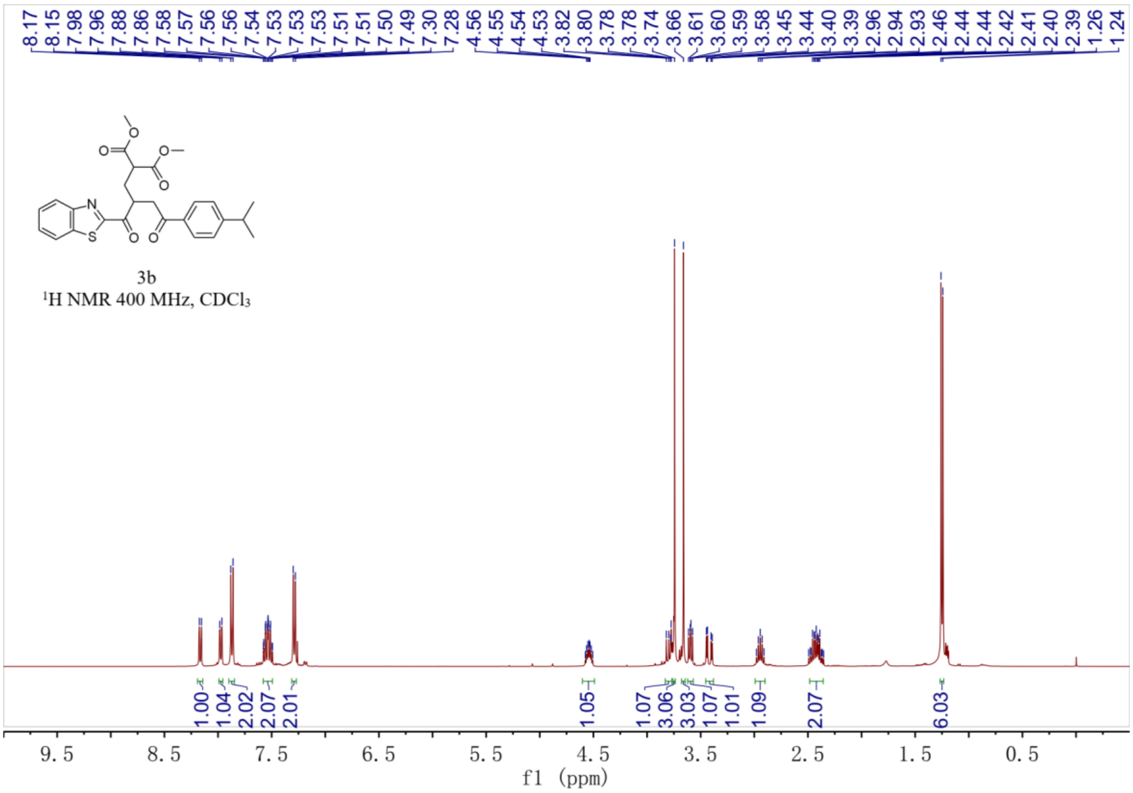
8.17
8.15
7.98
7.96
7.88
7.86
7.58
7.57
7.56
7.56
7.54
7.53
7.53
7.53
7.51
7.51
7.50
7.49
7.30
7.28
4.56
4.55
4.54
4.53
3.82
3.80
3.78
3.78
3.74
3.66
3.61
3.60
3.59
3.58
3.45
3.44
3.40
3.39
2.96
2.94
2.93
2.46
2.44
2.44
2.42
2.41
2.40
2.39
1.26
1.24
3b
^{1}H NMR 400 MHz, CDCl$_3$
1.00
1.04
2.02
2.07
2.01
1.05
1.07
3.06
3.03
1.07
1.01
1.09
2.07
6.03
9.5
8.5
7.5
6.5
5.5
4.5
3.5
2.5
1.5
0.5
f1 (ppm)

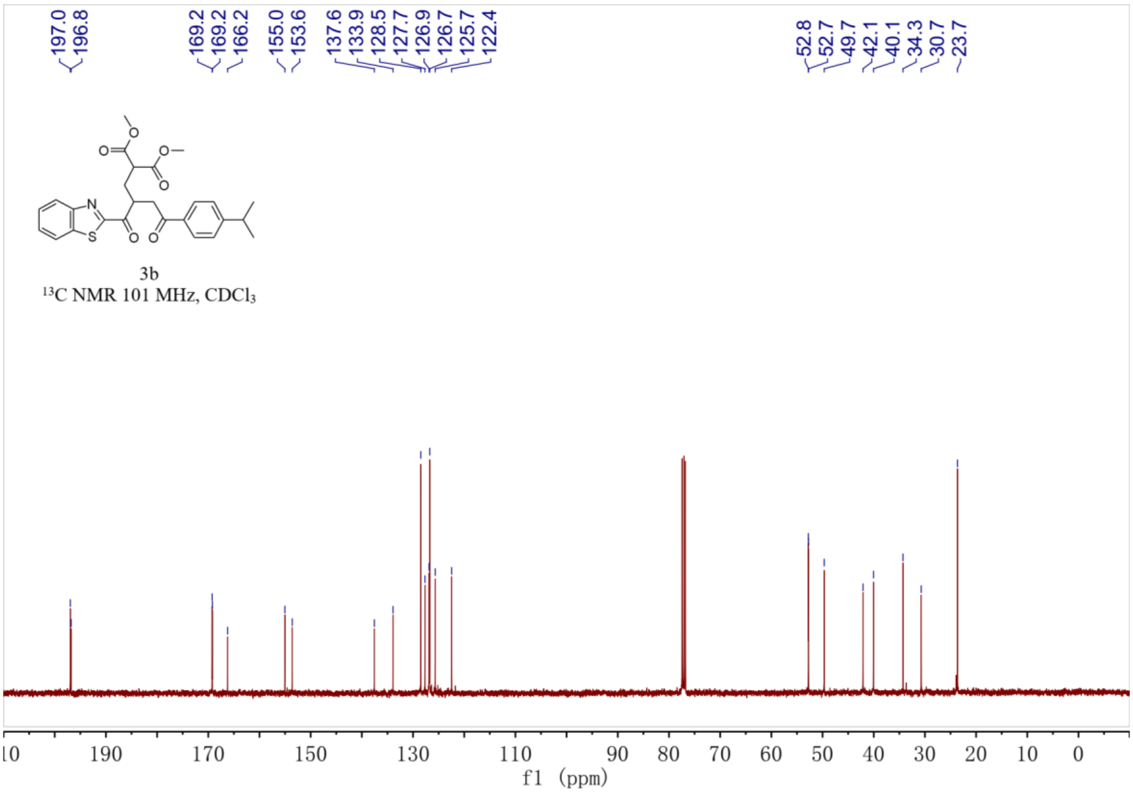
197.0
196.8
169.2
169.2
166.2
155.0
153.6
137.6
133.9
128.5
127.7
126.9
126.7
125.7
122.4
52.8
52.7
49.7
42.1
40.1
34.3
30.7
23.7
3b
^{13}C NMR 101 MHz, CDCl$_3$
190
170
150
130
110
90
80
70
60
50
40
30
20
10
0
f1 (ppm)

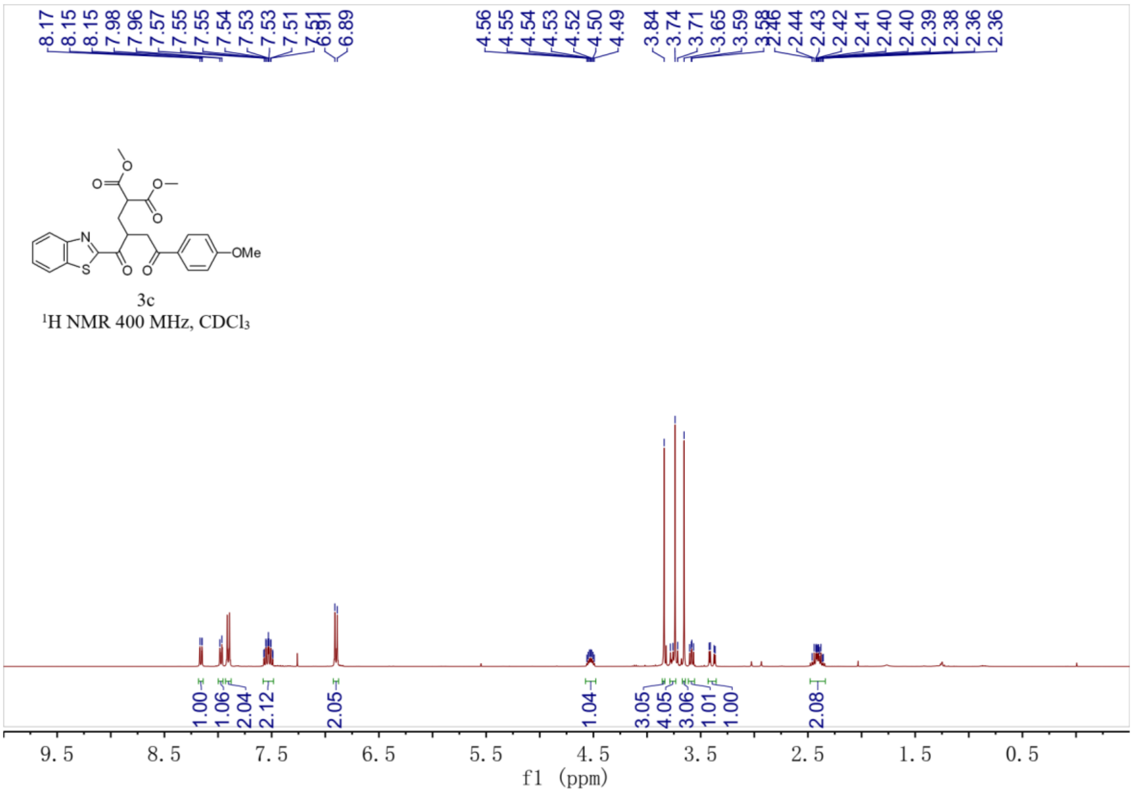

8.17
8.15
8.15
7.98
7.96
7.57
7.55
7.55
7.54
7.53
7.53
7.51
7.51
6.91
6.89
4.56
4.55
4.54
4.53
4.52
4.50
4.49
3.84
3.74
3.71
3.65
3.59
3.58
3.46
2.44
2.43
2.42
2.41
2.40
2.40
2.39
2.38
2.36
2.36
OMe
3c
1H NMR 400 MHz, CDCl3
1.00
1.06
2.04
2.12
2.05
1.04
3.05
4.05
3.06
1.01
1.00
2.08
9.5
8.5
7.5
6.5
5.5
4.5
3.5
2.5
1.5
0.5
f1 (ppm)

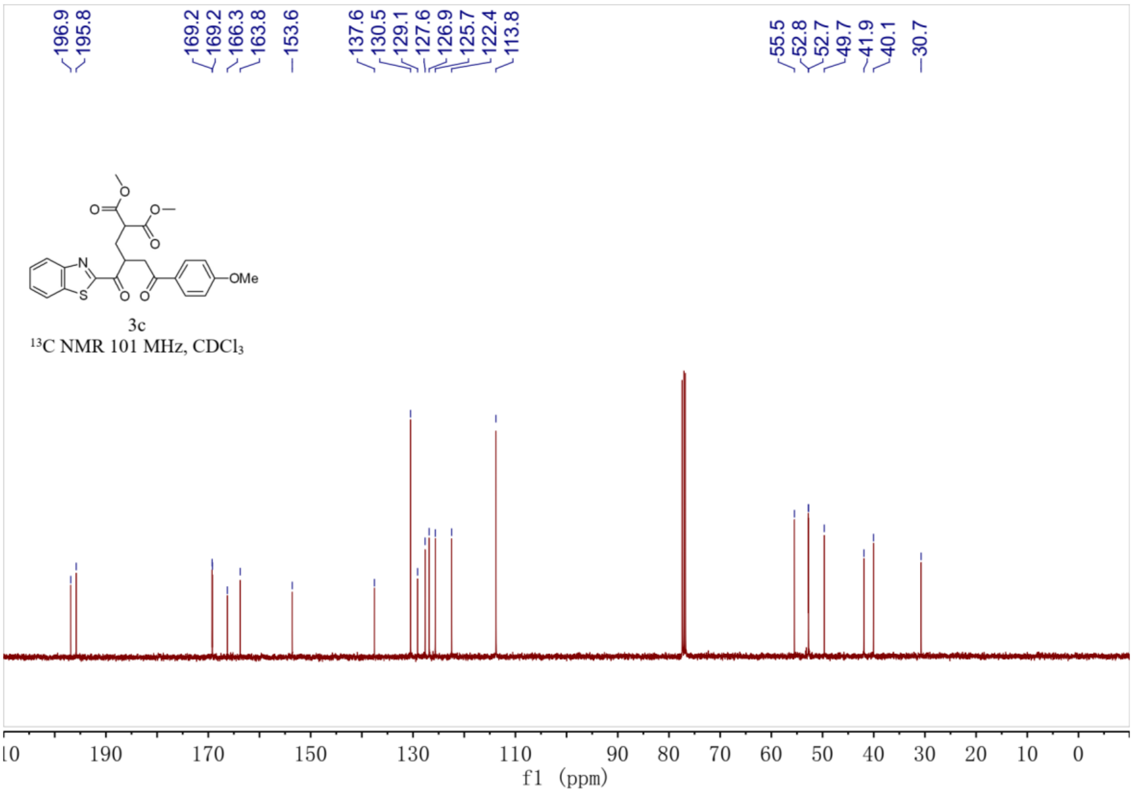

196.9
195.8
169.2
169.2
166.3
163.8
153.6
137.6
130.5
129.1
127.6
126.9
125.7
122.4
113.8
55.5
52.8
52.7
49.7
41.9
40.1
30.7
OMe
3c
13C NMR 101 MHz, CDCl3
190
170
150
130
110
90
80
70
60
50
40
30
20
10
0
f1 (ppm)

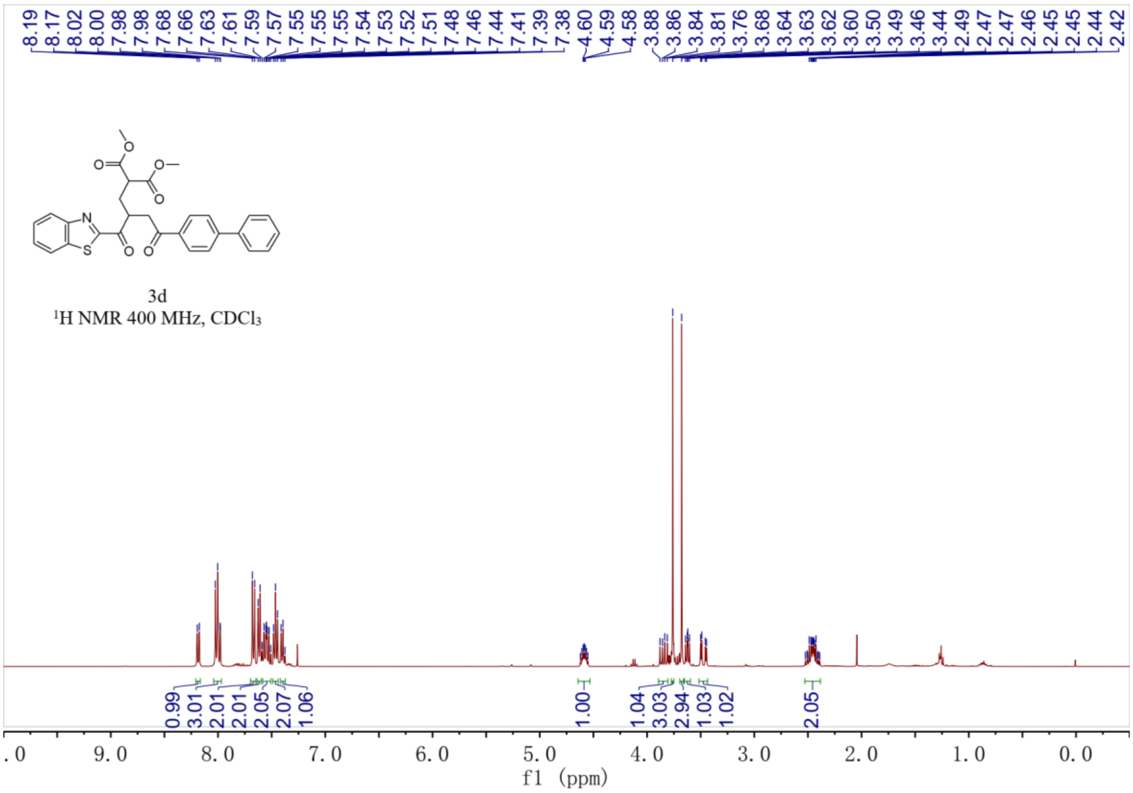
8.19 8.17 8.02 8.00 7.98 7.98 7.68 7.66 7.63 7.61 7.59 7.57 7.55 7.55 7.54 7.53 7.52 7.51 7.48 7.46 7.44 7.41 7.39 7.38 4.60 4.59 4.58 3.88 3.86 3.84 3.81 3.76 3.68 3.64 3.63 3.62 3.60 3.50 3.49 3.46 3.44 2.49 2.47 2.47 2.46 2.45 2.45 2.44 2.42
3d
1H NMR 400 MHz, CDCl3
0.99 3.01 2.01 2.01 2.05 2.07 1.06 1.00 1.04 3.03 2.94 1.03 1.02 2.05
f1 (ppm)

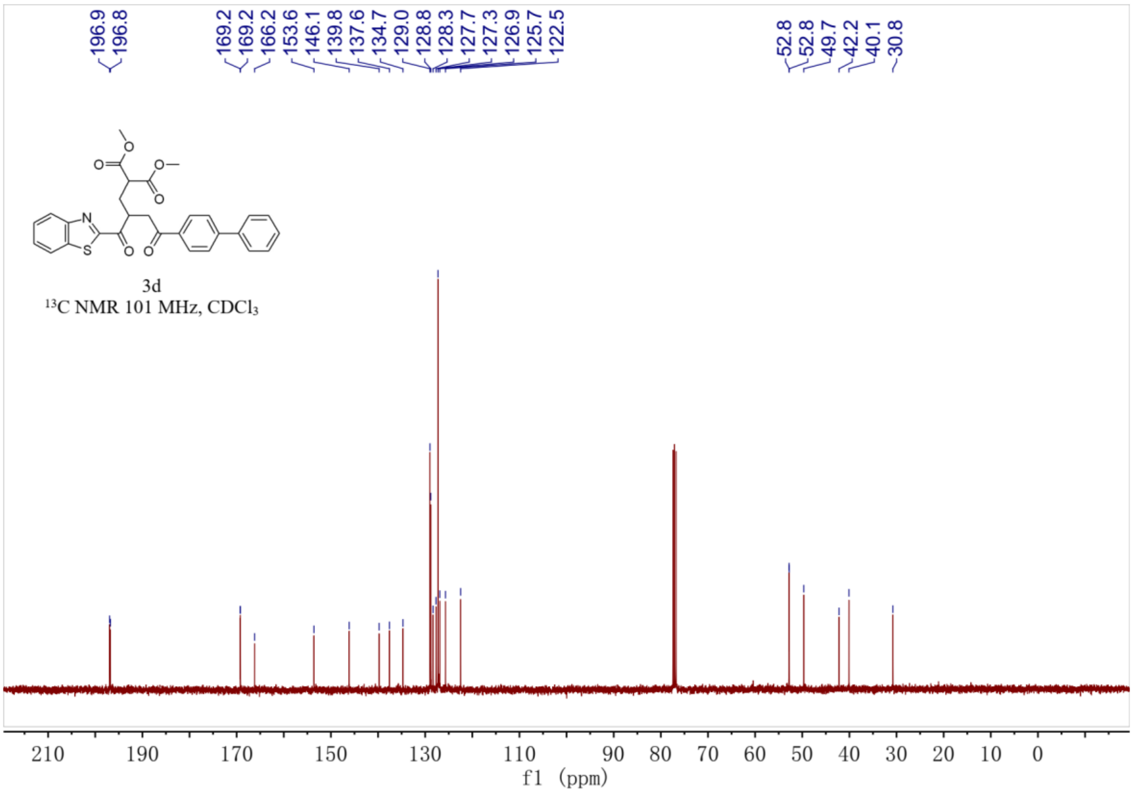
196.9 196.8 169.2 169.2 166.2 153.6 146.1 139.8 137.6 134.7 129.0 128.8 128.3 127.7 127.3 126.9 125.7 122.5 52.8 52.8 49.7 42.2 40.1 30.8
3d
13C NMR 101 MHz, CDCl3
f1 (ppm)

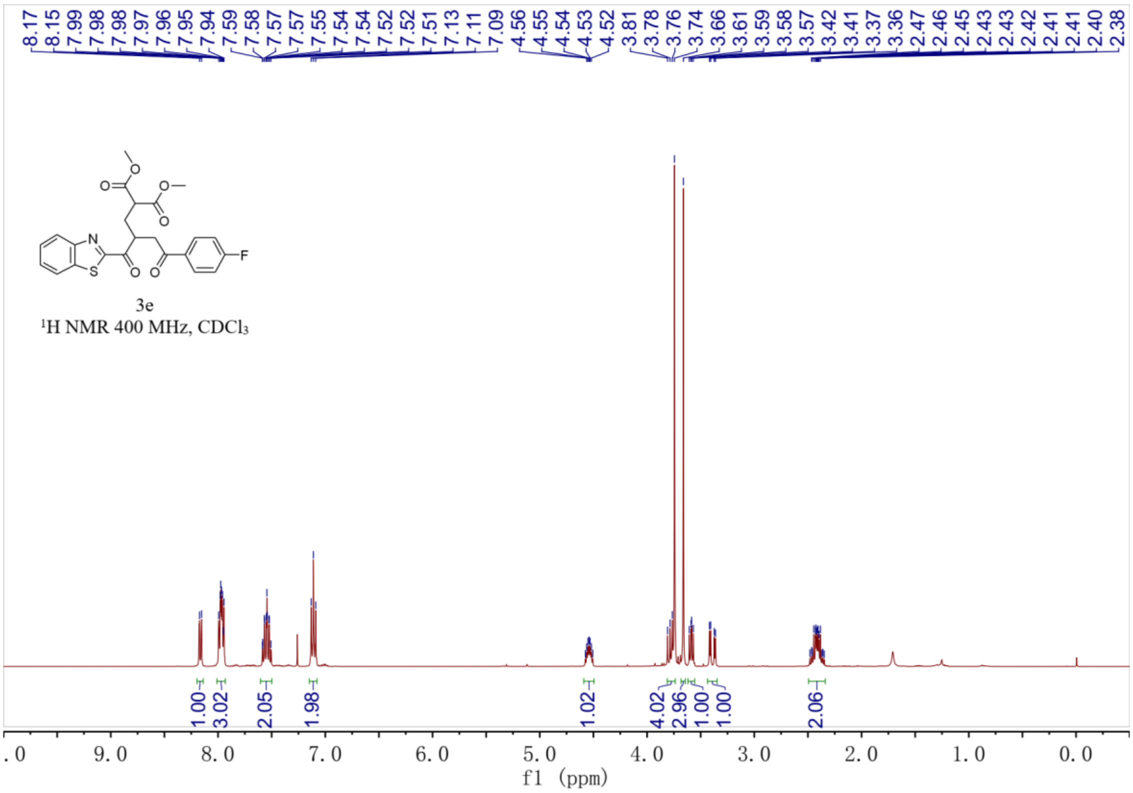
3e
^{1}H NMR 400 MHz, CDCl$_3$
8.17 8.15 7.99 7.98 7.97 7.96 7.95 7.94 7.59 7.58 7.57 7.57 7.55 7.54 7.54 7.52 7.52 7.51 7.13 7.11 7.09 4.56 4.55 4.54 4.53 4.52 3.81 3.78 3.76 3.74 3.66 3.61 3.59 3.58 3.57 3.42 3.41 3.37 3.36 2.47 2.46 2.45 2.43 2.43 2.42 2.41 2.41 2.40 2.38
1.00 3.02 2.05 1.98 1.02 4.02 2.96 1.00 1.00 2.06
f1 (ppm)

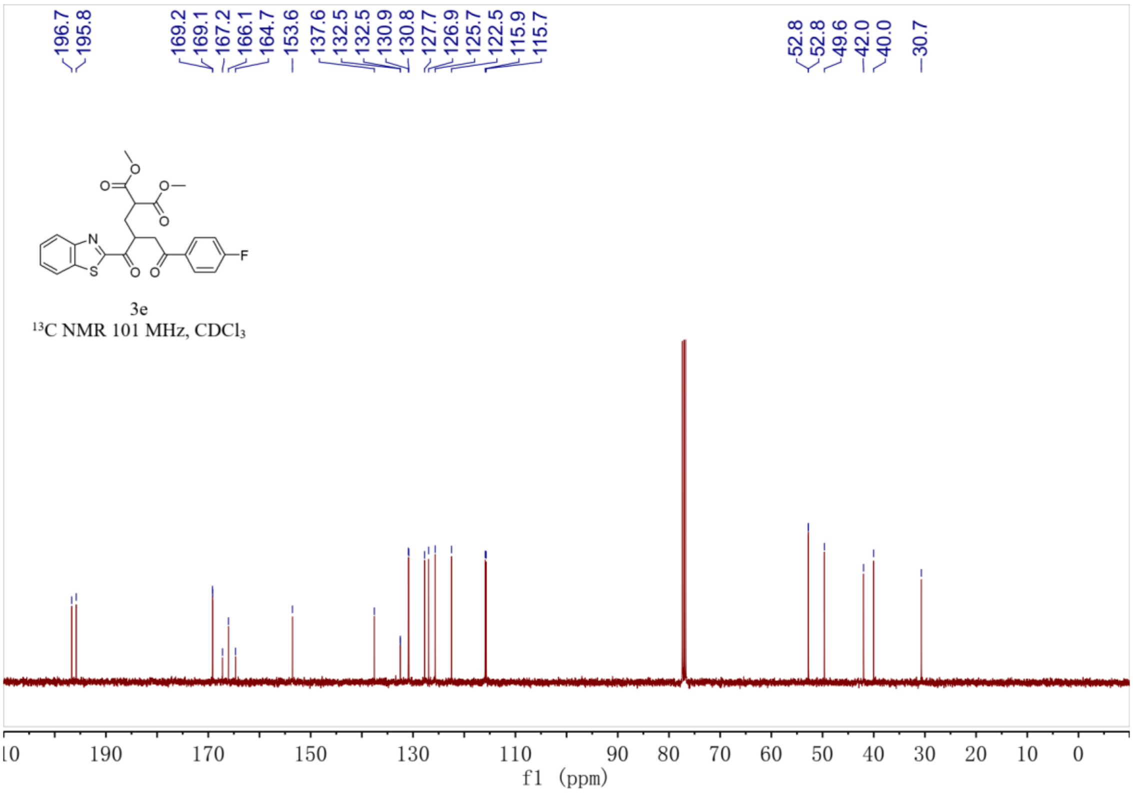
3e
^{13}C NMR 101 MHz, CDCl$_3$
196.7 195.8 169.2 169.1 167.2 166.1 164.7 153.6 137.6 132.5 132.5 130.9 130.8 127.7 126.9 125.7 122.5 115.9 115.7 52.8 52.8 49.6 42.0 40.0 30.7
f1 (ppm)

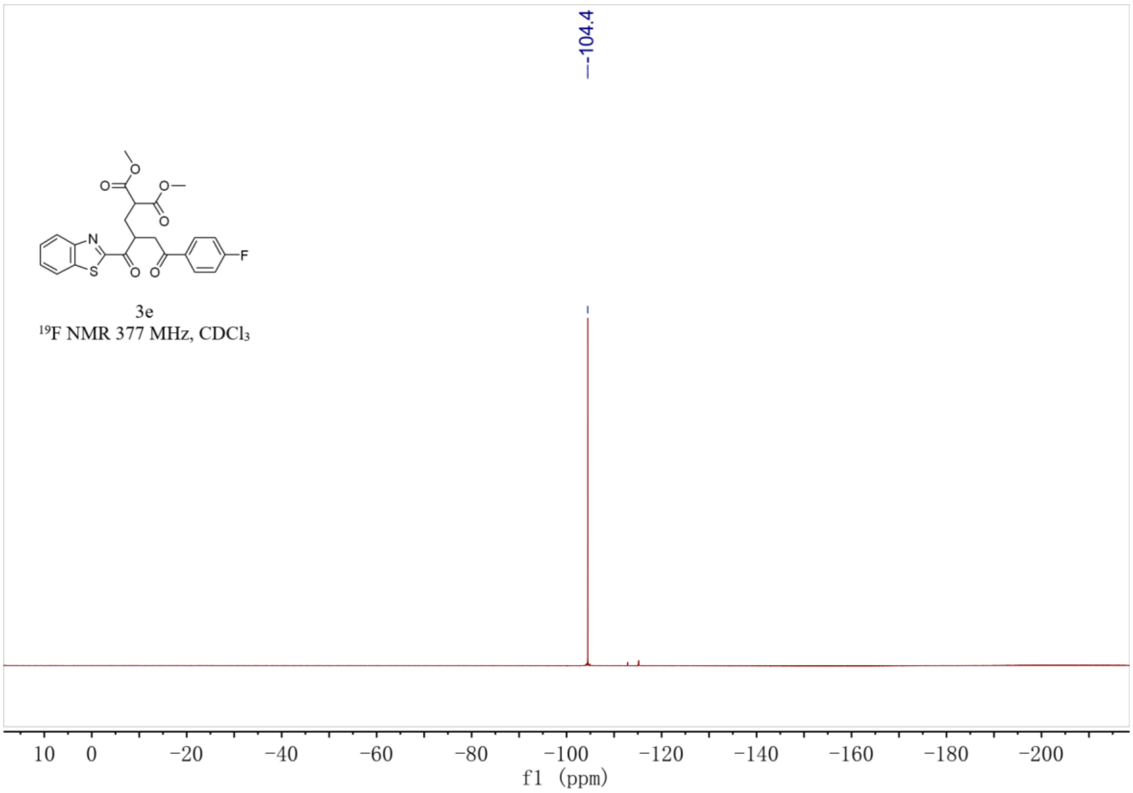
-104.4
3e
19F NMR 377 MHz, CDCl3
10
0
−20
−40
−60
−80
−100
−120
−140
−160
−180
−200
f1 (ppm)

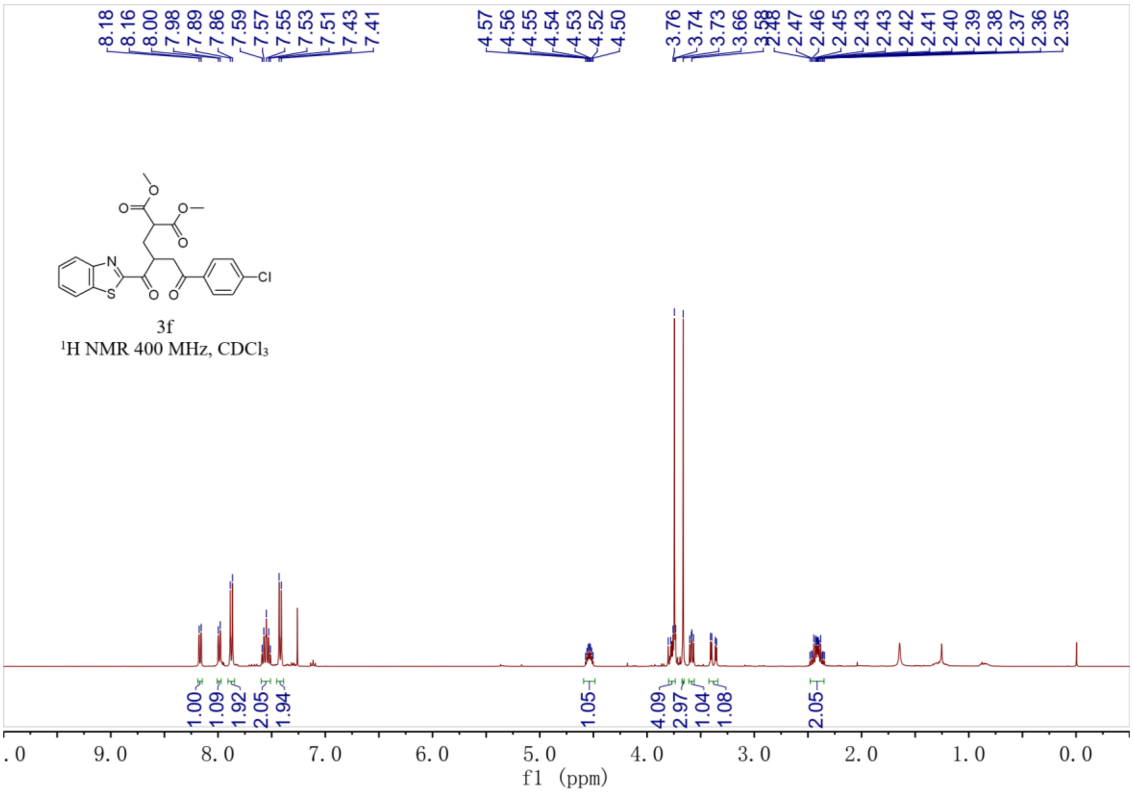
8.18
8.16
8.00
7.98
7.89
7.86
7.59
7.57
7.55
7.53
7.51
7.43
7.41
4.57
4.56
4.55
4.54
4.53
4.52
4.50
3.76
3.74
3.73
3.66
3.58
3.48
2.47
2.46
2.45
2.43
2.43
2.42
2.41
2.40
2.39
2.38
2.37
2.36
2.35
3f
^{1}H NMR 400 MHz, CDCl$_3$
1.00
1.09
1.92
2.05
1.94
1.05
4.09
2.97
1.04
1.08
2.05
f1 (ppm)

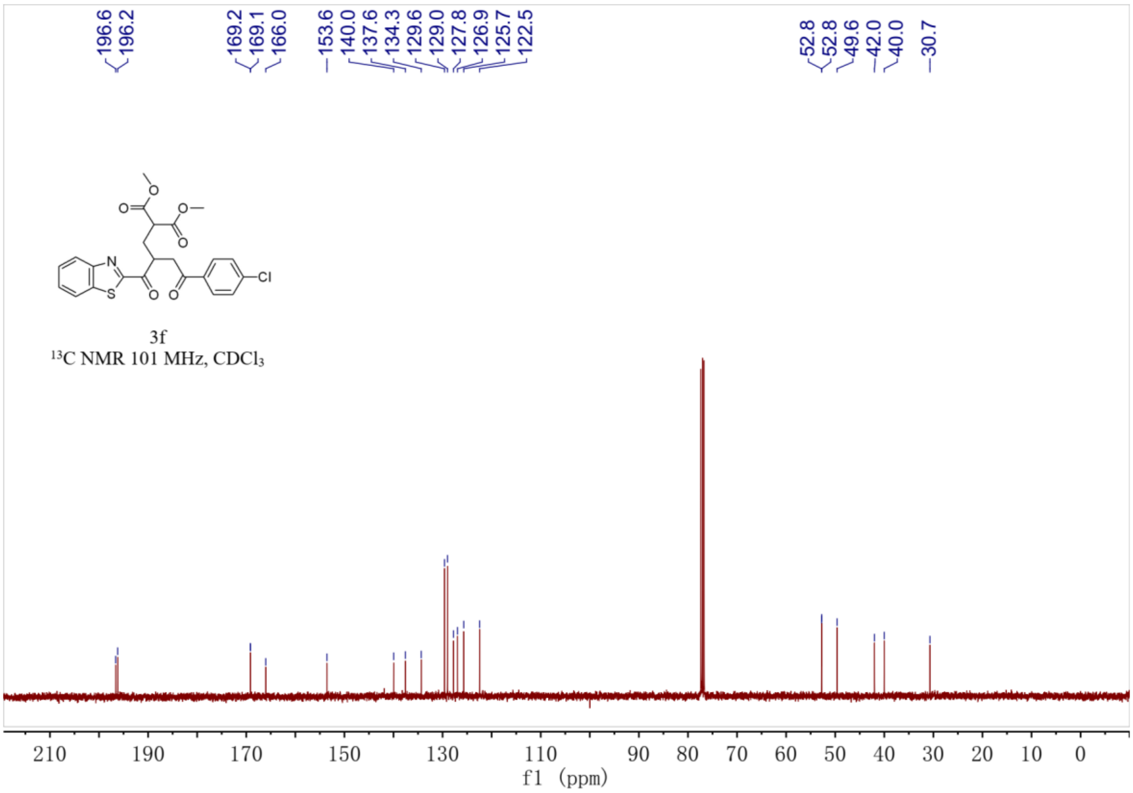
196.6
196.2
169.2
169.1
166.0
153.6
140.0
137.6
134.3
129.6
129.0
127.8
126.9
125.7
122.5
52.8
52.8
49.6
42.0
40.0
30.7
3f
^{13}C NMR 101 MHz, CDCl$_3$
f1 (ppm)

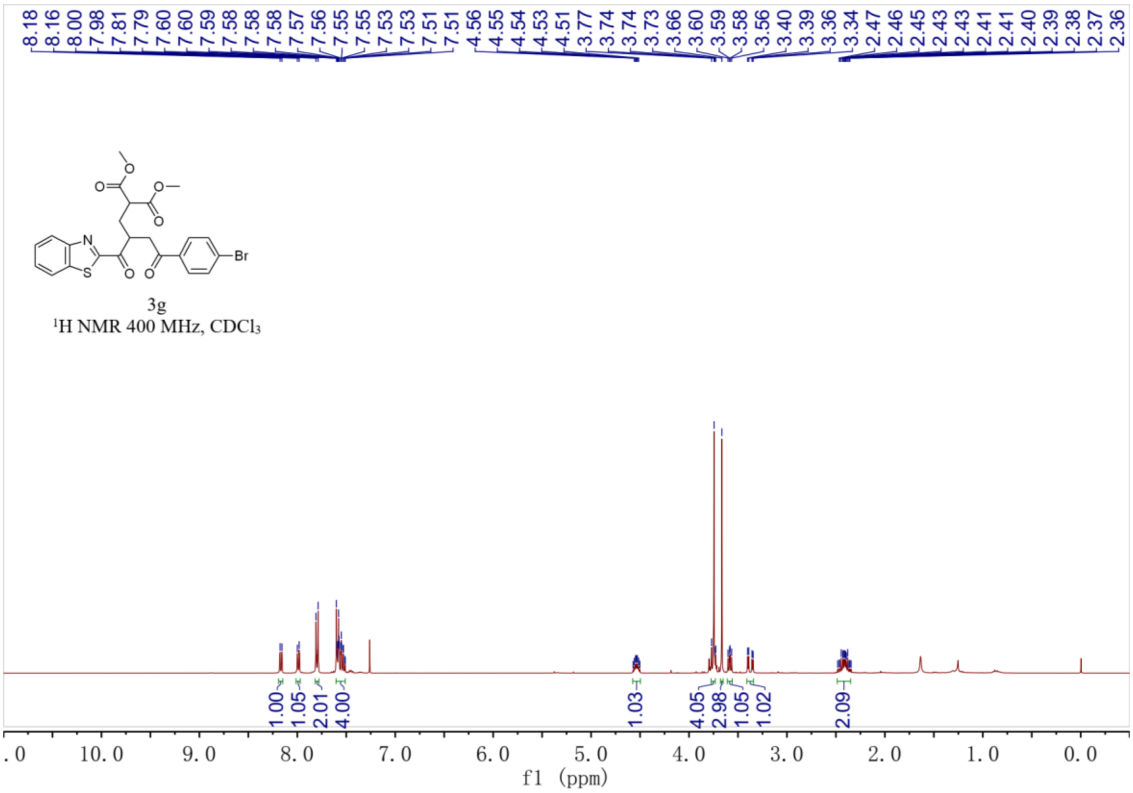
8.18 8.16 8.00 7.98 7.81 7.79 7.60 7.60 7.59 7.58 7.58 7.58 7.57 7.56 7.55 7.55 7.53 7.53 7.51 7.51 4.56 4.55 4.54 4.53 4.51 3.77 3.74 3.74 3.73 3.66 3.60 3.59 3.58 3.56 3.40 3.39 3.36 3.34 2.47 2.46 2.45 2.43 2.43 2.41 2.41 2.40 2.39 2.38 2.37 2.36
Br
N
S
O
3g
¹H NMR 400 MHz, CDCl₃
1.00 1.05 2.01 4.00 1.03 4.05 2.98 1.05 1.02 2.09
f1 (ppm)

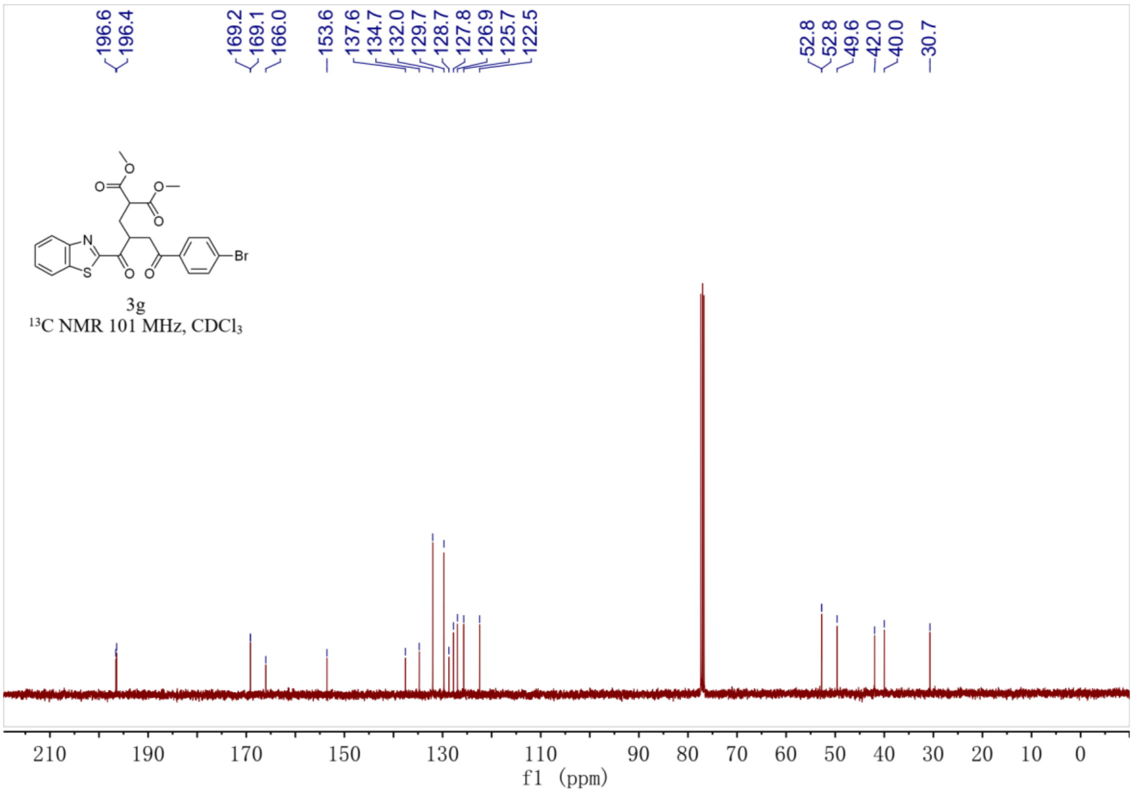
196.6 196.4 169.2 169.1 166.0 153.6 137.6 134.7 132.0 129.7 128.7 127.8 126.9 125.7 122.5 52.8 52.8 49.6 42.0 40.0 30.7
Br
N
S
O
3g
¹³C NMR 101 MHz, CDCl₃
f1 (ppm)

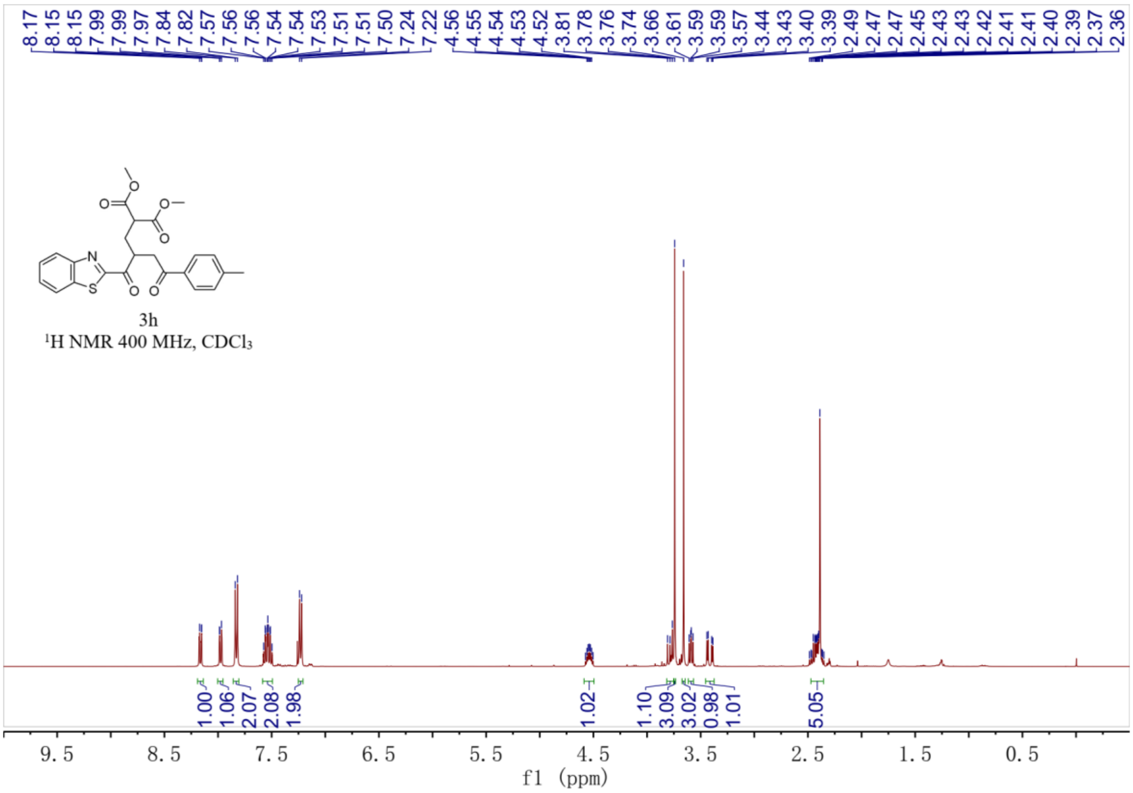

3h

^{1}H NMR 400 MHz, $CDCl_3$

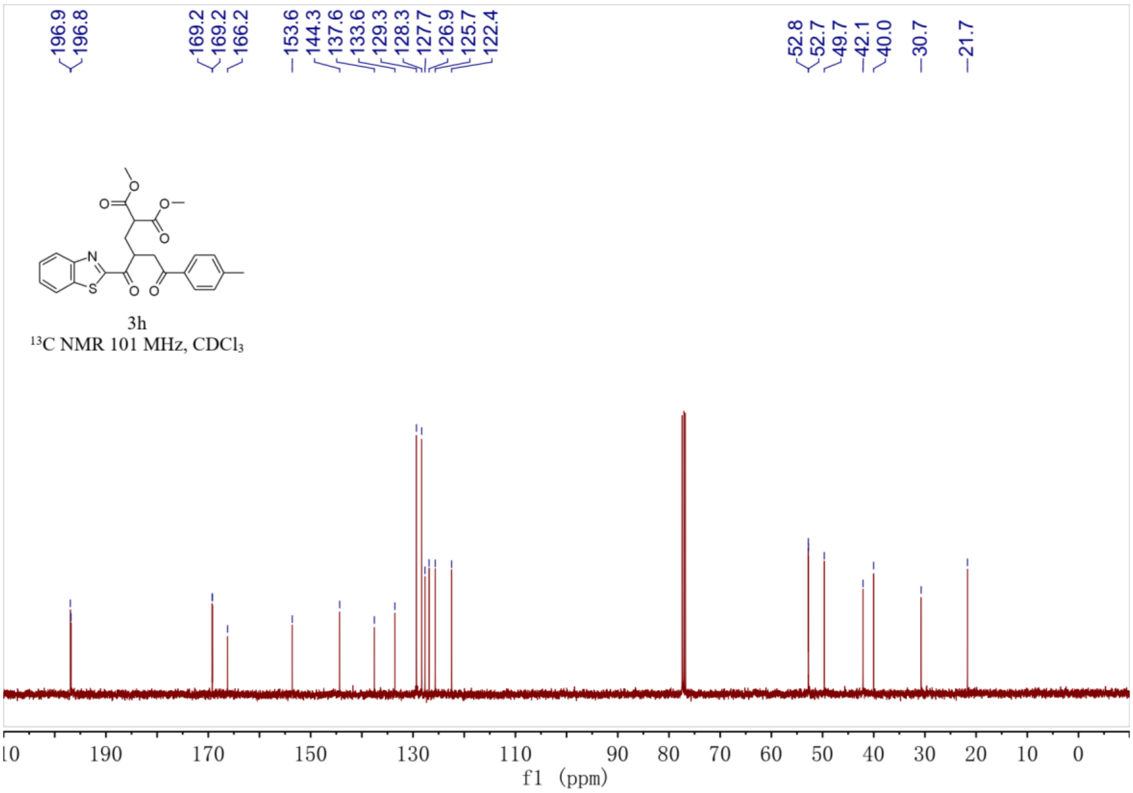

3h

^{13}C NMR 101 MHz, $CDCl_3$

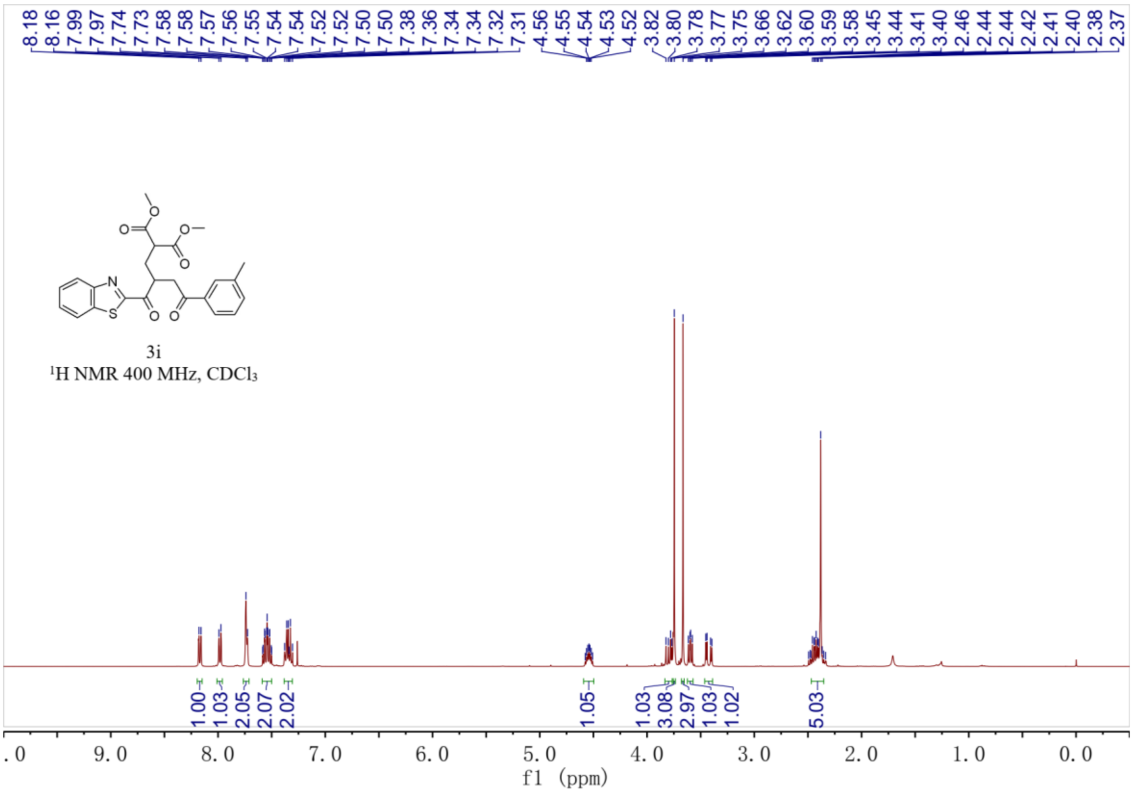
3i
¹H NMR 400 MHz, CDCl₃
f1 (ppm)

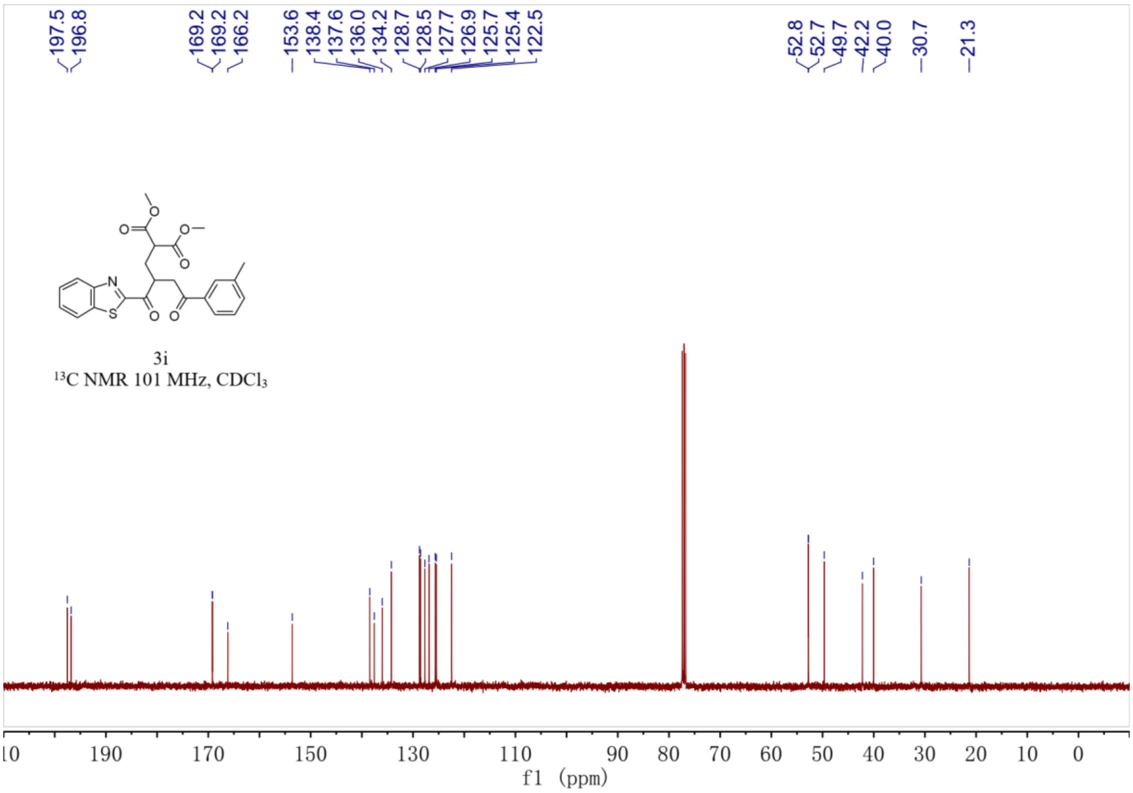
3i
¹³C NMR 101 MHz, CDCl₃
f1 (ppm)

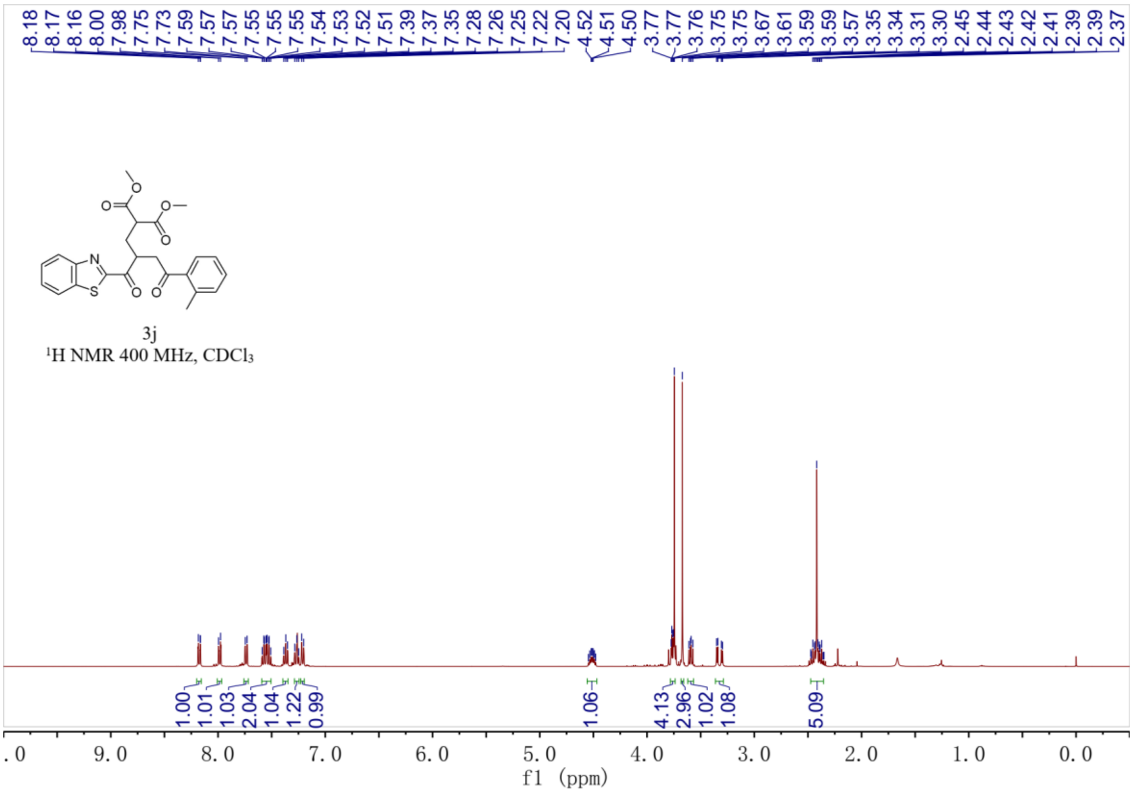
8.18 8.17 8.16 8.00 7.98 7.75 7.73 7.59 7.57 7.55 7.55 7.55 7.54 7.53 7.52 7.51 7.39 7.37 7.35 7.28 7.26 7.25 7.22 7.20 4.52 4.51 4.50 3.77 3.77 3.76 3.75 3.75 3.67 3.61 3.59 3.59 3.57 3.35 3.34 3.31 3.30 2.45 2.44 2.43 2.42 2.41 2.39 2.39 2.37
3j
1H NMR 400 MHz, CDCl3
1.00 1.01 1.03 2.04 1.04 1.22 0.99 1.06 4.13 2.96 1.02 1.08 5.09
f1 (ppm)

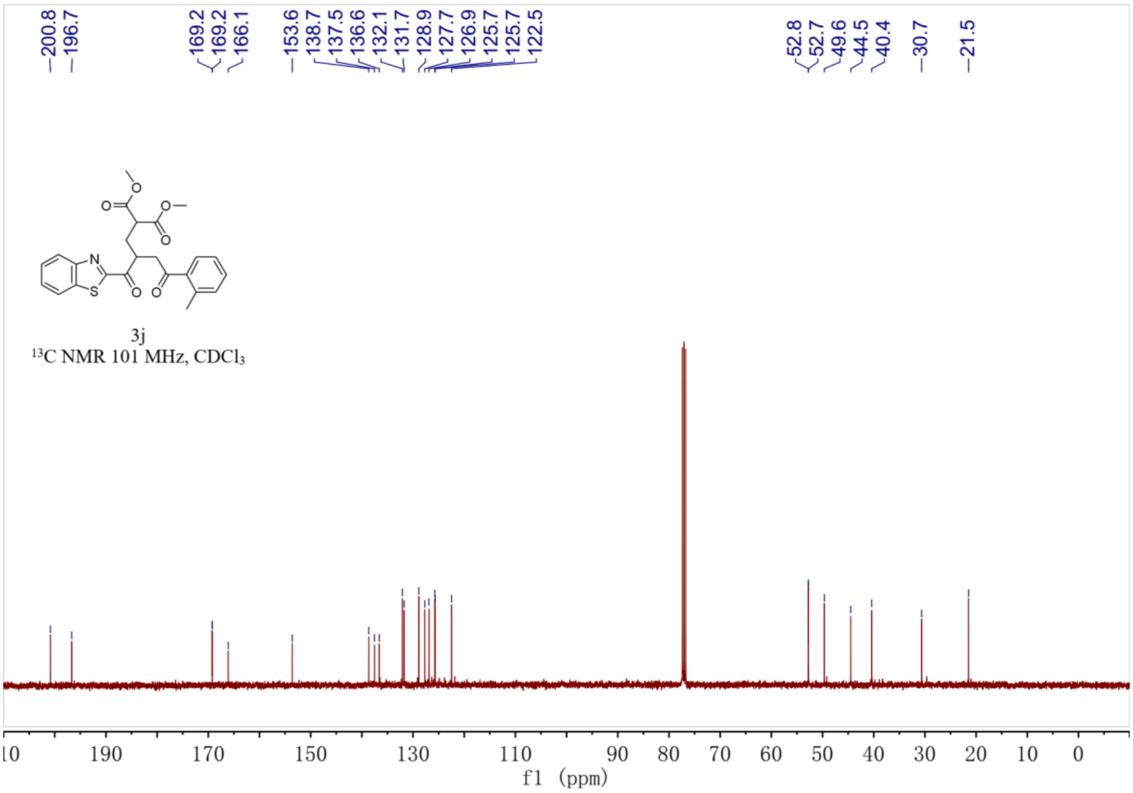
200.8 196.7 169.2 169.2 166.1 153.6 138.7 137.5 136.6 132.1 131.7 128.9 127.7 126.9 125.7 125.7 122.5 52.8 52.7 49.6 44.5 40.4 30.7 21.5
3j
13C NMR 101 MHz, CDCl3
f1 (ppm)

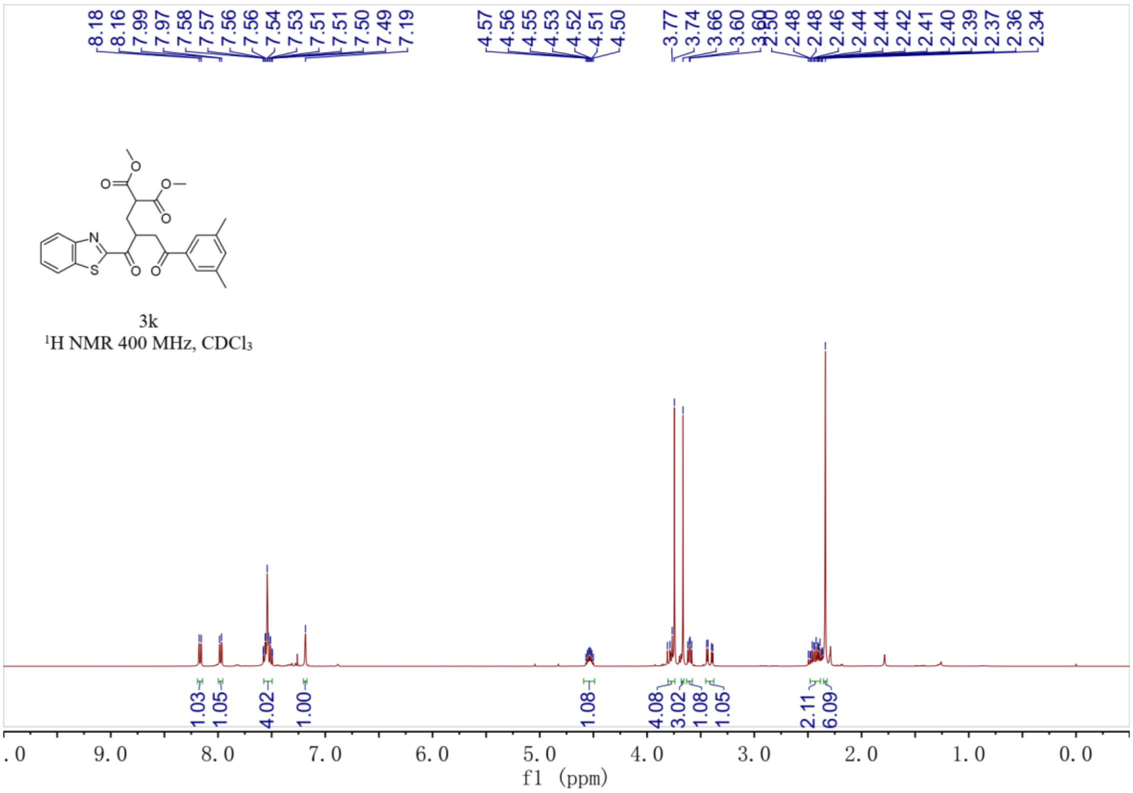
8.18
8.16
7.99
7.97
7.58
7.57
7.56
7.56
7.54
7.53
7.51
7.50
7.49
7.19
4.57
4.56
4.55
4.53
4.52
4.51
4.50
3.77
3.74
3.66
3.60
2.48
2.48
2.46
2.44
2.44
2.42
2.41
2.40
2.39
2.37
2.36
2.34
3k
¹H NMR 400 MHz, CDCl₃
1.03
1.05
4.02
1.00
1.08
4.08
3.02
1.08
1.05
2.11
6.09
9.0
8.0
7.0
6.0
5.0
4.0
3.0
2.0
1.0
0.0
f1 (ppm)

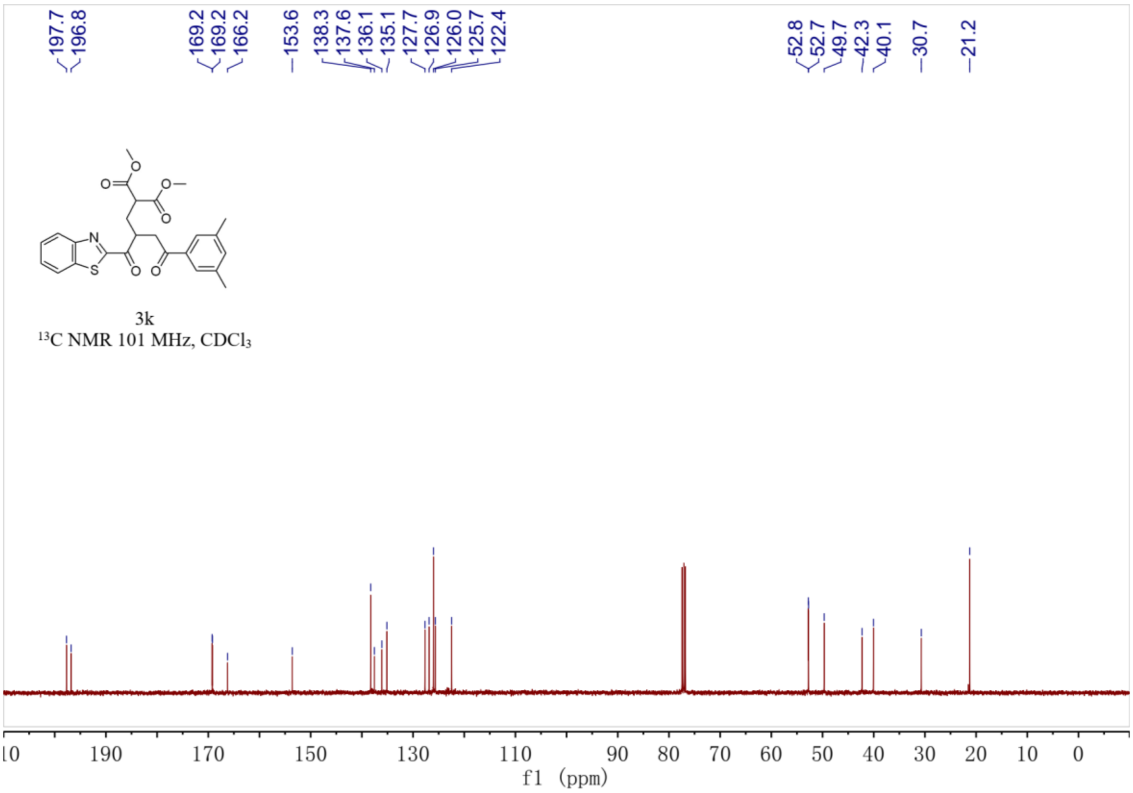
197.7
196.8
169.2
169.2
166.2
153.6
138.3
137.6
136.1
135.1
127.7
126.9
126.0
125.7
122.4
52.8
52.7
49.7
42.3
40.1
30.7
21.2
3k
¹³C NMR 101 MHz, CDCl₃
190
170
150
130
110
90
80
70
60
50
40
30
20
10
0
f1 (ppm)

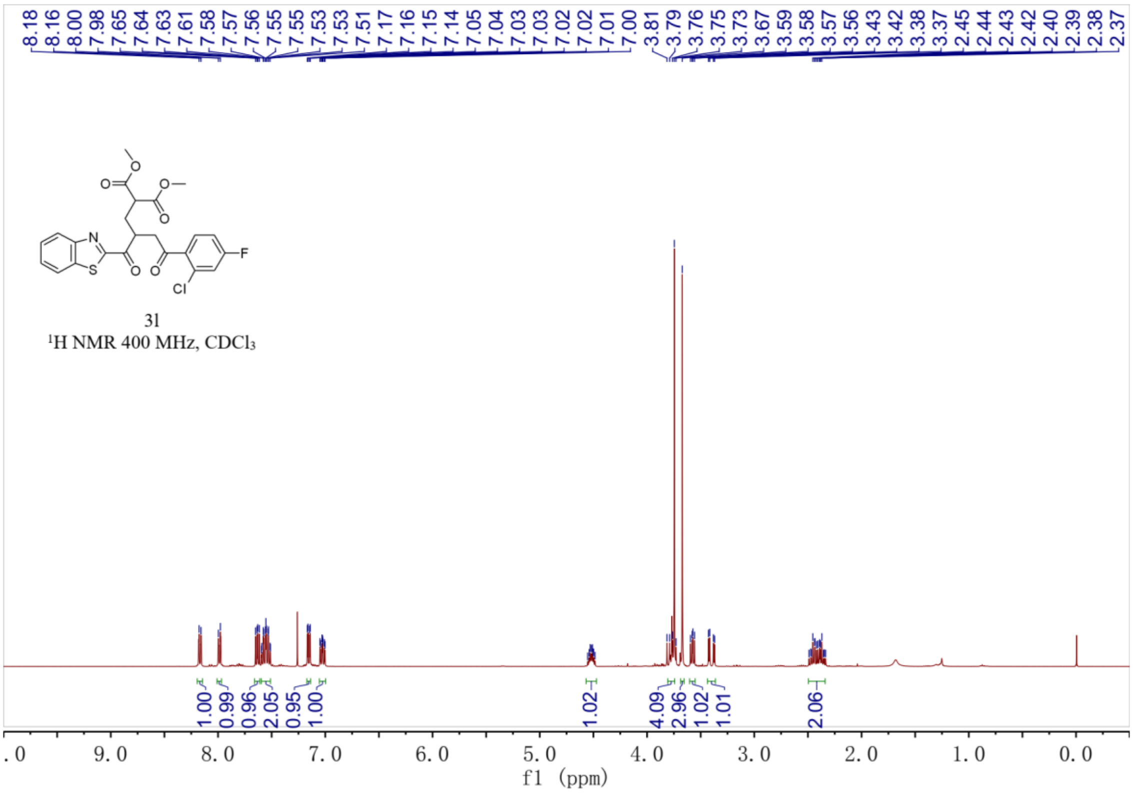
8.18 8.16 8.00 7.98 7.65 7.64 7.63 7.61 7.58 7.57 7.56 7.55 7.55 7.53 7.53 7.51 7.17 7.16 7.15 7.14 7.05 7.04 7.03 7.03 7.02 7.02 7.01 7.00 3.81 3.79 3.76 3.75 3.73 3.67 3.59 3.58 3.57 3.56 3.43 3.42 3.38 3.37 2.45 2.44 2.43 2.42 2.40 2.39 2.38 2.37
N
S
O
F
Cl
3l
1H NMR 400 MHz, CDCl3
1.00 0.99 0.96 2.05 0.95 1.00 1.02 4.09 2.96 1.02 1.01 2.06
.0 9.0 8.0 7.0 6.0 5.0 4.0 3.0 2.0 1.0 0.0
f1 (ppm)

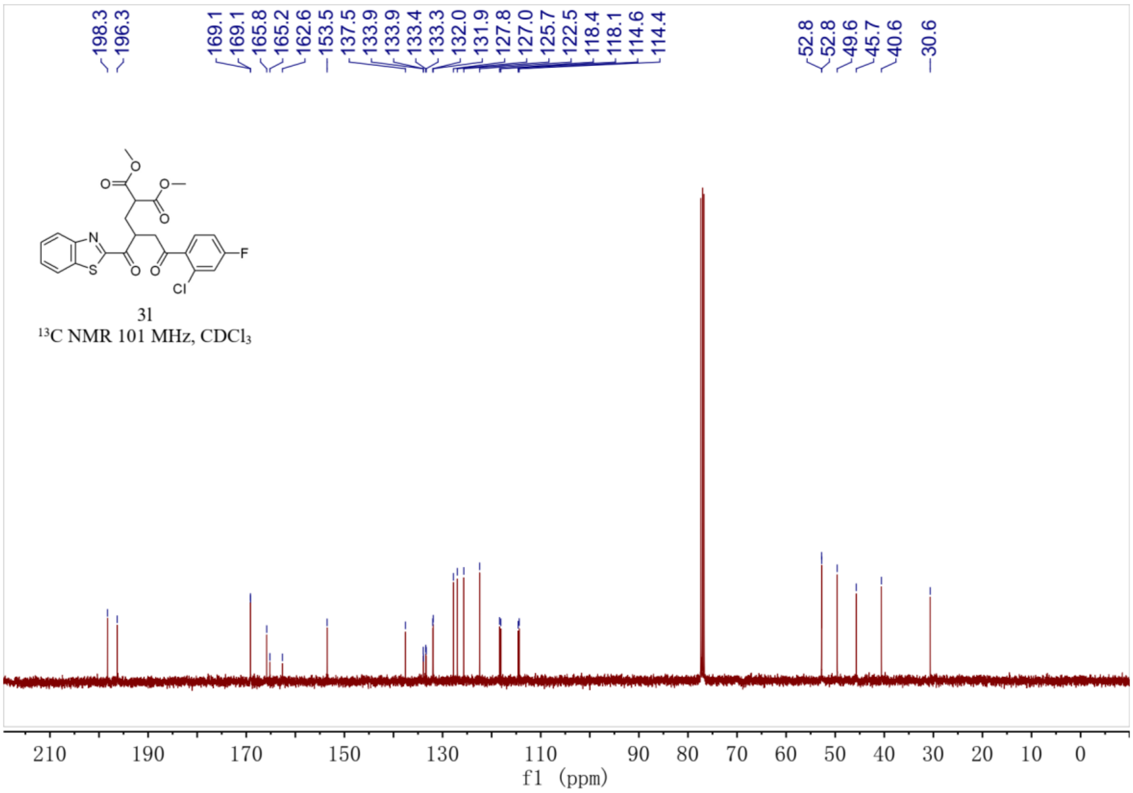
198.3 196.3 169.1 169.1 165.8 165.2 162.6 153.5 137.5 133.9 133.9 133.4 133.3 132.0 131.9 127.8 127.0 125.7 122.5 118.4 118.1 114.6 114.4 52.8 52.8 49.6 45.7 40.6 30.6
N
S
O
F
Cl
3l
13C NMR 101 MHz, CDCl3
210 190 170 150 130 110 90 80 70 60 50 40 30 20 10 0
f1 (ppm)

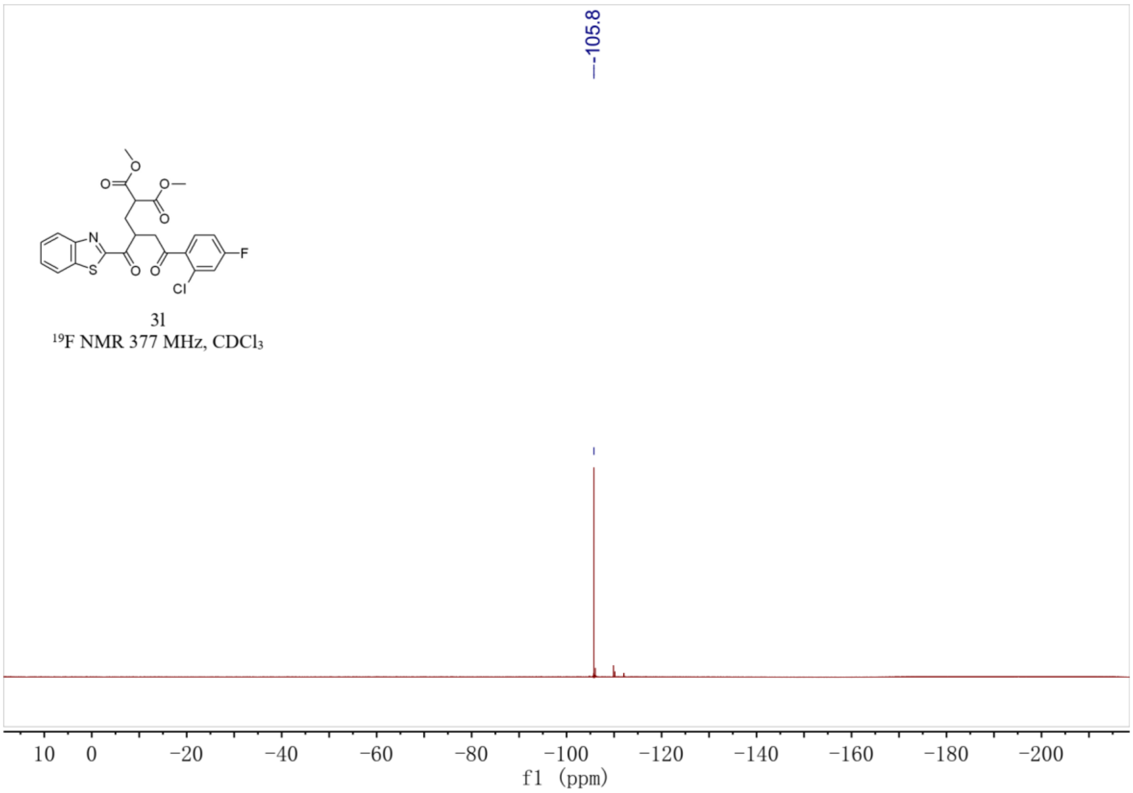
-105.8
3l
19F NMR 377 MHz, CDCl3
10
0
−20
−40
−60
−80
−100
−120
−140
−160
−180
−200
f1 (ppm)

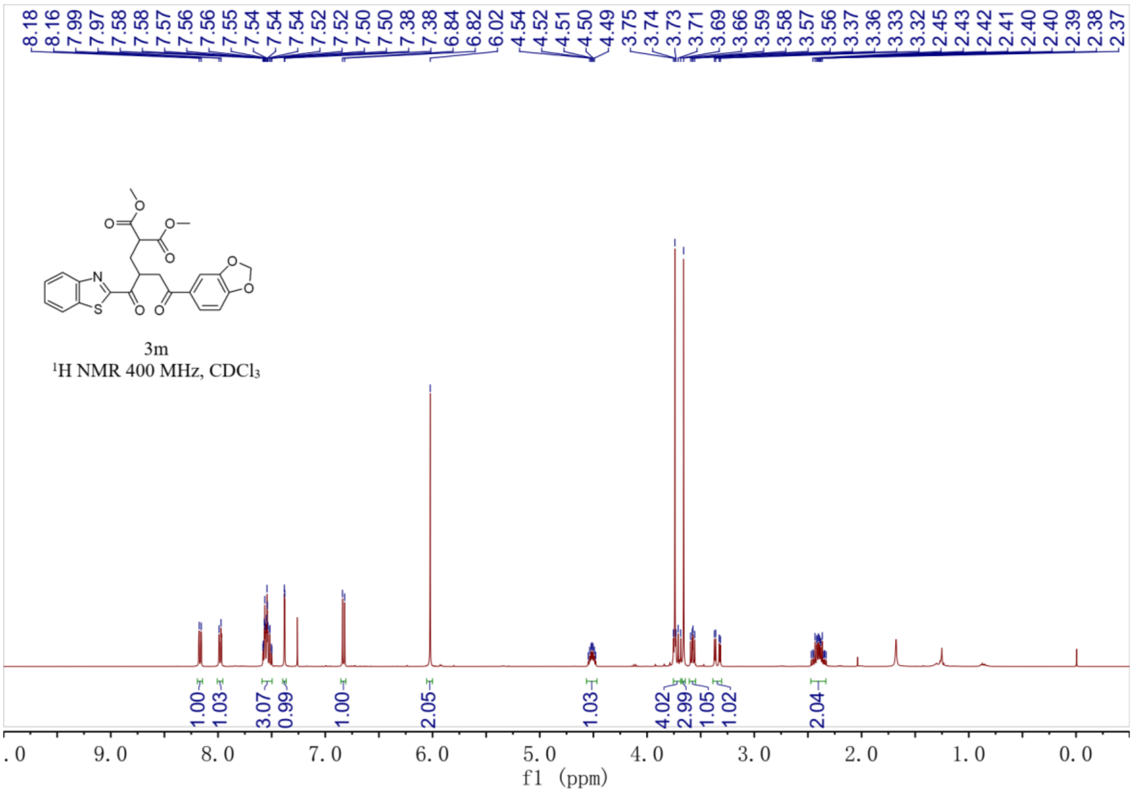
8.18
8.16
7.99
7.97
7.58
7.58
7.57
7.56
7.56
7.55
7.54
7.54
7.54
7.52
7.50
7.50
7.38
7.38
6.84
6.82
6.02
4.54
4.52
4.51
4.50
4.49
3.75
3.74
3.73
3.71
3.69
3.66
3.59
3.58
3.57
3.56
3.37
3.36
3.33
3.32
2.45
2.43
2.42
2.41
2.40
2.39
2.38
2.37
3m
¹H NMR 400 MHz, CDCl₃
1.00
1.03
3.07
0.99
1.00
2.05
1.03
4.02
2.99
1.05
1.02
2.04
9.0
8.0
7.0
6.0
5.0
4.0
3.0
2.0
1.0
0.0
f1 (ppm)

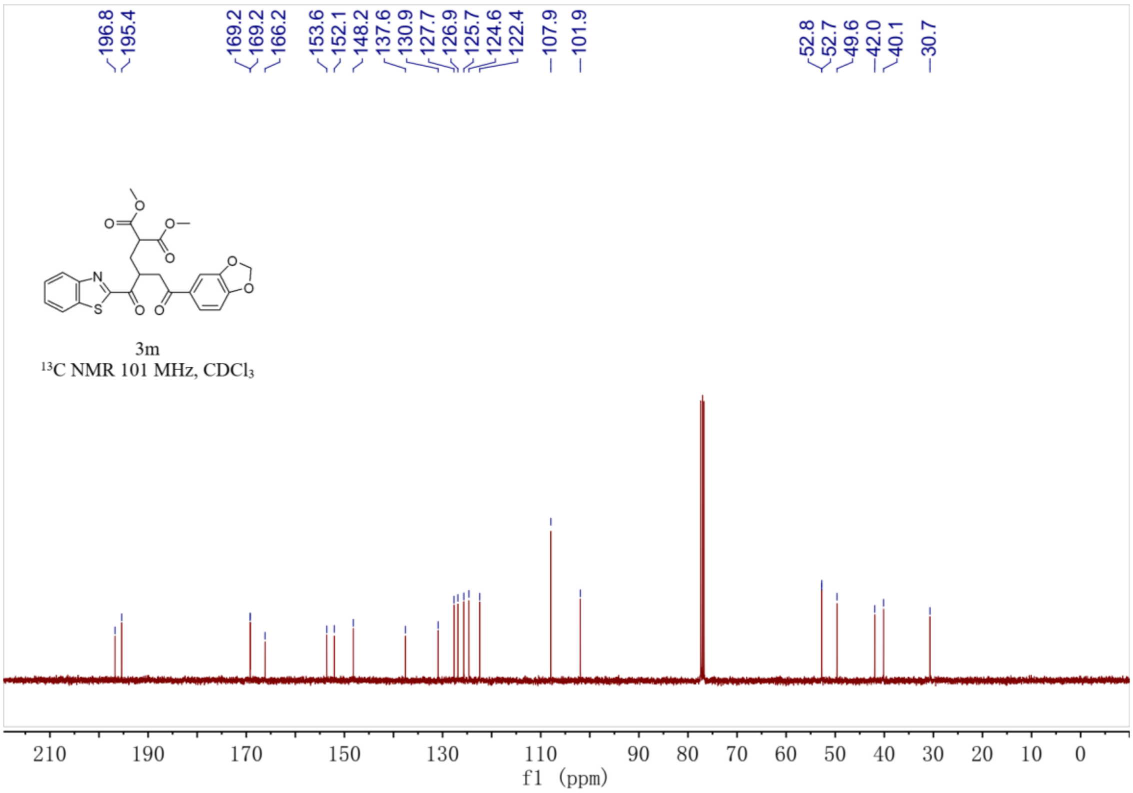
196.8
195.4
169.2
169.2
166.2
153.6
152.1
148.2
137.6
130.9
127.7
126.9
125.7
124.6
122.4
107.9
101.9
52.8
52.7
49.6
42.0
40.1
30.7
3m
¹³C NMR 101 MHz, CDCl₃
210
190
170
150
130
110
90
80
70
60
50
40
30
20
10
0
f1 (ppm)

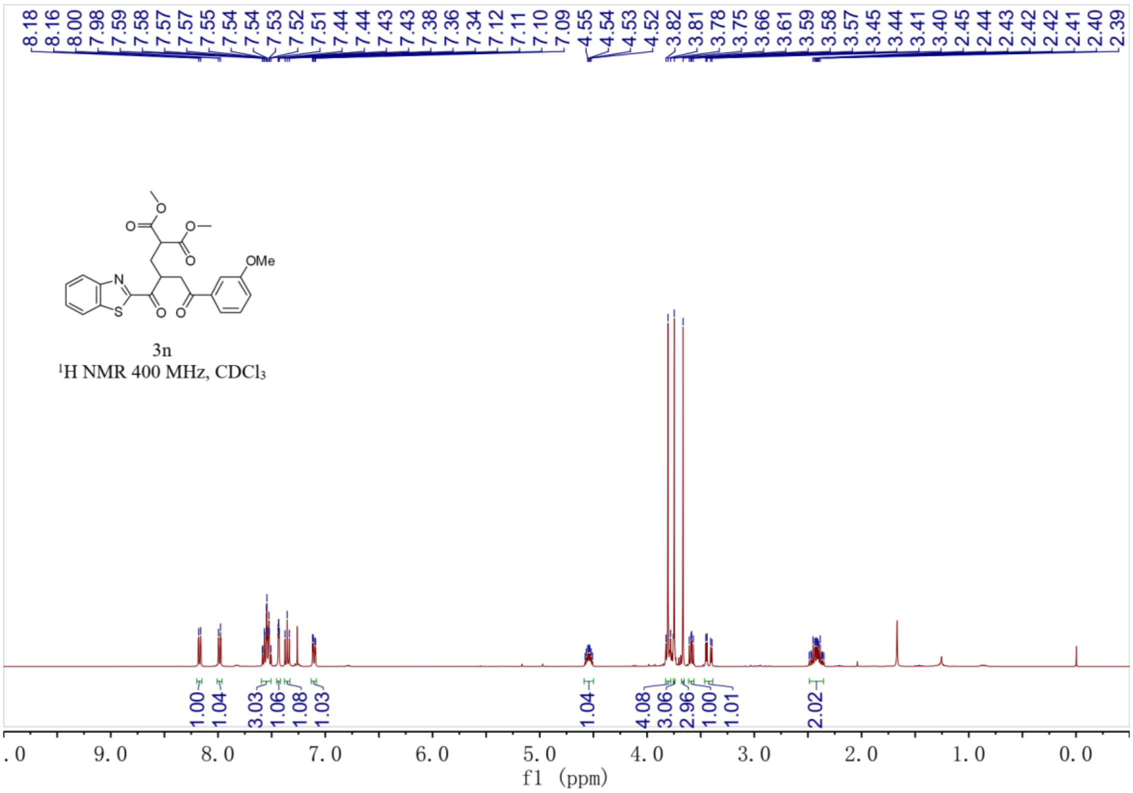
8.18 8.16 8.00 7.98 7.59 7.58 7.57 7.57 7.55 7.54 7.54 7.53 7.52 7.51 7.44 7.43 7.43 7.38 7.36 7.34 7.12 7.11 7.10 7.09 4.55 4.54 4.53 4.52 3.82 3.81 3.78 3.75 3.66 3.61 3.59 3.58 3.57 3.45 3.44 3.41 3.40 2.45 2.44 2.43 2.42 2.42 2.41 2.40 2.39
OMe
N
S
O
3n
¹H NMR 400 MHz, CDCl₃
1.00 1.04 3.03 1.06 1.08 1.03 1.04 4.08 3.06 2.96 1.00 1.01 2.02
9.0 8.0 7.0 6.0 5.0 4.0 3.0 2.0 1.0 0.0
f1 (ppm)

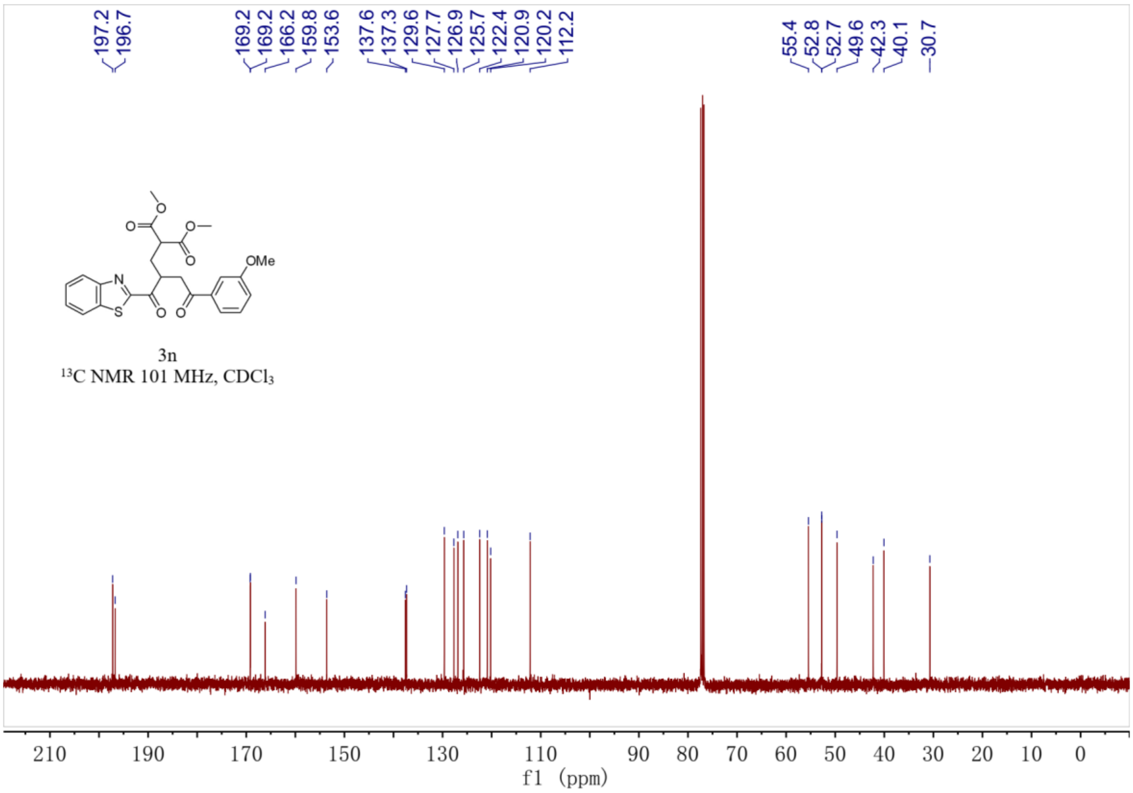
197.2 196.7 169.2 169.2 166.2 159.8 153.6 137.6 137.3 129.6 127.7 126.9 125.7 122.4 120.9 120.2 112.2 55.4 52.8 52.7 49.6 42.3 40.1 30.7
OMe
N
S
O
3n
¹³C NMR 101 MHz, CDCl₃
210 190 170 150 130 110 90 80 70 60 50 40 30 20 10 0
f1 (ppm)

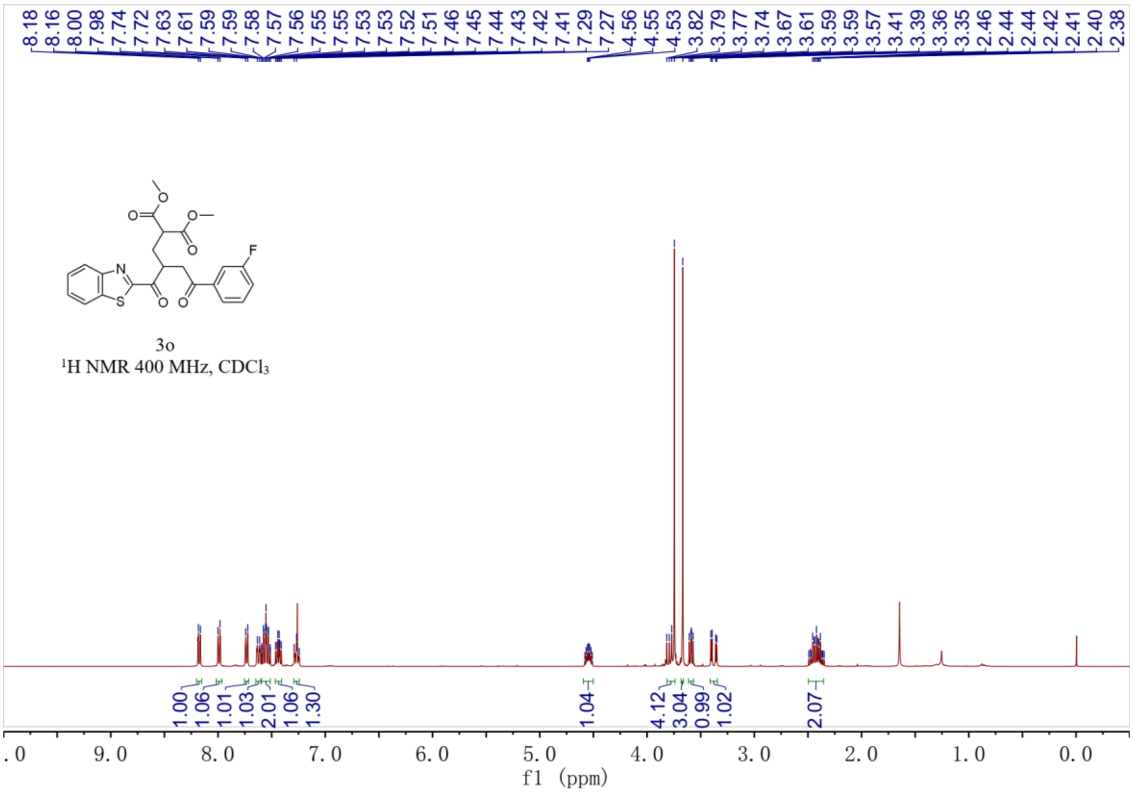
8.18
8.16
8.00
7.98
7.74
7.72
7.63
7.61
7.59
7.58
7.57
7.56
7.55
7.53
7.53
7.52
7.51
7.46
7.45
7.44
7.43
7.42
7.41
7.29
7.27
4.56
4.55
4.53
3.82
3.79
3.77
3.74
3.67
3.61
3.59
3.59
3.57
3.41
3.39
3.36
3.35
2.46
2.44
2.44
2.42
2.41
2.40
2.38
F
N
S
O
3o
^{1}H NMR 400 MHz, CDCl3
1.00
1.06
1.01
1.03
2.01
1.06
1.30
1.04
4.12
3.04
0.99
1.02
2.07
9.0
8.0
7.0
6.0
5.0
4.0
3.0
2.0
1.0
0.0
f1 (ppm)

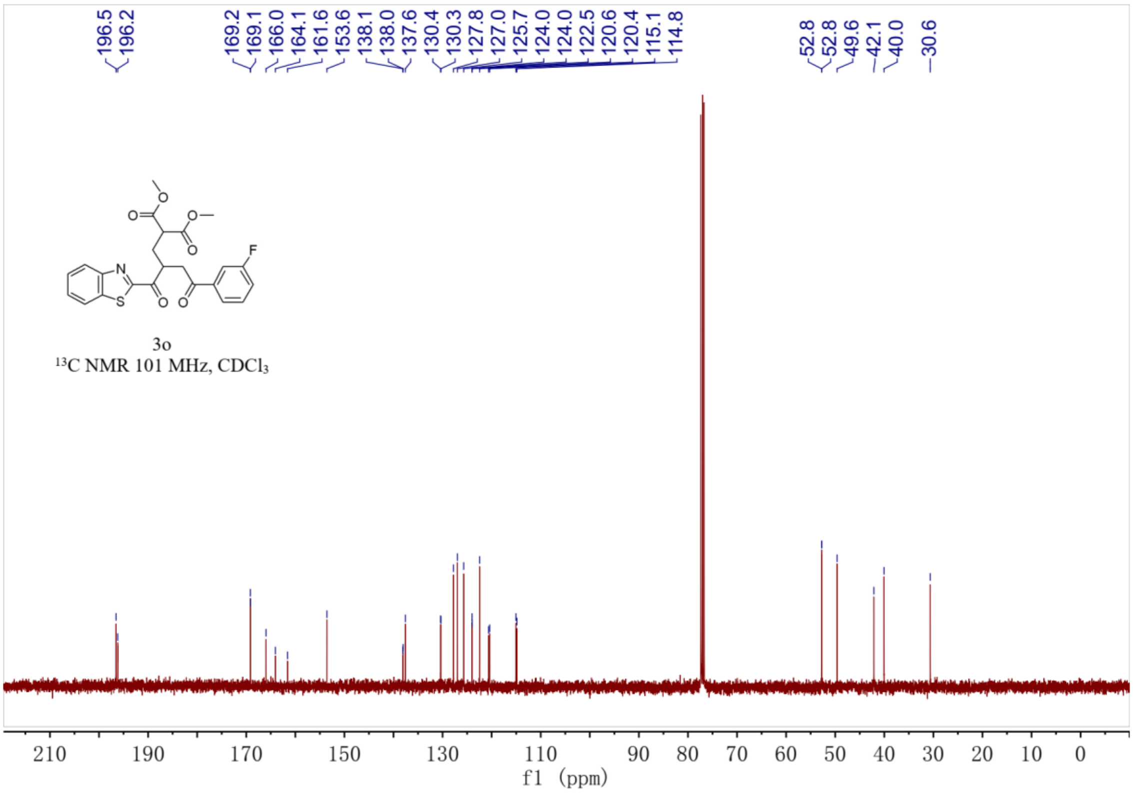
196.5
196.2
169.2
169.1
166.0
164.1
161.6
153.6
138.1
138.0
137.6
130.4
130.3
127.8
127.0
125.7
124.0
124.0
122.5
120.6
120.4
115.1
114.8
52.8
52.8
49.6
42.1
40.0
30.6
F
N
S
O
3o
^{13}C NMR 101 MHz, CDCl3
210
190
170
150
130
110
90
80
70
60
50
40
30
20
10
0
f1 (ppm)

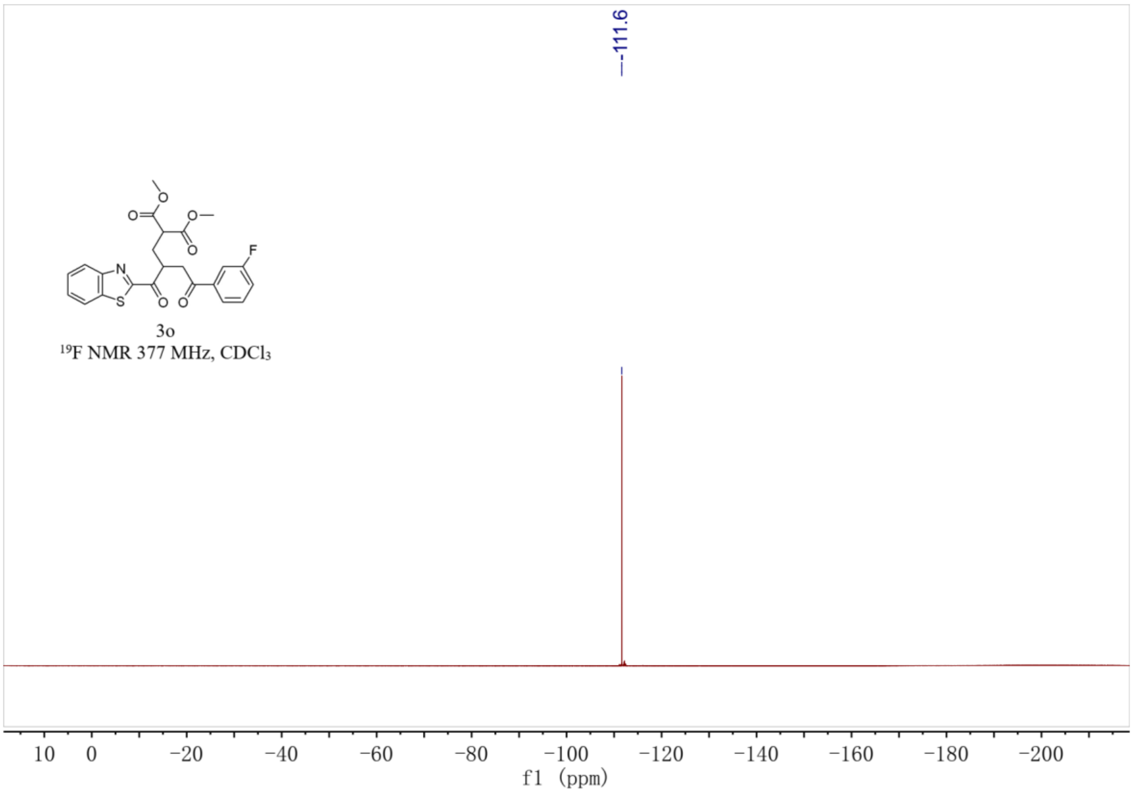

-111.6
F
N
S
O
3o
19F NMR 377 MHz, CDCl3
10 0 −20 −40 −60 −80 −100 −120 −140 −160 −180 −200
f1 (ppm)

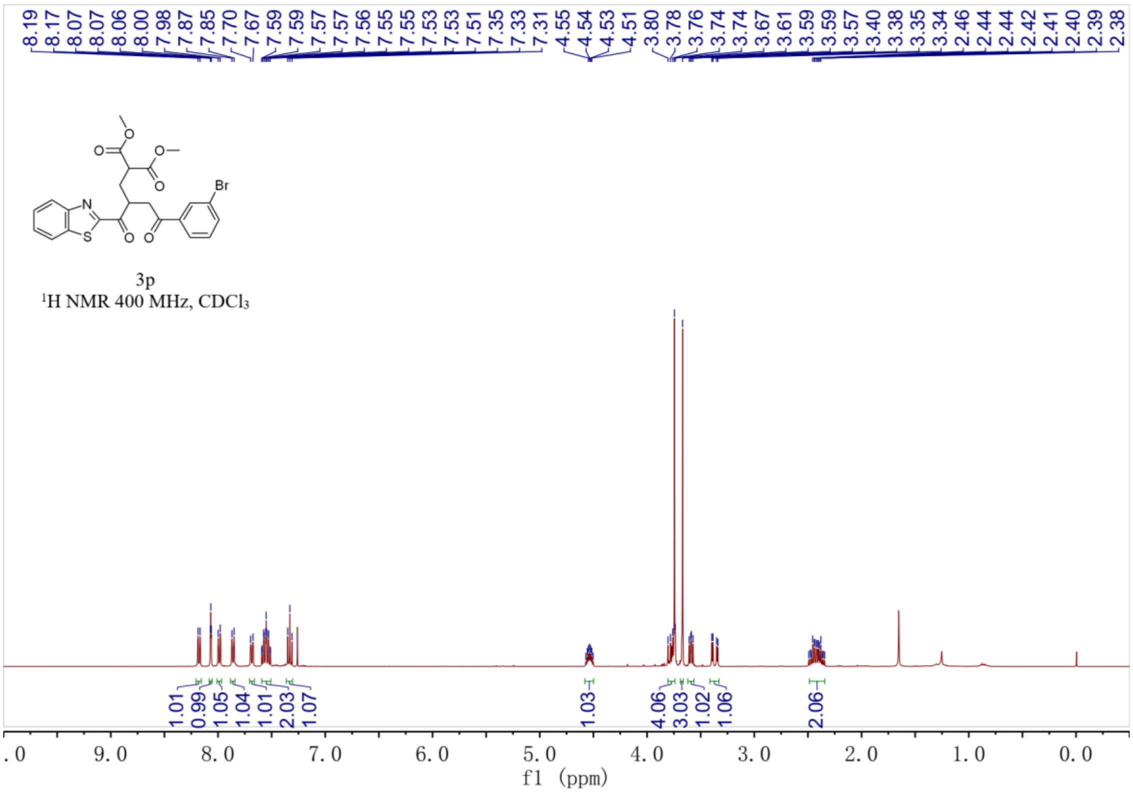
8.19 8.17 8.07 8.07 8.06 8.00 7.98 7.87 7.85 7.70 7.67 7.59 7.59 7.57 7.57 7.56 7.55 7.55 7.53 7.53 7.51 7.35 7.33 7.31 4.55 4.54 4.53 4.51 3.80 3.78 3.76 3.74 3.74 3.67 3.61 3.59 3.59 3.57 3.40 3.38 3.35 3.34 2.46 2.44 2.44 2.42 2.41 2.40 2.39 2.38
Br
N
S
O
3p
1H NMR 400 MHz, CDCl3
1.01 0.99 1.05 1.04 1.01 2.03 1.07 1.03 4.06 3.03 1.02 1.06 2.06
9.0 8.0 7.0 6.0 5.0 4.0 3.0 2.0 1.0 0.0
f1 (ppm)

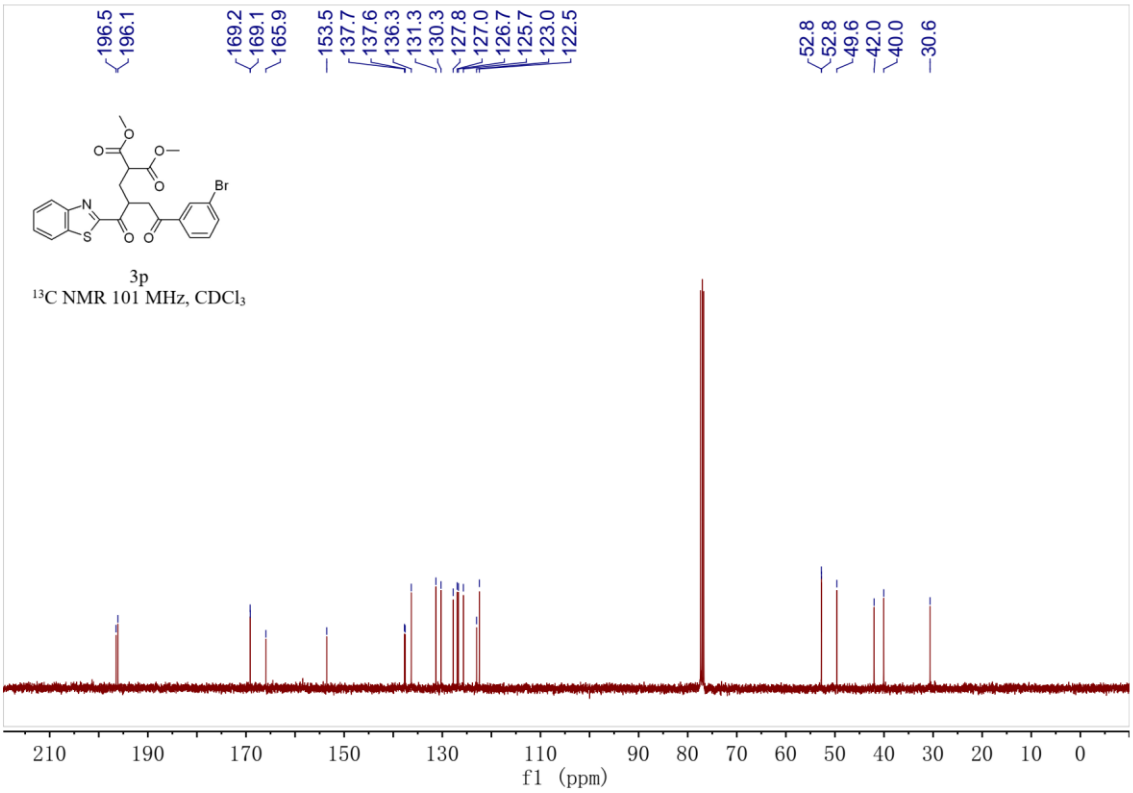
196.5 196.1 169.2 169.1 165.9 153.5 137.7 137.6 136.3 131.3 130.3 127.8 127.0 126.7 125.7 123.0 122.5 52.8 52.8 49.6 42.0 40.0 30.6
Br
N
S
O
3p
13C NMR 101 MHz, CDCl3
210 190 170 150 130 110 90 80 70 60 50 40 30 20 10 0
f1 (ppm)

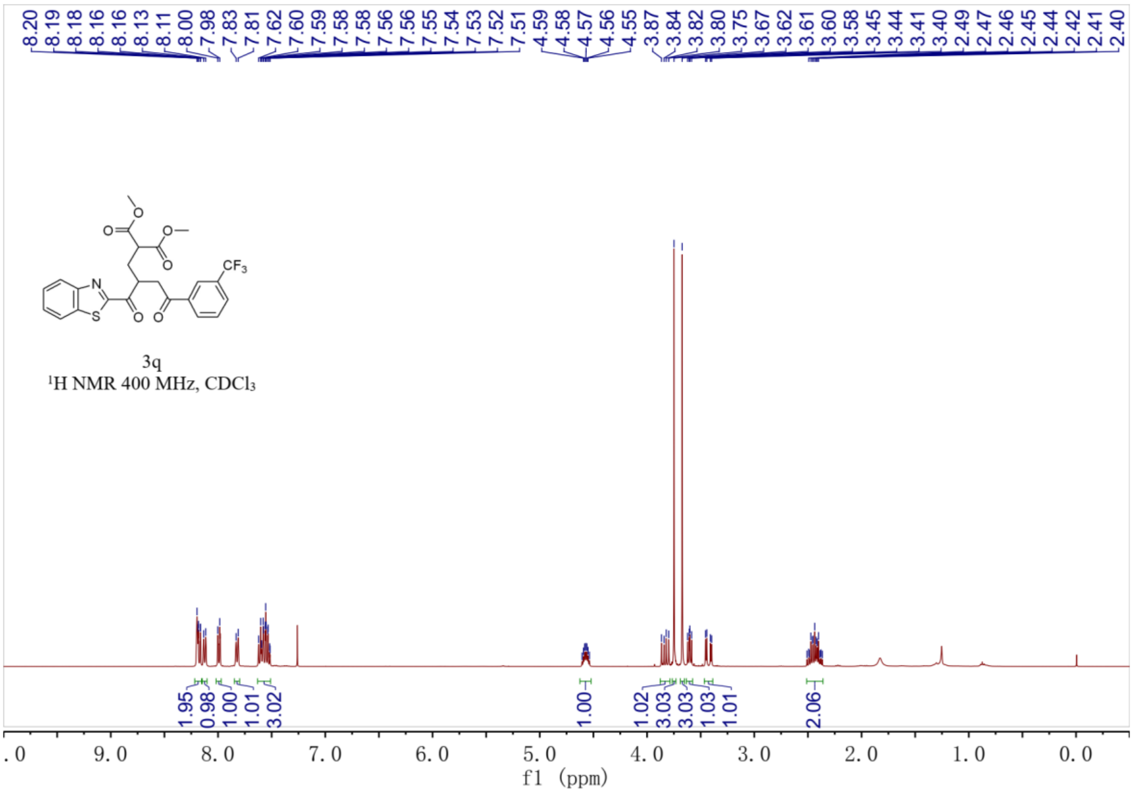
8.20
8.19
8.18
8.16
8.16
8.13
8.11
8.00
7.98
7.83
7.81
7.62
7.60
7.59
7.58
7.58
7.56
7.56
7.55
7.54
7.53
7.52
7.51
4.59
4.58
4.57
4.56
4.55
3.87
3.84
3.82
3.80
3.75
3.67
3.62
3.61
3.60
3.58
3.45
3.44
3.41
3.40
2.49
2.47
2.46
2.45
2.44
2.42
2.41
2.40
CF3
N
S
O
3q
1H NMR 400 MHz, CDCl3
1.95
0.98
1.00
1.01
3.02
1.00
1.02
3.03
3.03
1.03
1.01
2.06
9.0
8.0
7.0
6.0
5.0
4.0
3.0
2.0
1.0
0.0
f1 (ppm)

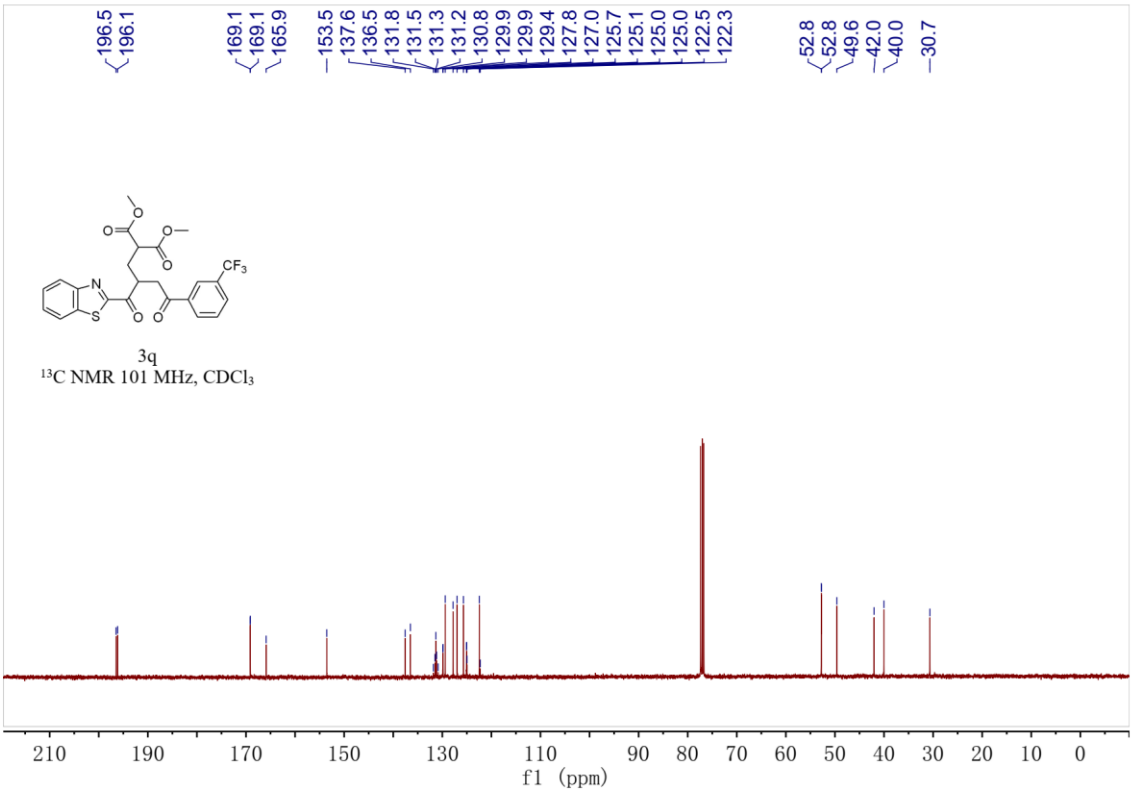
196.5
196.1
169.1
169.1
165.9
153.5
137.6
136.5
131.8
131.5
131.3
131.2
130.8
129.9
129.9
129.4
127.8
127.0
125.7
125.1
125.0
125.0
122.5
122.3
52.8
52.8
49.6
42.0
40.0
30.7
CF3
N
S
O
3q
13C NMR 101 MHz, CDCl3
210
190
170
150
130
110
90
80
70
60
50
40
30
20
10
0
f1 (ppm)

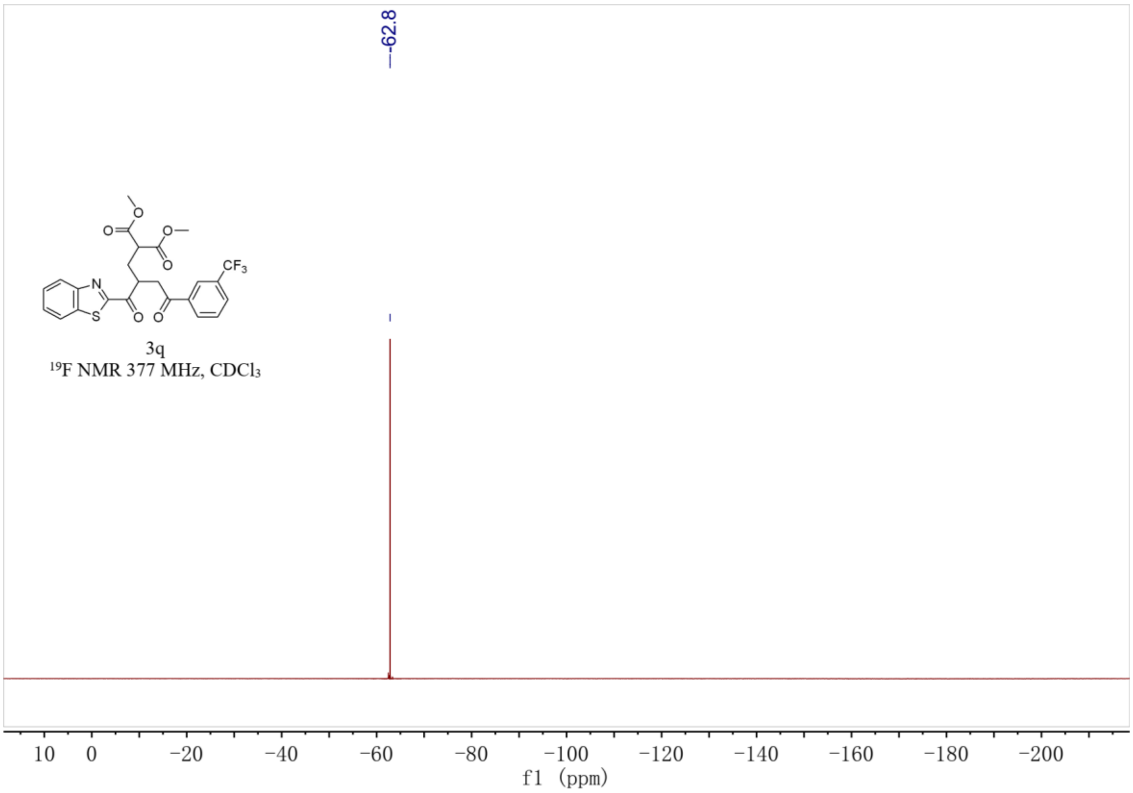
−62.8
CF3
N
S
O
3q
19F NMR 377 MHz, CDCl3
10 0 −20 −40 −60 −80 −100 −120 −140 −160 −180 −200
f1 (ppm)

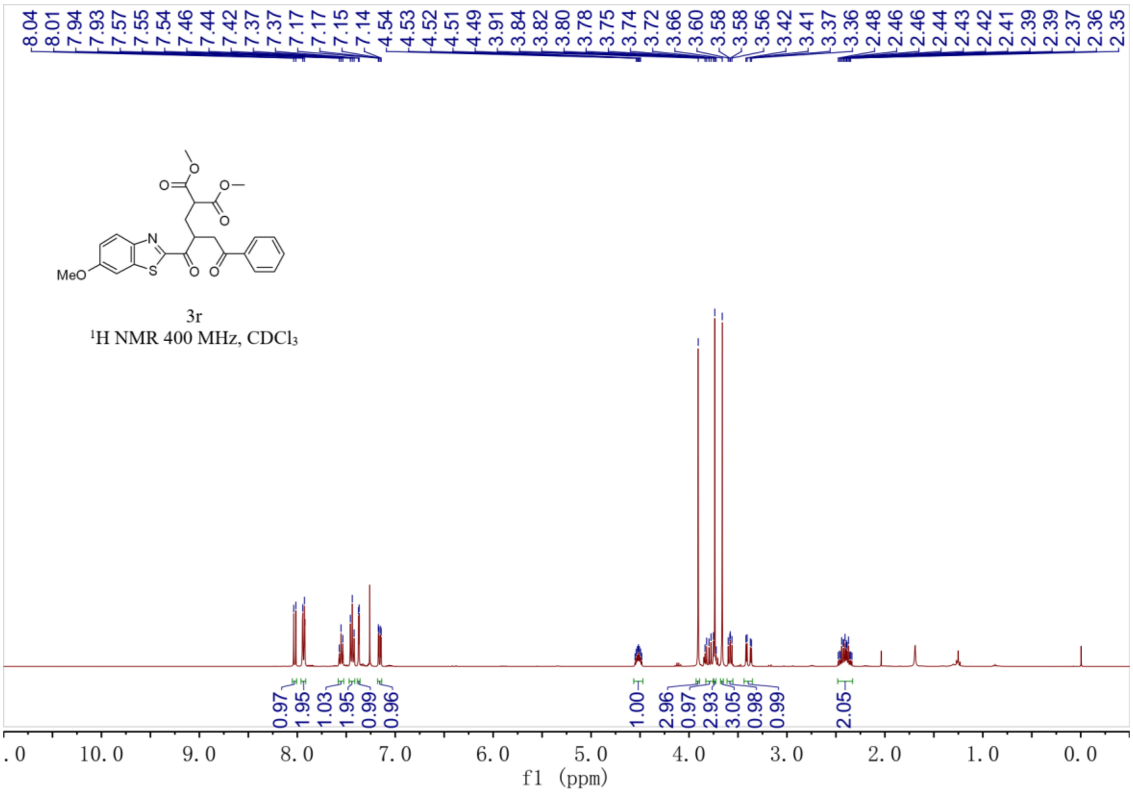
8.04 8.01 7.94 7.93 7.57 7.55 7.54 7.46 7.44 7.42 7.37 7.37 7.17 7.17 7.15 7.14 4.54 4.53 4.52 4.51 4.49 3.91 3.84 3.82 3.80 3.78 3.75 3.74 3.72 3.66 3.60 3.58 3.58 3.56 3.42 3.41 3.37 3.36 2.48 2.46 2.44 2.43 2.42 2.41 2.39 2.39 2.37 2.36 2.35
MeO
N
S
O
3r
1H NMR 400 MHz, CDCl3
0.97 1.95 1.03 1.95 0.99 0.96 1.00 2.96 0.97 2.93 3.05 0.98 0.99 2.05
.0 10.0 9.0 8.0 7.0 6.0 5.0 4.0 3.0 2.0 1.0 0.0
f1 (ppm)

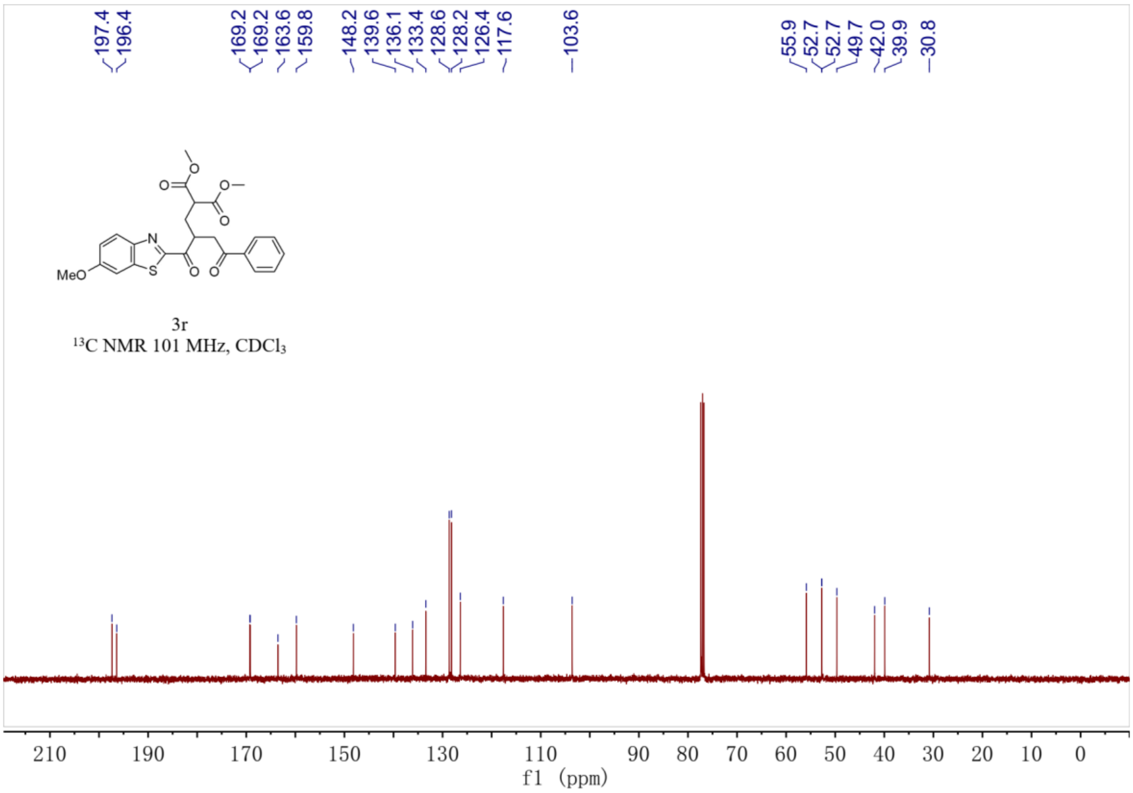
197.4 196.4 169.2 169.2 163.6 159.8 148.2 139.6 136.1 133.4 128.6 128.2 126.4 117.6 103.6 55.9 52.7 52.7 49.7 42.0 39.9 30.8
MeO
N
S
O
3r
13C NMR 101 MHz, CDCl3
210 190 170 150 130 110 90 80 70 60 50 40 30 20 10 0
f1 (ppm)

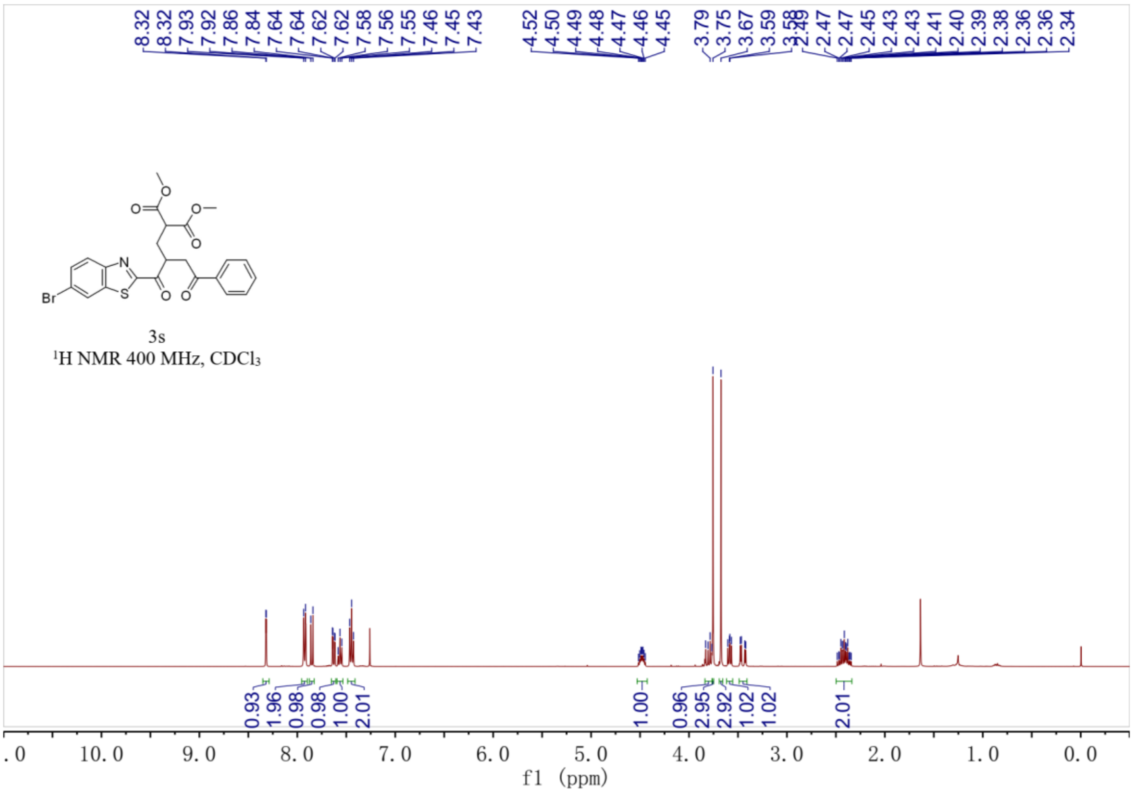
8.32
8.32
7.93
7.92
7.86
7.84
7.64
7.64
7.62
7.62
7.58
7.56
7.55
7.46
7.45
7.43
4.52
4.50
4.49
4.48
4.47
4.46
4.45
3.79
3.75
3.67
3.59
3.58
3.49
2.47
2.47
2.45
2.43
2.43
2.41
2.40
2.39
2.38
2.36
2.36
2.34
Br
N
S
O
3s
^{1}H NMR 400 MHz, CDCl3
0.93
1.96
0.98
0.98
1.00
2.01
1.00
0.96
2.95
2.92
1.02
1.02
2.01
f1 (ppm)

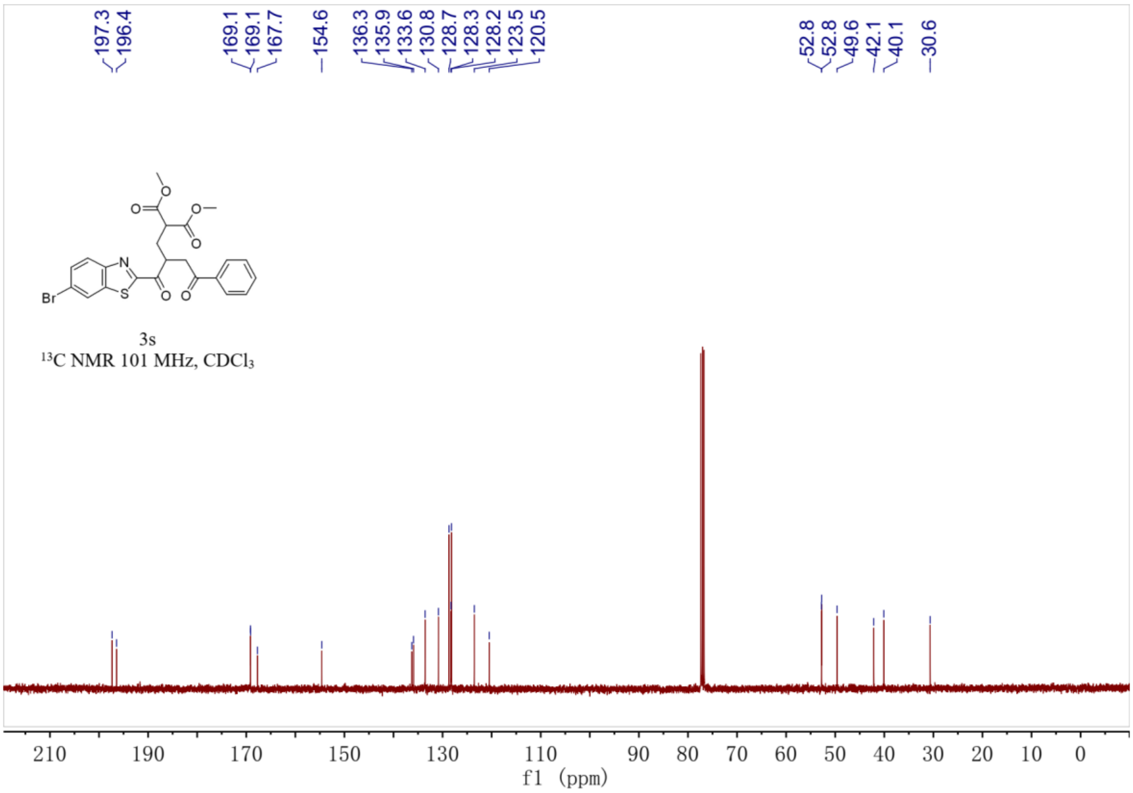
197.3
196.4
169.1
169.1
167.7
154.6
136.3
135.9
133.6
130.8
128.7
128.3
128.2
123.5
120.5
52.8
52.8
49.6
42.1
40.1
30.6
Br
N
S
O
3s
^{13}C NMR 101 MHz, CDCl3
f1 (ppm)

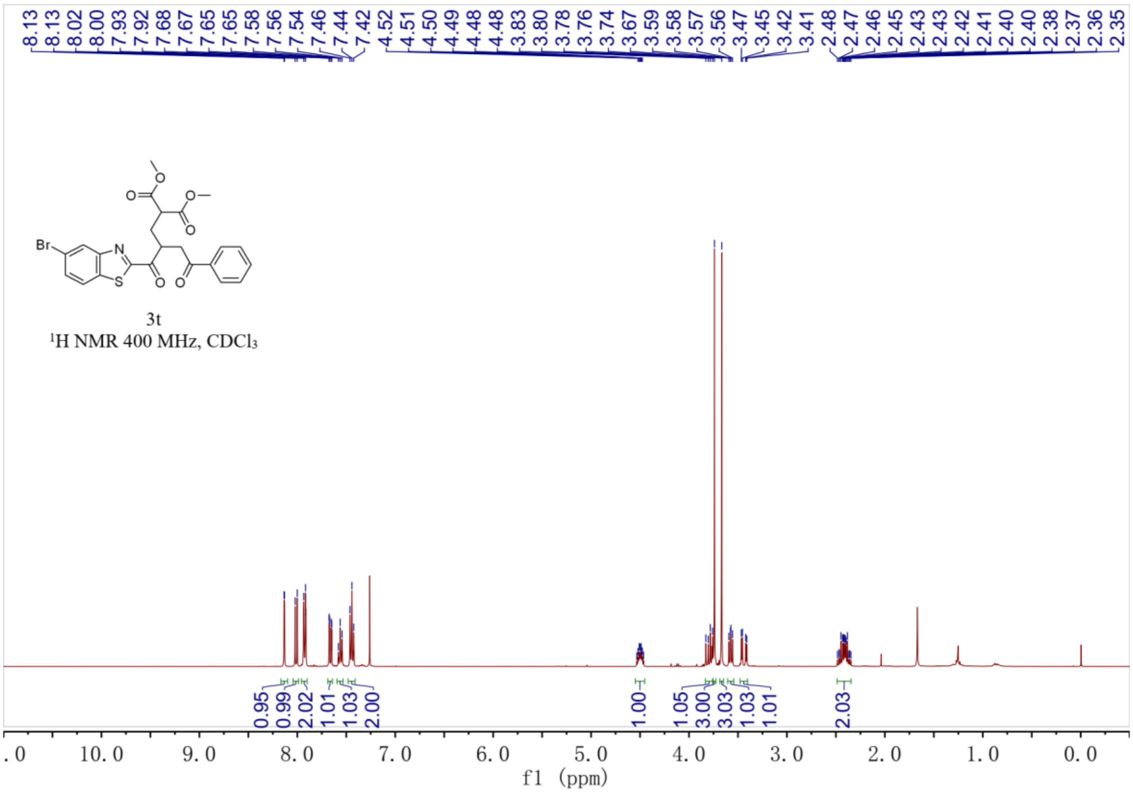

3t
1H NMR 400 MHz, CDCl3
f1 (ppm)

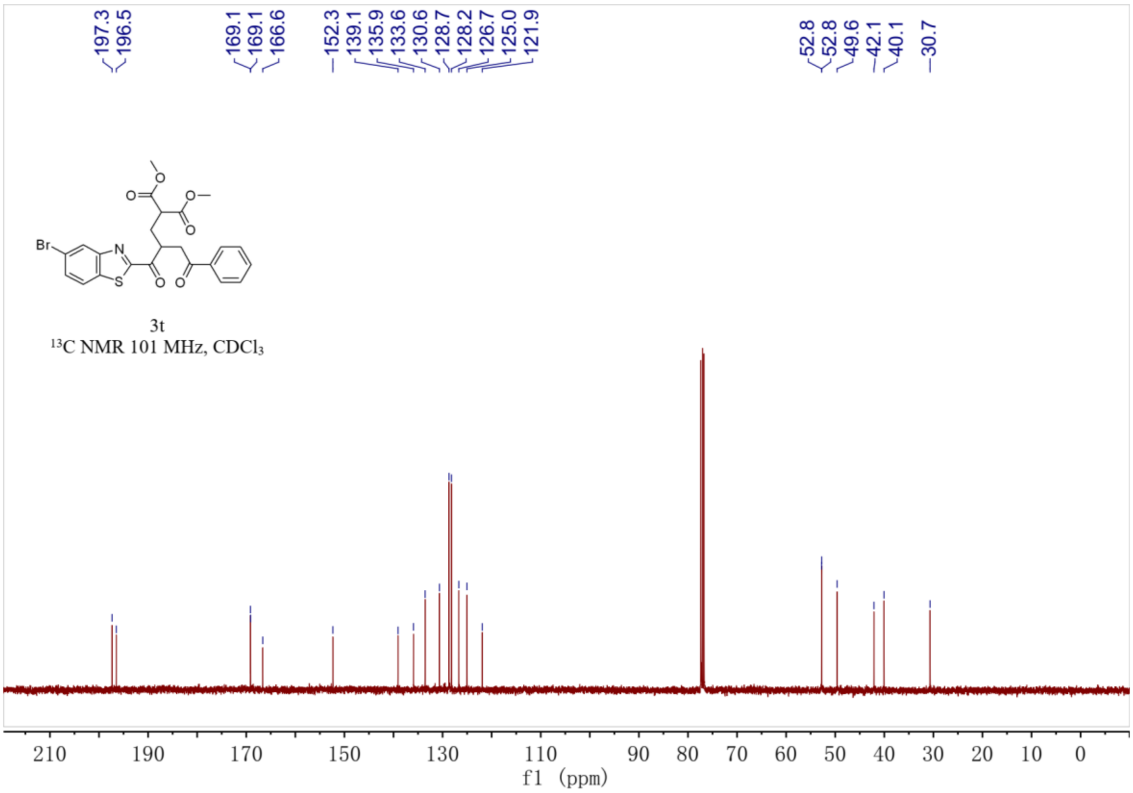

3t
13C NMR 101 MHz, CDCl3
f1 (ppm)

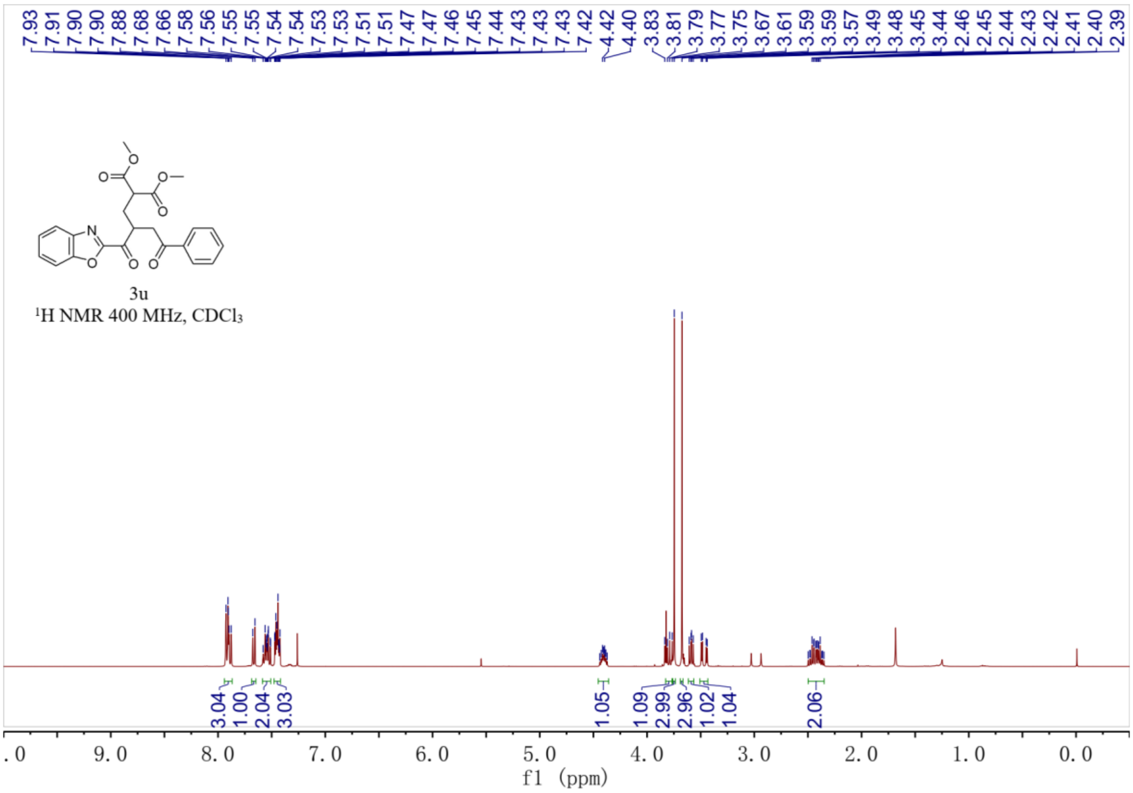
7.93
7.91
7.90
7.90
7.88
7.68
7.66
7.58
7.56
7.55
7.55
7.54
7.54
7.53
7.51
7.51
7.47
7.47
7.46
7.45
7.44
7.43
7.43
7.43
7.42
4.42
4.40
3.83
3.81
3.79
3.77
3.75
3.67
3.61
3.59
3.59
3.57
3.49
3.48
3.45
3.44
2.46
2.45
2.44
2.43
2.42
2.41
2.40
2.39
3u
¹H NMR 400 MHz, CDCl₃
3.04
1.00
2.04
3.03
1.05
1.09
2.99
2.96
1.02
1.04
2.06
f1 (ppm)

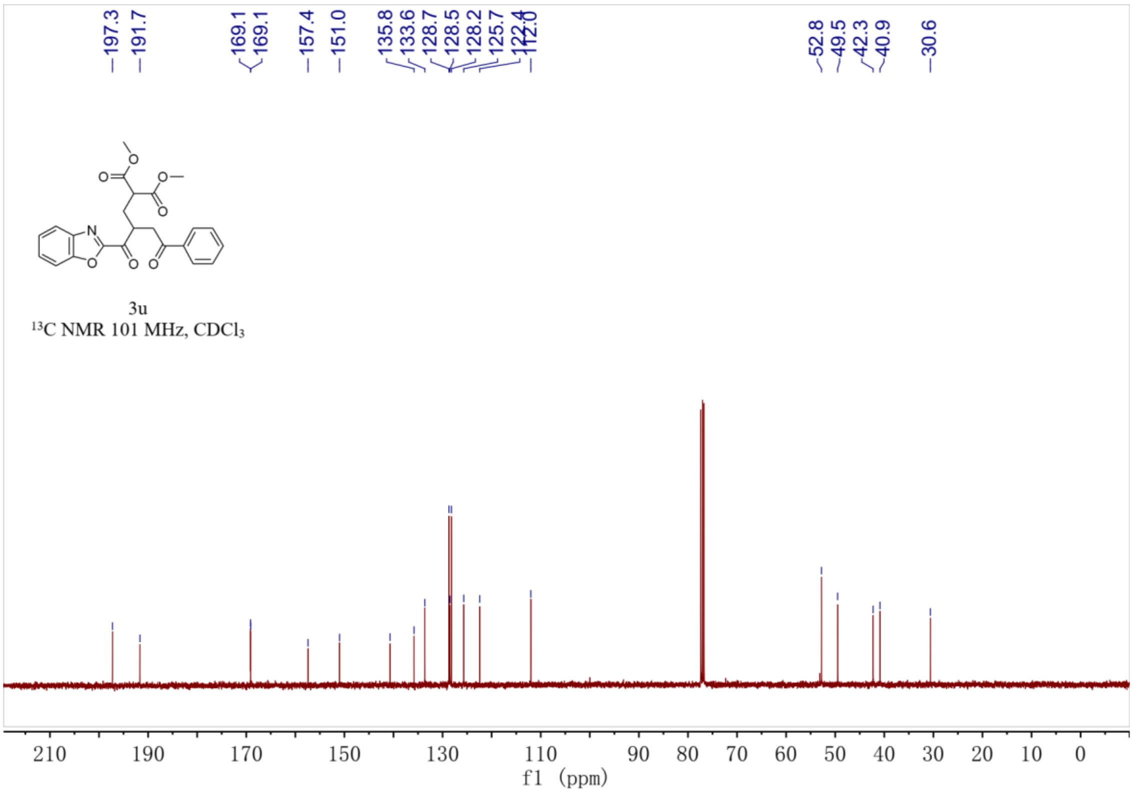
197.3
191.7
169.1
169.1
157.4
151.0
135.8
133.6
128.7
128.5
128.2
125.7
122.4
112.0
52.8
49.5
42.3
40.9
30.6
3u
¹³C NMR 101 MHz, CDCl₃
f1 (ppm)

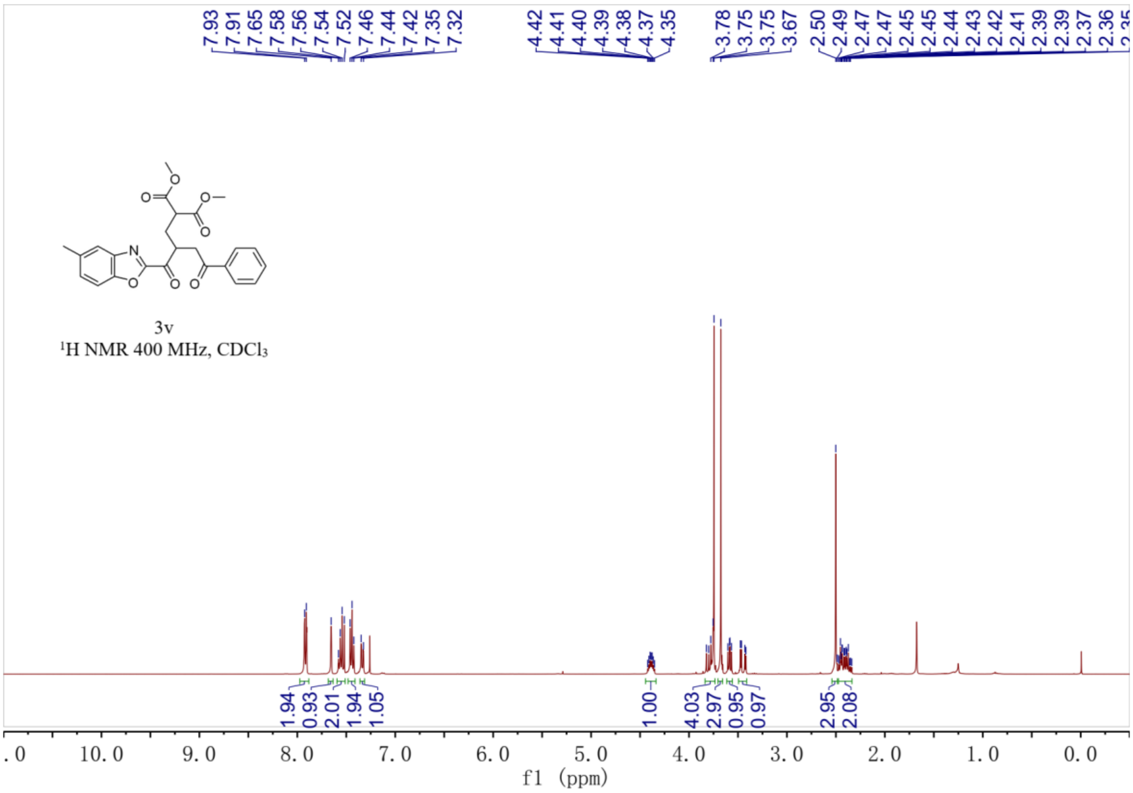
7.93 7.91 7.65 7.58 7.56 7.54 7.52 7.46 7.44 7.42 7.35 7.32
4.42 4.41 4.40 4.39 4.38 4.37 4.35
3.78 3.75 3.75 3.67
2.50 2.49 2.47 2.47 2.45 2.45 2.44 2.43 2.42 2.41 2.39 2.39 2.37 2.36
3v
1H NMR 400 MHz, CDCl3
1.94 0.93 2.01 1.94 1.05 1.00 4.03 2.97 0.95 0.97 2.95 2.08
10.0 9.0 8.0 7.0 6.0 5.0 4.0 3.0 2.0 1.0 0.0
f1 (ppm)

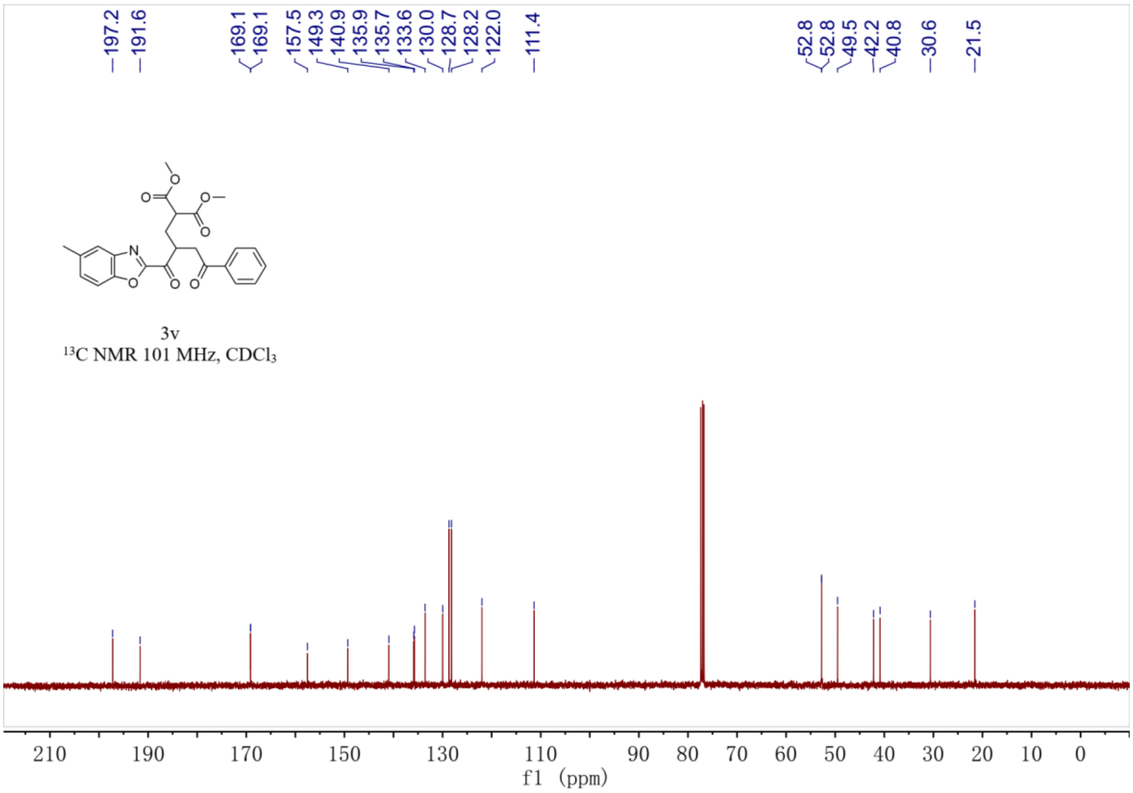
197.2 191.6 169.1 169.1 157.5 149.3 140.9 135.9 135.7 133.6 130.0 128.7 128.2 122.0 111.4
52.8 52.8 49.5 42.2 40.8 30.6 21.5
3v
13C NMR 101 MHz, CDCl3
210 190 170 150 130 110 90 80 70 60 50 40 30 20 10 0
f1 (ppm)

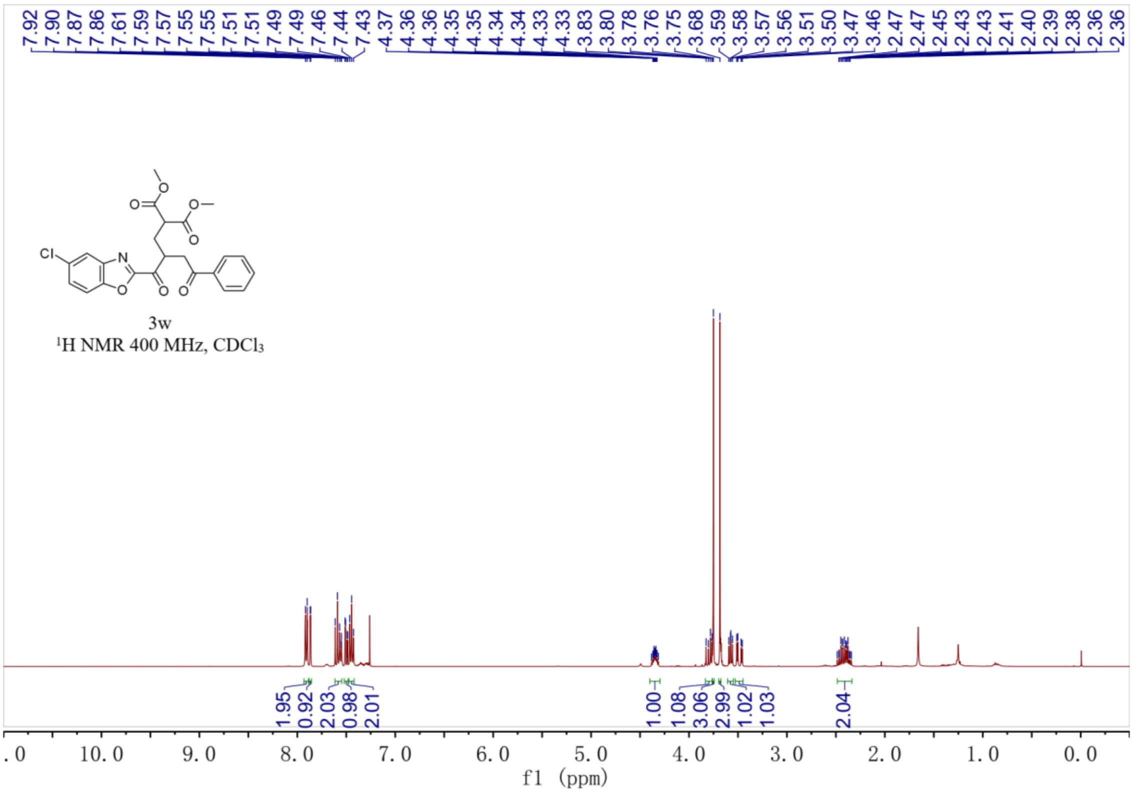
7.92 7.90 7.87 7.86 7.61 7.59 7.57 7.55 7.55 7.51 7.51 7.49 7.49 7.46 7.44 7.43 4.37 4.36 4.36 4.35 4.34 4.34 4.33 4.33 3.83 3.80 3.78 3.76 3.75 3.68 3.59 3.58 3.57 3.56 3.51 3.50 3.47 3.46 2.47 2.47 2.45 2.43 2.43 2.41 2.40 2.39 2.38 2.36 2.36
Cl
N
O
3w
^{1}H NMR 400 MHz, CDCl$_3$
1.95 0.92 2.03 0.98 2.01
1.00 1.08 3.06 2.99 1.02 1.03
2.04
10.0 9.0 8.0 7.0 6.0 5.0 4.0 3.0 2.0 1.0 0.0
f1 (ppm)

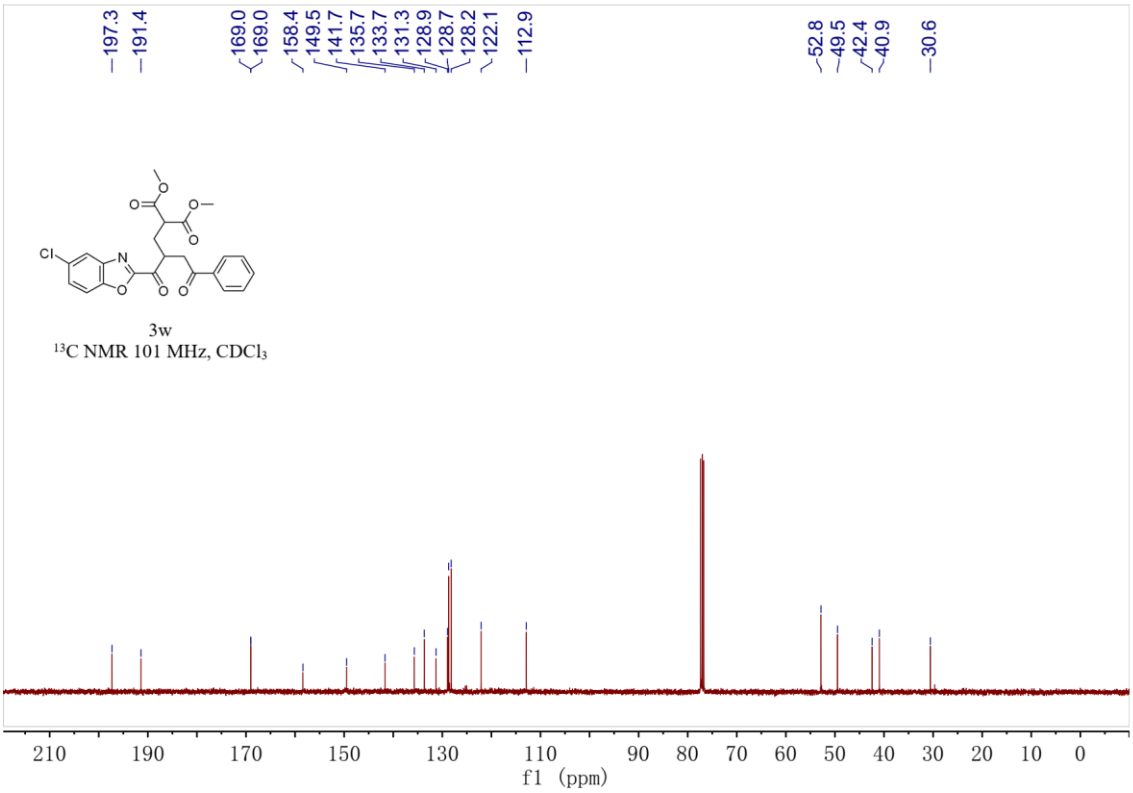
197.3 191.4 169.0 169.0 158.4 149.5 141.7 135.7 133.7 131.3 128.9 128.7 128.2 122.1 112.9
52.8 49.5 42.4 40.9 30.6
Cl
N
O
3w
^{13}C NMR 101 MHz, CDCl$_3$
210 190 170 150 130 110 90 80 70 60 50 40 30 20 10 0
f1 (ppm)

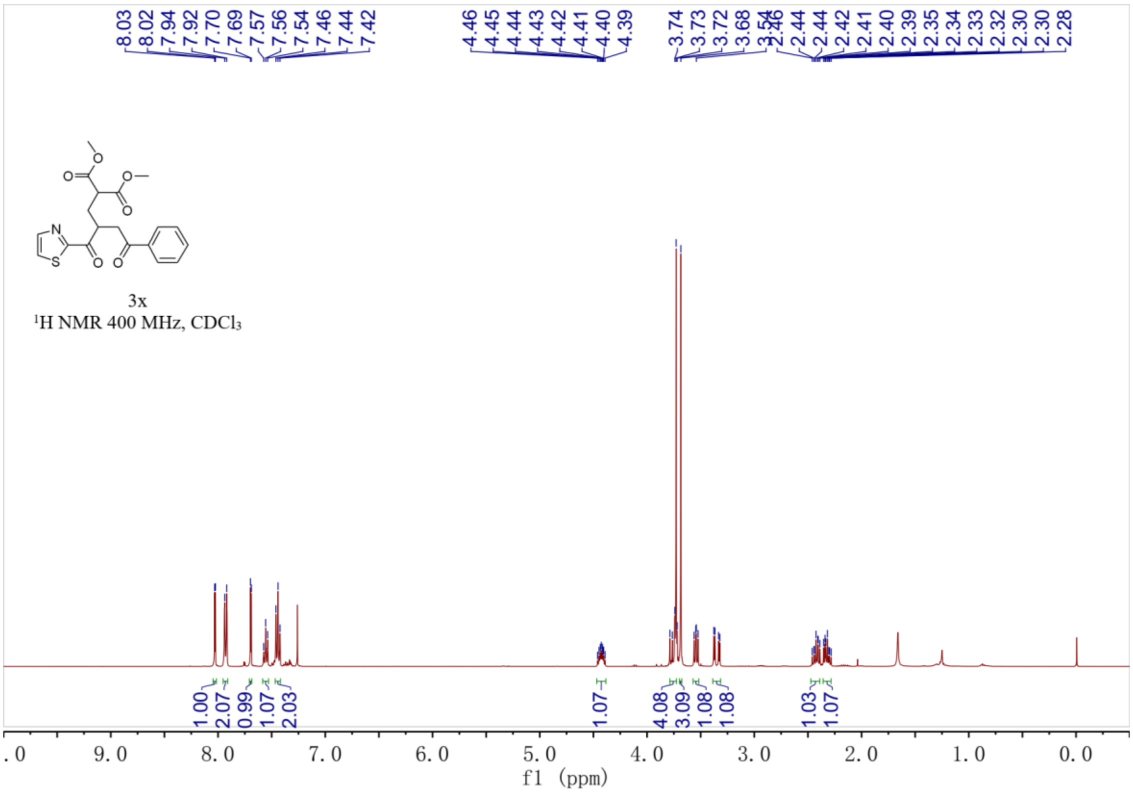
8.03 8.02 7.94 7.92 7.70 7.69 7.57 7.56 7.54 7.46 7.44 7.42
4.46 4.45 4.44 4.43 4.42 4.41 4.40 4.39
3.74 3.73 3.72 3.68 3.54 3.46 2.44 2.44 2.42 2.41 2.40 2.39 2.35 2.34 2.33 2.32 2.30 2.30 2.28
3x
1H NMR 400 MHz, CDCl3
1.00 2.07 0.99 1.07 2.03 1.07 4.08 3.09 1.08 1.08 1.03 1.07
.0 9.0 8.0 7.0 6.0 5.0 4.0 3.0 2.0 1.0 0.0
f1 (ppm)

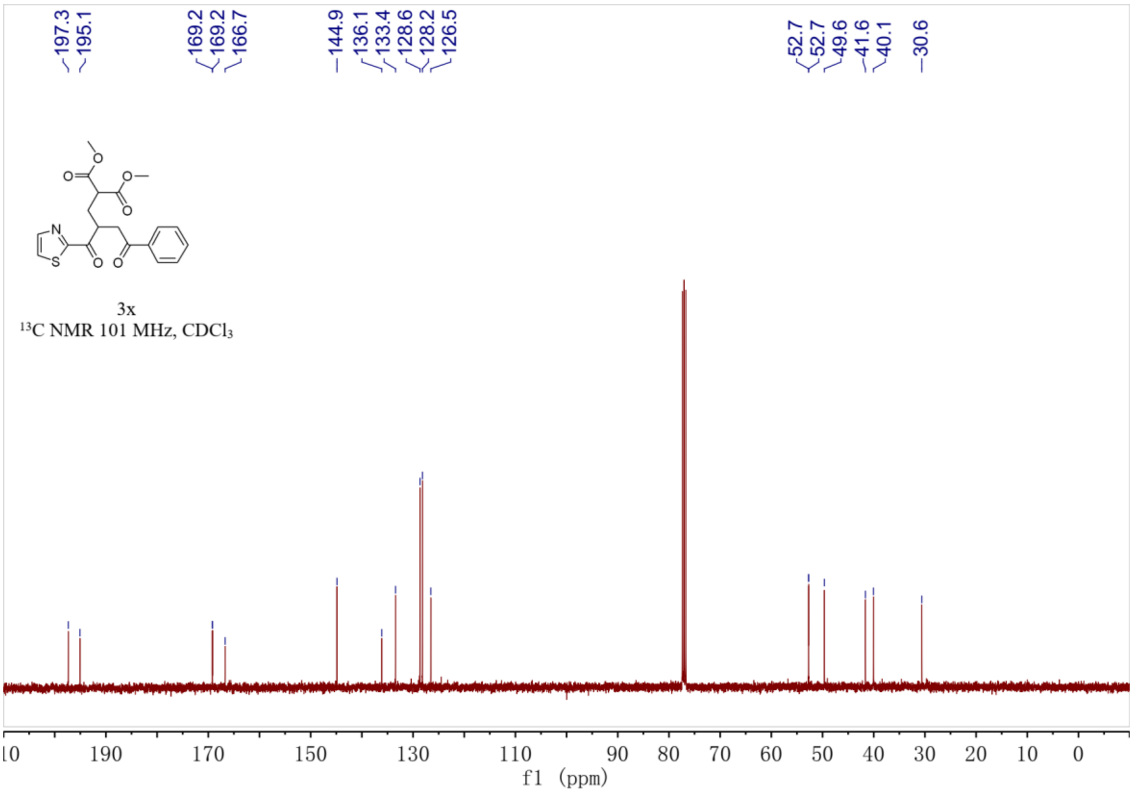
197.3 195.1 169.2 169.2 166.7 144.9 136.1 133.4 128.6 128.2 126.5 52.7 52.7 49.6 41.6 40.1 30.6
3x
13C NMR 101 MHz, CDCl3
10 190 170 150 130 110 90 80 70 60 50 40 30 20 10 0
f1 (ppm)

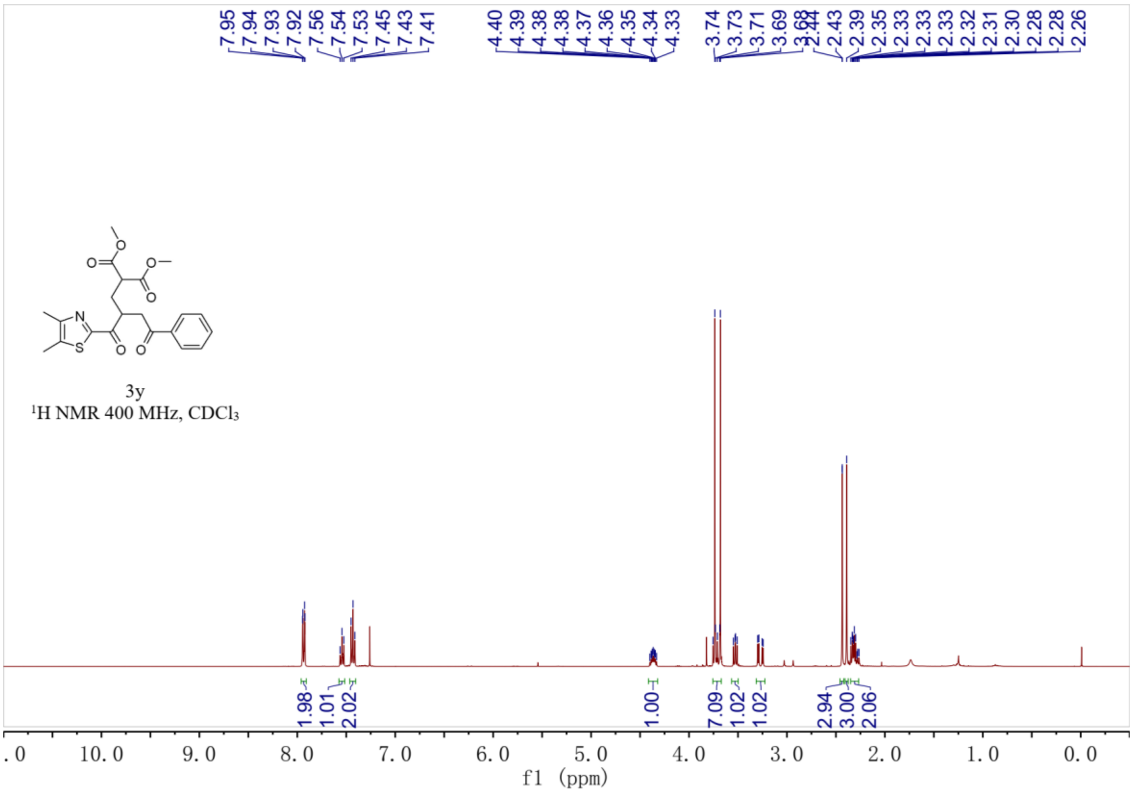
7.95 7.94 7.93 7.92 7.56 7.54 7.53 7.45 7.43 7.41
4.40 4.39 4.38 4.38 4.37 4.36 4.35 4.34 4.33
3.74 3.73 3.71 3.69 3.68 2.44 2.43 2.39 2.35 2.33 2.33 2.33 2.32 2.31 2.30 2.28 2.28 2.26
3y
^{1}H NMR 400 MHz, $CDCl_3$
1.98 1.01 2.02 1.00 7.09 1.02 1.02 2.94 3.00 2.06
.0 10.0 9.0 8.0 7.0 6.0 5.0 4.0 3.0 2.0 1.0 0.0
f1 (ppm)

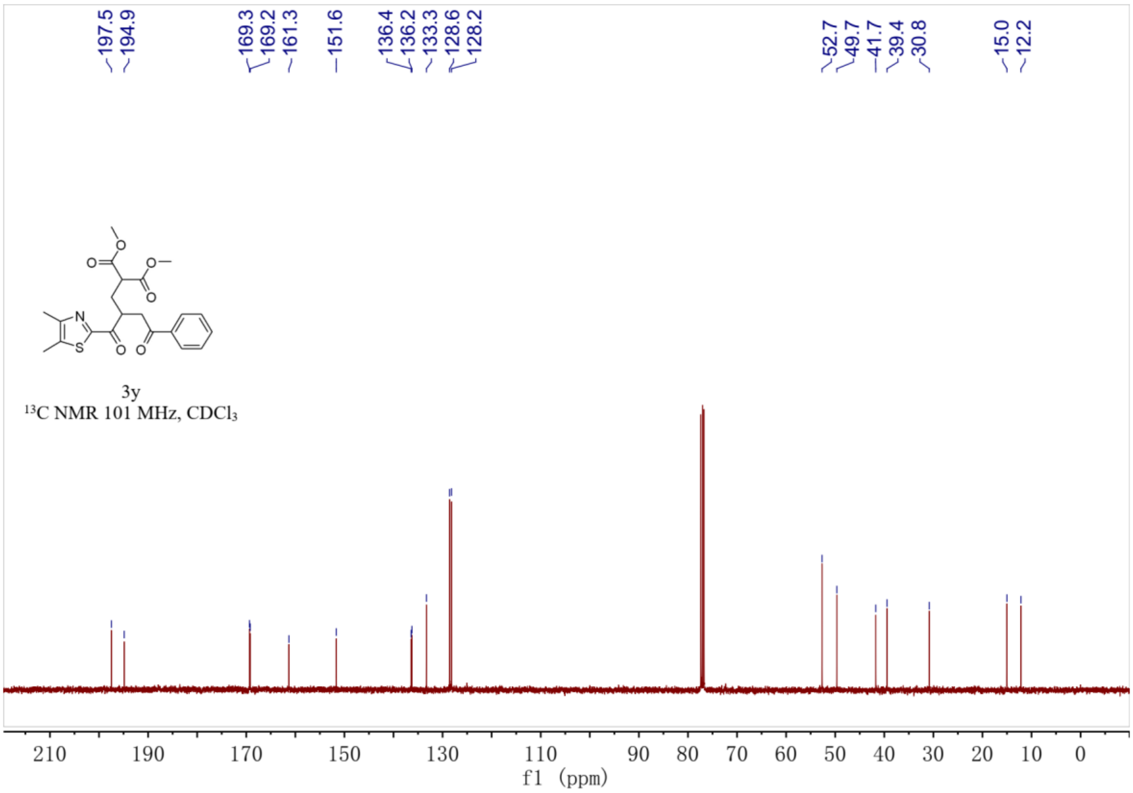
197.5 194.9
169.3 169.2 161.3 151.6
136.4 136.2 133.3 128.6 128.2
52.7 49.7 41.7 39.4 30.8
15.0 12.2
3y
^{13}C NMR 101 MHz, $CDCl_3$
210 190 170 150 130 110 90 80 70 60 50 40 30 20 10 0
f1 (ppm)

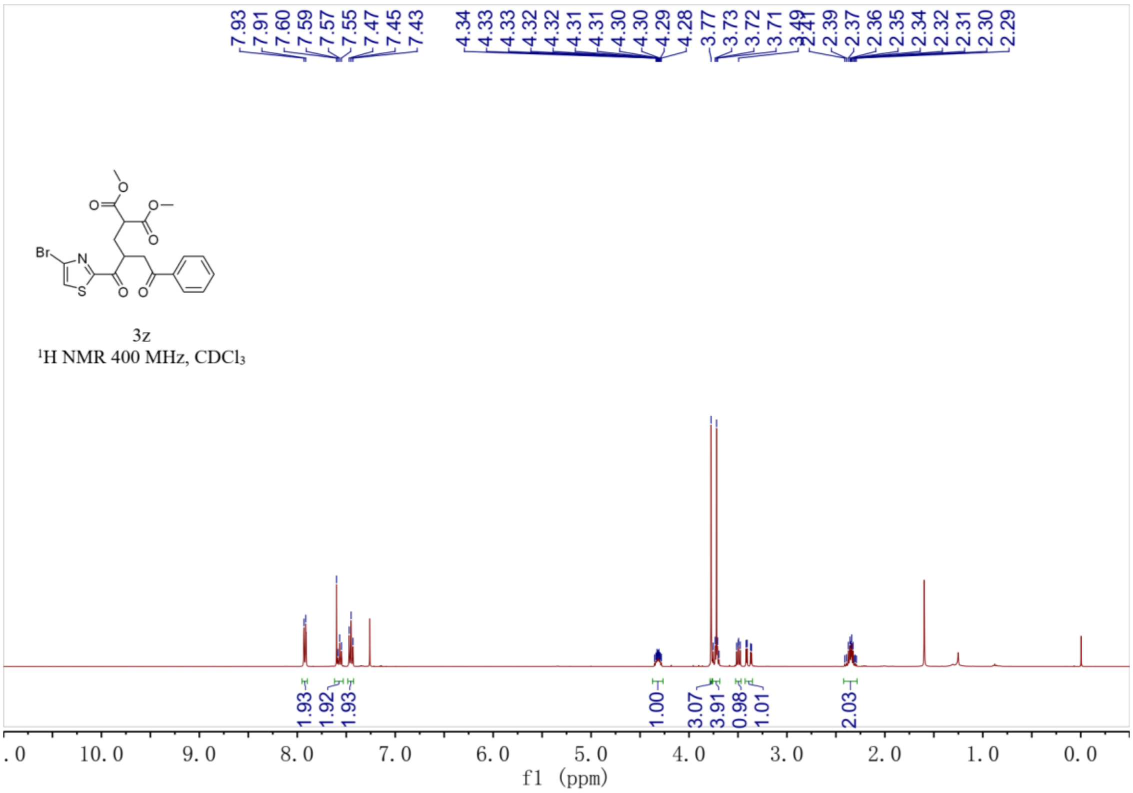
7.93
7.91
7.60
7.59
7.57
7.55
7.47
7.45
7.43
4.34
4.33
4.33
4.32
4.32
4.31
4.31
4.30
4.29
4.28
3.77
3.73
3.72
3.71
3.49
3.41
2.39
2.37
2.36
2.35
2.34
2.32
2.31
2.30
2.29
Br
N
S
O
3z
1H NMR 400 MHz, CDCl3
1.93
1.92
1.93
1.00
3.07
3.91
0.98
1.01
2.03
10.0
9.0
8.0
7.0
6.0
5.0
4.0
3.0
2.0
1.0
0.0
f1 (ppm)

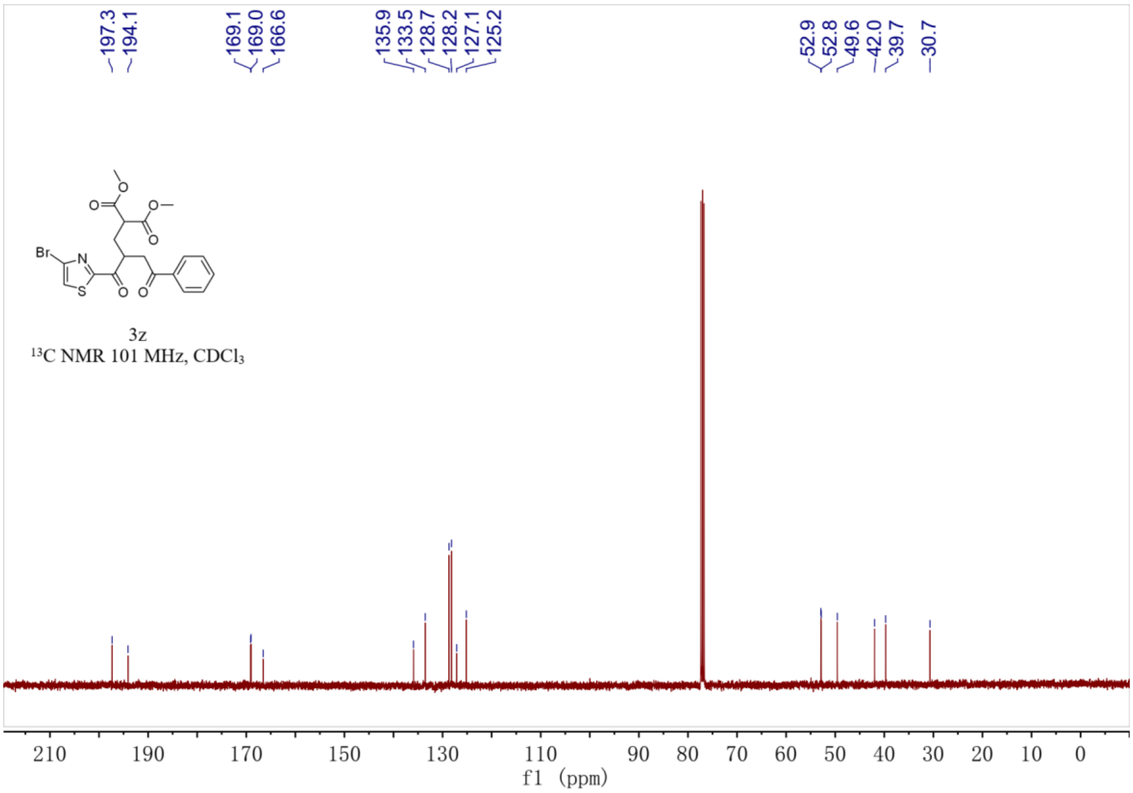
197.3
194.1
169.1
169.0
166.6
135.9
133.5
128.7
128.2
127.1
125.2
52.9
52.8
49.6
42.0
39.7
30.7
Br
N
S
O
3z
13C NMR 101 MHz, CDCl3
210
190
170
150
130
110
90
80
70
60
50
40
30
20
10
0
f1 (ppm)

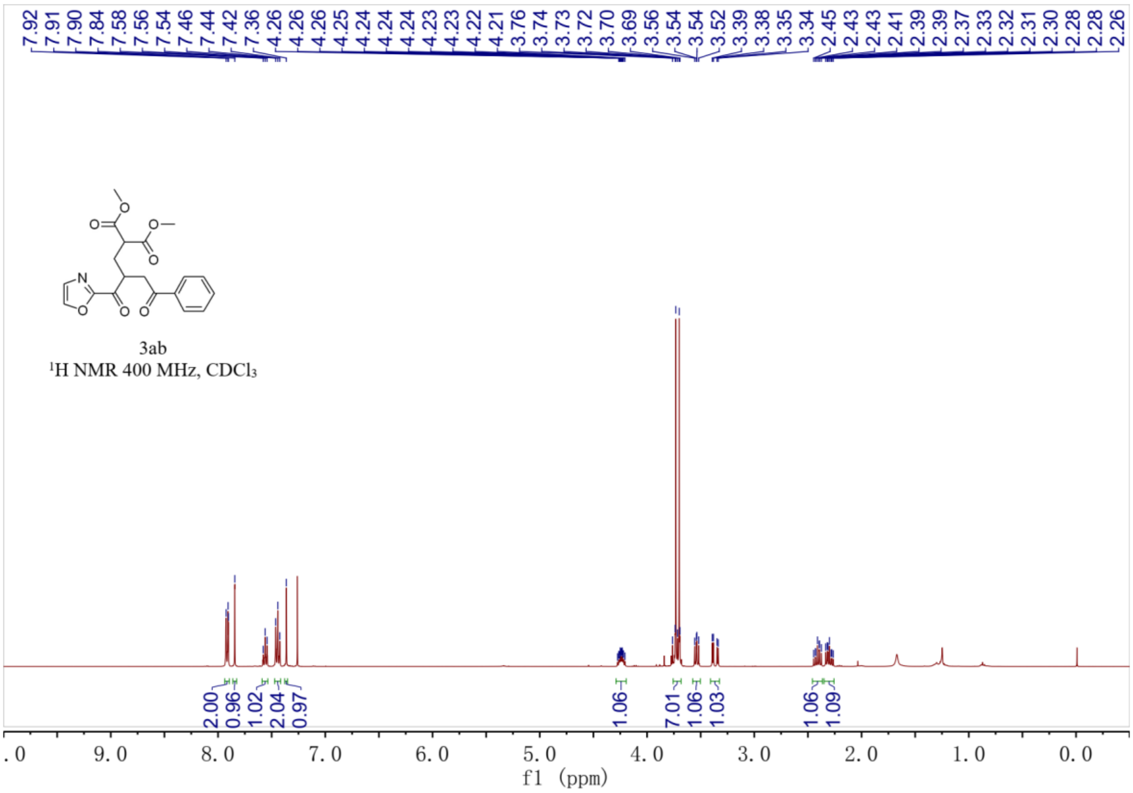
7.92
7.91
7.90
7.84
7.58
7.56
7.54
7.46
7.44
7.42
7.36
4.26
4.26
4.25
4.24
4.24
4.24
4.23
4.22
4.21
3.76
3.74
3.73
3.72
3.70
3.69
3.56
3.54
3.52
3.39
3.38
3.35
3.34
2.45
2.43
2.43
2.41
2.39
2.39
2.37
2.33
2.32
2.31
2.30
2.28
2.28
2.26
3ab
¹H NMR 400 MHz, CDCl₃
2.00
0.96
1.02
2.04
0.97
1.06
7.01
1.06
1.03
1.06
1.09
f1 (ppm)

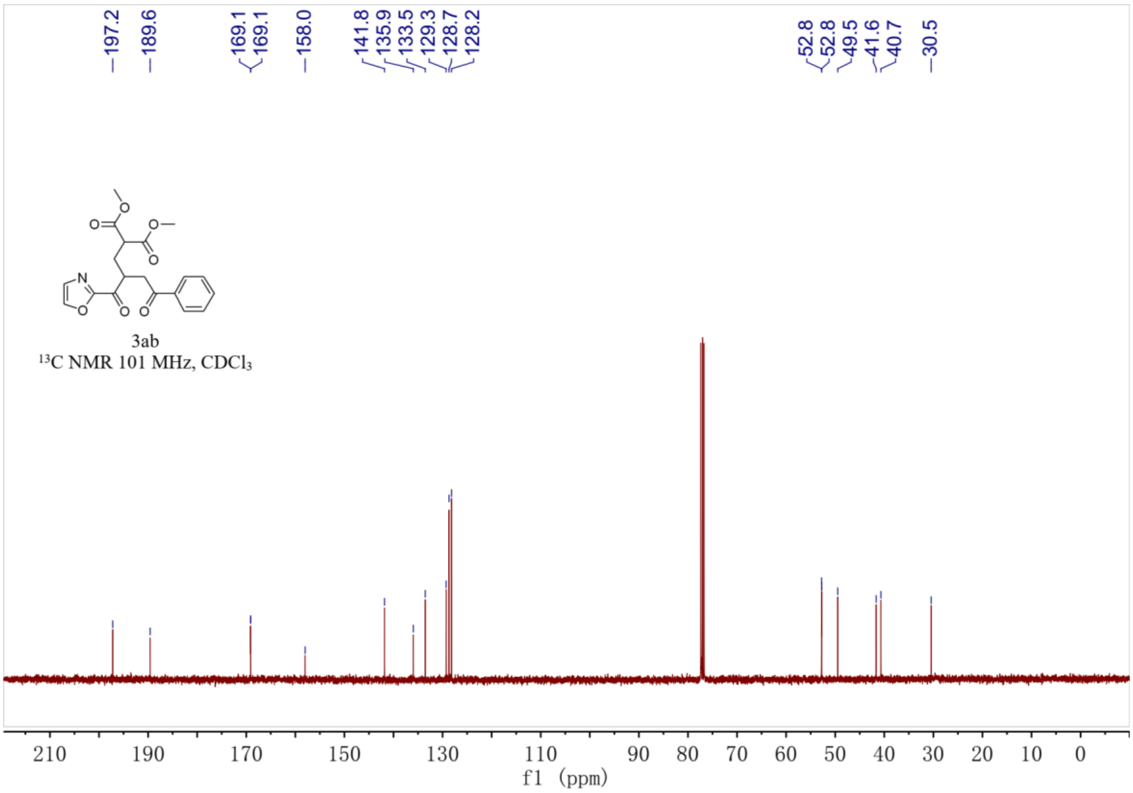
197.2
189.6
169.1
169.1
158.0
141.8
135.9
133.5
129.3
128.7
128.2
52.8
52.8
49.5
41.6
40.7
30.5
3ab
¹³C NMR 101 MHz, CDCl₃
f1 (ppm)

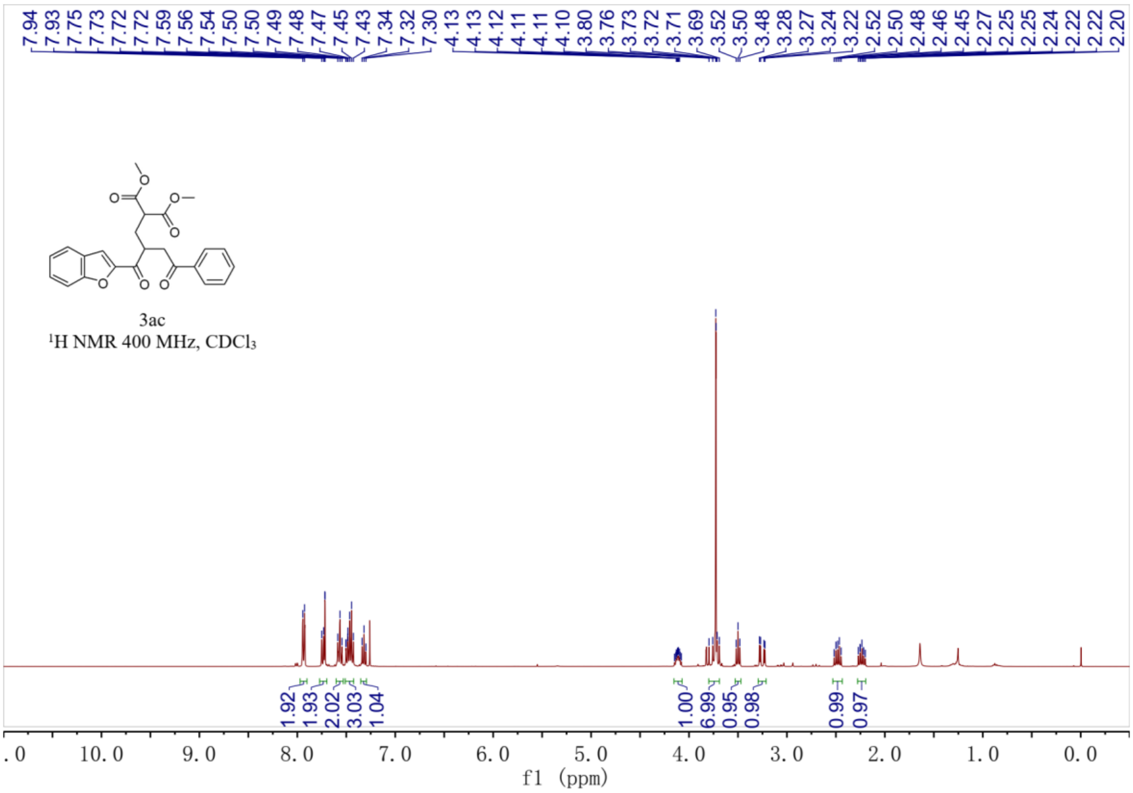

7.94
7.93
7.75
7.73
7.72
7.72
7.59
7.56
7.54
7.50
7.50
7.49
7.48
7.47
7.45
7.43
7.34
7.32
7.30
4.13
4.13
4.12
4.11
4.11
4.10
3.80
3.76
3.73
3.72
3.71
3.69
3.52
3.50
3.48
3.28
3.27
3.24
3.22
2.52
2.50
2.48
2.46
2.45
2.27
2.25
2.25
2.24
2.22
2.22
2.20
3ac
1H NMR 400 MHz, CDCl3
1.92
1.93
2.02
3.03
1.04
1.00
6.99
0.95
0.98
0.99
0.97
f1 (ppm)

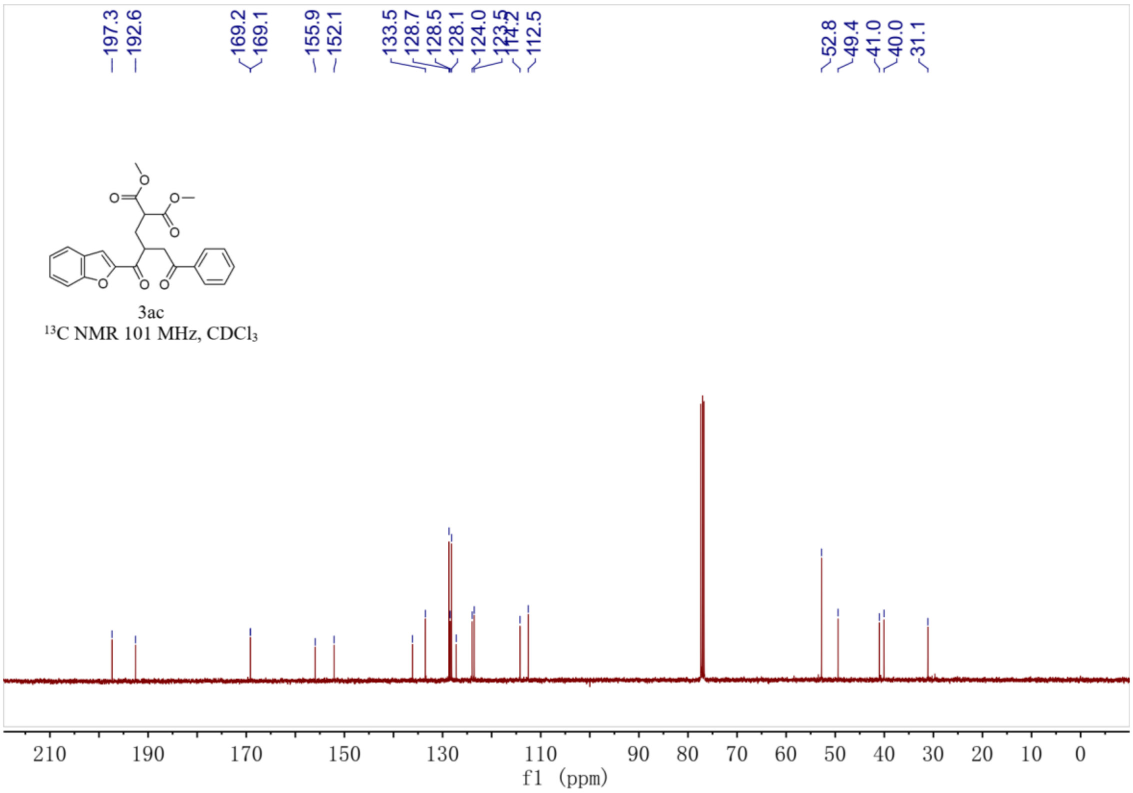

197.3
192.6
169.2
169.1
155.9
152.1
133.5
128.7
128.5
128.1
124.0
123.5
114.2
112.5
52.8
49.4
41.0
40.0
31.1
3ac
13C NMR 101 MHz, CDCl3
f1 (ppm)

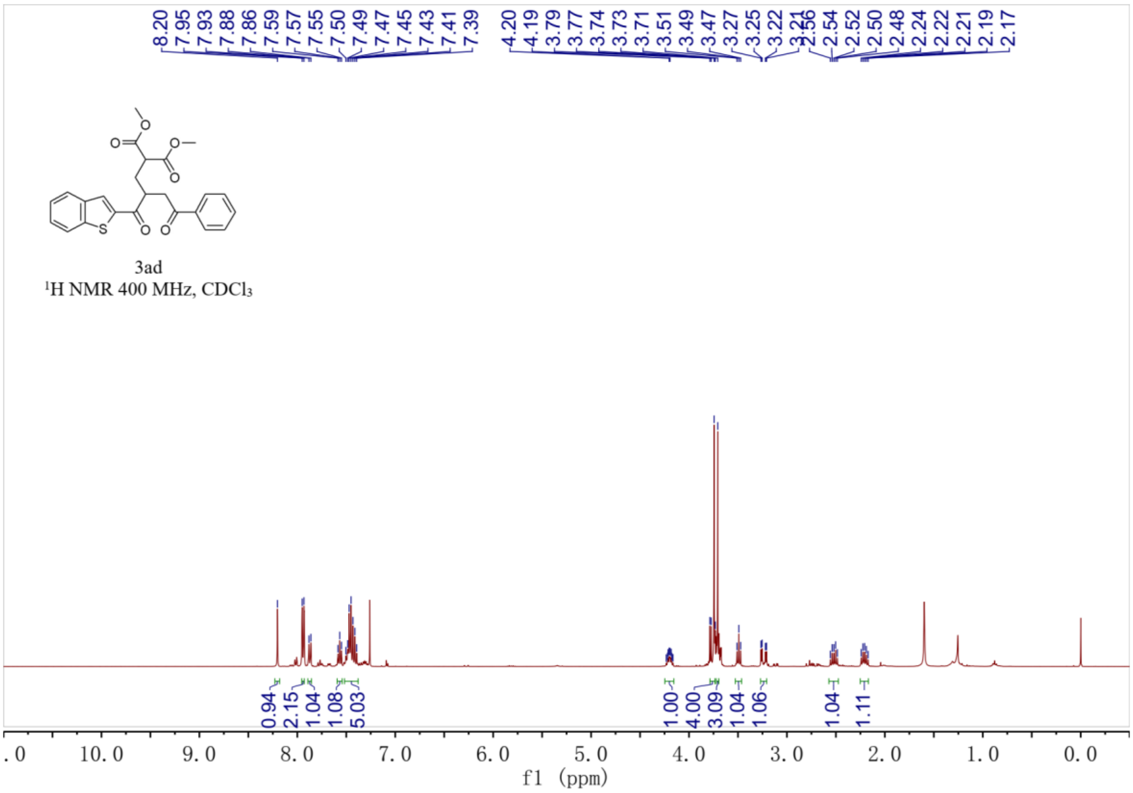
8.20
7.95
7.93
7.88
7.86
7.59
7.57
7.55
7.50
7.49
7.47
7.45
7.43
7.41
7.39
4.20
4.19
3.79
3.77
3.74
3.73
3.71
3.51
3.49
3.47
3.27
3.25
3.22
3.21
2.56
2.54
2.52
2.50
2.48
2.24
2.22
2.21
2.19
2.17
3ad
1H NMR 400 MHz, CDCl3
0.94
2.15
1.04
1.08
5.03
1.00
4.00
3.09
1.04
1.06
1.04
1.11
f1 (ppm)

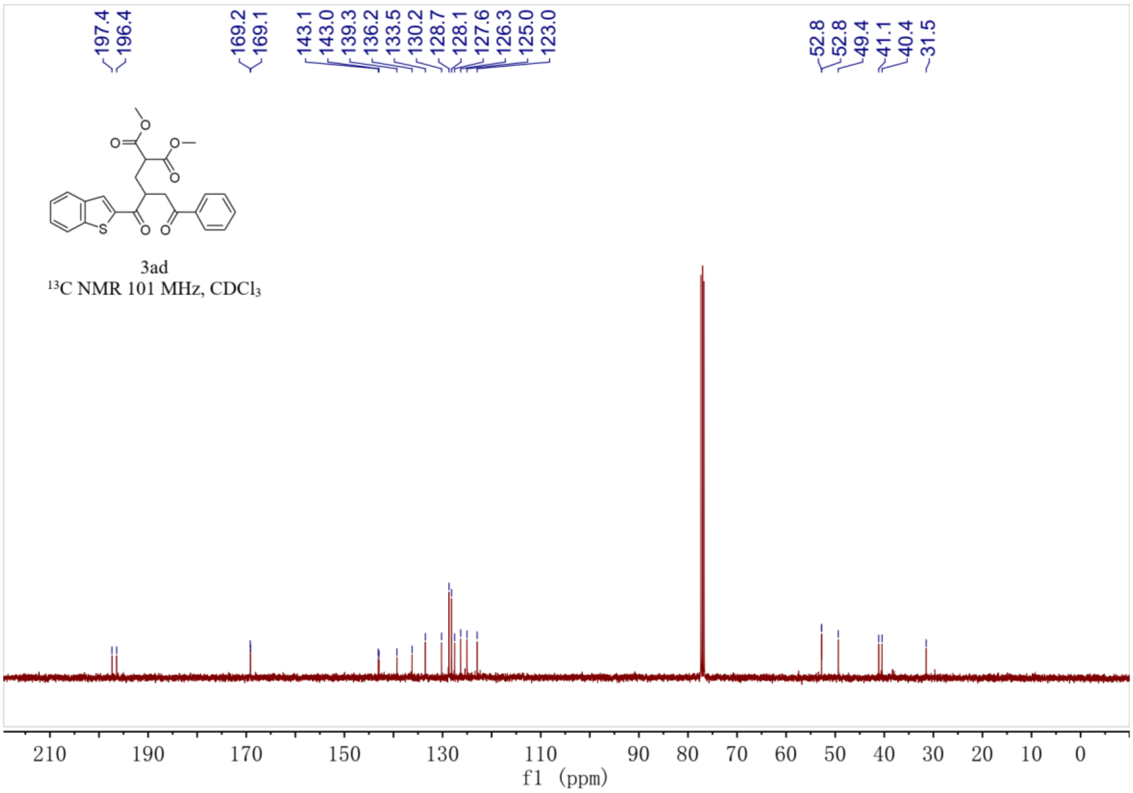
197.4
196.4
169.2
169.1
143.1
143.0
139.3
136.2
133.5
130.2
128.7
128.1
127.6
126.3
125.0
123.0
52.8
52.8
49.4
41.1
40.4
31.5
3ad
13C NMR 101 MHz, CDCl3
f1 (ppm)

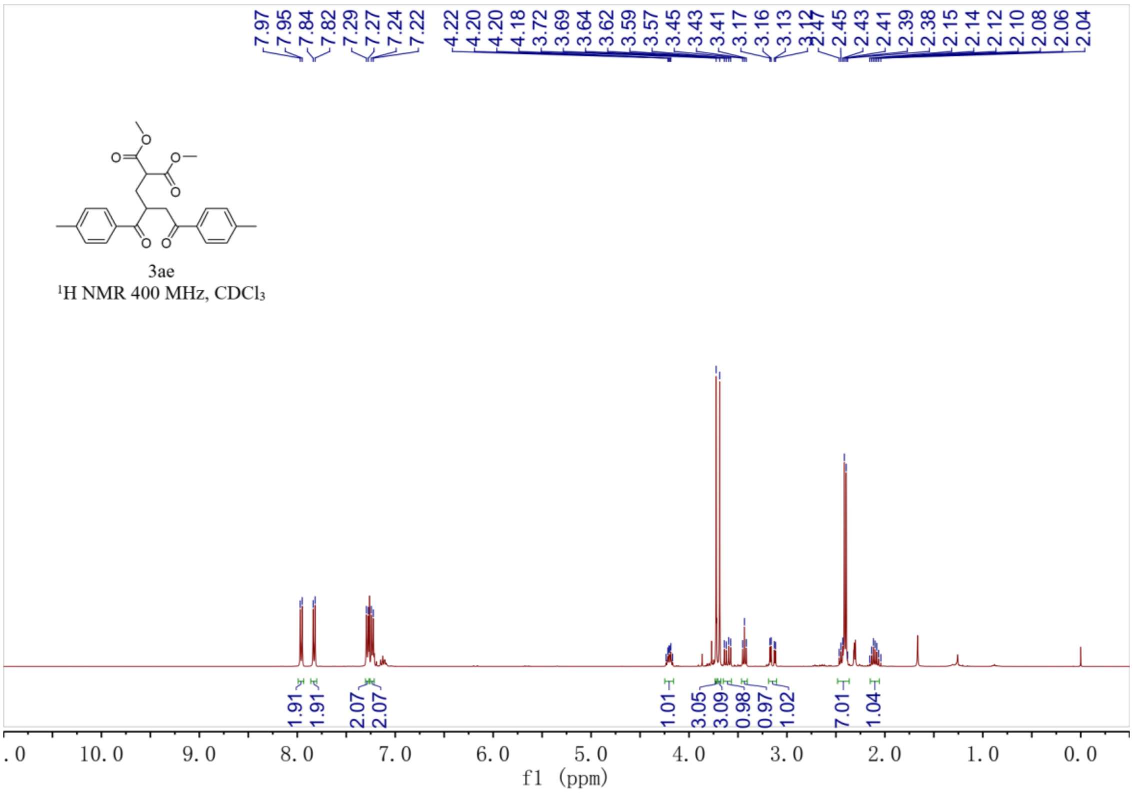
7.97
7.95
7.84
7.82
7.29
7.27
7.24
7.22
4.22
4.20
4.20
4.18
3.72
3.69
3.64
3.62
3.59
3.57
3.45
3.43
3.41
3.17
3.16
3.13
3.12
2.47
2.45
2.43
2.41
2.39
2.38
2.15
2.14
2.12
2.10
2.08
2.06
2.04
3ae
¹H NMR 400 MHz, CDCl₃
1.91
1.91
2.07
2.07
1.01
3.05
3.09
0.98
0.97
1.02
7.01
1.04
f1 (ppm)

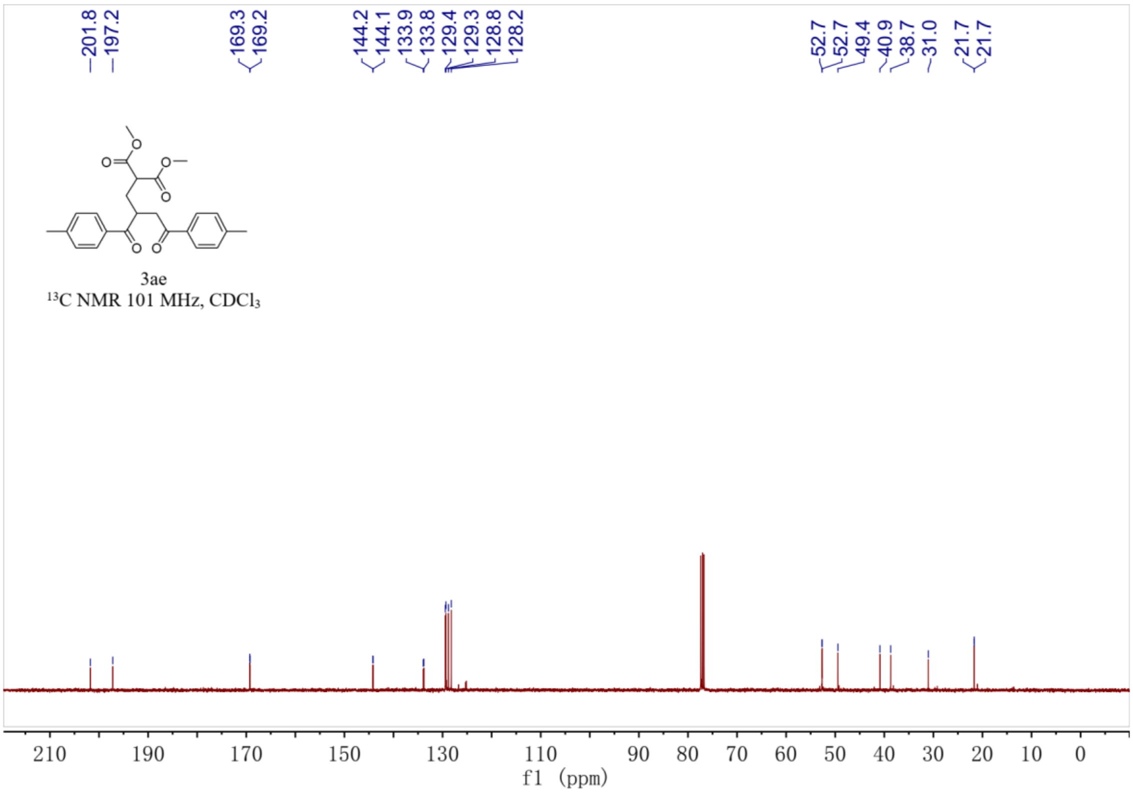
201.8
197.2
169.3
169.2
144.2
144.1
133.9
133.8
129.4
129.3
128.8
128.2
52.7
52.7
49.4
40.9
38.7
31.0
21.7
21.7
3ae
¹³C NMR 101 MHz, CDCl₃
f1 (ppm)

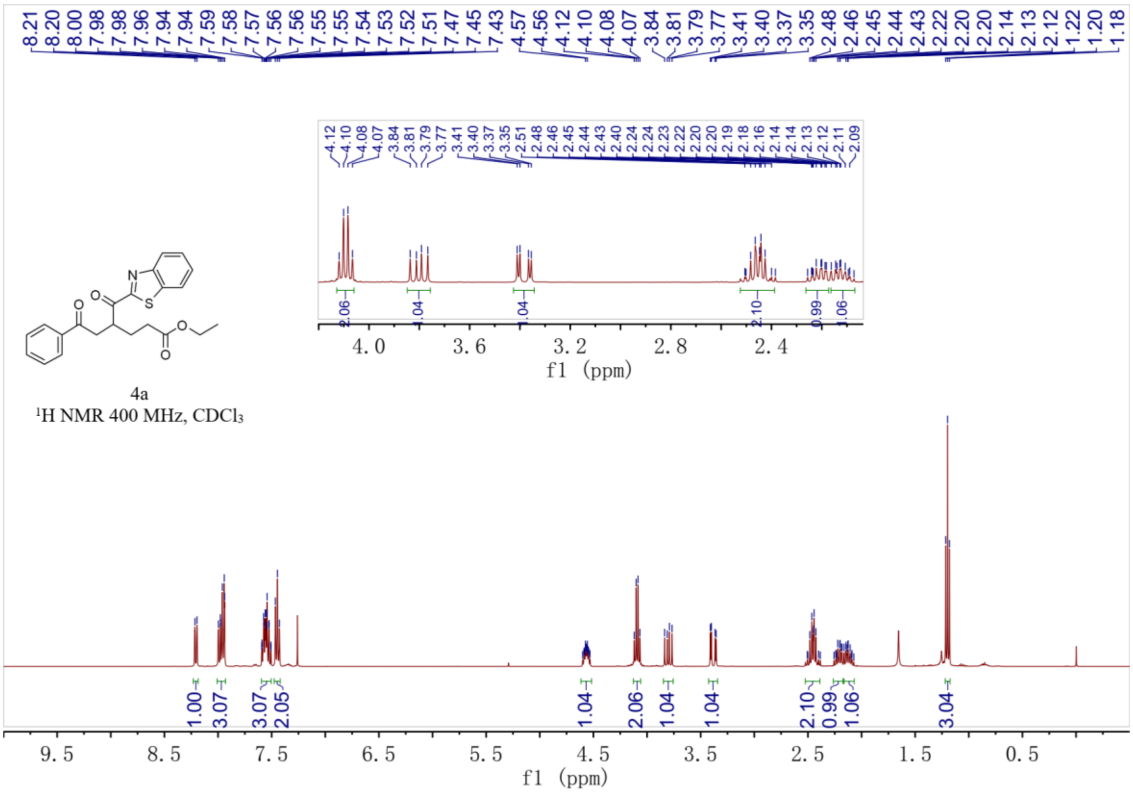
8.21 8.20 8.00 7.98 7.98 7.96 7.94 7.94 7.59 7.58 7.57 7.56 7.56 7.55 7.55 7.54 7.53 7.52 7.51 7.47 7.45 7.43 4.57 4.56 4.12 4.10 4.08 4.07 3.84 3.81 3.79 3.77 3.41 3.40 3.37 3.35 2.48 2.46 2.45 2.44 2.43 2.22 2.20 2.20 2.14 2.13 2.12 1.22 1.20 1.18
4a
^{1}H NMR 400 MHz, CDCl$_3$
f1 (ppm)

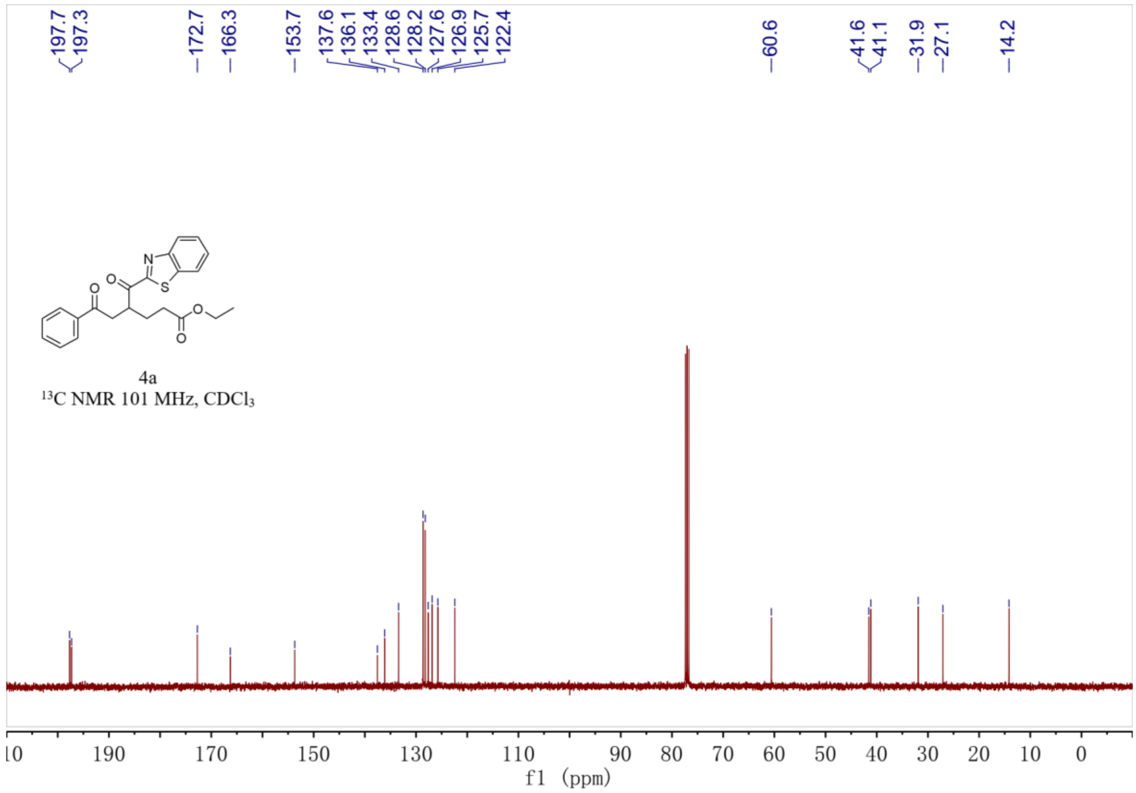
197.7 197.3 172.7 166.3 153.7 137.6 136.1 133.4 128.6 128.2 127.6 126.9 125.7 122.4 60.6 41.6 41.1 31.9 27.1 14.2
4a
^{13}C NMR 101 MHz, CDCl$_3$
f1 (ppm)

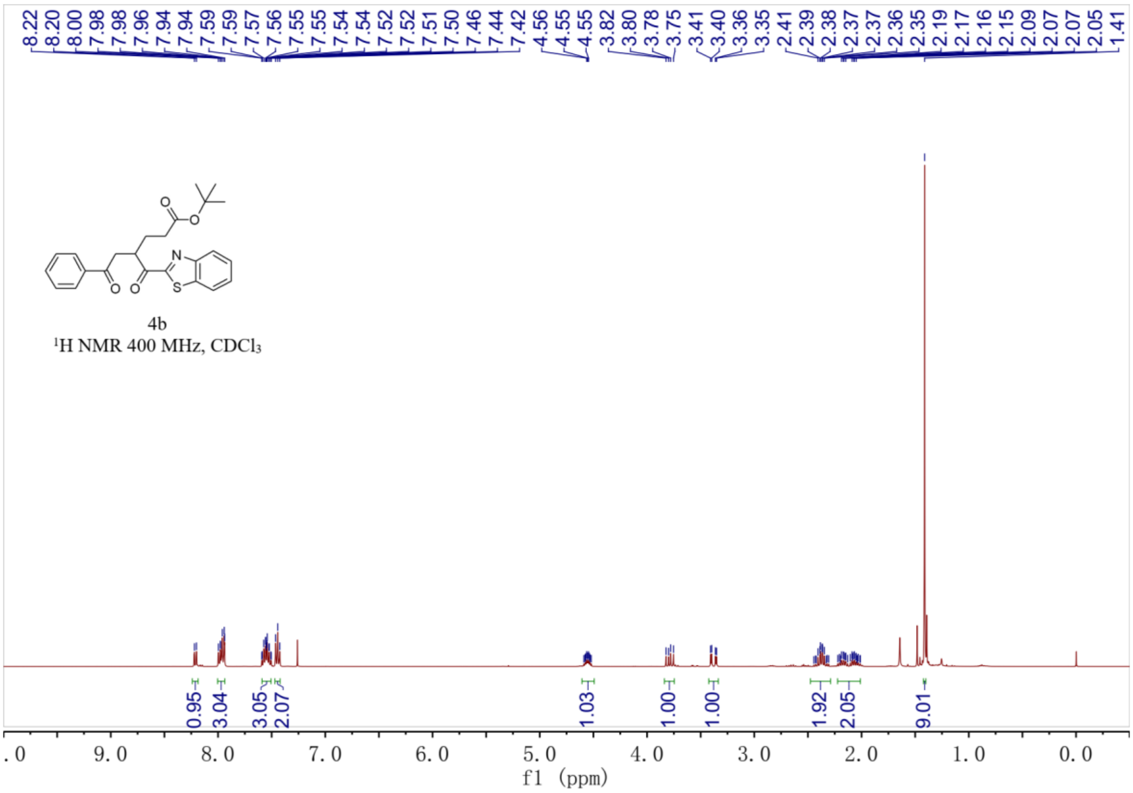
8.22 8.20 8.00 7.98 7.96 7.94 7.59 7.57 7.56 7.55 7.55 7.54 7.54 7.52 7.52 7.51 7.50 7.46 7.44 7.42 4.56 4.55 4.55 3.82 3.80 3.78 3.75 3.41 3.40 3.36 3.35 2.41 2.39 2.38 2.37 2.37 2.36 2.35 2.19 2.17 2.16 2.15 2.09 2.07 2.07 2.05 1.41
N
S
O
4b
1H NMR 400 MHz, CDCl3
0.95 3.04 3.05 2.07 1.03 1.00 1.00 1.92 2.05 9.01
9.0 8.0 7.0 6.0 5.0 4.0 3.0 2.0 1.0 0.0
f1 (ppm)

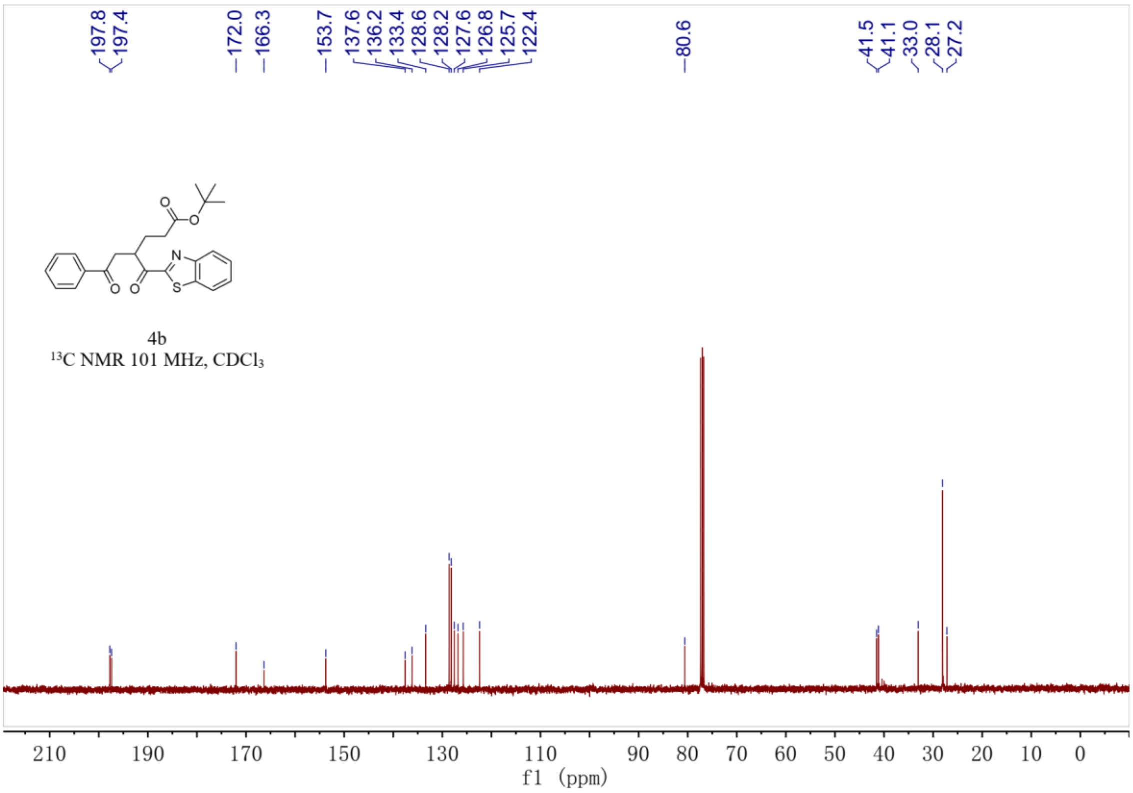
197.8 197.4 172.0 166.3 153.7 137.6 136.2 133.4 128.6 128.2 127.6 126.8 125.7 122.4 80.6 41.5 41.1 33.0 28.1 27.2
N
S
O
4b
13C NMR 101 MHz, CDCl3
210 190 170 150 130 110 90 80 70 60 50 40 30 20 10 0
f1 (ppm)

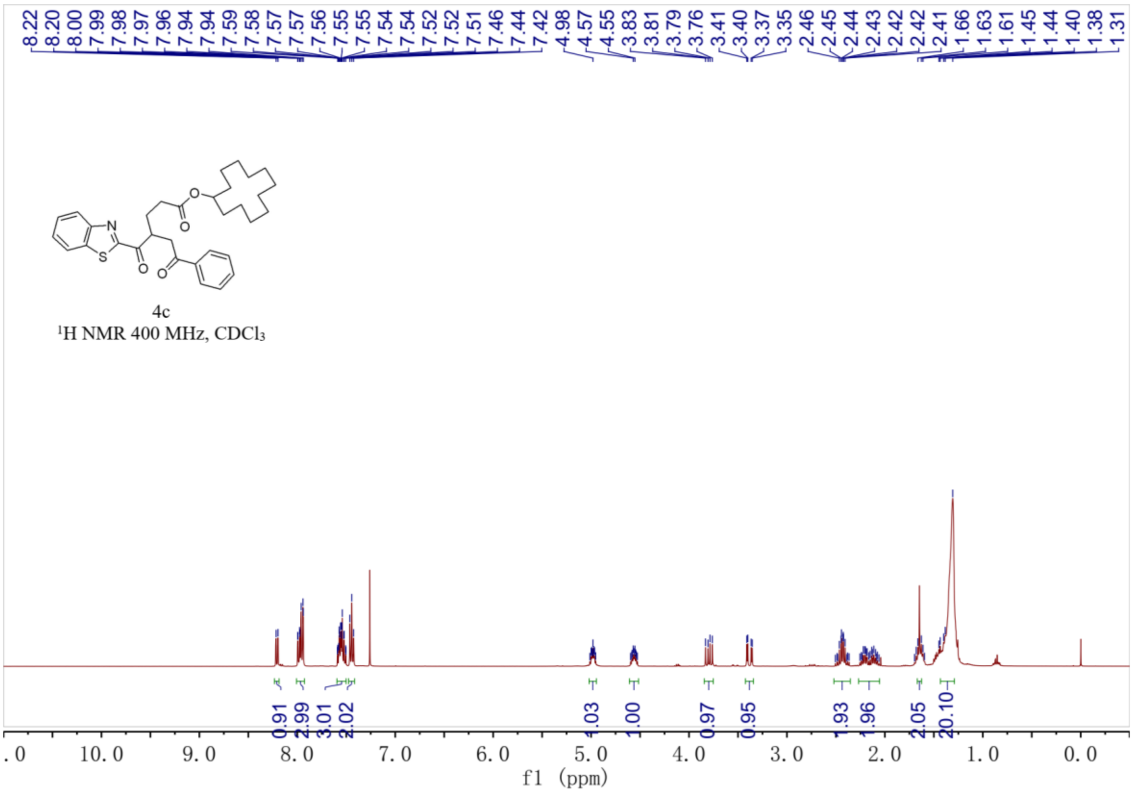
8.22
8.20
8.00
7.99
7.98
7.97
7.96
7.94
7.94
7.59
7.58
7.57
7.57
7.56
7.55
7.55
7.54
7.54
7.52
7.51
7.46
7.44
7.42
4.98
4.57
4.55
3.83
3.81
3.79
3.76
3.41
3.40
3.37
3.35
2.46
2.45
2.44
2.43
2.42
2.42
2.41
1.66
1.63
1.61
1.45
1.44
1.40
1.38
1.31
4c
¹H NMR 400 MHz, CDCl₃
0.91
2.99
3.01
2.02
1.03
1.00
0.97
0.95
1.93
1.96
2.05
20.10
10.0
9.0
8.0
7.0
6.0
5.0
4.0
3.0
2.0
1.0
0.0
f1 (ppm)

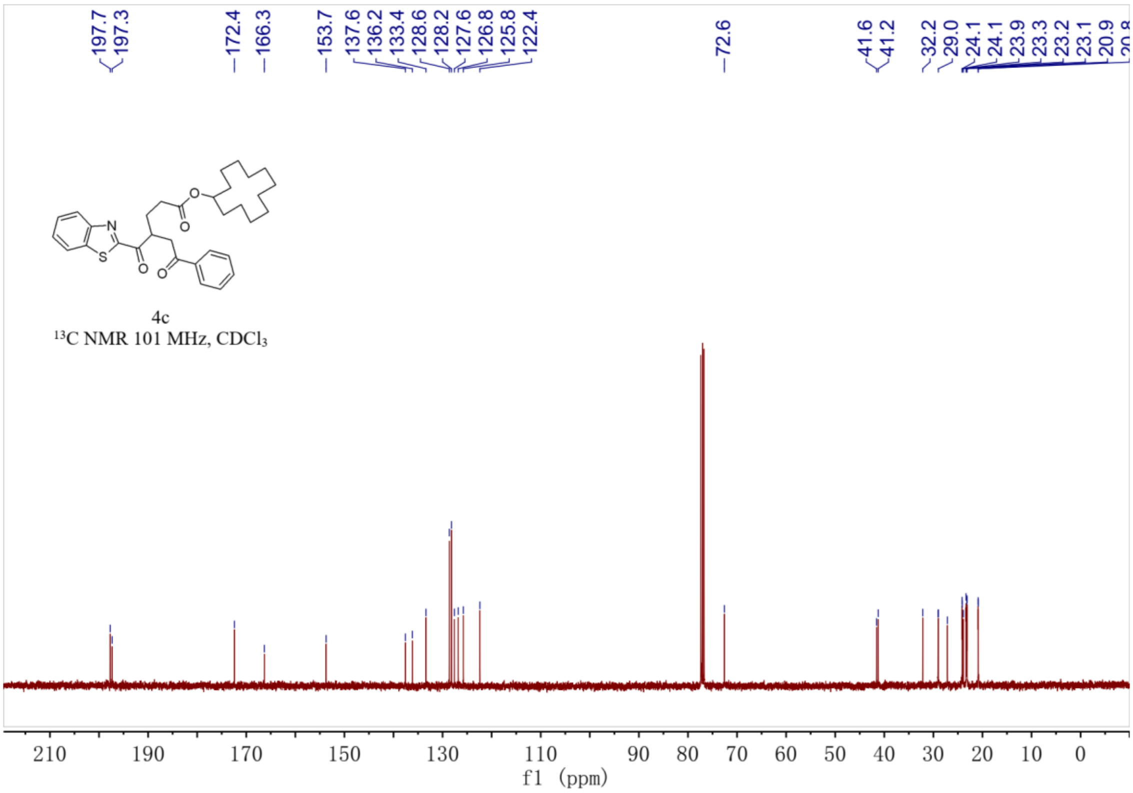
197.7
197.3
172.4
166.3
153.7
137.6
136.2
133.4
128.6
128.2
127.6
126.8
125.8
122.4
72.6
41.6
41.2
32.2
29.0
24.1
24.1
23.9
23.3
23.2
23.1
20.9
4c
¹³C NMR 101 MHz, CDCl₃
210
190
170
150
130
110
90
80
70
60
50
40
30
20
10
0
f1 (ppm)

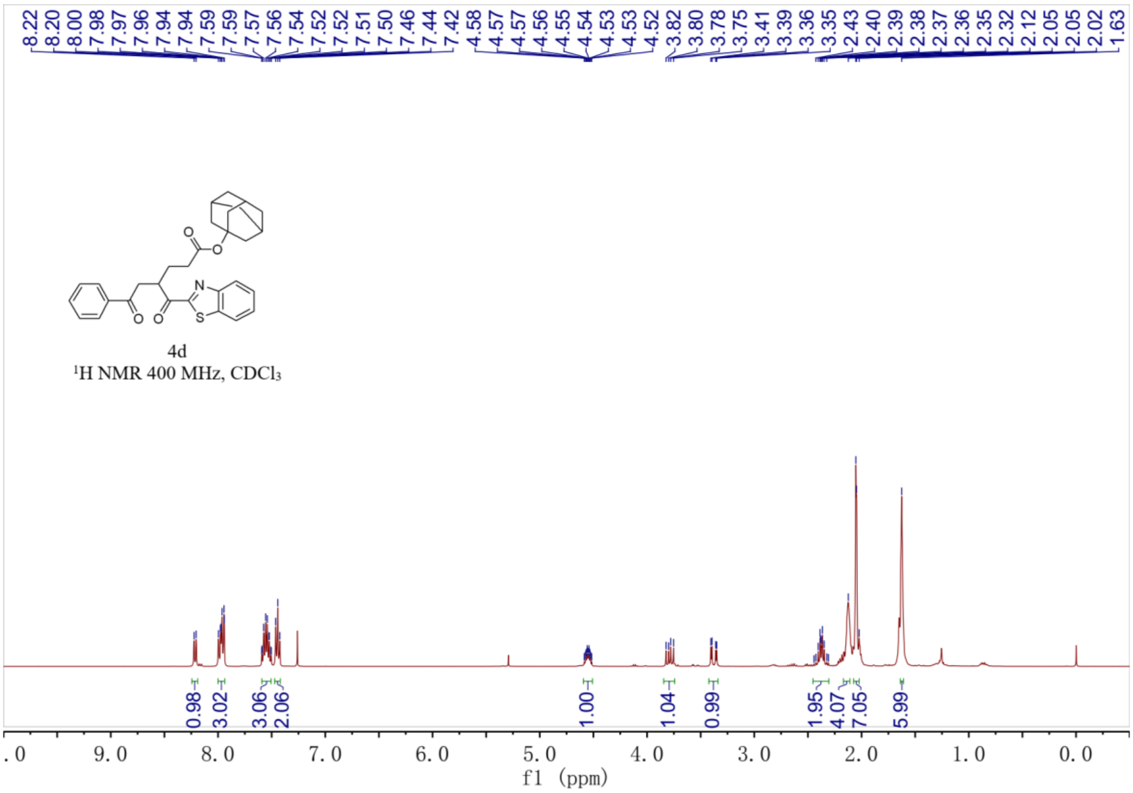
4d
1H NMR 400 MHz, CDCl3
8.22 8.20 8.00 7.98 7.97 7.96 7.94 7.94 7.59 7.57 7.56 7.54 7.52 7.51 7.50 7.46 7.44 7.42 4.58 4.57 4.57 4.56 4.55 4.54 4.53 4.53 4.52 3.82 3.80 3.78 3.75 3.41 3.39 3.36 3.35 2.43 2.40 2.39 2.38 2.37 2.36 2.35 2.32 2.12 2.05 2.05 2.02 1.63
0.98 3.02 3.06 2.06 1.00 1.04 0.99 1.95 4.07 7.05 5.99
f1 (ppm)

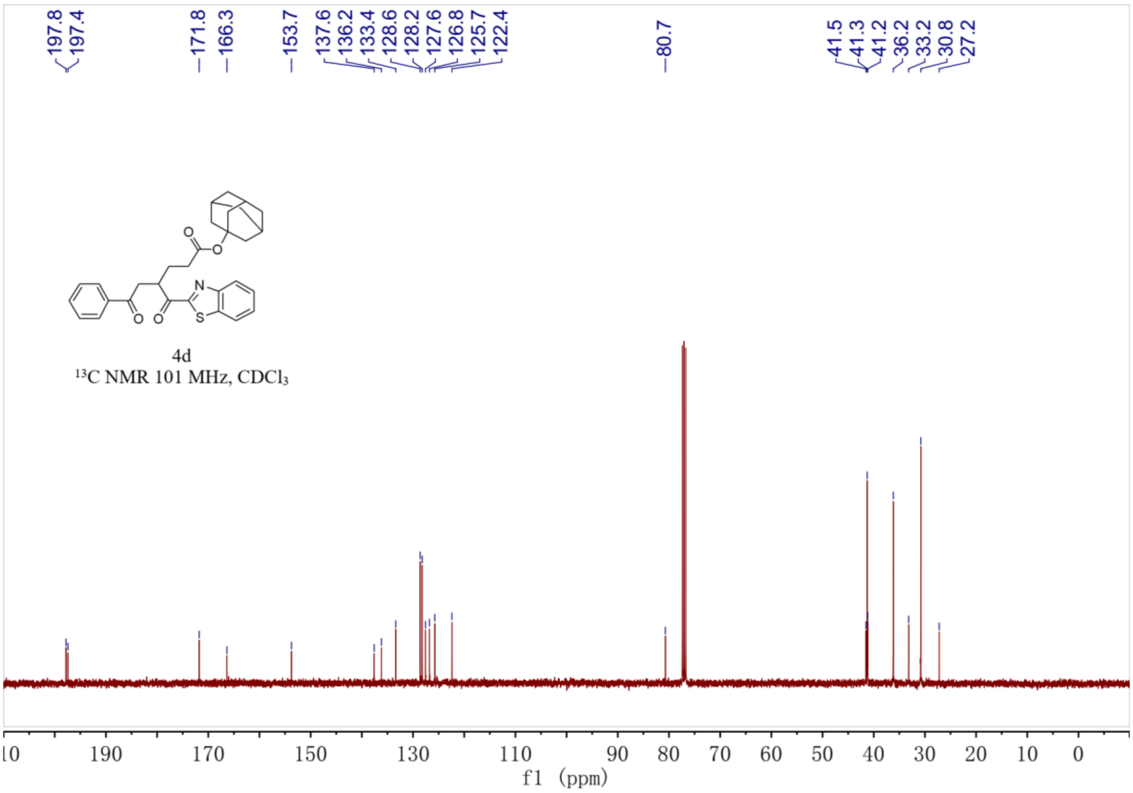
4d
13C NMR 101 MHz, CDCl3
197.8 197.4 171.8 166.3 153.7 137.6 136.2 133.4 128.6 128.2 127.6 126.8 125.7 122.4 80.7 41.5 41.3 41.2 36.2 33.2 30.8 27.2
f1 (ppm)

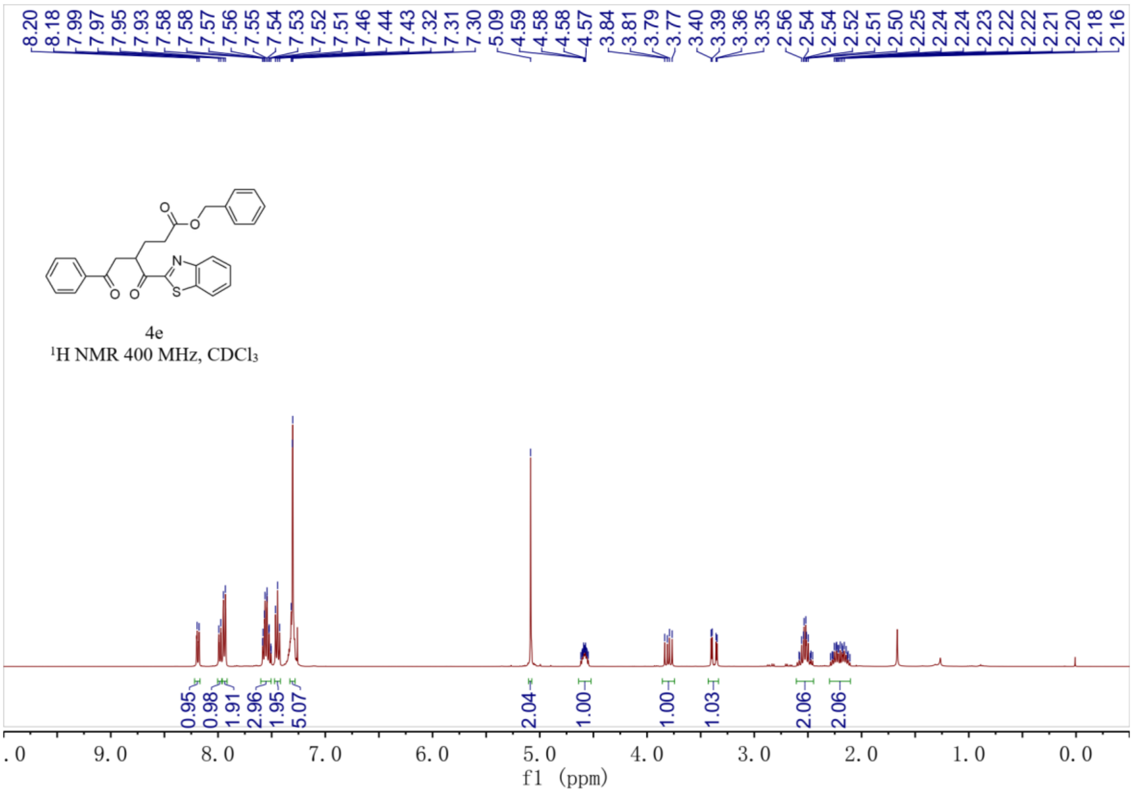
8.20 8.18 7.99 7.97 7.95 7.93 7.58 7.58 7.57 7.56 7.55 7.54 7.53 7.52 7.51 7.46 7.44 7.43 7.32 7.31 7.30 5.09 4.59 4.58 4.58 4.57 3.84 3.81 3.79 3.77 3.40 3.39 3.36 3.35 2.56 2.54 2.54 2.52 2.51 2.50 2.25 2.24 2.24 2.23 2.22 2.22 2.21 2.20 2.18 2.16
4e
^{1}H NMR 400 MHz, CDCl$_3$
0.95 0.98 1.91 2.96 1.95 5.07 2.04 1.00 1.00 1.03 2.06 2.06
f1 (ppm)

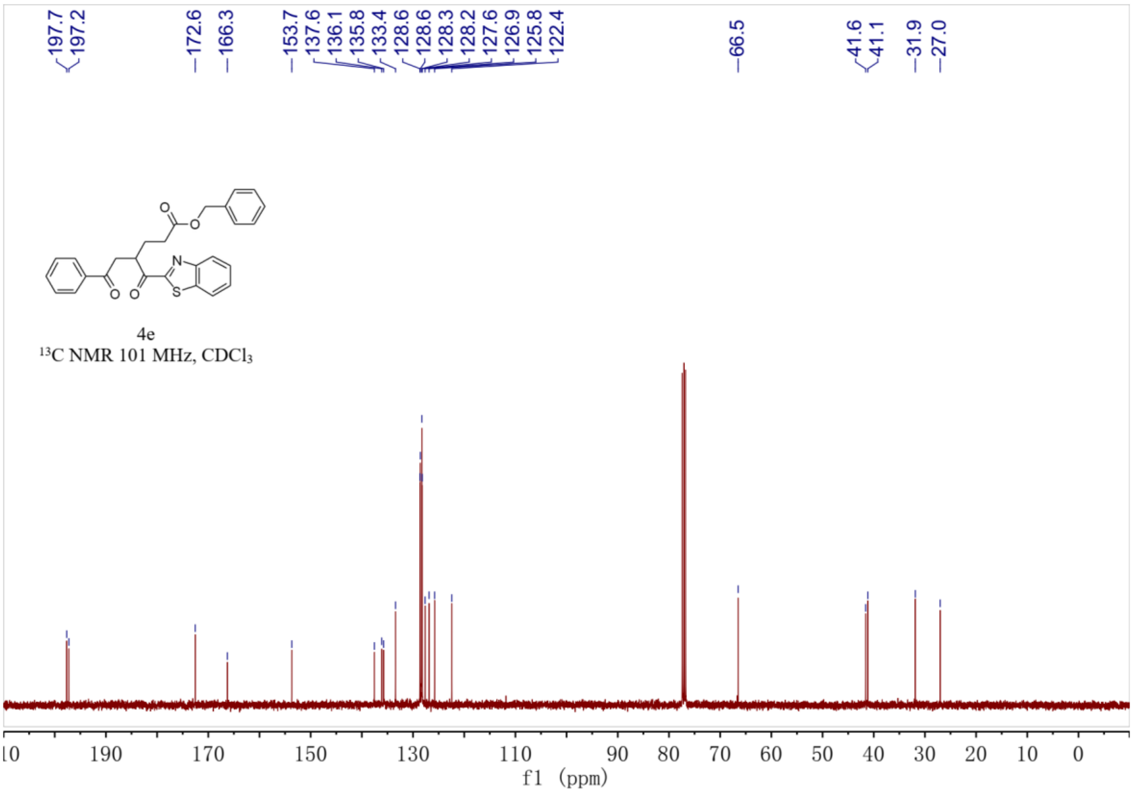
197.7 197.2 172.6 166.3 153.7 137.6 136.1 135.8 133.4 128.6 128.3 128.2 127.6 126.9 125.8 122.4 66.5 41.6 41.1 31.9 27.0
4e
^{13}C NMR 101 MHz, CDCl$_3$
f1 (ppm)

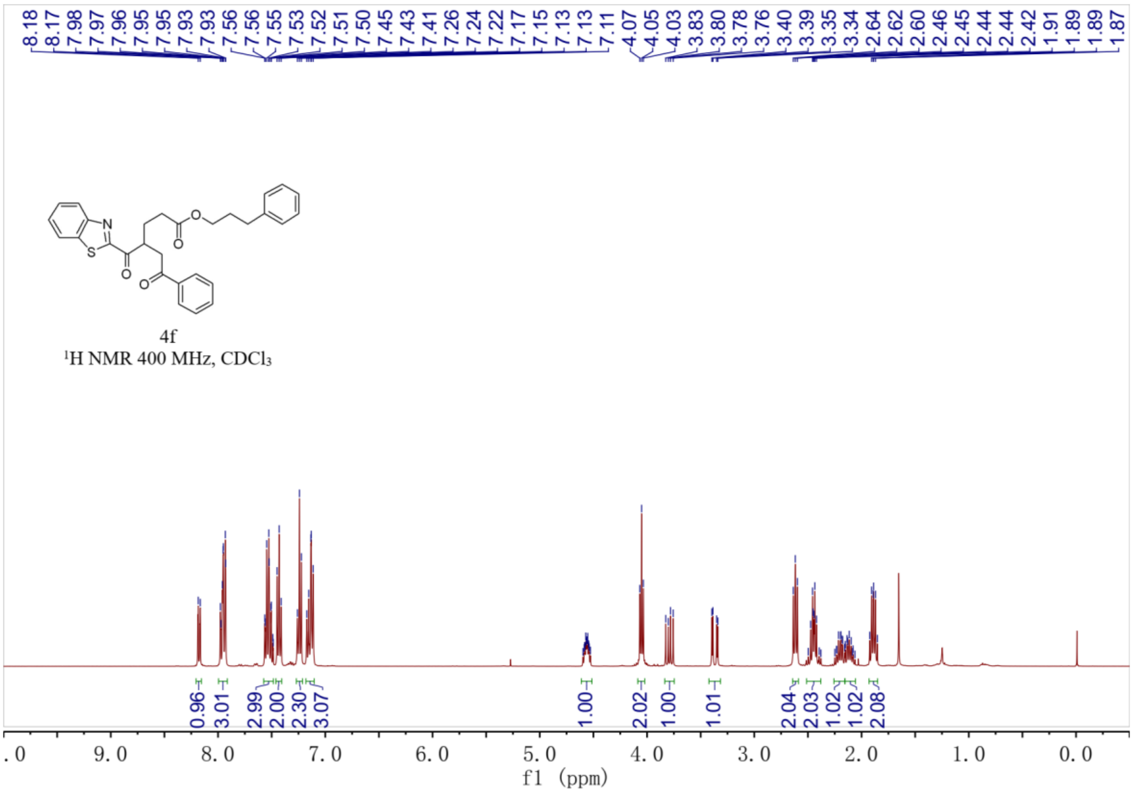
8.18
8.17
7.98
7.97
7.96
7.95
7.95
7.93
7.93
7.56
7.56
7.55
7.53
7.52
7.51
7.50
7.45
7.43
7.41
7.26
7.24
7.22
7.17
7.15
7.13
7.11
4.07
4.05
4.03
3.83
3.80
3.78
3.76
3.40
3.39
3.35
3.34
2.64
2.62
2.60
2.46
2.45
2.44
2.44
2.42
1.91
1.89
1.89
1.87
4f
1H NMR 400 MHz, CDCl3
0.96
3.01
2.99
2.00
2.30
3.07
1.00
2.02
1.00
1.01
2.04
2.03
1.02
1.02
2.08
.0
9.0
8.0
7.0
6.0
5.0
4.0
3.0
2.0
1.0
0.0
f1 (ppm)

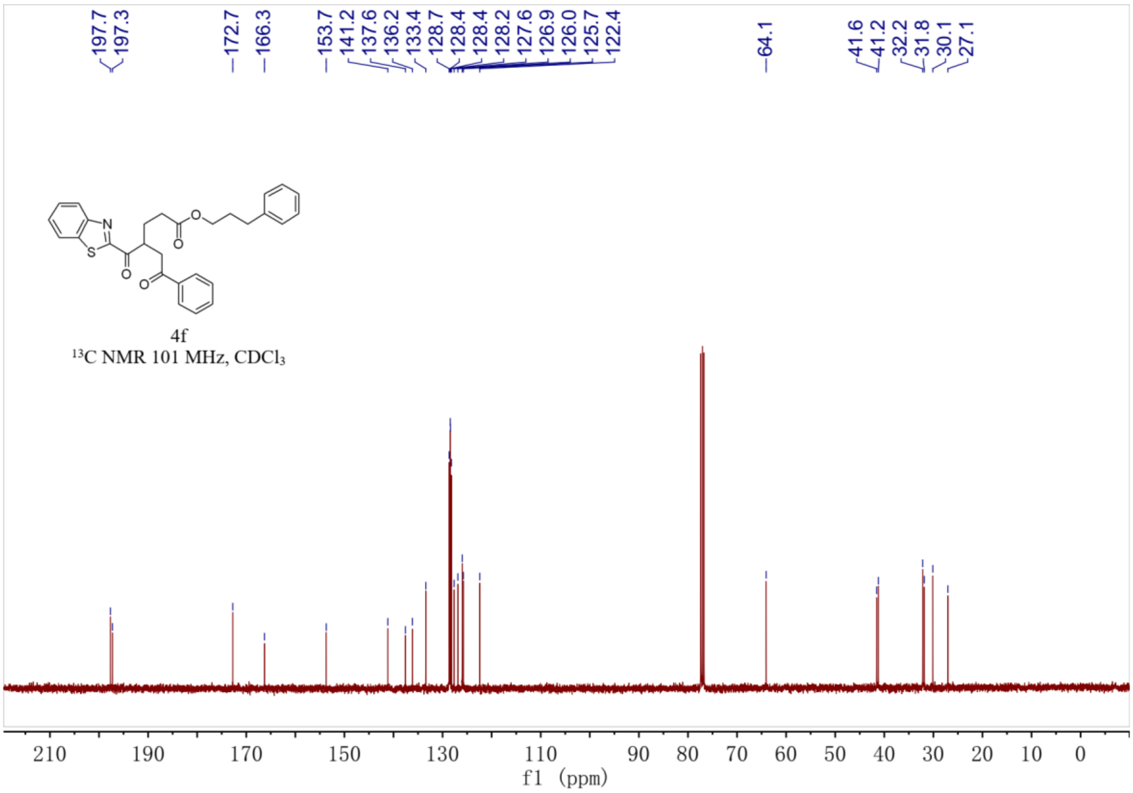
197.7
197.3
172.7
166.3
153.7
141.2
137.6
136.2
133.4
128.7
128.4
128.4
128.2
127.6
126.9
126.0
125.7
122.4
64.1
41.6
41.2
32.2
31.8
30.1
27.1
4f
13C NMR 101 MHz, CDCl3
210
190
170
150
130
110
90
80
70
60
50
40
30
20
10
0
f1 (ppm)

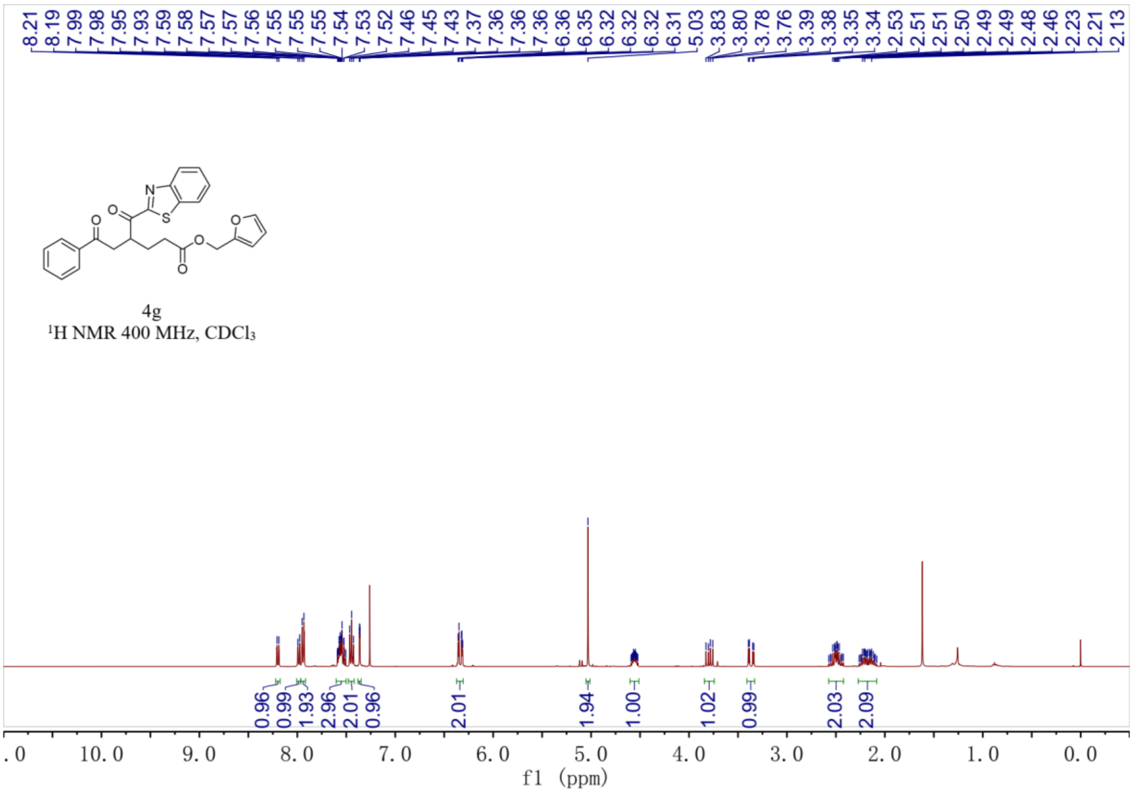
8.21 8.19 7.99 7.98 7.95 7.93 7.59 7.58 7.57 7.56 7.55 7.55 7.54 7.53 7.52 7.46 7.45 7.43 7.37 7.36 7.36 7.36 6.36 6.35 6.32 6.32 6.32 6.31 5.03 3.83 3.80 3.78 3.76 3.39 3.38 3.35 3.34 2.53 2.51 2.51 2.50 2.49 2.49 2.48 2.46 2.23 2.21 2.13
N
O
S
O
O
O
O
4g
¹H NMR 400 MHz, CDCl₃
0.96 0.99 1.93 2.96 2.01 0.96 2.01 1.94 1.00 1.02 0.99 2.03 2.09
.0 10.0 9.0 8.0 7.0 6.0 5.0 4.0 3.0 2.0 1.0 0.0
f1 (ppm)

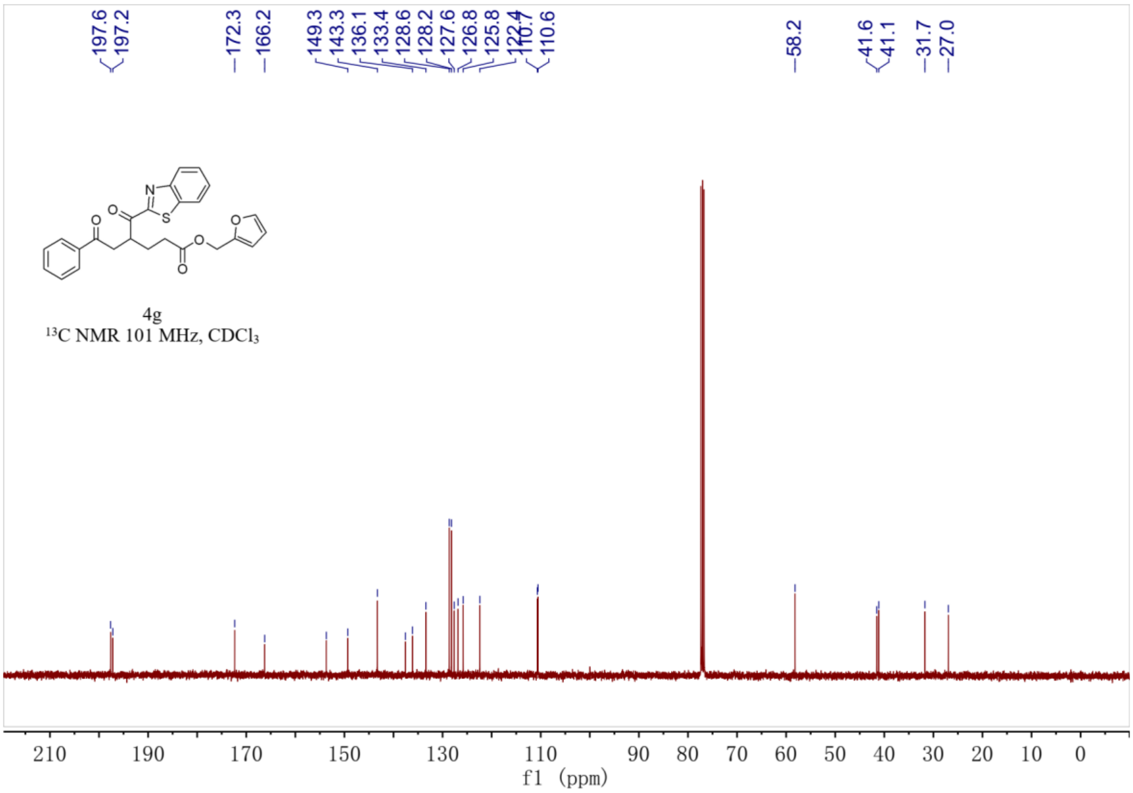
197.6 197.2 172.3 166.2 149.3 143.3 136.1 133.4 128.6 128.2 127.6 126.8 125.8 122.4 110.7 110.6 58.2 41.6 41.1 31.7 27.0
N
O
S
O
O
O
O
4g
¹³C NMR 101 MHz, CDCl₃
210 190 170 150 130 110 90 80 70 60 50 40 30 20 10 0
f1 (ppm)

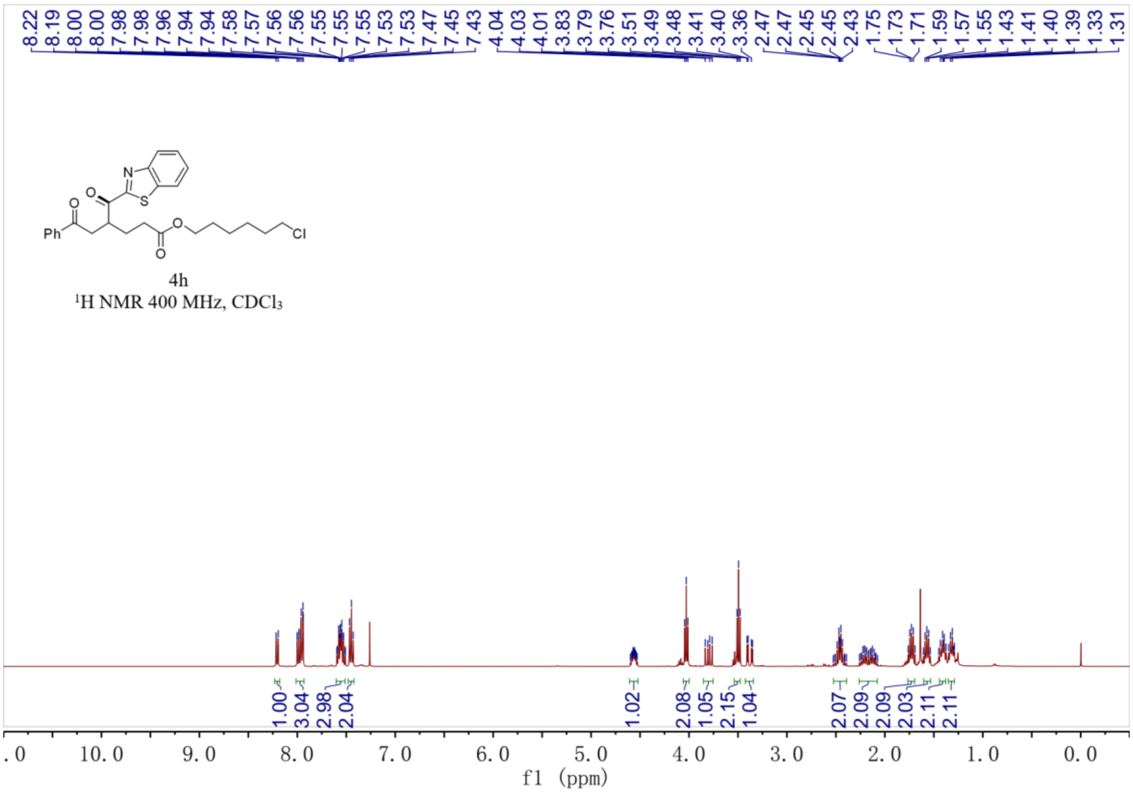
8.22 8.19 8.00 7.98 7.98 7.96 7.94 7.94 7.58 7.57 7.56 7.56 7.55 7.55 7.55 7.53 7.53 7.47 7.45 7.43 4.04 4.03 4.01 3.83 3.79 3.76 3.51 3.49 3.48 3.41 3.40 3.36 2.47 2.47 2.45 2.45 2.43 1.75 1.73 1.71 1.59 1.57 1.55 1.43 1.41 1.40 1.39 1.33 1.31
N
S
O
O
Ph
O
O
Cl
4h
^{1}H NMR 400 MHz, CDCl$_3$
1.00 3.04 2.98 2.04 1.02 2.08 1.05 2.15 1.04 2.07 2.09 2.09 2.03 2.11 2.11
.0 10.0 9.0 8.0 7.0 6.0 5.0 4.0 3.0 2.0 1.0 0.0
f1 (ppm)

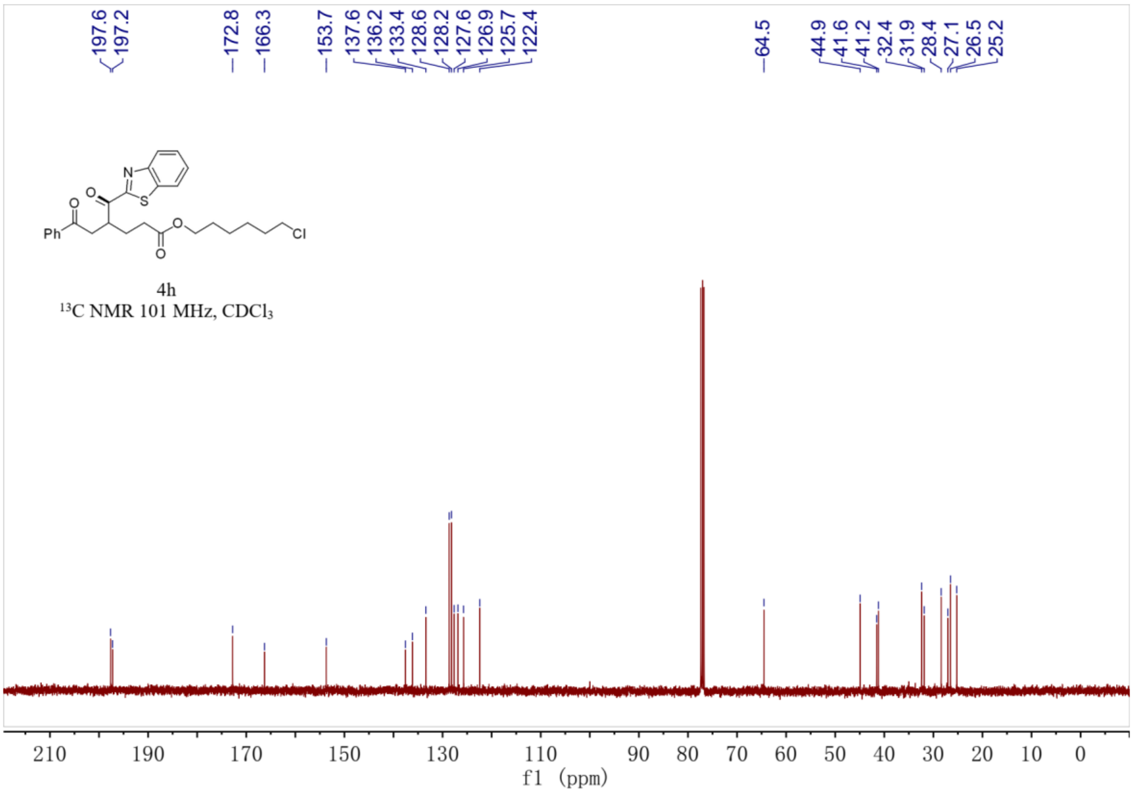
197.6 197.2 172.8 166.3 153.7 137.6 136.2 133.4 128.6 128.2 127.6 126.9 125.7 122.4 64.5 44.9 41.6 41.2 32.4 31.9 28.4 27.1 26.5 25.2
N
S
O
O
Ph
O
O
Cl
4h
^{13}C NMR 101 MHz, CDCl$_3$
210 190 170 150 130 110 90 80 70 60 50 40 30 20 10 0
f1 (ppm)

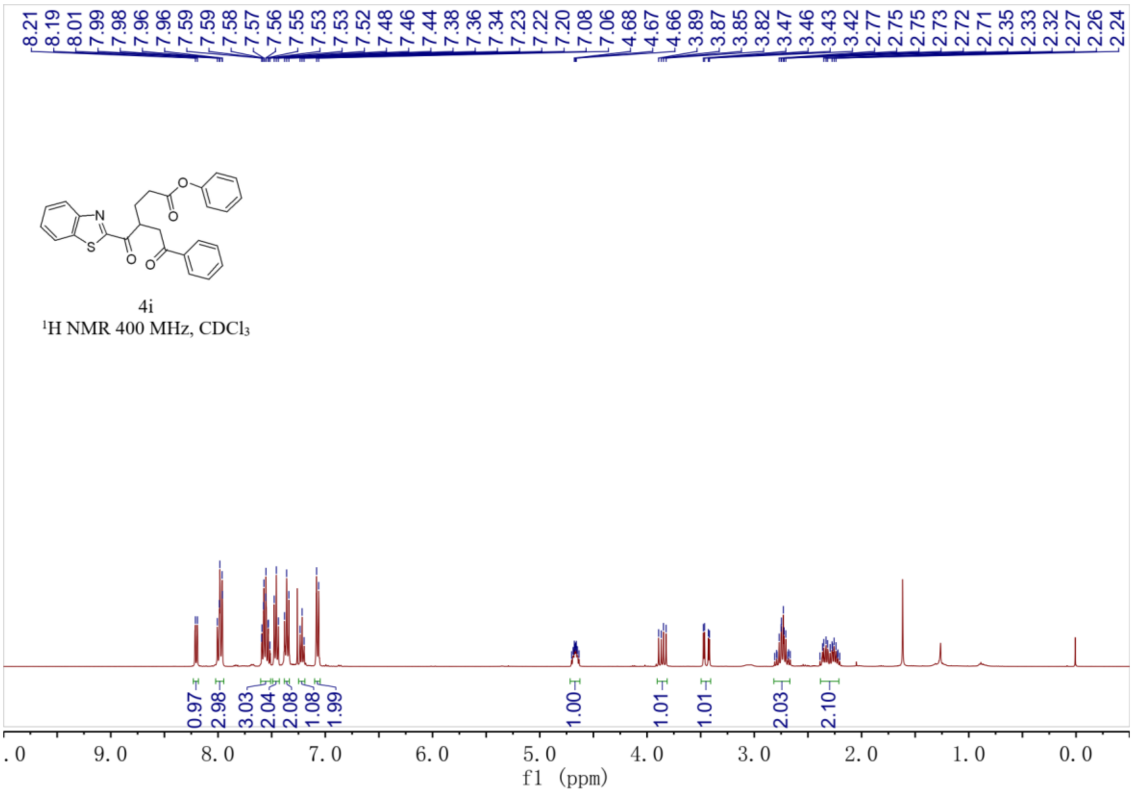
8.21 8.19 8.01 7.99 7.98 7.96 7.96 7.59 7.59 7.58 7.57 7.56 7.55 7.53 7.52 7.48 7.46 7.44 7.38 7.36 7.34 7.23 7.22 7.20 7.08 7.06 4.68 4.67 4.66 3.89 3.87 3.85 3.82 3.47 3.46 3.43 3.42 2.77 2.75 2.73 2.72 2.71 2.35 2.33 2.32 2.27 2.26 2.24
N
S
O
4i
1H NMR 400 MHz, CDCl3
0.97 2.98 3.03 2.04 2.08 1.08 1.99 1.00 1.01 1.01 2.03 2.10
9.0 8.0 7.0 6.0 5.0 4.0 3.0 2.0 1.0 0.0
f1 (ppm)

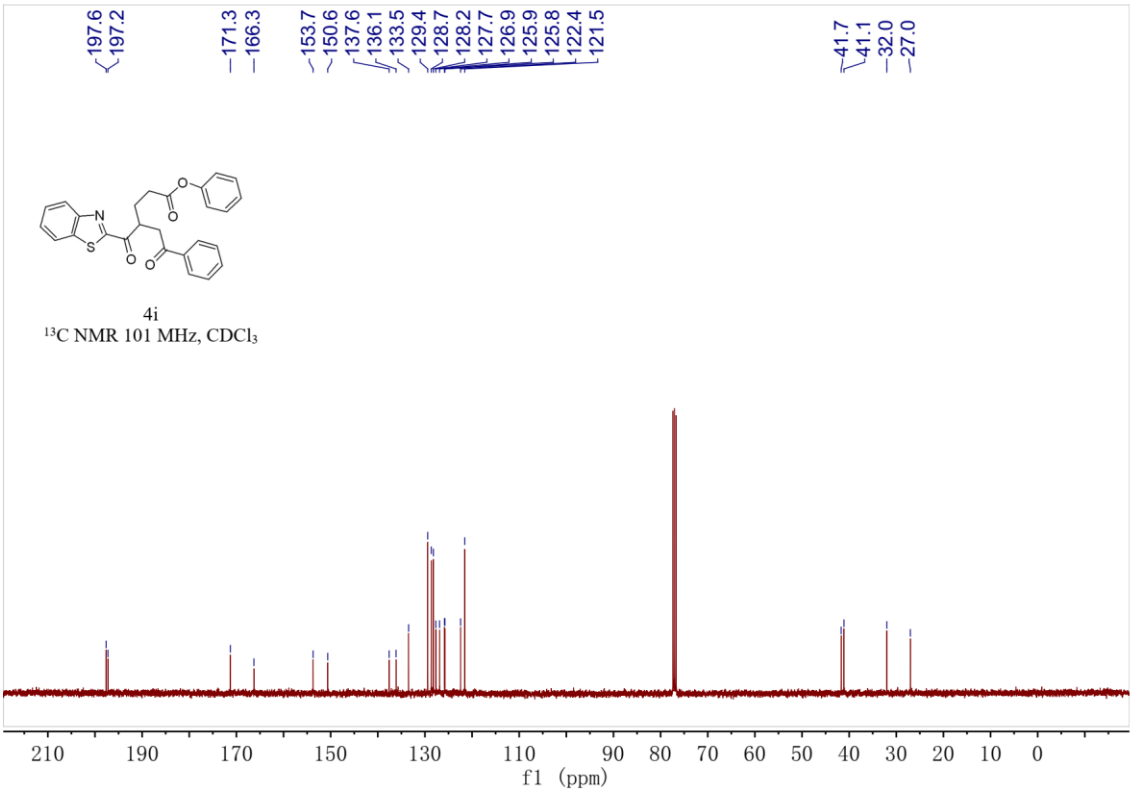
197.6 197.2 171.3 166.3 153.7 150.6 137.6 136.1 133.5 129.4 128.7 128.2 127.7 126.9 125.9 125.8 122.4 121.5 41.7 41.1 32.0 27.0
N
S
O
4i
13C NMR 101 MHz, CDCl3
210 190 170 150 130 110 90 80 70 60 50 40 30 20 10 0
f1 (ppm)

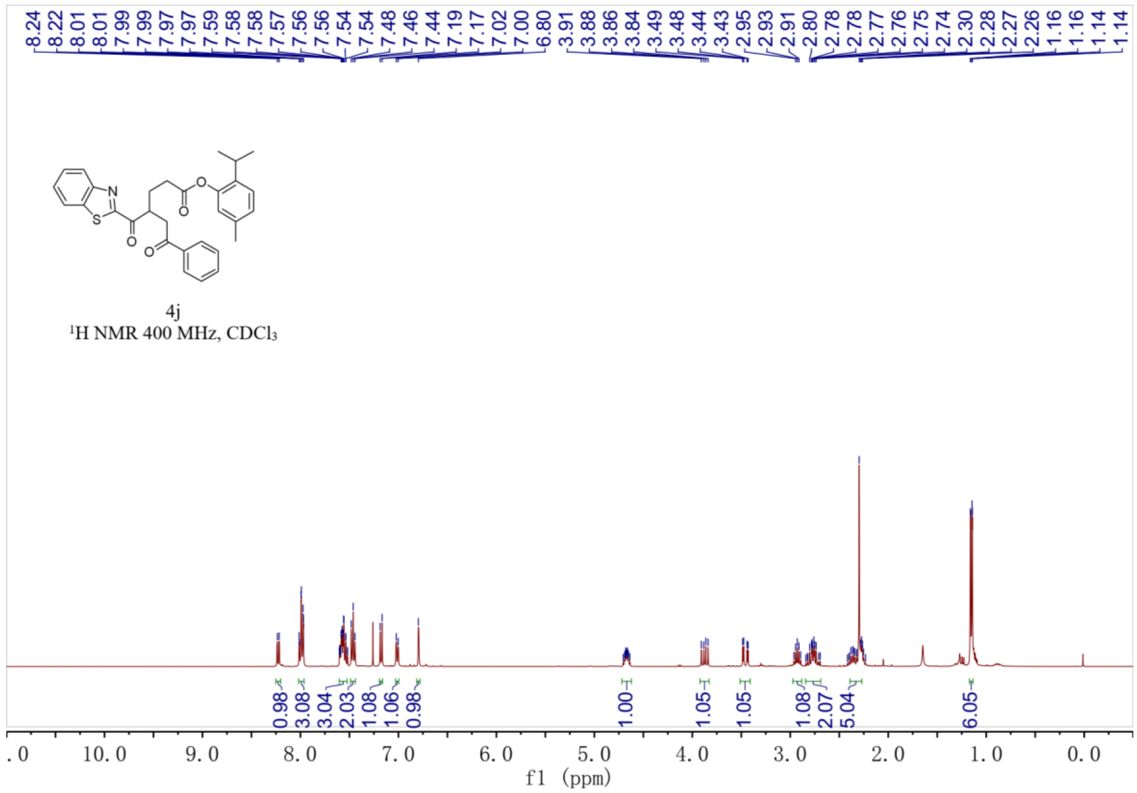
4j
^{1}H NMR 400 MHz, $CDCl_3$
f1 (ppm)

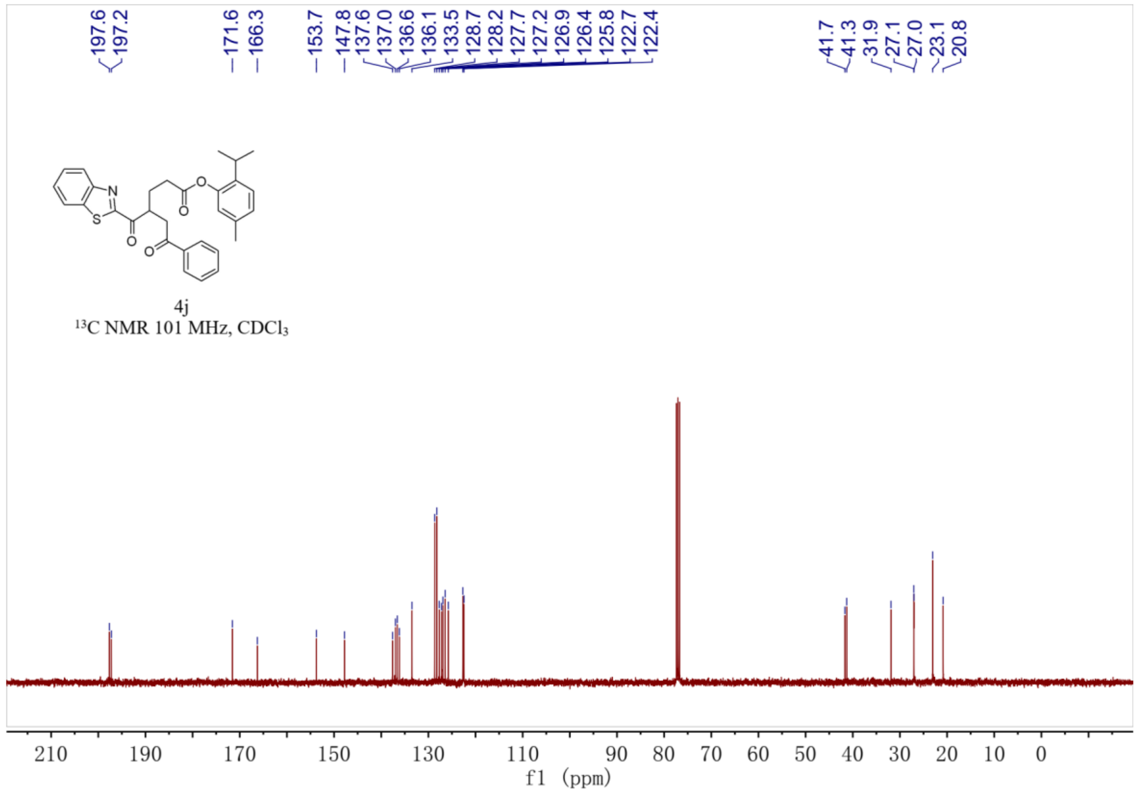
4j
^{13}C NMR 101 MHz, $CDCl_3$
f1 (ppm)

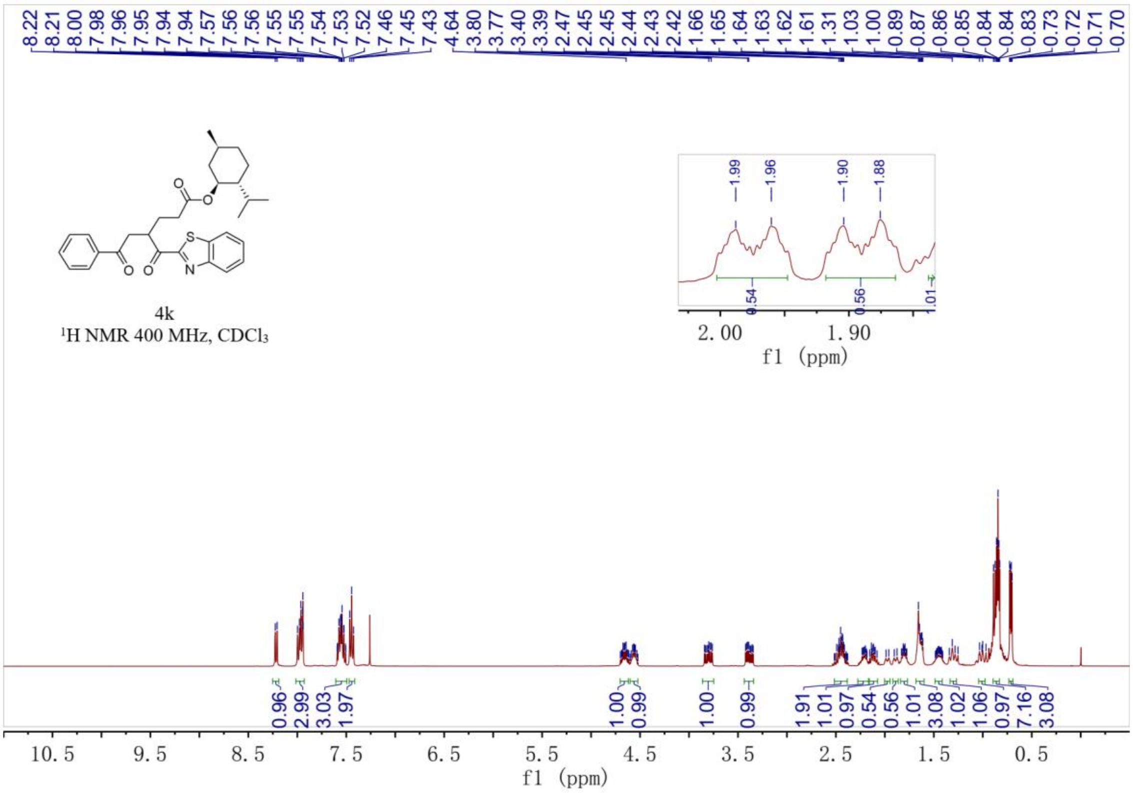
8.22 8.21 8.00 7.98 7.96 7.95 7.94 7.94 7.57 7.56 7.56 7.55 7.55 7.54 7.53 7.52 7.46 7.45 7.43 4.64 3.80 3.77 3.40 3.39 2.47 2.45 2.45 2.44 2.43 2.42 1.66 1.65 1.64 1.63 1.62 1.61 1.31 1.03 1.00 0.89 0.87 0.86 0.85 0.84 0.84 0.83 0.73 0.72 0.71 0.70
S
N
O
4k
^{1}H NMR 400 MHz, CDCl$_3$
1.99 1.96 1.90 1.88
0.54 0.56 1.01
2.00 1.90
f1 (ppm)
0.96 2.99 3.03 1.97 1.00 0.99 1.00 0.99 1.91 1.01 0.97 0.54 0.56 1.01 3.08 1.02 1.06 0.97 7.16 3.08
10.5 9.5 8.5 7.5 6.5 5.5 4.5 3.5 2.5 1.5 0.5
f1 (ppm)

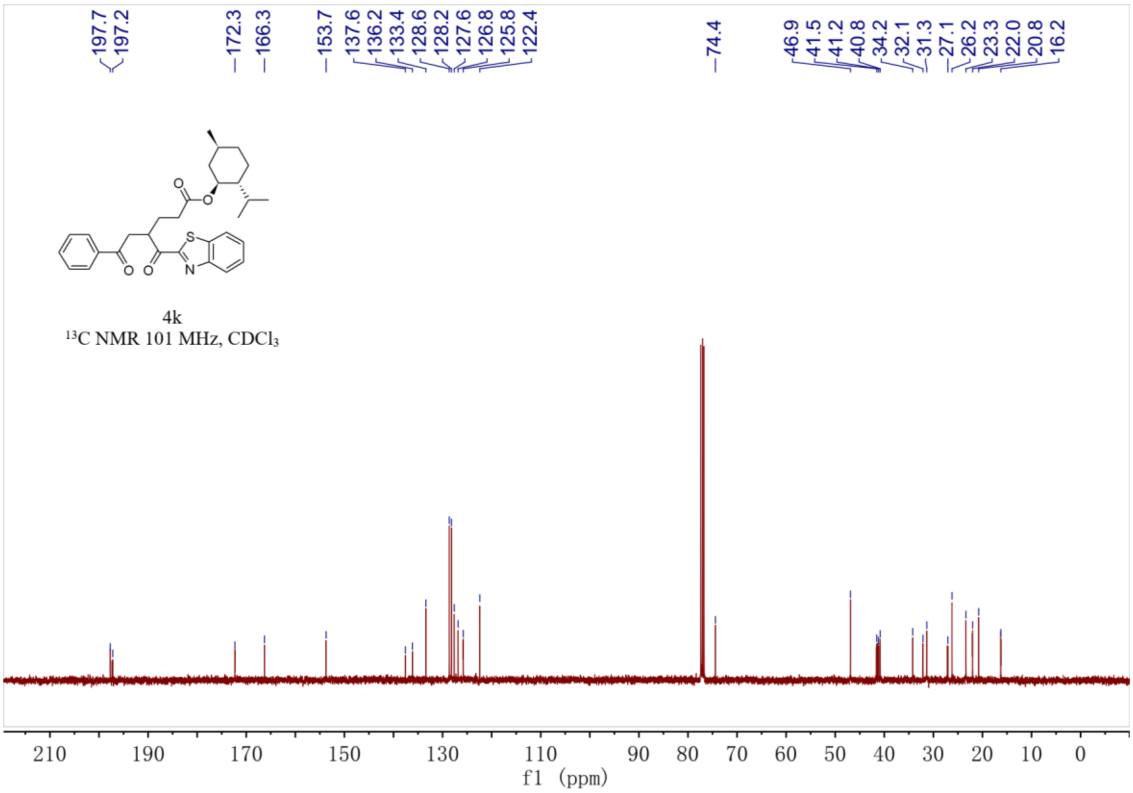
197.7 197.2 172.3 166.3 153.7 137.6 136.2 133.4 128.6 128.2 127.6 126.8 125.8 122.4 74.4 46.9 41.5 41.2 40.8 34.2 32.1 31.3 27.1 26.2 23.3 22.0 20.8 16.2
S
N
O
4k
^{13}C NMR 101 MHz, CDCl$_3$
210 190 170 150 130 110 90 80 70 60 50 40 30 20 10 0
f1 (ppm)

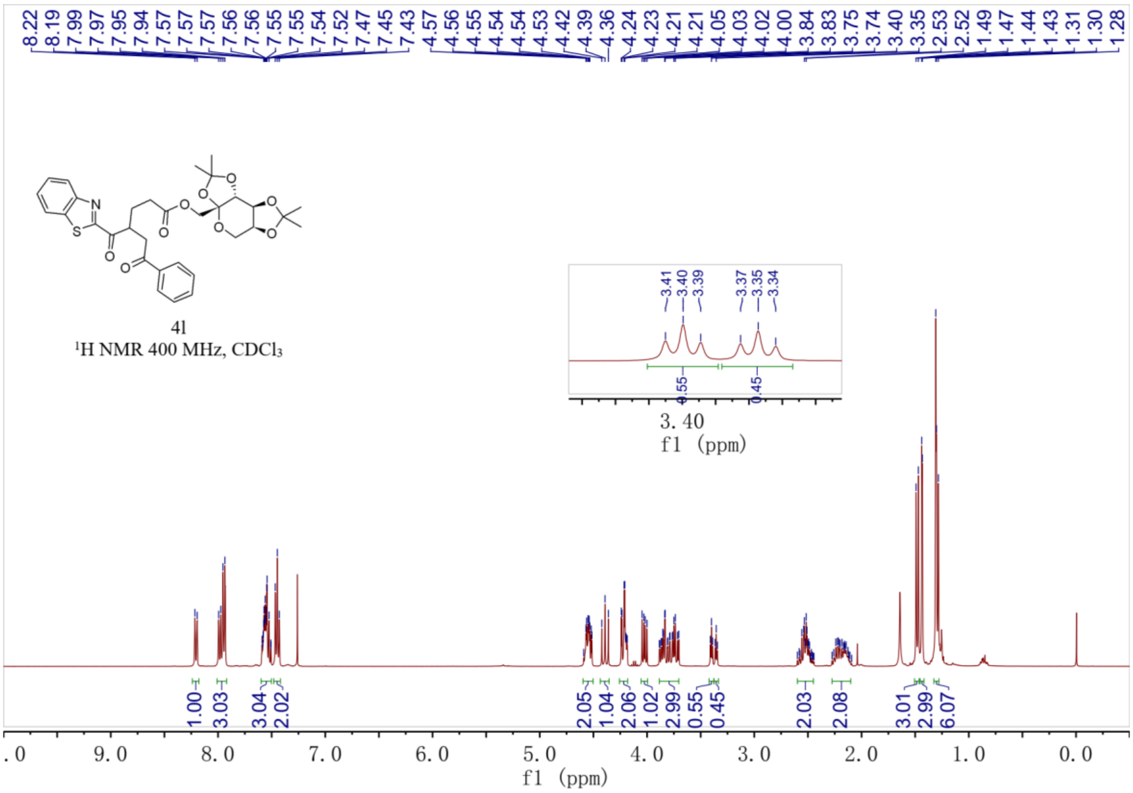

4l

^{1}H NMR 400 MHz, $CDCl_3$

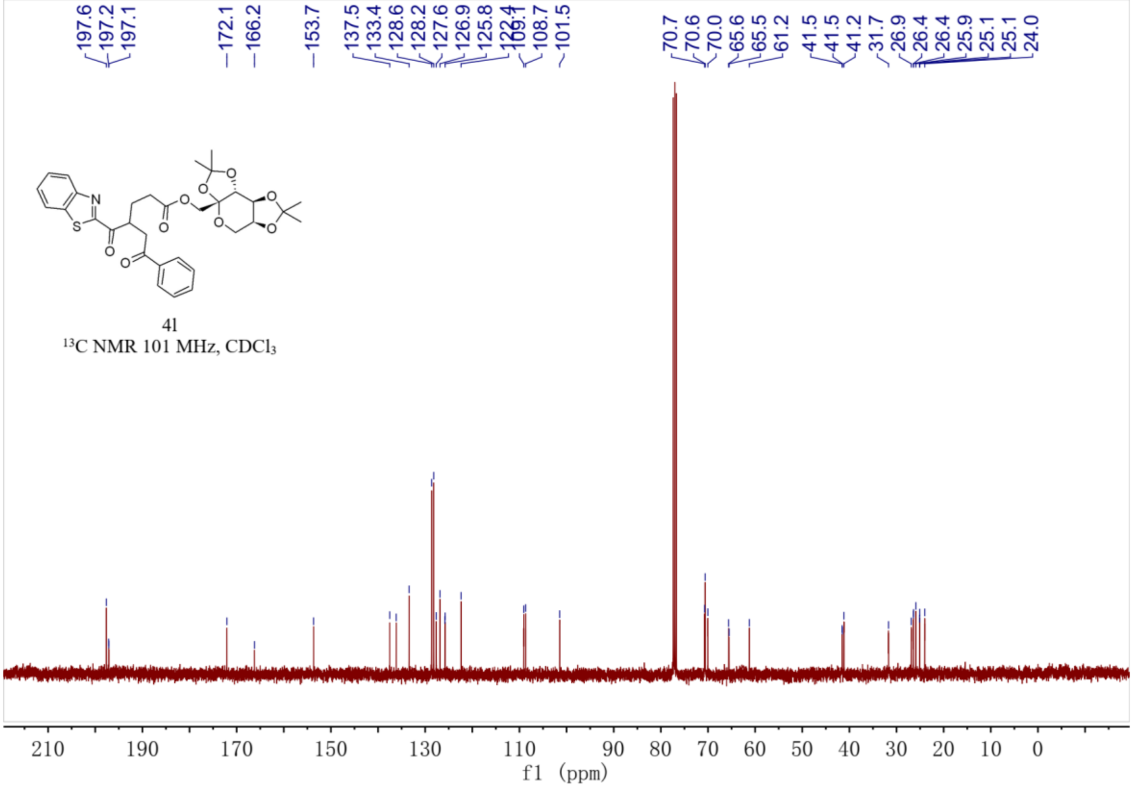

4l

^{13}C NMR 101 MHz, $CDCl_3$

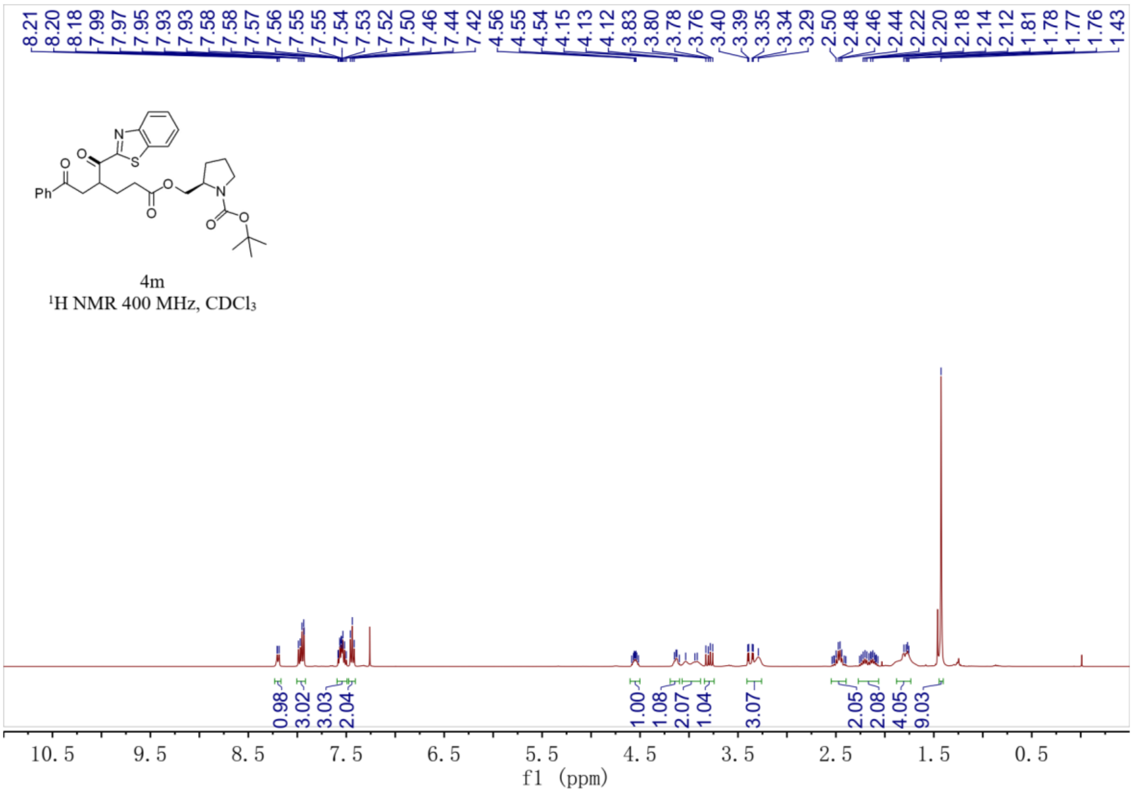
8.21 8.20 8.18 7.99 7.97 7.95 7.93 7.93 7.58 7.58 7.57 7.56 7.55 7.55 7.54 7.53 7.52 7.50 7.46 7.44 7.42 4.56 4.55 4.54 4.15 4.13 4.12 3.83 3.80 3.78 3.76 3.40 3.39 3.35 3.34 3.29 2.50 2.48 2.46 2.44 2.22 2.20 2.18 2.14 2.12 1.81 1.78 1.77 1.76 1.43
4m
^{1}H NMR 400 MHz, CDCl$_3$
0.98 3.02 3.03 2.04 1.00 1.08 2.07 1.04 3.07 2.05 2.08 4.05 9.03
10.5 9.5 8.5 7.5 6.5 5.5 4.5 3.5 2.5 1.5 0.5
f1 (ppm)

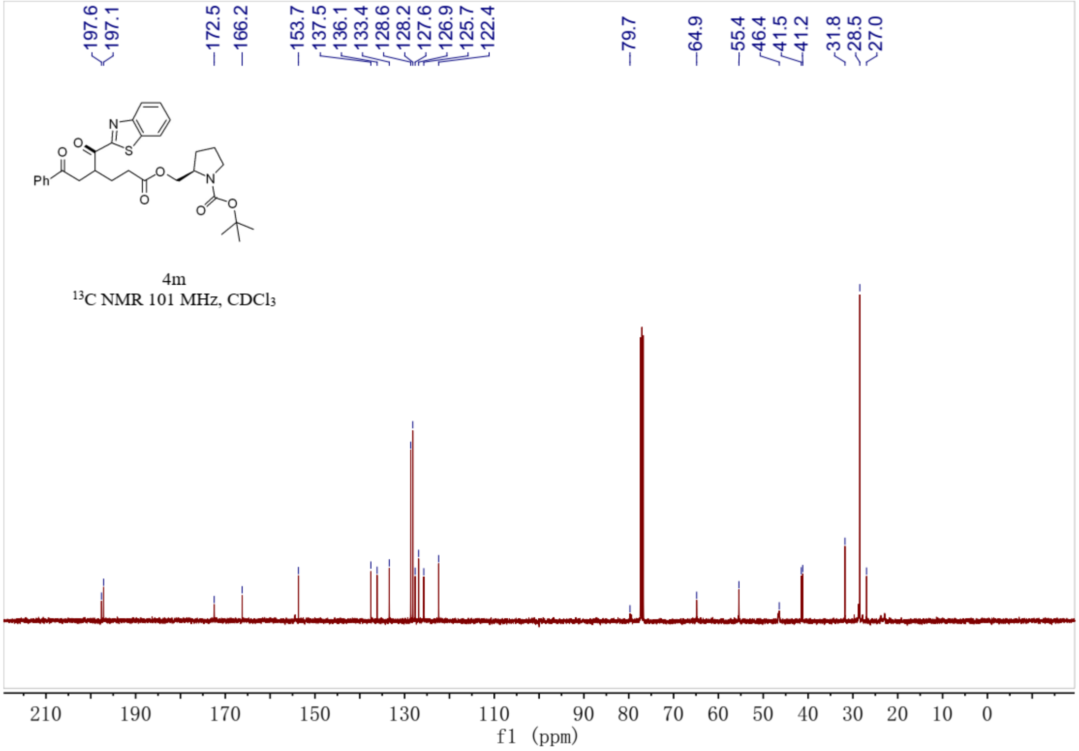
197.6 197.1 172.5 166.2 153.7 137.5 136.1 133.4 128.6 128.2 127.6 126.9 125.7 122.4 79.7 64.9 55.4 46.4 41.5 41.2 31.8 28.5 27.0
4m
^{13}C NMR 101 MHz, CDCl$_3$
210 190 170 150 130 110 90 80 70 60 50 40 30 20 10 0
f1 (ppm)

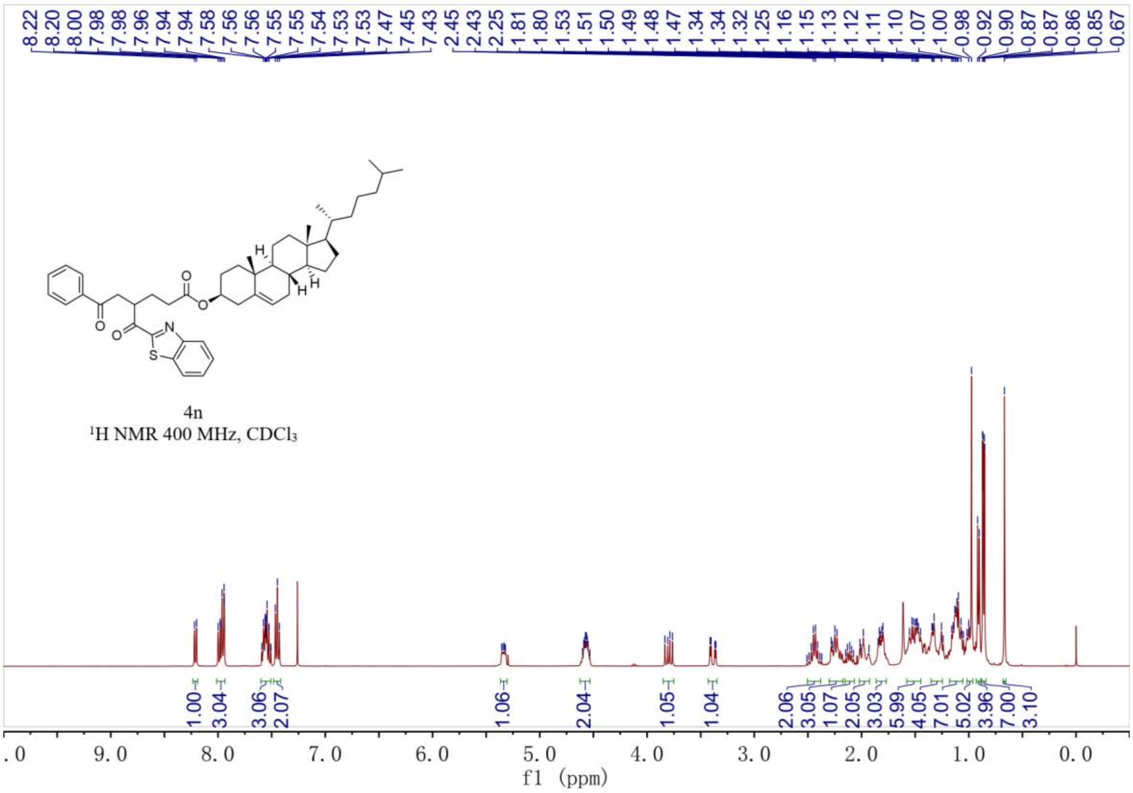
8.22 8.20 8.00 7.98 7.96 7.94 7.94 7.58 7.56 7.56 7.55 7.55 7.54 7.53 7.53 7.47 7.45 7.43 2.45 2.43 2.25 1.81 1.80 1.53 1.51 1.50 1.49 1.48 1.47 1.34 1.32 1.25 1.16 1.15 1.13 1.12 1.11 1.10 1.07 1.00 0.98 0.92 0.90 0.87 0.86 0.85 0.67
4n
^{1}H NMR 400 MHz, $CDCl_3$
1.00 3.04 3.06 2.07 1.06 2.04 1.05 1.04 2.06 3.05 1.07 2.05 3.03 5.99 4.05 7.01 5.02 3.96 7.00 3.10
f1 (ppm)

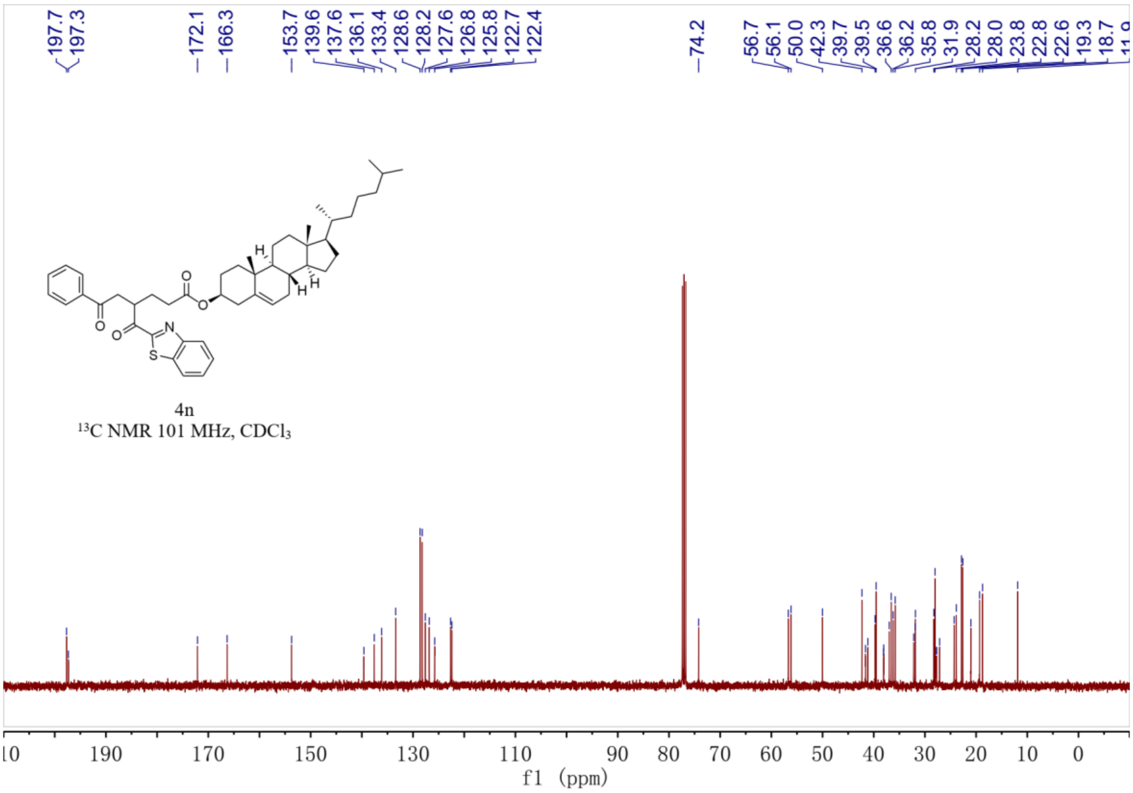
197.7 197.3 172.1 166.3 153.7 139.6 137.6 136.1 133.4 128.6 128.2 127.6 126.8 125.8 122.7 122.4 74.2 56.7 56.1 50.0 42.3 39.7 39.5 36.6 36.2 35.8 31.9 28.2 28.0 23.8 22.8 22.6 19.3 18.7
4n
^{13}C NMR 101 MHz, $CDCl_3$
f1 (ppm)
